# Catalytic Enantioselective Entry to Triflones Featuring a Quaternary Stereocenter

**DOI:** 10.1021/acs.orglett.2c01589

**Published:** 2022-06-10

**Authors:** Francesca Franco, Sara Meninno, Jacob Overgaard, Sergio Rossi, Maurizio Benaglia, Alessandra Lattanzi

**Affiliations:** † Dipartimento di Chimica e Biologia “A. Zambelli”, Università di Salerno, Via Giovanni Paolo II 132, 84084, Fisciano, Italy; ‡ Department of Chemistry, Aarhus University, Langelandsgade 140, 8000 Aarhus, Denmark; § Dipartimento di Chimica, Università degli Studi di Milano, Via Golgi 19, 20133, Milano, Italy

## Abstract

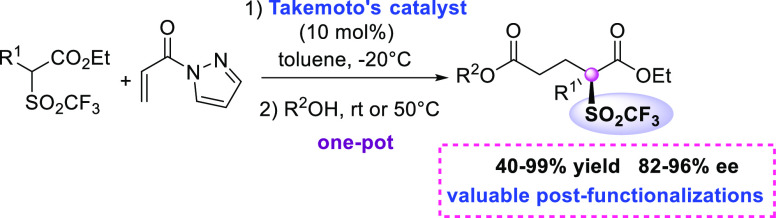

A highly enantioselective one-pot synthesis of functionalized triflones, bearing a quaternary stereocenter, has been developed, exploiting the Michael reaction of α-(trifluoromethylsulfonyl) aryl acetic acid esters with *N*-acryloyl-1*H*-pyrazole catalyzed by commercially available Takemoto’s catalyst, followed by nucleophilic acyl substitution with alcohols. Preliminary investigations highlighted the attractive potential of the triflinate anion as the leaving group for stereocontrolled postfunctionalizations.

Chiral nonracemic sulfones are a class of compounds of great importance in different areas, from organic synthesis, medicinal chemistry to material science. In particular, those bearing the sulfone group directly connected to the stereogenic center are endowed with different biological activities, such as antifungal agents (Agelasidine A),^1^ β-lactamase inhibitors (tazobactan),^2^ and γ-secretase inhibitor.^3^ The sulfonyl group is an accredited bioisoster of the carbonyl group and a strong H-bonding acceptor able to increase the interactions with the biological targets.^4^ Moreover, sulfones are highly useful synthetic building blocks amenable of different transformations.^5^

The asymmetric synthesis of sulfones, having this group directly attached to the stereogenic center is a challenging task,^6^ which has been mainly accomplished via metal-catalyzed substitution,^7^ hydrosulfonylation,^8^ hydrogenation,^9^ and conjugate addition.^10^ However, most of the protocols so far developed are focused on the generation of optically enriched secondary sulfones. In comparison, the stereoselective preparation of aryl and alkyl sulfones featuring a quaternary stereocenter is largely underdeveloped.^6,7,10c^ In this context, scant examples have been reported on the stereoselective preparation of either secondary and tertiary triflones (Scheme 1). Nakamura and Toru illustrated an interesting asymmetric reaction of *n*BuLi generated α-carbanion of benzyl trifluoromethylsulfone with aldehydes in the presence of 30 mol % of bis(oxazoline) ligands (Scheme 1a).^11^ The products were obtained in good to high diastereo- and enantioselectivity.

**Scheme 1:**
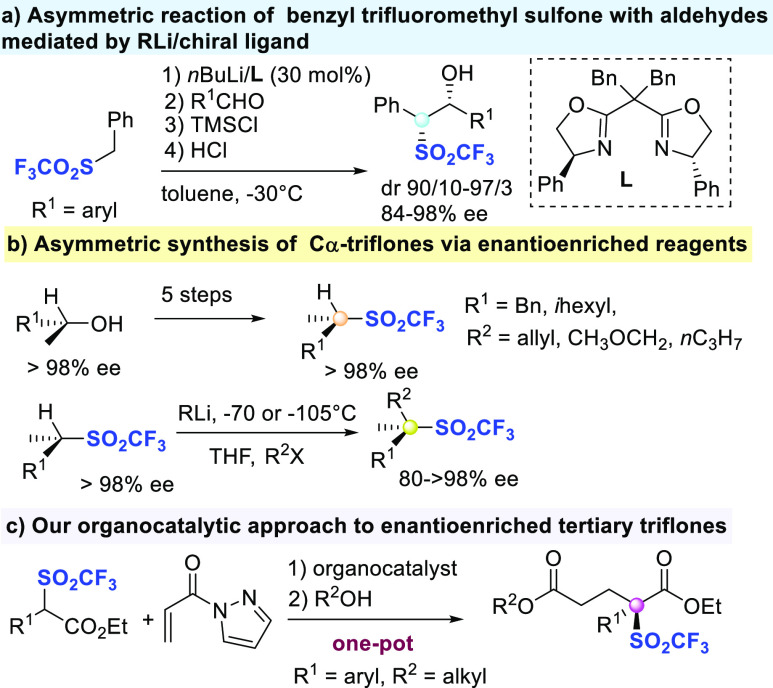
Approaches to Optically Enriched Triflones

Raabe and Gais, developed a five-step sequence from optically enriched secondary alcohols as the reagent to obtain secondary triflones, mantaining the level of enantioselectivity.^12^

The latter were then alkylated, under controlled conditions, to provide triflones with an all-carbon quaternary stereocenter in comparable ee values (Scheme 1b). We recently developed a one-pot α-trifluoromethylthiolation of readily available *N*-acyl pyrazoles, followed by oxidation to access α-trifluoromethansulfonyl aryl acetic acid esters.^13a^ The process has been also improved under continuous flow conditions, starting from carboxylic acids.^14^ The triflyl group is the strongest neutral electron-withdrawing group,^15^ showing mild lipophilicity. This prompted its introduction onto molecular scaffolds, as it affects the activity of fluorinated drugs^16^ and more in general the properties of the materials.^17^ As illustrated in Scheme 1, the asymmetric synthesis of tertiary triflones remains an elusive goal, where catalytic approaches still have to be developed.^18^ Having in hand a viable route to trifluoromethansulfonyl aryl acetic acid esters, we envisaged that they might serve as suitable pronucleophiles^15c,15d^ to employ in Michael reactions under mild organocatalytic conditions. Herein, we report a first catalytic and highly enantioselective preparation of triflones, featuring a quaternary stereocenter. Michael reaction of trifluoromethansulfonyl aryl acetic acid esters with *N*-acryloyl-1*H*-pyrazole has been mediated by Takemoto’s catalyst, followed by nucleophilic acyl substitution with alcohols in one pot. The final bis-ester triflones also demonstrated to be useful compounds for interesting postfunctionalizations.

At the ouset of the study, methyl vinyl ketone was reacted with compound **1a**, using readily available bifunctional organocatalysts at 20 mol % loading, in toluene at room temperature (Table 1). Pleasingly, quinidine (**QD**) catalyzed the conjugate addition, providing product **3a** in 82% yield and 20% ee (entry 1). This result prompted us to use Cinchona alkaloids-derived thiourea **eQNT** and **eQDT**, which unfortunately were much less effective promoters (entries 2 and 3). Sterically hindered amine-thiourea **4** gave a small improvement in the enantioselectivity up to 37% ee (entry 4). Takemoto’s catalyst **5** proved to be more active, leading to **3a** in 75% yield and 55% ee, after a short reaction time (entry 5). Next, phenyl vinyl ketone was treated with compound **1a** using catalyst **5**, observing the formation of the adduct **3b** with an increased level of enantioselectivity (entry 6). Readily available amine-thiourea **6** was then checked in the process, giving disappointing results (entry 7), as well as the commercially available squaramide **7**, which afforded racemic **3b** in only moderate yield (entry 8). When more sterically hindered isopropyl ester **1a′** (R^1^ = 4-BrC_6_H_4_) was reacted, a decreased level of enantioselectivity was observed (entry 9). For the purpose of improving the enantiocontrol, 1-naphthyl vinyl ketone was employed with **1a** in the presence of catalyst **5** (entry 10). However, the adduct **3d** was isolated in 85% yield and 44% ee. Activated acrylic acid derivatives were then checked, such as the 1,1,1,3,3,3-hexafluoroisopropyl acrylate, but it proved to be poorly reactive (entry 11). Given the utility displayed over recent years by α,β-unsaturated *N*-acyl pyrazoles in asymmetric catalysis,^19^ the corresponding *N*-acryloyl-1*H*-3-phenyl pyrazole was reacted under the optimized conditions (entry 12). Pleasingly, it was smoothly converted into the corresponding adduct **3e**,^20^ which was isolated in 42% yield and 75% ee. The same reaction when conducted at −20 °C afforded product **3e** with improved 86% ee (entry 13). Finally, when using *N*-acryloyl-1*H*-pyrazole as the acceptor, adduct **3f** was recovered in 50% yield^20^ and 89% ee (entry 14). Reduction of the catalyst loading to 10 mol % as well as the temperature as low as −20 °C enabled the product to be satisfactorily obtained in high yield^21^ and 94% ee (entries 15 and 16).

**Table 1:**
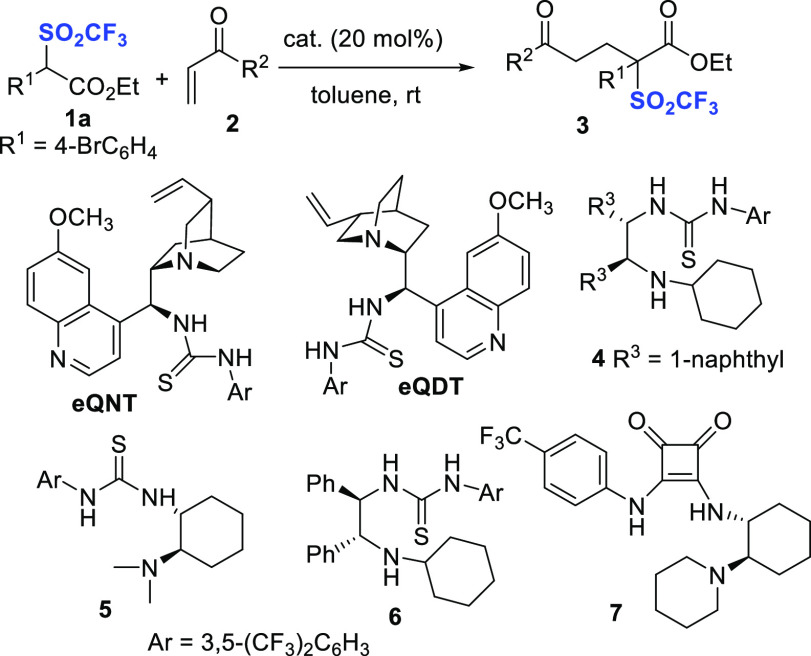
Reaction Optimization^a^

entry	cat.	R^2^	*t* (h)	**3** yield (%)^b^	**3** ee^c^
1	**QD**	Me **2a**	7	82 (**3a**)	20
2	**eQNT**	Me	23	10 (**3a**)	5
3	**eQDT**	Me	23	57 (**3a**)	*rac*
4	**4**	Me	23	41 (**3a**)	37
5	**5**	Me	6	75 (**3a**)	55(−)
6	**5**	Ph **2b**	16	79 (**3b**)	67
7	**6**	Ph	16	44 (**3b**)	37
8	**7**	Ph	16	23 (**3b**)	*rac*
9^d^	**5**	Ph	17	76 (**3c**)	63
10	**5**	1-naphthyl **2c**	17	85 (**3d**)	44
11	**5**	OCH(CF_3_)_2_**2d**	25	<10	n.d.
12	**5**	3-Phpyrazole **2e**	17	42 (**3e**)	75
13^e^	**5**	3-Phpyrazole **2e**	40	61 (**3e**)	86
14	**5**	pyrazole **2f**	17	50 (**3f**)	89
15^f^	**5**	pyrazole **2f**	24	90 (**3f**)	93
16^g^	**5**	pyrazole **2f**	24	95 (**3f**)	94

a Reactions performed at 0.1 mmol scale of **1a** (*C* 0.2 M) using **2** (1.2 equiv).

b Isolated yield after chromatography.

c Determined by chiral HPLC analysis; n.d. = not determined. Negative sign indicates enantiomeric excess for the opposite enantiomer.

d The isopropyl ester of compound **1a** was used.

e Run at −20 °C.

f 10 mol % of **5** was used at 0 °C.

g 10 mol % of **5** was used at −20 °C.

*N*-Acyl pyrazoles behave as useful carboxylic acid ester surrogates,^19^ due to the good leaving group ability of the pyrazole group. Hence, we thought to develop a simple one-pot methodology to directly obtain the bis-ester triflones **8**, treating compounds **3** with an alcohol at room temperature, after the end of the conjugate addition step. Under the optimized reaction conditions reported in Scheme 1, entry 16, the scope of the one-pot process was next investigated (Scheme 2). As illustrated in Scheme 2, triflones **1**, bearing halogens at *para*- and *ortho*-position of the phenyl ring, were converted into the corresponding methyl esters **8a**–**d** in excellent yields and high enantioselectivity (82–95% ee).

**Scheme 2:**
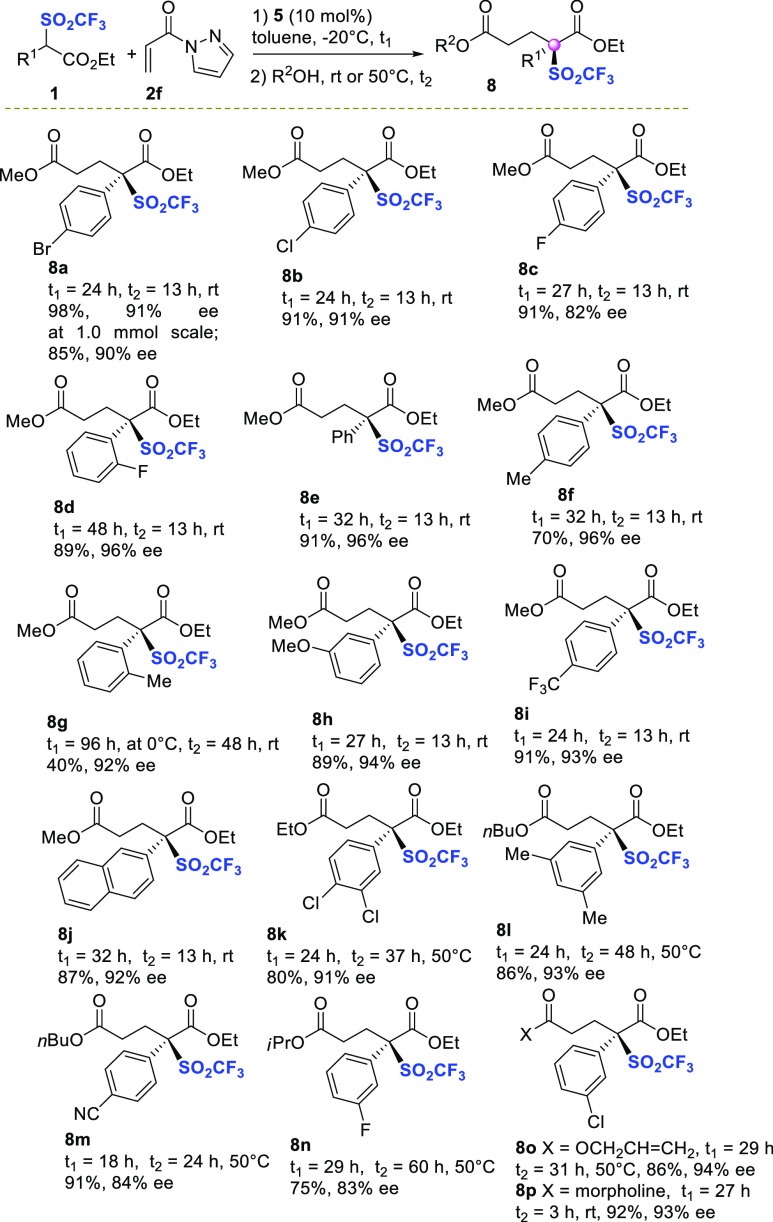
Substrate Scope of the One-Pot Process^,^^,^ First step: 0.1 mmol scale of **1** (C 0.2 M) using **2f** (1.5 equiv). Second step: addition of R^2^OH (50 equiv), in case of morpholine (3 equiv). Isolated yield after chromatography. Ee determined by chiral HPLC analysis.

Pleasingly, more sterically encumbered *ortho*-fluoro derivative **8d** was isolated with excellent ee value (95%). Electron-donating or -withdrawing substitution at the *para-*, *meta*-, and *ortho-* positions, including the phenyl and 2-naphthyl moieties, were well tolerated, as the products **8e**–**j** were recovered in good to high yields and 91–96% ee values. Only the sterically demanding *ortho*-methyl derivative **8g** was isolated in 40% yield, although a 96% ee value was observed. Then, we surveyed the suitability of other alcohols as nucleophiles in the second step on differently substituted trifluoromethansulfonyl phenyl acetic acid esters **1**. Ethanol, *n*-butanol to more sterically hindered isopropanol and allylic alcohol could be employed, performing the esterification at 50 °C. The corresponding triflones **8k**–**o**, bearing single or double substitution at the phenyl ring, have been obtained in fairly good to high yields and ee values (83–94%).

Finally, the pyrazole displacement with morpholine performed at room temperature led to the corresponding product **8p**, bearing a tertiary amide group, in 92% yield and 93% ee. The practicality of the process was investigated scaling-up reagent **1a** to 1.0 mmol. Triflone **8a** was isolated maintaining a high yield and enantioselectivity. Further experiments, carried out during the preparation of tertiary amide **8p**, allowed us to disclose a synthetically appealing derivatization, involving triflyl group displacement (Scheme 3).

**Scheme 3:**
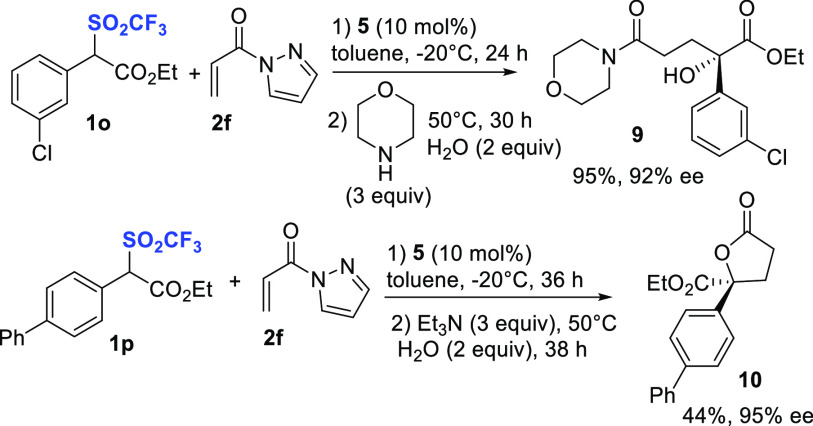
One-Pot Derivatizations of Compounds **8** Involving Triflyl Group Displacement

When the second step was performed using morpholine in the presence of water (2 equiv) at 50 °C, for a prolonged reaction time, the α-hydroxyl ester **9** was efficiently formed in 95% yield and 92% ee. This remarkable result would be rationalized invoking S_N_2 displacement of the triflinate anion, which is an excellent leaving group,^22^ by an in situ generated hydroxyl anion.^23^ The transformation is noteworthy, being a formal enantioselective hydroxylation at a congested α-position of an ester. The absolute configuration of compound **9** was determined to be *S* by X-ray crystallographic analysis (CCDC No.: 2165089).

Consequently, compounds **8** were assigned as *R*-configured, which was found to be consistent with DFT calculations (Figure 1).

**Figure 1:**
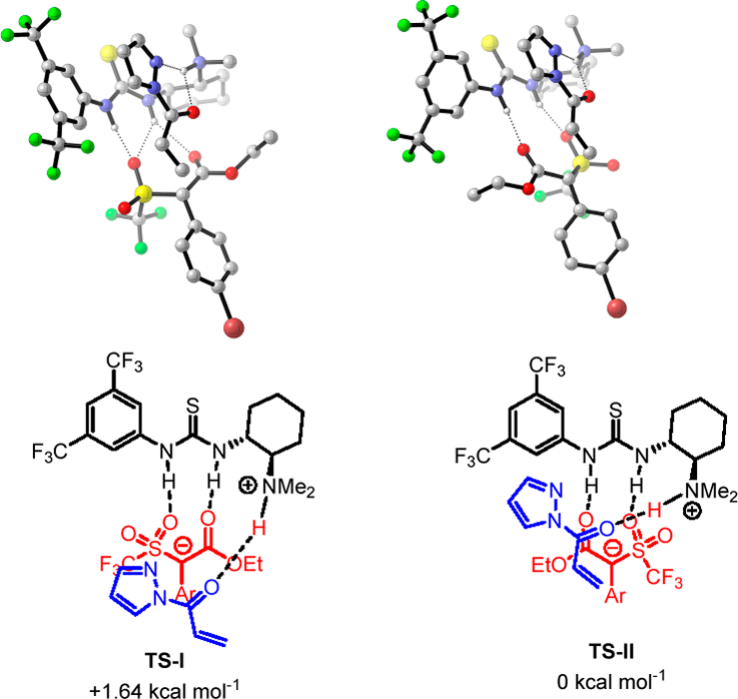
Proposed model of stereoselection. Geometries and ΔΔ*G*^0^ (kcal mol^–1^) of transition states related to the synthesis of enriched triflone **8a** were calculated at the M062X/6-31G(d,p)/PCM (toluene) level of theory. Hydrogens are omitted for clarity.

The transition states leading to the formation of both (*R*)-**8a** and (*S*)-**8a** for the Michael reaction, promoted by (*R,R*)-catalyst **5**, were fully optimized by DFT calculation at the M062X-631g(d,p)/PCM (toluene) level of theory using the M062X functional.^24^ Different models of substrates-catalyst coordination were investigated.^25^ The energetically most affordable calculated transition states would involve the activation model previously proposed by Papai,^26^ where triflone **1a** is coordinated to the thiourea group of the catalyst and acyl pyrazole **2f** to the tertiary amine group. According to this model, **TS-II** leading to product (*R*)-**8a** was found to be more stable by 1.64 kcal mol^–1^ than **TS-I** leading to product (*S*)-**8a**, which features weaker hydrogen bonding of thiourea with the SO_2_CF_3_ group, and, possibly, a destabilizing interaction between SO_2_CF_3_ and one of the CF_3_ residues in the catalyst. Noteworthy, remarkably good agreement between calculated (91% ee) and experimental (93% ee in entry 15, Table 1) ee values has been achieved.

We further applied the displacement to develop an asymmetric one-pot Michael/S_N_2 displacement/esterification to γ-butyrolactone, bearing a γ-quaternary stereocenter (Scheme 3). The in situ generated adduct **8p** was treated with Et_3_N, water at 50 °C, affording the expected lactone **10** in 44% yield and 95% ee. Although the process needs to be optimized, it represents an interesting and useful application of optically active triflones **8** as intermediates toward difficult to prepare γ-butyrolactones **10**.^27^

Additional postfunctionalizations on representative compound **8a** have been performed under reductive conditions (Scheme 4). Unexpectedly, treatment with DIBALH under controlled conditions afforded alcohol **11** in 76% yield, without erosion of the ee value. Reduction of the less activated methyl ester might be likely ascribed to the congested nature of the ethyl ester portion. Having ascertained that selective reduction of the ester to aldehyde occurred in a shorter reaction time, a one-pot process from reagent **1a**, involving asymmetric Michael reaction/reduction to aldehyde/Horner–Emmons olefination, has been developed. The diversely functionalized product **12** was recovered in satisfactory 50% overall yield and 93% ee.

**Scheme 4:**
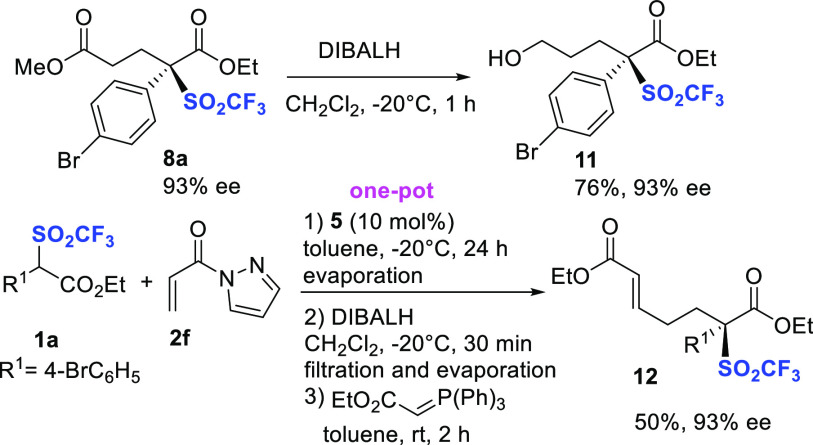
Additional Derivatizations of Compounds **8**

In summary, we successfully develop a first enantioselective catalytic route to triflones, featuring a quaternary stereocenter. The asymmetric Michael reaction between α-(trifluoromethylsulfonyl) aryl acetic acid esters with *N*-acryloyl-1*H*-pyrazole was efficiently catalyzed by commercial Takemoto’s catalyst, followed by nucleophilic acyl substitution with alcohols. The bis-ester triflones were obtained in good to excellent yields and high enantioselectivity in one-pot. Moreover, this work provides useful knowledge on the application of tertiary triflones in stereoselective organic synthesis. The utility of the products has been demonstrated via triflone displacement and under reductive conditions to conveniently access a variety of attractive enantioenriched scaffolds.

## Supporting Information Available

Full experimental procedures, characterization data, NMR spectra, HPLC traces, computational details and crystallographic data for **9** are available. (PDF)

## Accession Codes

CCDC 21065089 contains the supplementary crystallographic data for this paper. These data can be obtained free of charge via www.ccdc.cam.ac.uk/data_request/cif, or by emailing data_request@ccdc.cam.ac.uk, or by contacting The Cambridge Crystallographic Data Centre, 12 Union Road, Cambridge CB2 1EZ, UK; fax: +44 1223 336033.
