# Supplementary material for: Catalytic Enantioselective Entry to Triflones Featuring a Quaternary Stereocenter
Source: Org Lett. 2022 Jun 10;24(24):4371–6. doi: 10.1021/acs.orglett.2c01589 (PMC9490835; doi:10.1021/acs.orglett.2c01589)
Supplement: Supplementary file 1 — ol2c01589_si_001.pdf [file ol2c01589_si_001.pdf]

Supporting Information

Catalytic Enantioselective Entry to Triflones Featuring a Quaternary Stereocenter

Francesca Franco,‡ Sara Meninno,‡ Jacob Overgaard,† Sergio Rossi,‖ Maurizio Benaglia,‖ Alessandra Lattanzi*,‡
e-mail: lattanzi@unisa.it

‡*Dipartimento di Chimica e Biologia "A. Zambelli", Università di Salerno, Via Giovanni Paolo II 132, 84084, Fisciano, Italy*
†*Department of Chemistry, Aarhus University, Langelandsgade 140, 8000 Aarhus, Denmark*
‖*Dipartimento di Chimica, Università degli Studi di Milano, Via Golgi 19, 20133, Milano, Italy*

Table of contents

General Methods 2

Experimental Procedures and Compounds Characterization 3

Table 1. Solvent screening 3

General synthesis of α-trifluoromethylsulfonyl esters 3

Synthesis of acrylpyrazoles (**2e-f**) 9

General procedure for racemic Michael reaction of vinyl ketones 10

General procedure for enantioselective Michael reaction of vinyl ketones 10

General procedure for racemic Michael reaction of α,β-unsaturated pyrazoleamides 13

General procedure for enantioselective Michael reaction of α,β-unsaturated pyrazoleamides 13

General procedure for racemic Michael reaction and one-pot derivatization 15

General procedure for enantioselective Michael reaction and one-pot derivatization 15

Scale-up of model one-pot reaction on compound **8a** 16

Procedure for asymmetric synthesis of α-hydroxyl ester 23

Procedure for the asymmetric synthesis of γ-butyrolactone 24

Table 2. Optimization of the ester reduction 25

Procedure for reduction of enantioenriched **8a** to alcohol 25

Procedure for enantioselective one-pot Michael addition/reduction/Horner-Emmons olefination 26

X-Ray Data for the Absolute Configuration Assignment of Compound 9 28

Computational Details 30

NMR Spectra 110

HPLC Chromatograms 195

General Methods

All reactions requiring dry or inert conditions were conducted in flame-dried glassware under a positive pressure of nitrogen. Anhydrous toluene was purchased from Aldrich and used as received, all other solvents were dried over molecular sieves. Molecular sieves (Aldrich Molecular Sieves, 3 Å, 1.6 mm pellets) were activated under vacuum at 200 °C overnight. Reactions were monitored by thin layer chromatography (TLC) on Macherey-Nagel pre-coated silica gel plates (0.25 mm) and visualized by UV light. Flash chromatography was performed on Merck silica gel (60, particle size: 0.040–0.063 mm). ^{1}H NMR ^{13}C NMR and ^{19}F NMR spectra were recorded on Bruker Avance III HD 600, Bruker Avance-400 or Bruker Avance-300 spectrometer in $CDCl_3$ and CD_2Cl_2 as solvents at room temperature. Chemical shifts for protons are reported using residual solvent protons (^{1}H NMR: δ = 7.26 ppm for $CDCl_3$) as internal standard. Carbon spectra were referenced to the shift of the ^{13}C signal of $CDCl_3$ (δ = 77.16 ppm) and CD_2Cl_2 (δ = 53.84).

The following abbreviations are used to indicate the multiplicity in NMR spectra: s - singlet; d - doublet; t - triplet; q - quartet; quint - quintuplet; dd - double doublet; ddd - doublet of doublet of doublets; dq - doublet of quartets; qd - quartet of doublets; m - multiplet; sept - septet; bs - broad signal.

Optical rotation of compounds was performed on a Jasco P-2000 digital polarimeter using WI (Tungsten-Halogen) lamp (λ = 589 nm). High resolution mass spectra (HRMS) were acquired using a Bruker solariX XR Fourier transform ion cyclotron resonance mass spectrometer (Bruker Daltonik GmbH, Bremen, Germany) equipped with a 7T refrigerated actively-shielded superconducting magnet. The samples were ionized in positive ion mode using a ESI ionization sources. Melting points were measured with a Stuart Model SMP 30 melting point apparatus and are uncorrected.

Petrol ether (PE) refers to light petroleum ether (boiling point 40-60 °C). All starting materials (unless otherwise noted) were purchased from Aldrich or TCI and used as received.

Reagent **1a-1p** and **1a’** were prepared according to the literature.[1] **2b-f** are known compounds, they were prepared according to the literature.[2,3]

[1] F. Franco, S. Meninno, M. Benaglia, A. Lattanzi, *Chem. Commun*., **2020**, 56, 3073.
[2] A. Bugarin, K. D. Jones, B. T. Connell, *Chem. Commun.* **2010**, *46*, 1715.
[3] C. Ye, S. Chen, F. Han, X. Xie, S. Ivlev, K. N. Houk, E. Meggers, *Angew. Chem. Int. Ed.* **2020**, *59*, 13552.

Experimental Procedures and Compounds Characterization

Table 1. Solvent screening

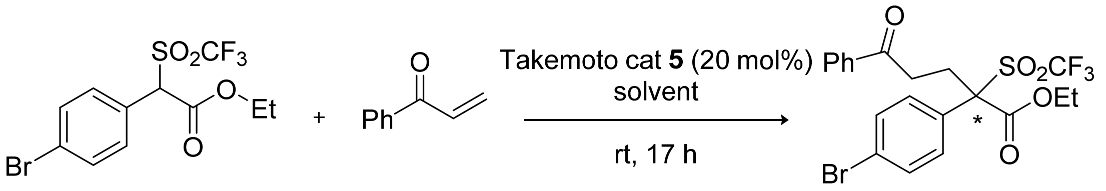

1a **2b** **3b**

Es.	solvent	Yield (%)	ee (%) [a]
1[b]	toluene	84	68
2[c]	toluene	71	71
3[d]	toluene	65	75
4	DCM	85	53
5	hexane	75	51
6	trifluorotoluene	86	56

Unless otherwise noted reactions were conducted with **1a** (0.1 mmol), **2b** (0.12 mmol), (R,R)-Takemoto catalyst **5** (0.02 mmol), in anhydrous solvent (0.5 mL) under nitrogen atmosphere. [a] Determined by chiral HPLC analysis on isolated product. [b] Reaction performed at 0°C. [c] Reaction performed at -30°C. [d] Reaction performed at -30°C and at C = 0.1 M.

General synthesis of α-trifluoromethylsulfonyl esters

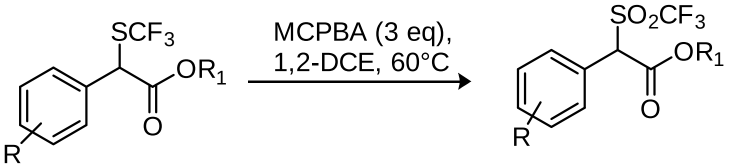

1a-o

In an oven-dried vial *α*-trifluoromethylthio derivative (0.5 mmol), MCPBA (336.2 mg, 1.5 mmol) and anhydrous 1,2-dichlorethane (2.5 mL) were introduced. The reaction mixture was stirred at 60°C for 16 hours by using an oil bath. After completion of the reaction, PPh_3 (131.1 mg 0.5 mmol) was added and the mixture was stirred for 30 min and then extracted with EtOAc and washed with $NaHCO_3$ (x3) and brine. The organic layer was dried over Na_2SO_4, filtered and evaporated. The crude mixture was purified by flash chromatography (eluent: hexane/ethyl acetate 100/0 to 90/10) to afford products **1a-p, 1a'** in 47-98% yield.

Ethyl 2-(4-bromophenyl)-2-((trifluoromethyl)sulfonyl)acetate (1a)

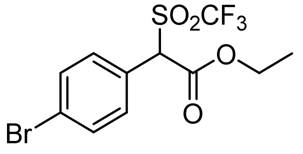

White solid (flash chromatography eluent: hexanes/EtOAc, 98/2 to 90/10), 150.1 mg, 80% yield. **mp** 60.2-61.7 °C. **^{1}H NMR** ($CDCl_3$, 400 MHz): δ 7.61 (d, 2H, J = 8.5 Hz), 7.49 (d, 2H, J = 8.5 Hz), 5.26 (s, 1H), 4.37 (dq, 1H, J = 14.3 Hz, J = 7.2 Hz), 4.31 (dq, 1H, J = 14.3 Hz, J = 7.2 Hz), 1.33 (t, 3H, J = 7.2 Hz). **^{13}C NMR** ($CDCl_3$, 100 MHz): δ 161.2, 132.6, 132.2, 125.7, 124.6, 119.6 (q, $^1J_{CF}$ = 329.9 Hz), 69.6, 63.9, 13.8. **^{19}F NMR** ($CDCl_3$, 376 MHz): δ -73.4. **HRMS (MALDI)** m/z: $[M+H]^+$ Calcd for $C_{11}H_{10}BrF_3O_4S$ 374.9435; found 374.9416. The spectral data of the compound matched with the literature.[1]

Isopropyl 2-(4-bromophenyl)-2-((trifluoromethyl)sulfonyl)acetate (1a')

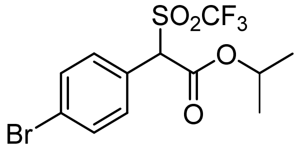

White solid (flash chromatography eluent: hexanes/EtOAc, 98/2 to 90/10), 140.1 mg, 72% yield. **mp** 39.7-40.5 °C. **^{1}H NMR** ($CDCl_3$, 600 MHz): δ 7.60 (d, 2H, J = 8.6 Hz), 7.49 (d, 2H, J = 8.6 Hz), 5.22 (s, 1H), 5.16 (sept, 1H, J = 6.4 Hz), 1.34 (d, 3H, J = 6.4 Hz), 1.29 (d, 3H, J = 6.4 Hz). **^{13}C NMR** ($CDCl_3$, 150 MHz): δ 161.5, 132.3, 125.8, 123.1, 124.6, 119.9 (q, $^1J_{CF}$ = 329.9 Hz), 72.6, 70.0, 21.6, 21.5. **^{19}F NMR** ($CDCl_3$, 376 MHz): δ -73.4. **HRMS (MALDI)** m/z: $[M+H]^+$ Calcd for $C_{12}H_{13}BrF_3O_4S$ 388.9670; found 388.9676.

Ethyl 2-(4-chlorophenyl)-2-((trifluoromethyl)sulfonyl)acetate (1b)

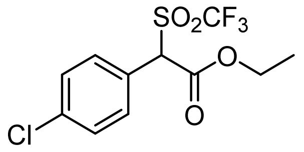

Colourless oil (flash chromatography eluent: hexanes/EtOAc, 98/2 to 90/10), 115.7 mg, 70% yield. **^{1}H NMR** ($CDCl_3$, 300 MHz): δ 7.57 (d, 2H, J = 8.6 Hz), 7.44 (d, 2H, J = 8.6 Hz), 5.31 (s, 1H), 4.37 (dq, 1H, J = 13.4 Hz, J = 7.2 Hz), 4.30 (dq, 1H, J = 13.4 Hz, J = 7.2 Hz), 1.32 (t, 3H, J = 7.2 Hz). **^{13}C NMR** ($CDCl_3$, 75 MHz): δ 162.1, 137.5, 132.1, 129.7, 123.0, 119.9 (q, $^1J_{CF}$ = 330.6 Hz), 69.7, 64.0, 13.9. **^{19}F NMR** ($CDCl_3$, 282 MHz): δ -73.5. **HRMS (MALDI)** m/z: $[M+H]^+$ Calcd for $C_{11}H_{11}ClF_3O_4S$ 331.0019; found 331.0020.

Ethyl 2-(4-fluorophenyl)-2-((trifluoromethyl)sulfonyl)acetate (1c)

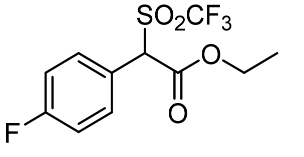

Colourless oil (flash chromatography eluent: hexanes/EtOAc, 98/2 to 90/10), 154.0 mg, 98% yield. ^{1}**H NMR** ($CDCl_3$, 300 MHz): δ 7.57 (d, 2H, J = 8.6 Hz), 7.44 (d, 2H, J = 8.6 Hz), 5.31 (s, 1H), 4.37 (dq, 1H, J = 13.4 Hz, J = 7.2 Hz), 4.30 (dq, 1H, J = 13.4 Hz, J = 7.2 Hz), 1.32 (t, 3H, J = 7.2 Hz). ^{13}**C NMR** ($CDCl_3$, 62.5 MHz): δ 164.4 (d,$^1J_{CF}$ = 252.2 Hz), 162.2, 132.9 (d,$^3J_{CF}$ = 8.8 Hz), 120.3 (d,$^4J_{CF}$ = 3.1 Hz), 119.9 (q,$^1J_{CF}$ = 330.3 Hz), 116.6 (d,$^2J_{CF}$ = 22.1 Hz), 69.9, 64.0, 13.9. ^{19}**F NMR** ($CDCl_3$, 282 MHz): δ -73.5. **HRMS (MALDI)** m/z: $[M+H]^+$ Calcd for $C_{11}H_{11}F_4O_4S$ 315.0314; found 315.0312.

Ethyl 2-(2-fluorophenyl)-2-((trifluoromethyl)sulfonyl)acetate (1d)

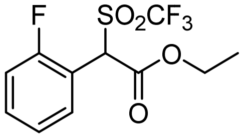

Colourless oil(flash chromatography eluent: hexanes/EtOAc, 98/2 to 90/10), 102.1 mg, 65% yield. ^{1}**H NMR** ($CDCl_3$, 300 MHz): δ 7.92 (t, 1H, J = 7.4 Hz), 7.50 (qd, 1H, J = 7.6 Hz J = 1.6 Hz), 7.29 (d, 1H, J = 7.1 Hz), 7.19 (t, 1H, J = 8.6 Hz), 5.84 (s, 1H), 4.38 (dq, 1H, 1H, J = 14.2 Hz, J = 7.2 Hz), 4.38 (dq, 1H, 1H, J = 14.2 Hz, J = 7.2 Hz), 1.35 (t, 3H, J = 7.2 Hz). ^{13}**C NMR** ($CDCl_3$, 62.5 MHz): δ 161.9, 160.9 (d,$^1J_{CF}$ = 251.1 Hz), 132.9 (d,$^3J_{CF}$ = 8.7 Hz), 131.6, 125.2, 119.8 (q,$^1J_{CF}$ = 330.0 Hz), 116.0 (d,$^2J_{CF}$ = 22.0 Hz), 112.6 (d,$^2J_{CF}$ = 13.6 Hz), 64.0, 62.1, 13.9. ^{19}**F NMR** ($CDCl_3$, 376 MHz): δ -74.5, -116.6. **HRMS (MALDI)** m/z: $[M+H]^+$ Calcd for $C_{11}H_{11}F_4O_4S$ 315.0314; found 315.0311.

Ethyl 2-phenyl-2-((trifluoromethyl)sulfonyl)acetate (1e)

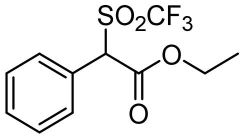

White solid (flash chromatography eluent: hexanes/EtOAc, 98/2 to 90/10), 120.0 mg, 81% yield. **mp** 49.5-50.1°C. ^{1}**H NMR** ($CDCl_3$, 300 MHz): δ 7.62 (dd, 2H, J = 7.6 Hz, J = 1.6 Hz), 7.51-7.43 (m, 3H), 5.31 (s, 1H), 4.36 (dq, 1H, J = 14.3 Hz, J = 7.2 Hz), 4.32 (dq, 1H, J = 14.3 Hz, J = 7.2 Hz), 1.33 (t, 3H, J = 7.2 Hz). ^{13}**C NMR** ($CDCl_3$, 75 MHz): δ 162.4, 130.9, 130.8, 129.4, 124.5, 119.9 (q,$^1J_{CF}$ = 329.9 Hz), 70.5, 63.8, 13.9. ^{19}**F NMR** ($CDCl_3$, 282 MHz): δ -73.6. **HRMS (MALDI)** m/z: $[M+H]^+$ Calcd for $C_{11}H_{12}F_3O_4S$ 297.0408; found 297.0410.

Ethyl 2-(p-tolyl)-2-((trifluoromethyl)sulfonyl)acetate (1f)

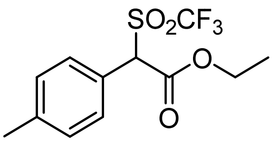

White solid(flash chromatography eluent: hexanes/EtOAc, 98/2 to 90/10), 96.2 mg, 62% yield. **mp** 49.9-50.7 °C. ^{1}**H NMR** ($CDCl_3$, 300 MHz): δ 7.49 (d, 2H, J = 8.2 Hz), 7.27 (d, 2H, J = 8.2 Hz), 5.28 (s, 1H), 4.34 (dq, 1H, J = 14.3 Hz, J = 7.2 Hz), 4.31 (dq, 1H, J = 14.3 Hz, J = 7.2 Hz), 2.39 (s, 3H), 1.32 (t, 3H, J = 7.2 Hz). ^{13}**C NMR** ($CDCl_3$, 75 MHz): δ 162.5, 141.3, 130.7, 130.1, 121.4, 119.9 (q,$^{1}J_{CF}$ = 330.1 Hz), 70.3, 63.7, 21.4, 13.9. ^{19}**F NMR** ($CDCl_3$, 282 MHz): δ -73.6. **HRMS (MALDI)** m/z: $[M+H]^+$ Calcd for $C_{12}H_{14}F_3O_4S$ 311.0565; found 311.0564.

Ethyl 2-(o-tolyl)-2-((trifluoromethyl)sulfonyl)acetate (1g)

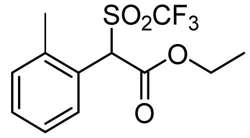

Colourless oil (flash chromatography eluent: hexanes/EtOAc, 98/2 to 90/10), 86.9 mg, 56% yield. ^{1}**H NMR** ($CDCl_3$, 300 MHz): δ 7.85 (dd, 1H, J = 8.0 Hz, J = 1.5 Hz), 7.40-7.28 (m, 3H), 5.66 (s, 1H), 4.34 (dq, 1H, J = 14.3 Hz, J = 7.1 Hz), 4.29 (dq, 1H, J = 14.3 Hz, J = 7.1 Hz), 2.46 (s, 3H), 1.31 (t, 3H, J = 7.1 Hz). ^{13}**C NMR** ($CDCl_3$, 75 MHz): δ 162.8, 138.6, 131.4, 130.8, 130.3, 127.1, 122.9, 120.0 (q,$^{1}J_{CF}$ = 330.1 Hz), 65.6, 63.7, 19.8, 13.9. ^{19}**F NMR** ($CDCl_3$, 376 MHz): δ -74.4. **HRMS (MALDI)** m/z: $[M+H]^+$ Calcd for $C_{12}H_{14}F_3O_4S$ 311.0565; found 311.0566.

Ethyl 2-(3-methoxyphenyl)-2-((trifluoromethyl)sulfonyl)acetate (1h)

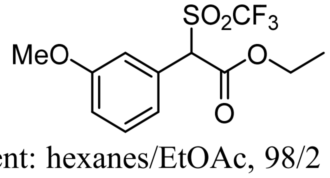

Colourless oil (flash chromatography eluent: hexanes/EtOAc, 98/2 to 85/15), 155.0 mg, 95% yield. ^{1}**H NMR** ($CDCl_3$, 400 MHz): δ 7.36 (t, 1H, J = 7.9 Hz), 7.18 (s, 1H), 7.15 (d, 1H, J = 7.7 Hz), 7.03 (d, 1H, J = 8.0 Hz), 5.28 (s, 1H), 4.35 (dq, 1H, J = 14.0 Hz, J = 7.1 Hz), 4.31 (dq, 1H, J = 14.0 Hz, J = 7.1 Hz), 3.82 (s, 3H), 1.32 (t, 3H, J = 7.1 Hz). ^{13}**C NMR** ($CDCl_3$, 100 MHz): δ 162.3, 160.2, 130.3, 125.6, 123.1, 119.9 (q,$^{1}J_{CF}$ = 329.8 Hz), 116.7, 116.1, 70.3, 63.8, 55.5, 13.9. ^{19}**F NMR** ($CDCl_3$, 376 MHz): δ -73.7. **HRMS (MALDI)** m/z: $[M+H]^+$ Calcd for $C_{12}H_{14}F_3O_5S$ 327.0514; found 327.0517.

Ethyl 2-(4-(trifluoromethyl)phenyl)-2-((trifluoromethyl)sulfonyl)acetate (1i)

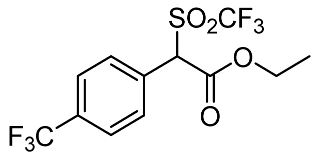

White solid (flash chromatography eluent: hexanes/EtOAc, 98/2 to 90/10), 109.3 mg, 60% yield. **mp** 46.2-47.4°C. **^{1}H NMR** ($CDCl_3$, 300 MHz): δ 7.78 (d, 2H, J = 8.6 Hz), 7.73 (d, 2H, J = 8.6 Hz), 5.36 (s, 1H), 4.38 (dq, 1H, J = 14.3 Hz, J = 7.2 Hz), 4.34 (dq, 1H, J = 14.3 Hz, J = 7.2 Hz), 1.34 (t, 3H, J = 7.2 Hz). **^{13}C NMR** ($CDCl_3$, 75 MHz): δ 161.8, 133.1 (q,$^2J_{CF}$ = 33.0 Hz), 131.3, 128.5, 126.3 (q,$^3J_{CF}$ = 3.5 Hz), 123.6 (q,$^1J_{CF}$ = 272.8 Hz), 119.8 (q,$^1J_{CF}$ = 329.6 Hz), 69.8, 64.2, 13.9. **^{19}F NMR** ($CDCl_3$, 376 MHz): δ -63.1, -73.5. **HRMS (MALDI)** m/z: $[M+H]^+$ Calcd for $C_{12}H_{11}F_6O_4S$ 365.0282; found 365.0284.

Ethyl 2-(naphthalen-2-yl)-2-((trifluoromethyl)sulfonyl)acetate (1j)

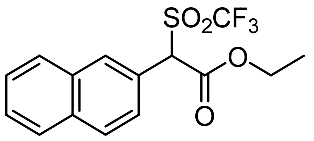

White solid (flash chromatography eluent: hexanes/EtOAc, 98/2 to 90/10), 154.1 mg, 89% yield. **mp** 96.0-98.7°C. **^{1}H NMR** ($CDCl_3$, 400 MHz): δ 8.11 (d, 1H, J = 1.5 Hz), 7.95-7.87 (m, 3H), 7.68 (dd, 1H, J = 8.6 Hz, J = 1.9 Hz), 7.61-7.54 (m, 2H), 5.48 (s, 1H), 4.38 (dq, 1H, J = 14.3 Hz, J = 7.2 Hz), 4.34 (dq, 1H, J = 14.3 Hz, J = 7.2 Hz), 1.34 (t, 3H, J = 7.2 Hz). **^{13}C NMR** ($CDCl_3$, 100 MHz): δ 162.5, 134.1, 133.1, 131.4, 129.3, 128.6, 127.9 (2C), 127.1, 126.7, 121.7, 120.0 (q,$^1J_{CF}$ = 329.5 Hz), 70.6, 63.8, 13.9. **^{19}F NMR** ($CDCl_3$, 376 MHz): δ -73.5. **HRMS (MALDI)** m/z: $[M+H]^+$ Calcd for $C_{15}H_{14}F_3O_4S$ 347.0565; found 347.0569.

Ethyl 2-(3,4-dichlorophenyl)-2-((trifluoromethyl)sulfonyl)acetate (1k)

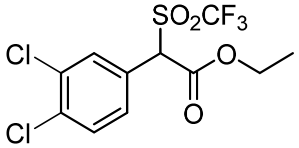

Colourless oil (flash chromatography eluent: hexanes/EtOAc, 98/2 to 90/10), 85.8 mg, 47% yield. **^{1}H NMR** ($CDCl_3$, 400 MHz): δ 7.73 (d, 1H, J = 2.1 Hz), 7.55 (d, 1H, J = 8.4 Hz), 7.49 (dd, 1H, J = 8.4 Hz, J = 2.1 Hz), 5.25 (s, 1H), 4.37 (dq, 1H, J = 14.3 Hz, J = 7.2 Hz), 4.33 (dq, 1H, J = 14.4 Hz, J = 7.2 Hz), 1.34 (t, 3H, J = 7.2 Hz). **^{13}C NMR** ($CDCl_3$, 100 MHz): δ 161.7, 135.9, 133.8, 132.6, 131.3, 130.0, 124.4, 119.8 (q,$^1J_{CF}$ = 330.1 Hz), 69.1, 64.3, 13.9. **^{19}F NMR** ($CDCl_3$, 376 MHz): δ -73.4. **HRMS (MALDI)** m/z: $[M+H]^+$ Calcd for $C_{11}H_{10}Cl_2F_3O_4S$ 366.9629; found 366.9624.

Ethyl 2-(3,5-dimethylphenyl)-2-((trifluoromethyl)sulfonyl)acetate (1l)

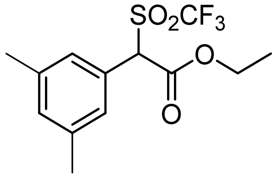

Colourless oil (flash chromatography eluent: hexanes/EtOAc, 98/2 to 90/10), 111.0 mg, 72% yield. ^{1}H NMR ($CDCl_3$, 300 MHz): δ 7.21 (s, 2H), 7.12 (s, 1H), 5.23 (s, 1H), 4.35 (dq, 1H, J = 14.2 Hz, J = 7.2 Hz), 4.30 (dq, 1H, J = 14.2 Hz, J = 7.2 Hz), 2.35 (s, 6H), 1.33 (t, 3H, J = 7.2 Hz). ^{13}C NMR ($CDCl_3$, 100 MHz): δ 162.6, 139.2, 132.7, 128.4, 124.0, 119.9 (q,$^{1}J_{CF}$ = 329.9 Hz), 70.3, 63.6, 21.3, 13.9. ^{19}F NMR ($CDCl_3$, 376 MHz): δ -73.6. **HRMS (MALDI)** m/z: $[M+H]^+$ Calcd for $C_{13}H_{16}F_3O_4S$ 325.0721; found 325.0727.

Ethyl 2-(4-cyanophenyl)-2-((trifluoromethyl)sulfonyl)acetate (1m)

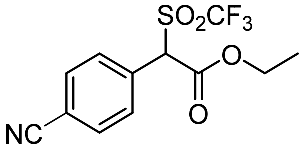

Colourless oil (flash chromatography eluent: hexanes/EtOAc, 98/2 to 85/15), 117.3 mg, 73% yield (75% purity). **^{1}H NMR** ($CDCl_3$, 300 MHz): δ 7.76 (m, 4H), 5.37 (s, 1H), 4.37 (dq, 1H, J = 14.3 Hz, J = 7.1 Hz), 4.33 (dq, 1H, J = 14.3 Hz, J = 7.1 Hz), 1.33 (t, 3H, J = 7.1 Hz). **^{13}C NMR** ($CDCl_3$, 150 MHz): δ 161.5, 132.9, 131.7, 129.5, 119.8 (q,$^{1}J_{CF}$ = 329.5 Hz), 117.8, 115.1, 69.8, 64.4, 13.9. **^{19}F NMR** ($CDCl_3$, 376 MHz): δ -73.3. **HRMS (MALDI)** m/z: $[M+H]^+$ Calcd for $C_{12}H_{11}F_3NO_4S$ 322.0361; found 322.0365.

Ethyl 2-(3-fluorophenyl)-2-((trifluoromethyl)sulfonyl)acetate (1n)

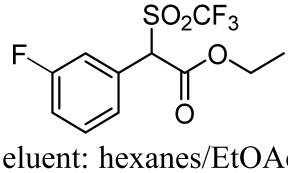

Colourless oil (flash chromatography eluent: hexanes/EtOAc, 98/2 to 90/10), 138.2 mg, 86% yield. **^{1}H NMR** ($CDCl_3$, 300 MHz): δ 7.48-7.35 (m, 3H), 7.21 (tt, 1H, J = 8.3 Hz, J = 1.3 Hz,), 5.30 (s, 1H), 4.36 (dqd, 1H, J = 14.4 Hz, J = 7.2 Hz, J = 1.0 Hz), 4.33 (dqd, 1H, J = 14.4 Hz, J = 7.2 Hz, J = 1.0 Hz), 1.33 (dt, 3H, J = 7.2 Hz, J = 1.0 Hz). **^{13}C NMR** ($CDCl_3$, 75 MHz): δ 162.8 (d,$^{1}J_{CF}$ = 247.5 Hz), 161.9, 130.9 (d,$^{1}J_{CF}$ = 7.5 Hz), 126.8, 126.5 (d,$^{1}J_{CF}$ = 8.3 Hz), 119.9 (q,$^{1}J_{CF}$ = 330.1 Hz), 118.1, 118.1 (d,$^{1}J_{CF}$ = 44.6 Hz), 69.8, 64.1, 13.9. **^{19}F NMR** ($CDCl_3$, 376 MHz): δ -73.5, -110.5. **HRMS (MALDI)** m/z: $[M+H]^+$ Calcd for $C_{11}H_{11}F_4O_4S$ 315.0314; found 315.0313.

Ethyl 2-(3-chlorophenyl)-2-((trifluoromethyl)sulfonyl)acetate (1o)

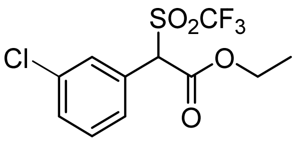

Colourless oil (flash chromatography eluent: hexanes/EtOAc, 98/2 to 90/10), 110.8 mg, 67% yield. **^{1}H NMR** ($CDCl_3$, 400 MHz): δ 7.63 (t, 1H, J = 1.7 Hz), 7.50 (td, 2H, J = 7.9 Hz, J = 1.7 Hz), 7.40 (t, 1H, J = 7.9 Hz), 5.26 (s, 1H), 4.36 (dq, 1H, J = 14.3 Hz, J = 7.2 Hz), 4.32 (dq, 1H, J = 14.3 Hz, J = 7.2 Hz), 1.34 (t, 3H, J = 7.2 Hz). **^{13}C NMR** ($CDCl_3$, 100 MHz): δ 161.9, 135.4, 131.2, 130.8, 130.5, 129.0, 126.3, 119.9 (q, $^{1}J_{CF}$ = 330.1 Hz), 69.7, 64.1, 13.9. **^{19}F NMR** ($CDCl_3$, 376 MHz): δ -73.3. **HRMS (MALDI)** m/z: $[M+H]^+$ Calcd for $C_{11}H_{11}ClF_3O_4S$ 331.0019; found 331.0022.

Ethyl 2-([1,1'-biphenyl]-4-yl)-2-((trifluoromethyl)sulfonyl)acetate (1p)

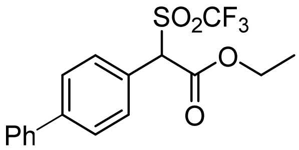

White solid (flash chromatography eluent: hexanes/EtOAc, 98/2 to 90/10), 160.1 mg, 86% yield. **mp** 125.9-127.6°C. **^{1}H NMR** ($CDCl_3$, 300 MHz): δ 7.70 (m, 4H), 7.61 (d, 2H, J = 7.2 Hz), 7.48 (t, 2H, J = 7.3 Hz), 7.40 (t, 1H, J = 7.1 Hz**),** 5.39 (s, 1H), 4.40 (dq, 1H, J = 14.2 Hz, J = 7.1 Hz), 4.33 (dq, 1H, J = 14.1 Hz, J = 7.1 Hz), 1.36 (t, 3H, J = 7.1 Hz). **^{13}C NMR** ($CDCl_3$, 75 MHz): δ 162.4, 143.8, 139.8, 131.2, 129.0, 128.2, 128.0, 127.3, 123.1, 119.9 (q, $^{1}J_{CF}$ = 331.0 Hz), 70.2, 63.8, 13.9. **^{19}F NMR** ($CDCl_3$, 376 MHz): δ -73.5. **HRMS (MALDI)** m/z: $[M+H]^+$ Calcd for $C_{17}H_{16}F_3O_4S$ 373.0321; found 373.0329.

Synthesis of acrylpyrazoles (2e-f)

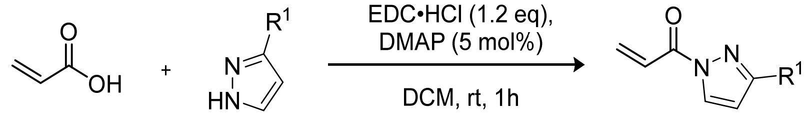

2e:R^{1}= Ph,
2f:R^{1}= H

In an oven-dried 250 mL round bottom flask, acrylic acid (14.6 mmol, 1 mL), the proper pyrazole (12.2 mmol) and DCM (61 mL) were introduced. To this solution EDC*HCl (14.6 mmol, 3.01 g) and DMAP (0.61 mmol, 75.5 mg) were added, and the reaction mixture was stirred at room temperature for 1 h. After full conversion of starting material, as monitored by TLC, the solvent was evaporated under reduced pressure and the residue was purified by column chromatography (flash silica gel, eluted by a mixture of n-hexane/ EtOAc 95:5) to afford compounds **2e.**

The compound **2f** was purified by column chromatography (flash silica gel, eluted by a mixture of n-pentane/Et_2O 95:5). ATTENTION to the high volatility of compound **2f**: carefully evaporate the

solvent (120 mbar, 25°C) to concentrate the crude mixture before purification. **2f** is a colorless volatile oil. The spectral data of the compound matched with the literature.[3]

1-(3-phenyl-1H-pyrazol-1-yl)prop-2-en-1-one (2e)

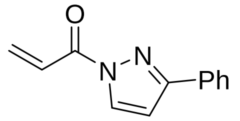

White wax, 1.81 g, 75% yield. **^{1}H NMR** ($CDCl_3$, 600 MHz): δ 8.37 (d, 1H, J = 2.9 Hz), 7.79 (dd, 2H, J = 7.1 Hz, J = 1.4 Hz), 7.71 (dd, 1H, J = 17.3 Hz, J = 10.5 Hz), 7.46 (t, 2H, J = 7.2 Hz), 7.41 (t, 1H, J = 7.3 Hz), 6.81 (d, 1H, J = 2.8 Hz), 6.78 (dd, 1H, J = 17.3 Hz, J = 1.4 Hz, J = 6.8 Hz), 6.10 (dd, 1H, J = 10.4 Hz, J = 1.4 Hz). **^{13}C NMR** ($CDCl_3$, 150 MHz): δ 163.2, 155.6, 133.3, 131.9, 130.1, 129.3, 128.9, 126.7, 126.4, 108.0. **HRMS (ESI)** m/z $[M+Na]^+$ Calcd for $C_{12}H_{10}N_2ONa$ 221.0694; found 221.0688.

General procedure for racemic Michael reaction of vinyl ketones

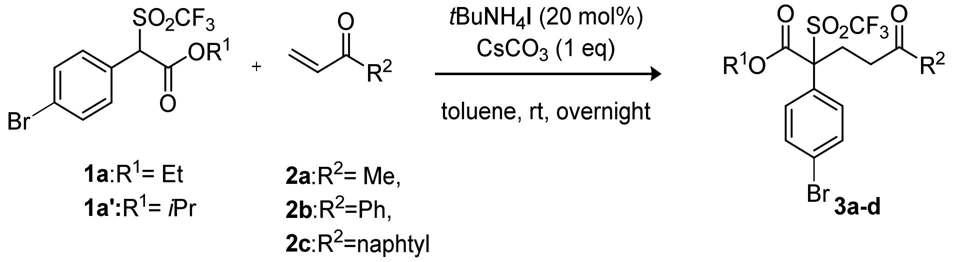

In an oven-dried vial, α-trifluoromethylsulfonyl esters **1a-1a'** (0.1 mmol), vinyl ketone **2b-c** (0.12 mmol), or methyl vinyl ketone **2a** (0.25 mmol, 21 μL), and anhydrous toluene (0.5 mL) were introduced. To this solution *t*BuNH$_4$I (0.02 mmol, 7.39 mg) and $CsCO_3$ (0.1 mmol, 32.6 mg) were added under nitrogen atmosphere and the reaction mixture was stirred at room temperature for 19 h. After completion of the reaction, the reaction was quenched with HCl (1N) and the mixture was extracted with diethyl ether (x3). The organic layers were dried over Na_2SO_4, and the solvent was evaporated. The crude mixture was purified by flash chromatography (eluent: hexane/ethyl acetate 100/0 to 90/10) to afford products **3a-d** in 25-56% yield.

General procedure for enantioselective Michael reaction of vinyl ketones

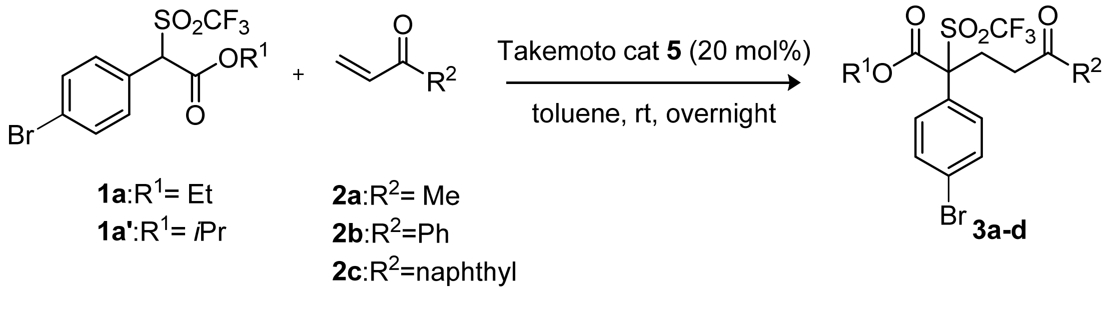

In an oven-dried vial, *α*-trifluoromethylsulfonyl esters **1a-1a'** (0.1 mmol), vinyl ketone **2b-c** (0.12 mmol), or methyl vinyl ketone **2a** (0.25 mmol, 21 μL), and anhydrous toluene (0.5 mL) were introduced. To this solution (*R*,*R*)-Takemoto catalyst **5** (0.02 mmol, 8.26 mg) was added under nitrogen atmosphere and the reaction mixture was stirred at room temperature for 17 h, as reported in the Table 1. After completion of the reaction the solvent was evaporated and the crude mixture was purified by flash chromatography (eluent: hexane/ethyl acetate 100/0 to 90/10) to afford products **3a-d** in 75-85% yield.

(*R*)-Ethyl 2-(4-bromophenyl)-5-oxo-2-((trifluoromethyl)sulfonyl)hexanoate (3a)

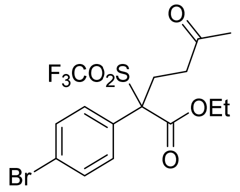

Colourless oil (flash chromatography eluent: hexanes/EtOAc, 98/2 to 90/10), 33.4 mg, 75% yield (55% ee). $[\alpha]_D^{23}$ = +10.61 (*c* 0.89, $CHCl_3$). **^{1}H NMR** ($CDCl_3$, 300 MHz): *δ* 7.58 (d, 2H, *J* = 8.9 Hz), 7.34 (d, 2H, *J* = 8.9 Hz), 4.44 (dq, 2H, *J* = 7.2 Hz, *J* = 1.6 Hz), 2.92 (dd, 2H, *J* = 8.0 Hz, *J* = 6.8 Hz), 2.79 (ddd, 1H, *J* = 18.0 Hz, *J* = 8.0 Hz, *J* = 6.8 Hz), 2.41 (ddd, 1H, *J* = 18.0 Hz, *J* = 8.0 Hz, *J* = 6.8 Hz), 2.11 (s, 3H), 1.39 (t, 3H, *J* = 7.2 Hz). **^{13}C NMR** ($CDCl_3$, 75 MHz): *δ* 205.3, 165.7, 132.3, 131.2, 127.8, 125.3, 120.7 (q, $^1J_{CF}$ = 334.5 Hz), 82.5, 64.1, 38.6, 26.5, 13.9. **^{19}F NMR** ($CDCl_3$, 376 MHz): *δ* -67.2. **HRMS (ESI)** m/z: $[M+Na]^+$ Calcd for $C_{15}H_{16}BrF_3O_5SNa$ 466.9752; found 466.9798. **HPLC** (AD-H, 2-propanol/ n-hexane = 10/90, flow rate 1.0 mL/min, l = 220 nm) t_R = 12.5 min, (major) t_R = 10.6 min (minor).

(*R*)-Ethyl 2-(4-bromophenyl)-5-oxo-5-phenyl-2-((trifluoromethyl)sulfonyl)pentanoate (3b)

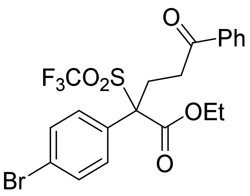

Colourless oil (flash chromatography eluent: hexanes/EtOAc, 98/2 to 90/10), 40.1 mg, 79% yield (67% ee). $[\alpha]_D^{16}$ = +1.84 (*c* 0.58, $CHCl_3$). **^{1}H NMR** ($CDCl_3$, 300 MHz): *δ* 7.88 (dd, 2H, *J* = 8.3 Hz, *J* = 1.2 Hz), 7.60-7.54 (m, 3H), 7.47-7.38 (m, 4H), 4.45 (q, 2H, *J* = 7.2 Hz), 3.37 (ddd, 1H, *J* = 17.3 Hz, *J* = 9.2 Hz, *J* = 6.2 Hz), 3.11 (m, 2H), 2.93 (ddd, 1H, *J* = 17.4 Hz, *J* = 8.9 Hz, *J* = 5.3 Hz), 1.37 (t, 3H, *J* = 7.2 Hz). **^{13}C NMR** ($CDCl_3$, 75 MHz): *δ* 197.1, 165.7, 136.3, 133.7, 132.4, 131.3, 128.9, 128.1, 127.9, 125.4, 120.8 (q, $^1J_{CF}$ = 333.3 Hz), 82.8, 64.1, 33.9, 27.1, 13.9. **^{19}F NMR** ($CDCl_3$, 282 MHz): *δ* -67.1. **HRMS (ESI)** m/z: $[M+Na]^+$ Calcd for $C_{20}H_{18}BrF_3O_5SNa$ 528.9903; found

528.9898. **HPLC** (AD-H, 2-propanol/ n-hexane = 10/90, flow rate 1.0 mL/min, l = 220 nm), t_R = 17.3 min (major), t_R = 14.6 min (minor).

(*R*)-Isopropyl 2-(4-bromophenyl)-5-oxo-5-phenyl-2-((trifluoromethyl)sulfonyl)pentanoate (3c)

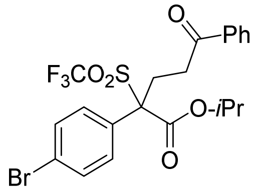

Colourless oil (flash chromatography eluent: hexanes/EtOAc, 98/2 to 90/10), 39.6 mg, 76% yield (63% ee). $[\alpha]_D^{17}$ = +1.59 (*c* 0.53, $CHCl_3$). **^{1}H NMR** ($CDCl_3$, 400 MHz): δ 7.89 (dd, 2H, *J* = 8.4 Hz, *J* = 1.2 Hz), 7.58 (dd, 2H, *J* = 8.7 Hz, *J* = 1.6 Hz), 7.57 (t, 1H, *J* = 7.5 Hz), 7.45 (t, 2H, *J* = 7.5 Hz), 7.40 (dd, 2H, *J* = 8.7 Hz, *J* = 1.6 Hz), 5.32 (hept, 1H, *J* = 6.1 Hz), 3.38 (ddd, 1H, *J* = 17.5 Hz, *J* = 9.5 Hz, *J* = 5.3 Hz), 3.11 (m, 2H), 2.93 (ddd, 1H, *J* = 17.5 Hz, *J* = 9.5 Hz, *J* = 5.7 Hz), 1.38 (d, 3H, *J* = 6.0 Hz), 1. (d, 3H, *J* = 6.0 Hz). **^{13}C NMR** ($CDCl_3$, 100 MHz): δ 197.1, 165.2, 136.2, 133.7, 132.3, 131.2, 128.9, 128.1, 128.0, 125.3, 120.7 (q, $^1J_{CF}$ = 336.3 Hz), 82.6, 72.9, 33.9, 27.1, 21.6, 21.4. **^{19}F NMR** ($CDCl_3$, 376 MHz): δ -66.9. **HRMS (ESI)** m/z: $[M+Na]^+$ Calcd for $C_{21}H_{20}BrF_3O_5SNa$ 543.0059; found 543.0043. **HPLC** (AD 2-propanol/ n-hexane = 10/90, flow rate 1.0 mL/min, l = 220 nm), t_R = 13.7 min (major), t_R = 9.1 min (minor).

Ethyl 2-(4-bromophenyl)-5-(naphthalen-1-yl)-5-oxo-2-((trifluoromethyl)sulfonyl)pentanoate (3d)

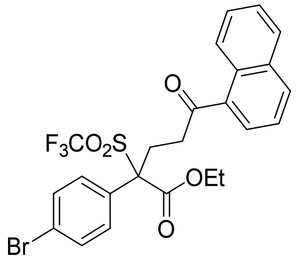

Colourless oil (flash chromatography eluent: hexanes/EtOAc, 98/2 to 90/10), 49.1 mg, 85% yield (44% ee). $[\alpha]_D^{14}$ = +0.67 (*c* 0.29, $CHCl_3$). **^{1}H NMR** ($CDCl_3$, 400 MHz): δ 8.59 (d, 1H, *J* = 8.5 Hz), 7.99 (d, 1H, *J* = 8.3 Hz), 7.87 (d, 1H, *J* = 8.3 Hz), 7.76 (d, 1H, *J* = 7.2 Hz), 7.63-7.53 (m, 4H), 7.48-7.42 (m, 3H), 4.46 (q, 2H, *J* = 7.2 Hz), 3.45 (ddd, 1H, *J* = 17.6 Hz, *J* = 8.2 Hz, *J* = 6.7 Hz), 3.20 (m, 2H), 3.01 (ddd, 1H, *J* = 17.6 Hz, *J* = 8.2 Hz, *J* = 5.9 Hz), 1.38 (t, 3H, *J* = 7.2 Hz). **^{13}C NMR** ($CDCl_3$, 100 MHz): δ 200.9, 165.7, 134.9, 134.1, 133.4, 132.4, 131.3, 130.2, 128.7, 128.4, 128.0, 127.8, 126.7, 125.7, 125.4, 124.4, 120.8 (q, $^1J_{CF}$ = 334.1 Hz), 82.8, 64.1, 37.1, 27.3, 13.9. **^{19}F NMR** ($CDCl_3$, 376 MHz): δ -67.1. **HRMS (ESI)** m/z: $[M+Na]^+$ Calcd for $C_{24}H_{20}BrF_3O_5SNa$ 579.0065;

found 579.0047. **HPLC** (AD, 2-propanol/ n-hexane = 10/90, flow rate 1.0 mL/min, l = 254-220 nm) t_R = 16.4 min (major), t_R = 26.4 min (minor).

General procedure for racemic Michael reaction of α,β-unsaturated pyrazoleamides

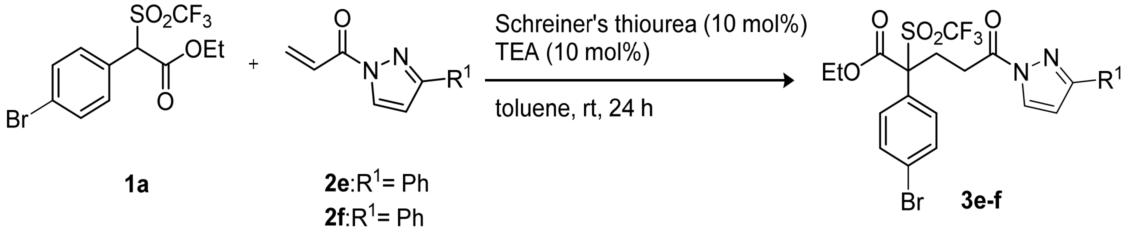

In an oven-dried vial α-trifluoromethylsulfonyl esters **1a** (0.1 mmol), the proper acrylpyrazole **2e-f** (0.15 mmol) and anhydrous toluene (0.5 mL) were introduced. To this solution Schreiner′s thiourea catalyst (0.01 mmol, 5.00 mg) and triethylamine (0.01 mmol, 1.5 μL) were added under nitrogen atmosphere and the reaction mixture was stirred at room temperature for 24 h and monitored by TLC. After completion of the reaction the solvent was evaporated, and the crude mixture was purified by flash chromatography (eluent: hexane/ethyl acetate 100/0 to 90/10) to afford products **3e-f** in 70-93% yield.

General procedure for enantioselective Michael reaction of α,β-unsaturated pyrazoleamides

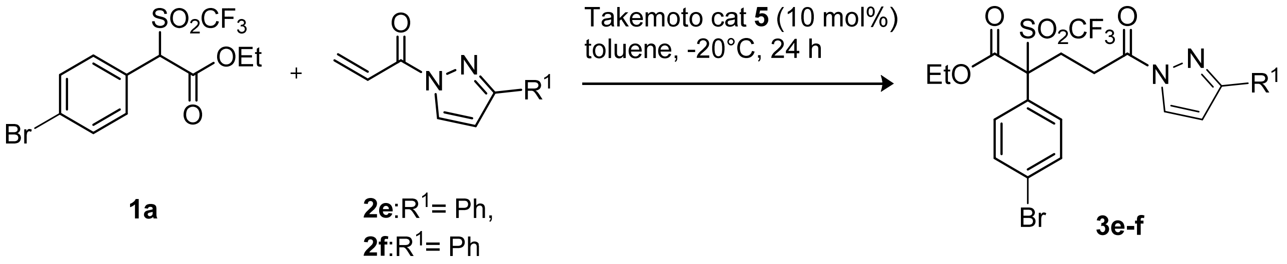

In an oven-dried vial α-trifluoromethylsulfonyl esters **1a** (0.1 mmol), the proper acrylpyrazole **2e-f** (0.15 mmol) and anhydrous toluene (0.5 mL) were introduced. The reaction mixture was cooled at -20°C and Takemoto catalyst **5** (0.01 mmol, 4.13 mg) was added. The reaction mixture was stirred at -20°C for 24 h and monitored by TLC, as reported in Table 1. After completion of the reaction the solvent was evaporated, and the crude mixture was purified by flash chromatography (eluent: hexane/ethyl acetate 100/0 to 90/10) to afford products **3e-f** in 93-96% yield.

(*R*)-Ethyl 2-(4-bromophenyl)-5-oxo-5-(3-phenyl-1H-pyrazol-1-yl)-2 ((trifluoromethyl)sulfonyl)pentanoate (3e)

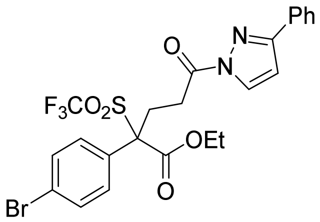

Colourless oil (flash chromatography eluent: hexanes/EtOAc, 98/2 to 90/10), 35.0 mg, 61% yield (86% ee). **[α]**$_D^{14}$ = -25.1 (*c* 0.47, $CHCl_3$). **^{1}H NMR** ($CDCl_3$, 300 MHz): *δ* 8.25 (d, 1H, *J* = 2.9 Hz), 7.82 (dd, 2H, *J* = 7.8 Hz, *J* = 1.5 Hz), 7.60 (d, 2H, *J* = 8.7 Hz), 7.49-7.38 (m, 5H), 6.78 (d, 1H, *J* = 2.9 Hz), 4.49 (dq, 2H, *J* = 7.1 Hz, *J* = 1.5 Hz), 3.53-3.42 (m, 1H), 3.28-3.16 (m, 3H), 1.40 (t, 3H, *J* = 7.1 Hz). **^{13}C NMR** ($CDCl_3$, 75 MHz): *δ* 170.1, 165.4, 156.0, 132.4, 131.6, 131.5, 129.7, 129.5, 128.9, 127.2, 126.5, 125.5, 120.8 (q,$^1J_{CF}$ = 333.5 Hz), 108.0, 82.7, 64.2, 29.9, 27.4, 13.9. **^{19}F NMR** ($CDCl_3$, 282 MHz): *δ* -67.0. **HRMS (ESI)** m/z: $[M+Na]^+$ Calcd for $C_{23}H_{20}BrF_3N_2O_5SNa$ 595.0126; found 595.0118. **HPLC** (AD, 2-propanol/ n-hexane = 10/90, flow rate 1.0 mL/min, l = 254-220 nm) t_R = 12.7 min (major), t_R = 15.1 min (minor).

(*R*)-Ethyl 2-(4-bromophenyl)-5-oxo-5-(1H-pyrazol-1-yl)-2-((trifluoromethyl)sulfonyl)pentanoate (3f)

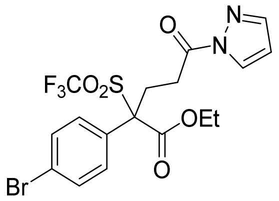

Colourless oil (flash chromatography eluent: hexanes/EtOAc, 98/2 to 90/10), 47.0 mg, 95% yield (94% ee). [α]$_D^{21}$ = +6.62 (*c* 0.64, $CHCl_3$). **^{1}H NMR** ($CDCl_3$, 300 MHz): *δ* 8.22 (s, 1H), 7.67 (s, 1H), 7.59 (d, 2H, *J* = 8.5 Hz), 7.43 (d, 2H, *J* = 8.5 Hz), 6.44 (s, 1H), 4.46 (q, 2H, *J* = 7.2 Hz), 3.54-3.39 (m, 1H), 3.17-3.05 (m, 3H), 1.38 (t, 3H, *J* = 7.2 Hz). **^{13}C NMR** ($CDCl_3$, 75 MHz): *δ* 170.2, 165.4, 144.5, 132.4, 131.3, 128.5, 127.2, 125.5, 120.8 (q,$^1J_{CF}$ = 334.1 Hz), 110.1, 82.6, 64.2, 29.9, 27.2, 13.8. **^{19}F NMR** ($CDCl_3$, 282 MHz): *δ* -67.0. **HRMS (ESI)** m/z: $[M+Na]^+$ Calcd for $C_{17}H_{16}BrF_3N_2O_5SNa$ 518.9813; found 518.9801. **HPLC** (AD, 2-propanol/ n-hexane = 10/90, flow rate 1.0 mL/min, l = 254-220 nm) t_R = 12.6 min (major), t_R = 18.3 min (minor).

General procedure for racemic Michael reaction and one-pot derivatization

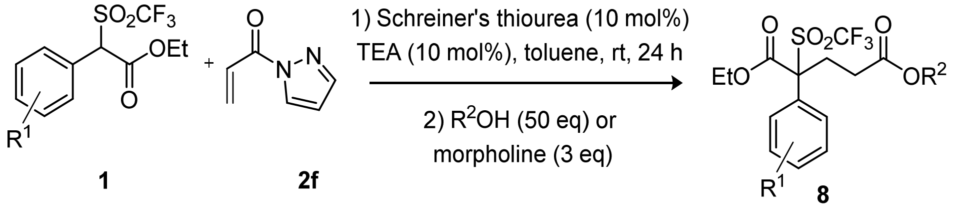

In an oven-dried vial, α-trifluoromethylsulfonyl esters **1a-o** (0.1 mmol), the acrylpyrazole **2f** (0.15 mmol, 18.3 mg) and anhydrous toluene (0.5 mL) were introduced. To this solution Schreiner's thiourea catalyst (0.01 mmol, 5.00 mg) and triethylamine (0.01 mmol, 1.5 μL) were added under nitrogen atmosphere and the reaction mixture was stirred at room temperature for 24 h and monitored by TLC. After completion of the first step, anhydrous alcohol (5 mmol), or morpholine in the case of compound **8p** (0.3 mmol, 26 μL), was added and the mixture was stirred for 13-60 hours at room temperature or 50°C, by using an oil bath, according to Scheme 2, and monitored by TLC. After completion of the reaction the solvent was evaporated, and the crude mixture was purified by flash chromatography (eluent: hexane/ethyl acetate 100/0 to 90/10) to afford products **8a-p** in 70-93% yield.

General procedure for enantioselective Michael reaction and one-pot derivatization

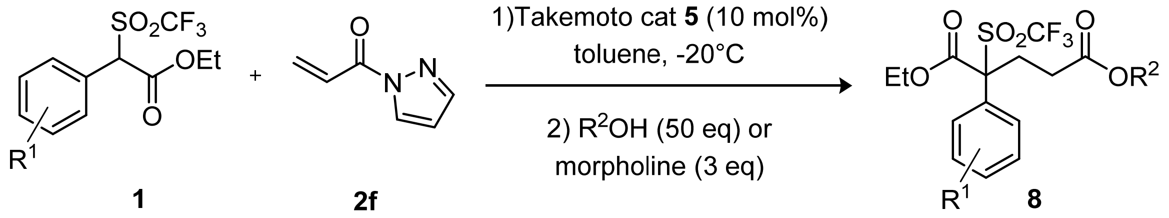

In an oven-dried vial α-trifluoromethylsulfonyl esters **1a-o** (0.1 mmol), acrylpyrazole **2f** (0.15 mmol, 18.3 mg) and anhydrous toluene (0.5 mL) were introduced. The reaction mixture was cooled at -20°C and Takemoto catalyst **5** was added (0.01 mmol, 4.13 mg) at the same temperature. The reaction mixture was stirred at -20°C for the time reported in the Scheme 2 and monitored by TLC. After completion of the first step, anhydrous alcohol (5 mmol), or morpholine in the case of compound **8p** (0.3 mmol, 26 μL), was added and the mixture was stirred for the indicated time and temperature reported in Scheme 2 and monitored by TLC. After completion of the reaction the solvent was evaporated and the crude mixture was purified by flash chromatography (eluent: hexane/ethyl acetate 100/0 to 90/10) to afford products **8a-p** in 40-98% yield and 82-96% ee.

Scale-up of model one-pot reaction on compound 8a

In an oven-dried vial, *α*-trifluoromethylsulfonyl esters **1a** (1.0 mmol, 375.2 mg), acrylpyrazole **2f** (1.5 mmol, 183.2 mg) and anhydrous toluene (5 mL) were introduced. The reaction mixture was cooled at -20°C and Takemoto catalyst **5** was added (0.1 mmol, 41.4 mg) at the same temperature. The reaction mixture was stirred at -20°C for the time reported in the Scheme 2 and monitored by TLC. After completion of the first step, anhydrous methanol (50 mmol, 2 mL) was added and the mixture was stirred for the indicated time and temperature reported in Scheme 2 and monitored by TLC. After completion of the reaction the solvent was evaporated and the crude mixture was purified by flash chromatography (eluent: hexane/ethyl acetate 100/0 to 90/10) to afford product **8a** in 85% yield and 90% ee.

(*R*)-1-Ethyl 5-methyl 2-(4-bromophenyl)-2-((trifluoromethyl)sulfonyl)pentanedioate (8a)

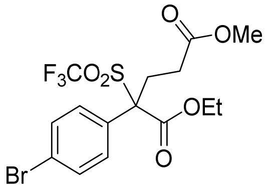

Colourless oil (flash chromatography eluent: hexanes/EtOAc, 98/2 to 90/10), 45.2 mg, 98% yield (91% ee). $[\alpha]_D^{14}$ = +16.85 (*c* 0.47, $CHCl_3$). **^{1}H NMR** ($CDCl_3$, 250 MHz): *δ* 7.58 (d, 2H, *J* = 8.9 Hz), 7.34 (d, 2H, *J* = 8.9 Hz), 4.45 (q, 2H, *J* = 7.2 Hz), 3.64 (s, 3H), 3.01-2.94 (m, 2H), 2.60 (ddd, 1H, *J* = 16.7 Hz, *J* = 9.6 Hz, *J* = 6.6 Hz), 2.28 (ddd, 1H, *J* = 16.7 Hz, *J* = 9.6 Hz, *J* = 6.6 Hz), 1.38 (t, 3H, *J* = 7.2 Hz). **^{13}C NMR** ($CDCl_3$, 75 MHz): *δ* 172.0, 165.4, 132.3, 131.1, 127.4, 125.4, 120.7 (q, $^1J_{CF}$ = 334.1 Hz), 82.4, 64.1, 52.2, 29.5, 27.9, 13.9. **^{19}F NMR** ($CDCl_3$, 376 MHz): *δ* -67.1. **HRMS (ESI)** m/z: $[M+Na]^+$ Calcd for $C_{15}H_{16}BrF_3O_6SNa$ 482.9695, found 482.9697. **HPLC** (AD, 2-propanol/ n-hexane = 10/90, flow rate 1.0 mL/min, l = 254-220 nm) t_R = 10.3 min (major), t_R = 8.6 min (minor).

(*R*)-1-Ethyl 5-methyl 2-(4-chlorophenyl)-2-((trifluoromethyl)sulfonyl)pentanedioate (8b)

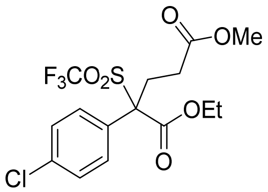

Colourless oil (flash chromatography eluent: hexanes/EtOAc, 98/2 to 90/10), 42.0 mg, 91% yield (91% ee). $[\alpha]_D^{15}$ = +25.02 (*c* 0.62, $CHCl_3$). **^{1}H NMR** ($CDCl_3$, 400 MHz): *δ* 7.43 (m, 4H), 4.45 (dq, 2H, *J* = 7.1 Hz, *J* = 1.9 Hz), 3.68 (s, 3H), 3.01-2.95 (m, 2H), 2.61 (ddd, 1H, *J* = 16.8 Hz, *J* = 10.3 Hz, *J* = 6.6 Hz), 2.29 (ddd, 1H, *J* = 16.8 Hz, *J* = 10.1 Hz, *J* = 5.7 Hz), 1.39 (t, 3H, *J* = 7.1 Hz). **^{13}C NMR** ($CDCl_3$, 100 MHz): *δ* 172.0, 165.4, 137.1, 131.1, 129.4, 126.9, 120.8 (q, $^1J_{CF}$ = 333.1 Hz),

82.3, 64.1, 52.2, 29.6, 28.0, 13.9. **^{19}F NMR** ($CDCl_3$, 376 MHz): δ -67.1. **HRMS (ESI)** m/z: $[M+Na]^+$ Calcd for $C_{15}H_{16}ClF_3O_6SNa$ 439.0200; found 439.0191. **HPLC** (AD, 2-propanol/ n-hexane = 10/90, flow rate 1.0 mL/min, l = 254-220 nm) t_R = 9.5 min (major), t_R = 7.9 min (minor).

(*R*)-1-Ethyl 5-methyl 2-(4-fluorophenyl)-2-((trifluoromethyl)sulfonyl)pentanedioate (8c)

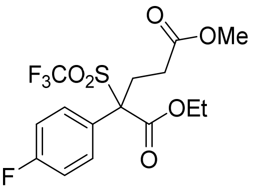

Colourless oil (flash chromatography eluent: hexanes/EtOAc, 98/2 to 90/10), 36.4 mg, 91% yield (82% ee). $[\alpha]_D^{15}$ = +17.1 (*c* 0.39, $CHCl_3$). **^{1}H NMR** ($CDCl_3$, 600 MHz): δ 7.50 (m, 2H), 7.14 (t, 2H, J = 8.5 Hz), 4.45 (q, 2H, J = 7.2 Hz), 3.68 (s, 3H), 3.03-2.95 (m, 2H), 2.61 (ddd, 1H, J = 16.8 Hz, J = 10.4 Hz, J = 6.2 Hz), 2.31 (ddd, 1H, J = 16.8 Hz, J = 10.5 Hz, J = 5.4 Hz), 1.40 (t, 3H, J = 7.2 Hz). **^{13}C NMR** ($CDCl_3$, 150 MHz): δ 172.0, 165.6, 163.8 (d,$^1J_{CF}$ = 251.9 Hz), 131.9 (d,$^3J_{CF}$ = 8.7 Hz), 124.0 (d,$^4J_{CF}$ = 3.5 Hz), 120.8 (q,$^1J_{CF}$ = 333.8 Hz), 116.3 (d,$^2J_{CF}$ = 22.2 Hz), 82.3, 64.1, 52.2, 29.6, 28.1, 13.9. **^{19}F NMR** ($CDCl_3$, 376 MHz): δ -67.3, -109.3. **HRMS (ESI)** m/z: $[M+Na]^+$ Calcd for $C_{15}H_{16}F_4O_6SNa$ 423.0496; found 423.0494. **HPLC** (AD, 2-propanol/ n-hexane = 10/90, flow rate 1.0 mL/min, l = 254-220 nm) t_R = 9.5 min (major), t_R = 7.7 min (minor).

(*R*)-1-Ethyl 5-methyl 2-(2-fluorophenyl)-2-((trifluoromethyl)sulfonyl)pentanedioate (8d)

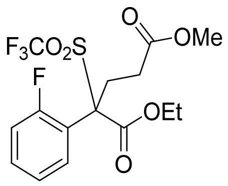

Colourless oil (flash chromatography eluent: hexanes/EtOAc, 98/2 to 90/10), 35.6 mg, 89% yield (96% ee). $[\alpha]_D^{14}$ = -2.92 (*c* 0.49, $CHCl_3$). **^{1}H NMR** ($CDCl_3$, 300 MHz): δ 7.59 (t, 1H, J = 7.7 Hz), 7.48 (q, 1H, J = 8.2 Hz), 7.27 (t, 1H, J = 7.7 Hz), 7.13 (q, 1H, J = 8.3 Hz), 4.40 (q, 2H, J = 7.1 Hz), 3.68 (s, 3H), 3.04-2.99 (m, 2H), 2.71 (ddd, 1H, J = 16.8 Hz, J = 9.3 Hz, J = 6.0 Hz), 2.40 (ddd, 1H, J = 16.8 Hz, J = 9.3 Hz, J = 6.0 Hz), 1.35 (t, 3H, J = 7.1 Hz). **^{13}C NMR** ($CDCl_3$, 62.5 MHz): δ 172.1, 165.6, 161.4 (d,$^1J_{CF}$ = 252.6 Hz), 132.9 (d,$^3J_{CF}$ = 8.5 Hz), 130.7 124.9 (d,$^4J_{CF}$ = 3.1 Hz), 120.8 (q,$^1J_{CF}$ = 334.0 Hz), 117.2 (d,$^2J_{CF}$ = 23.9 Hz), 117.1, 79.5, 63.9, 52.2, 29.4, 28.0, 13.7. **^{19}F NMR** ($CDCl_3$, 376 MHz): δ -67.5, -105.6. **HRMS (ESI)** m/z: $[M+Na]^+$ Calcd for $C_{15}H_{16}F_4O_6SNa$ 423.0496; found 423.0494. **HPLC** (AD, 2-propanol/ n-hexane = 10/90, flow rate 1.0 mL/min, l = 254-220 nm) t_R = 7.6 min (major), t_R = 7.1 min (minor).

(*R*)-1-Ethyl 5-methyl 2-phenyl-2-((trifluoromethyl)sulfonyl)pentanedioate (8e)

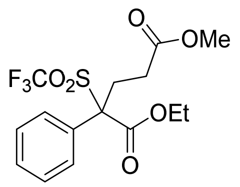

Colourless oil (flash chromatography eluent: hexanes/EtOAc, 98/2 to 90/10), 34.8 mg, 91% yield (96% ee). $[\alpha]_D^{17}$ = +26.45 (*c* 0.54, $CHCl_3$). **^{1}H NMR** ($CDCl_3$, 400 MHz): δ 7.50-7.44 (m, 5H), 4.46 (dq, 2H, *J* = 7.1 Hz, *J* = 1.9 Hz), 3.68 (s, 3H), 3.00 (t, 2H, *J* = 8.0 Hz), 2.65 (ddd, 1H, *J* = 16.8 Hz, *J* = 9.3 Hz, *J* = 7.3 Hz), 2.40 (ddd, 1H, *J* = 16.7 Hz, *J* = 9.1 Hz, *J* = 6.3 Hz), 1.40 (t, 3H, *J* = 7.1 Hz). **^{13}C NMR** ($CDCl_3$,100 MHz): δ 172.2, 165.8, 130.5, 129.4, 129.1, 128.5, 120.8 (q,$^1J_{CF}$ = 334.1 Hz), 83.0, 63.9, 52.1, 29.7, 28.2, 13.9. **^{19}F NMR** ($CDCl_3$, 376 MHz): δ -67.3. **HRMS (ESI)** m/z $[M+Na]^+$ Calcd for $C_{15}H_{17}F_3O_6SNa$ 405.0590; found 405.0602. **HPLC** (AD, 2-propanol/ n-hexane = 10/90, flow rate 1.0 mL/min, l = 254-220 nm) t_R = 7.4 min (major), t_R = 7.0 min (minor).

(*R*)-1-Ethyl 5-methyl 2-(p-tolyl)-2-((trifluoromethyl)sulfonyl)pentanedioate (8f)

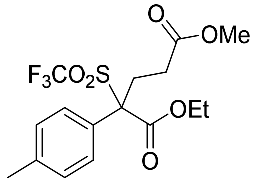

Colourless oil (flash chromatography eluent: hexanes/EtOAc, 98/2 to 90/10), 27.7 mg, 70% yield (96% ee). $[\alpha]_D^{15}$ = +23.12 (*c* 0.49, $CHCl_3$). **^{1}H NMR** ($CDCl_3$, 400 MHz): δ 7.36 (d, 2H, *J* = 8.1 Hz), 7.24 (d, 2H, *J* = 8.0 Hz), 4.45 (q, 2H, *J* = 7.1 Hz), 3.67 (s, 3H), 2.98 (t, 2H, *J* = 8.0 Hz), 2.63 (dt, 1H, *J* = 16.8 Hz, *J* = 8.0 Hz), 2.38 (s, 3H), 2.32 (dt, 1H, *J* = 16.8 Hz, *J* = 8.0 Hz), 1.39 (t, 3H, *J* = 7.1 Hz). **^{13}C NMR** ($CDCl_3$, 62.5 MHz): δ 172.3, 165.9, 140.9, 129.8, 129.4, 125.2, 120.8 (q,$^1J_{CF}$ = 333.6 Hz), 83.0, 63.8, 52.1, 29.7, 28.0, 21.3, 13.9. **^{19}F NMR** ($CDCl_3$, 376 MHz): δ -67.3. **HRMS (ESI)** m/z: $[M+K]^+$ Calcd for $C_{16}H_{19}F_3O_6SK$ 435.0486; found 435.0477. **HPLC** (AD, 2-propanol/ n-hexane = 10/90, flow rate 1.0 mL/min, l = 254-220 nm) t_R = 6.8 min (major), t_R = 6.2 min (minor).

(*R*)-1-Ethyl 5-methyl 2-(o-tolyl)-2-((trifluoromethyl)sulfonyl)pentanedioate (8g)

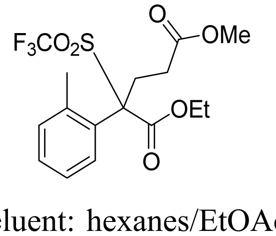

Colourless oil (flash chromatography eluent: hexanes/EtOAc, 98/2 to 90/10), 15.9 mg, 40% yield (92% ee). $[\alpha]_D^{16}$ = -21.90 (*c* 0.60, $CHCl_3$). **^{1}H NMR** ($CDCl_3$, 400 MHz): δ 7.46 (d, 1H, *J* = 7.7 Hz),

7.36 (d, 1H, J = 7.5 Hz), 7.30 (t, 2H, , J = 7.5 Hz), 4.40 (q, 2H, J = 7.2 Hz), 3.69 (s, 3H), 3.17 (ddd, 1H, J = 15.0 Hz , J = 9.5 Hz , J = 5.3 Hz), 3.00 (ddd, 1H, J = 15.1 Hz , J = 9.3 Hz , J = 6.4 Hz), 2.57 (ddd, 1H, J = 15.8 Hz, J = 9.3 Hz, J = 6.3 Hz), 2.52 (ddd, 1H, J = 15.3 Hz, J = 9.4 Hz, J = 5.6 Hz), 2.38 (s, 3H), 1.38 (t, 3H, J = 7.2 Hz). **^{13}C NMR** ($CDCl_3$, 75 MHz): δ 172.3, 166.7, 141.2, 133.6, 130.7, 130.5, 126.4, 125.7, 121.1 (q,$^1J_{CF}$ = 334.0 Hz), 82.8, 63.9, 52.2, 29.5, 28.6, 21.8, 13.8. **^{19}F NMR** ($CDCl_3$, 376 MHz): δ -67.4. **HRMS (ESI)** m/z: $[M+Na]^+$ Calcd for $C_{16}H_{19}F_3O_6SNa$ 419.0747; found 419.0740. **HPLC** (AD, 2-propanol/ n-hexane = 5/95, flow rate 1.0 mL/min, l = 220 nm) t_R = 8.8 min (major), t_R = 8.3 min (minor).

(*R*)-1-Ethyl 5-methyl 2-(3-methoxyphenyl)-2-((trifluoromethyl)sulfonyl)pentanedioate (8h)

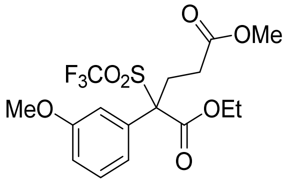

Colourless oil (flash chromatography eluent: hexanes/EtOAc, 98/2 to 85/15), 36.7 mg, 89% yield (94% ee). $[\alpha]_D^{15}$ = +24.12 (*c* 0.49, $CHCl_3$). **^{1}H NMR** ($CDCl_3$, 400 MHz): δ 7.34 (t, 1H, J = 8.3 Hz), 7.04-7.02 (m, 2H), 6.97 (dd, 1H, J = 8.3 Hz, J =2.3 Hz), 4.45 (dq, 2H, J = 7.1 Hz, J = 2.8 Hz), 3.81 (s, 3H), 3.67 (s, 3H), 2.96 (dd, 2H, J = 8.5 Hz, J = 7.5 Hz), 2.67 (ddd, 1H, J = 16.8 Hz, J = 9.3 Hz, J = 7.3 Hz), 2.32 (dt, 1H, J = 16.8 Hz, J = 9.2 Hz, J = 6.3 Hz), 1.39 (t, 3H, J = 7.1 Hz). **^{13}C NMR** ($CDCl_3$, 75 MHz): δ 172.3, 165.7, 159.9, 130.1, 130.0, 121.4, 120.8 (q,$^1J_{CF}$ = 334.3 Hz), 115.7, 115.5, 82.9, 63.8, 55.5, 52.1, 29.7, 28.4, 13.9. **^{19}F NMR** ($CDCl_3$, 376 MHz): δ -67.2. **HRMS (ESI)** m/z: $[M+Na]^+$ Calcd for $C_{16}H_{19}F_3O_7SNa$ 435.0696, found 435.0687. **HPLC** (IC, 2-propanol/ n-hexane = 10/90, flow rate 1.0 mL/min, l = 220 nm) t_R = 12.2 min (major), t_R = 8.8 min (minor).

(*R*)-1-Ethyl 5-methyl 2-(4-(trifluoromethyl)phenyl)-2-((trifluoromethyl)sulfonyl)pentanedioate (8i)

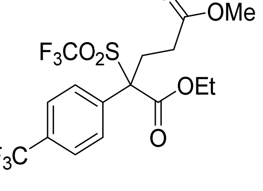

Colourless oil (flash chromatography eluent: hexanes/EtOAc, 98/2 to 90/10), 41.0 mg, 91% yield (93% ee). $[\alpha]_D^{17}$ = +20.32 (*c* 0.52, $CHCl_3$). **^{1}H NMR** ($CDCl_3$, 400 MHz): δ 7.71 (d, 2H, J = 8.5 Hz), 7.64 (d, 2H, J = 8.5 Hz), 4.48 (dq, 2H, J = 7.1 Hz, J = 3.0 Hz), 3.68 (s, 3H), 3.08-2.95 (m, 2H), 2.63 (ddd, 1H, J = 16.7 Hz, J = 10.1 Hz, J = 6.3 Hz), 2.28 (ddd, 1H, J = 16.7 Hz, J = 10.1 Hz, J = 5.2 Hz), 1.41 (t, 3H, J = 7.1 Hz). **^{13}C NMR** ($CDCl_3$, 100 MHz): δ 171.9, 165.2, 132.6, 132.5 (q,$^2J_{CF}$ =

33.2 Hz), 130.1, 126.0 (q,$^{3}J_{CF}$ = 3.6 Hz), 123.5 (q,$^{1}J_{CF}$ = 272.9 Hz), 120.7 (q,$^{1}J_{CF}$ = 333.4 Hz), 82.2, 64.3, 52.2, 29.5, 28.1, 13.9.–^{19}**F NMR** (CDCl$_3$, 376 MHz): δ -63.2, -67.1. **HRMS (ESI)** m/z: $[M+Na]^+$ Calcd for $C_{16}H_{16}F_6O_6SNa$ 473.0464; found 473.0477. **HPLC** (AD, 2-propanol/ n-hexane = 10/90, flow rate 1.0 mL/min, l = 254-220 nm) t_R = 7.6 min (major), t_R = 5.9 min (minor).

(*R*)-1-Ethyl 5-methyl 2-(naphthalen-2-yl)-2-((trifluoromethyl)sulfonyl)pentanedioate (8j)

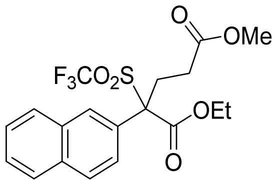

Colourless oil (flash chromatography eluent: hexanes/EtOAc, 98/2 to 90/10), 37.6 mg, 87% yield (92% ee). $[\alpha]_D^{17}$ = +25.53 (*c* 0.58, CHCl$_3$). ^{1}**H NMR** (CDCl$_3$, 300 MHz): δ 7.96-7.85 (m, 4H), 7.61-7.52 (m, 3H), 4.51 (dq, 2H, *J* = 7.2 Hz, *J* = 2.0 Hz), 3.66 (s, 3H), 3.15-3.09 (m, 2H), 2.70 (ddd, 1H, *J* = 16.7 Hz, *J* = 9.5 Hz, *J* = 7.3 Hz), 2.35 (ddd, 1H, *J* = 16.7 Hz, *J* = 9.5 Hz, *J* = 6.7 Hz), 1.43 (t, 3H, *J* = 7.2 Hz). ^{13}**C NMR** (CDCl$_3$, 100 MHz): δ 172.2, 165.9, 133.7, 132.7, 129.9, 129.0, 128.8, 128.1, 127.7, 127.1, 125.7, 125.6, 120.8 (q,$^{1}J_{CF}$ = 334.1 Hz), 83.2, 63.9, 52.1, 29.7, 28.1, 13.9. ^{19}**F NMR** (CDCl$_3$, 376 MHz): δ -67.2. **HRMS (ESI)** m/z: $[M+Na]^+$ Calcd for $C_{19}H_{19}F_3O_6SNa$ 455.0747; found 455.0739. **HPLC** (AD, 2-propanol/ n-hexane = 10/90, flow rate 1.0 mL/min, l = 254-220 nm) t_R = 11.3 min (major), t_R = 10.3 min (minor).

(*R*)-Diethyl 2-(3,4-dichlorophenyl)-2-((trifluoromethyl)sulfonyl)pentanedioate (8k)

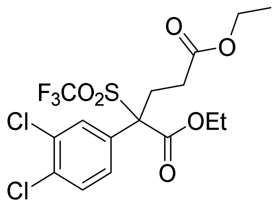

Colourless oil (flash chromatography eluent: hexanes/EtOAc, 98/2 to 90/10), 37.2 mg, 80% yield (91% ee). $[\alpha]_D^{17}$ = +21.81 (*c* 0.48, CHCl$_3$). ^{1}**H NMR** (CDCl$_3$, 400 MHz): δ 7.59 (d, 1H, *J* = 2.2 Hz), 7.53 (d, 1H, *J* = 8.6 Hz), 7.36 (dd, 1H, *J* = 8.6 Hz, *J* = 2.2 Hz), 4.46 (dq, 2H, *J* = 7.1 Hz, *J* = 3.5 Hz), 4.16 (q, 2H, *J* = 7.1 Hz), 3.04-2.88 (m, 2H), 2.58 (ddd, 1H, *J* = 16.7 Hz, *J* = 10.3 Hz, *J* = 6.1 Hz), 2.28 (ddd, 1H, *J* = 16.6 Hz, *J* = 10.0 Hz, *J* = 5.0 Hz), 1.40 (t, 3H, *J* = 7.1 Hz), 1.25 (t, 3H, *J* = 7.3 Hz). ^{13}**C NMR** (CDCl$_3$, 100 MHz): δ 171.4, 165.0, 135.4, 133.6, 131.6, 131.1, 128.8, 128.6, 120.7 (q,$^{1}J_{CF}$ = 333.3 Hz), 81.8, 64.3, 61.3, 29.7, 27.9, 14.3, 13.9. ^{19}**F NMR** (CDCl$_3$, 376 MHz): δ -67.0. **HRMS (ESI)** m/z: $[M+H]^+$ Calcd for $C_{16}H_{17}Cl_2F_3O_6S$ 465.0148; found 465.0135. **HPLC** (AD, 2-propanol/ n-hexane = 5/95, flow rate 1.0 mL/min, l = 220 nm) t_R = 7.4 min (major), t_R = 6.6 min (minor).

(*R*)-5-Butyl 1-ethyl 2-(3,5-dimethylphenyl)-2-((trifluoromethyl)sulfonyl)pentanedioate (8l)

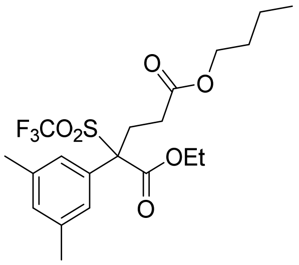

Colourless oil (flash chromatography eluent: hexanes/EtOAc, 98/2 to 90/10), 38.9 mg, 86% yield (93% ee). $[\alpha]_D^{16}$ = +23.38 (*c* 0.54, $CHCl_3$). **^{1}H NMR** ($CDCl_3$, 300 MHz): δ 7.07 (s, 1H), 7.05 (s, 2H), 4.45 (q, 2H, *J* = 7.1 Hz), 4.07 (t, 2H, *J* = 6.7 Hz), 2.96 (t, 2H, *J* = 7.8 Hz), 2.71-2.60 (m, 1H), 2.33 (s, 6H), 2.40-2.25 (m, 1H), 1.58 (sixt, 2H, *J* = 7.6 Hz), 1.42-1.32 (m, 5H), 0.92 (t, 3H, *J* = 7.1 Hz). **^{13}C NMR** ($CDCl_3$, 75 MHz): δ 172.0, 166.0, 138.7, 132.2, 128.3, 126.9, 120.8 (q,$^1J_{CF}$ = 334.3 Hz), 83.1, 65.0, 63.7, 30.7, 29.9, 28.2, 21.6, 19.2, 13.9, 13.8. **^{19}F NMR** ($CDCl_3$, 376 MHz): δ -67.2. **HRMS (ESI)** m/z: $[M+Na]^+$ Calcd for $C_{20}H_{27}F_3O_6SNa$ 475.1373; found 475.1367. **HPLC** (AD, 2-propanol/ n-hexane = 10/90, flow rate 1.0 mL/min, l = 220 nm) t_R = 4.8 min (major), t_R = 3.9 min (minor).

(*R*)-5-Butyl 1-ethyl 2-(4-cyanophenyl)-2-((trifluoromethyl)sulfonyl)pentanedioate (8m)

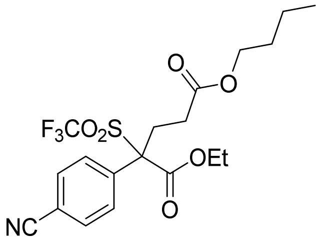

Colourless oil (flash chromatography eluent: hexanes/EtOAc, 98/2 to 85/15), 40.9 mg, 91% yield (84% ee). $[\alpha]_D^{16}$ = +19.75 (*c* 0.61, $CHCl_3$). **^{1}H NMR** ($CDCl_3$, 600 MHz): δ 7.75 (d, 2H, *J* = 8.8 Hz), 7.65 (d, 2H, *J* = 8.8 Hz), 4.48 (q, 2H, *J* = 7.2 Hz), 4.41 (dt, 2H, *J* = 6.8 Hz , *J* = 2.9 Hz), 3.03 (ddd, 1H, *J* = 16.3 Hz, *J* = 10.5 Hz, *J* = 6.3 Hz), 2.98 (ddd, 1H, *J* = 15.3 Hz, *J* = 10.4 Hz, *J* = 5.9 Hz), 2.59 (ddd, 1H, *J* = 16.7 Hz, *J* = 10.6 Hz, *J* = 6.1 Hz), 2.25 (ddd, 1H, *J* = 16.4 Hz, *J* = 10.3 Hz, *J* = 4.8 Hz), 1.59 (quint, 2H, *J* = 6.9 Hz), 1.41 (t, 3H, *J* = 7.2 Hz), 1.36 (quint, 2H, *J* = 7.5 Hz), 0.92 (t, 3H, *J* = 7.5 Hz). **^{13}C NMR** ($CDCl_3$, 150 MHz): δ 171.4, 164.9, 133.7, 132.6, 130.5, 120.7 (q,$^1J_{CF}$ = 333.8 Hz), 117.7, 114.6, 82.2, 65.2, 64.4, 30.7, 29.6, 27.9, 19.2, 13.9, 13.8. **^{19}F NMR** ($CDCl_3$, 376 MHz): δ -67.0. **HRMS (ESI)** m/z: $[M+Na]^+$ Calcd for $C_{19}H_{22}F_3NO_6SNa$ 472.1012; found 472.1009 **HPLC** (AS-H, 2-propanol/ n-hexane = 5/95, flow rate 1.0 mL/min, l = 254-220 nm) t_R = 16.1 min (major), t_R = 14.7 min (minor).

(*R*)-1-Ethyl 5-isopropyl 2-(3-fluorophenyl)-2-((trifluoromethyl)sulfonyl)pentanedioate (8n)

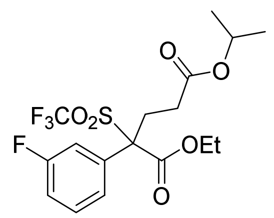

Colourless oil (flash chromatography eluent: hexanes/EtOAc, 98/2 to 90/10), 32.1 mg, 75% yield (83% ee). $[\alpha]_D^{17}$ = +16.56 (*c* 0.47, $CHCl_3$). **^{1}H NMR** ($CDCl_3$, 600 MHz): *δ* 7.43 (dt, 1H, *J* = 8.1 Hz, *J* = 6.0 Hz), 7.29 (dd, 1H, *J* = 8.0 Hz, *J* = 1.2 Hz), 7.25 (dt, 1H, *J* = 10.1 Hz *J* = 2.1 Hz), 7.17 (td, 1H, *J* = 8.1 Hz *J* = 2.1 Hz), 5.00 (sept, 1H, *J* = 6.3 Hz), 4.47 (dq, 2H, *J* = 7.2 Hz, *J* =3.6 Hz), 2.98 ddd (s, 1H, *J* = 16.3 Hz *J* = 10.4 Hz *J* = 5.9 Hz), 2.95 (ddd, 1H, *J* = 14.7 Hz, *J* =10.6 Hz *J* = 4.9 Hz), 2.59 (ddd, 1H, *J* = 16.7 Hz, *J* = 10.7 Hz, *J* = 5.9 Hz), 2.25 (ddd, 1H, *J* = 16.4 Hz *J* = 10.4 Hz *J* = 4.9 Hz), 1.40 (t, 3H, *J* = 7.2 Hz), 1.23 (d, 3H, *J* = 6.3 Hz), 1.21 (d, 3H, *J* = 6.3 Hz). **^{13}C NMR** ($CDCl_3$, 150 MHz): *δ* 171.9, 165.3, 162.7 (d,$^1J_{CF}$ = 248.1 Hz), 131.1 (d,$^3J_{CF}$ = 7.6 Hz), 130.6 (d,$^3J_{CF}$ = 8.2 Hz), 125.2 (d,$^4J_{CF}$ = 2.4 Hz), 120.7 (q,$^1J_{CF}$ = 334.1 Hz), 117.7 (d,$^2J_{CF}$ = 20.9 Hz), 116.9 (d,$^2J_{CF}$ = 24.4 Hz), 82.4, 68.7, 64.1, 30.1, 28.2, 21.9 (2C), 13.9. **^{19}F NMR** ($CDCl_3$, 376 MHz): *δ* -67.2, -110.4. **HRMS (ESI)** m/z: $[M+Na]^+$ Calcd for $C_{17}H_{20}F_4O_6SNa$ 451.0809; found 451.0803. **HPLC** (AD, 2-propanol/ n-hexane = 10/90, flow rate 1.0 mL/min, l = 220 nm) t_R = 5.1 min (major), t_R = 4.5 min (minor).

(*R*)-5-Allyl 1-ethyl 2-(3-chlorophenyl)-2-((trifluoromethyl)sulfonyl)pentanedioate (8o)

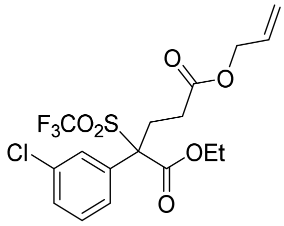

Colourless oil (flash chromatography eluent: hexanes/EtOAc, 98/2 to 90/10), 38.1 mg, 86% yield (94% ee). $[\alpha]_D^{17}$ = +17.78 (*c* 0.60, $CHCl_3$). **^{1}H NMR** ($CDCl_3$, 300 MHz): *δ* 7.48-7.38 (m, 4H), 5.89 (ddd, 1H, *J* = 16.3 Hz *J* = 11.5 Hz *J* = 5.9 Hz), 5.30 (dd, 1H, *J* = 17.1 Hz *J* = 1.2 Hz), 5.24 (dd, 1H, *J* = 11.5 Hz *J* = 1.2 Hz), 4.58 (d, 2H, *J* = 5.8 Hz), 4.47 (dq, 2H, *J* = 7.2 Hz, *J* = 1.9 Hz), 3.05-2.90 (m, 2H), 2.67 (ddd, 1H, *J* = 16.8 Hz, *J* = 10.3 Hz, *J* = 6.3 Hz), 2.32 (ddd, 1H, *J* = 16.6 Hz *J* = 10.1 Hz *J* = 5.9 Hz), 1.40 (t, 3H, *J* = 7.2 Hz) **^{13}C NMR** ($CDCl_3$, 75 MHz): *δ* 171.2, 165.2, 135.2, 131.8, 130.8, 130.6, 130.3, 129.6, 127.5, 120.9 (q,$^1J_{CF}$ = 333.8 Hz), 118.9, 82.2, 65.9, 64.2, 29.7, 28.1, 13.9. **^{19}F NMR** ($CDCl_3$, 376 MHz): *δ* -67.1. **HRMS (ESI)** m/z: $[M+Na]^+$ Calcd for $C_{17}H_{18}ClF_3O_6SNa$ 465.0357; found 465.0351. **HPLC** (OD-H, 2-propanol/ n-hexane = 10/90, flow rate 1.0 mL/min, l = 220 nm) t_R = 5.9 min (major), t_R = 5.4 min (minor).

(*R*)-Ethyl 2-(3-chlorophenyl)-5-morpholino-5-oxo-2-((trifluoromethyl)sulfonyl)pentanoate (8p)

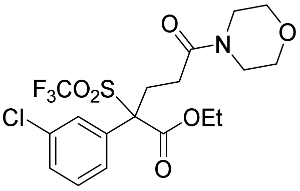

Colourless oil (flash chromatography eluent: hexanes/EtOAc, 98/2 to 90/10), 42.9 mg, 92% yield (93% ee). $[\alpha]_D^{16}$ = -7.59 (*c* 0.54, $CHCl_3$). **^{1}H NMR** ($CDCl_3$, 600 MHz): δ 7.47 (t, 1H, *J* = 1.8 Hz), 7.44 (dt, 1H, *J* = 7.5 Hz *J* = 1.9 Hz), 7.40 (dt, 1H, *J* = 8.1 Hz *J* = 7 .7 Hz), 7.38 (t, 1H, *J* = 7.6 Hz), 4.46 (dq, 2H, *J* = 7.1 Hz, *J* = 3.5 Hz), 3.65-3.57 (m, 6H), 3.39-3.32 (m, 2H), 3.04 (ddd, 1H, *J* = 15.3 Hz, *J* = 10.8 Hz, *J* = 4.7 Hz), 2.99 (ddd, 1H, *J* = 15.2 Hz, *J* = 10.4 Hz *J* = 5.4 Hz), 2.67 (ddd, 1H, *J* = 16.3 Hz, *J* = 10.8 Hz, *J* = 5.6 Hz), 2.24 (ddd, 1H, *J* = 15.7 Hz, *J* = 10.3 Hz, *J* = 4.8 Hz), 1.39 (t, 3H, *J* = 7.1 Hz). **^{13}C NMR** ($CDCl_3$, 150 MHz): δ 169.9, 165.5, 135.1, 131.0, 130.7, 130.2, 129.5, 127.5, 120.7 (q, $^1J_{CF}$ = 334.2 Hz), 82.5, 66.9, 66.5, 64.1, 45.7, 42.2, 28.8, 28.3, 13.8. **^{19}F NMR** ($CDCl_3$, 376 MHz): δ -67.1. **HRMS (ESI)** m/z: $[M+Na]^+$ Calcd for $C_{18}H_{21}ClF_3NO_6SNa$ 494.0622; found 494.0638. **HPLC** (IC, 2-propanol/ n-hexane = 10/90, flow rate 1.0 mL/min, l = 254-220 nm) t_R = 12.1 min (major), t_R = 13.8 min (minor).

Procedure for asymmetric synthesis of α-hydroxyl ester

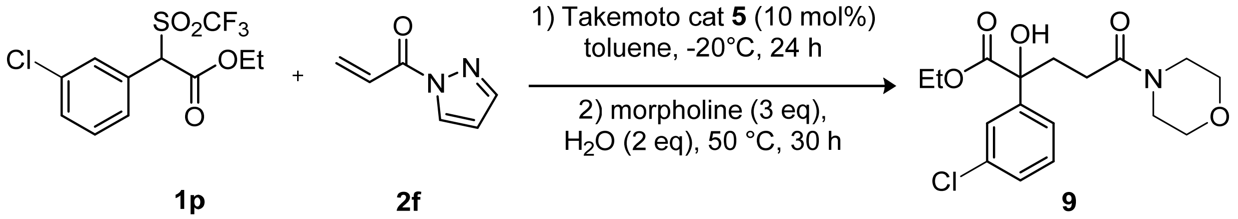

1p **2f** **9**

In an oven-dried vial α-trifluoromethylsulfonyl esters **1p** (0.1 mmol), acrylpyrazole **2f** (0.15 mmol, 18.3 mg) and anhydrous toluene (0.5 mL) were introduced. The reaction mixture was cooled at -20°C and Takemoto catalyst **5** was added (0.01 mmol, 4.13 mg). The reaction mixture was stirred at -20°C for 24 hours and monitored by TLC. After completion of the first step, anhydrous morpholine (0.3 mmol, 26 µL) and water (0.2 mmol, 3.6 µL) were added and the mixture was stirred for 30 hours at 50 °C by using an oil bath and it was monitored by TLC. After completion of the reaction the solvent was evaporated and the crude mixture was purified by flash chromatography (eluent: hexane/ethyl acetate 100/0 to 80/20) to afford product **9** in 95% yield and 92% ee.

(*S*)-Ethyl 2-(3-chlorophenyl)-2-hydroxy-5-morpholino-5-oxopentanoate (9)

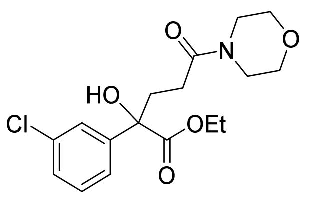

White solid, 33.8 mg, 95% yield (92% ee). **mp** 90.0-90.8°C. $[\alpha]_D^{16}$ = -1.24 (*c* 0.43, $CHCl_3$). **^{1}H NMR** ($CDCl_3$, 300 MHz): δ 7.63-7.61 (m, 1H), 7.50-7.46 (m, 1H), 7.28-7.26 (m, 2H), 4.51 (bs, 1H), 4.26 (dq, 1H, J = 14.1 Hz, J = 7.1 Hz), 4.21 (dq, 1H, J = 14.1 Hz, J = 7.1 Hz), 3.64-3.60 (m, 6H), 3.83 (t, 2H, J = 5.1 Hz), 2.50-2.44 (m, 2H), 2.36-2.30 (m, 2H), 1.27 (t, 3H, J = 7.1 Hz). **^{13}C NMR** (CD_2Cl_2, 100 MHz): δ 174.4, 171.6, 144.2, 134.5, 130.0, 128.1, 126.3, 124.4, 77.9, 67.1, 66.8, 63.0, 46.3, 42.4, 35.1, 27.9, 14.2. **HRMS (ESI)** m/z: $[M+Na]^+$ Calcd for $C_{17}H_{22}ClNO_5Na$ 378.1079; found 378.1070. **HPLC** (AD, 2-propanol/ n-hexane = 20/80, flow rate 1.0 mL/min, l = 220 nm) t_R = 9.2 min (major), t_R = 10.3 min (minor).

Procedure for the asymmetric synthesis of γ-butyrolactone

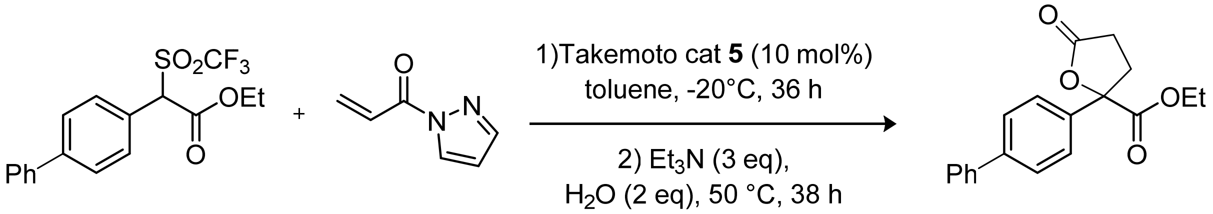

1q **2f** **10**

In an oven-dried vial *α*-trifluoromethylsulfonyl ester **1q** (0.1 mmol), acrylpyrazole **2f** (0.15 mmol, 18.3 mg) and anhydrous toluene (0.5 mL) were introduced. The reaction mixture was cooled at -20°C and Takemoto catalyst **5** was added (0.01 mmol, 4.13 mg). The reaction mixture was stirred at -20°C for 36 hours and monitored by TLC. After completion of the first step, anhydrous triethylamine (0.3 mmol, 42 μL) and water (0.2 mmol, 3.6 μL) were added and the mixture was stirred for 38 hours at 50 °C by using an oil bath and it was monitored by TLC. After completion of the reaction the solvent was evaporated and the crude mixture was purified by flash chromatography (eluent: hexane/ethyl acetate 100/0 to 90/10) to afford product **10** in 44% yield and 95% ee.

(*S*)-Ethyl 2-([1,1'-biphenyl]-4-yl)-5-oxotetrahydrofuran-2-carboxylate (10)

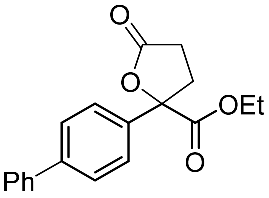

Colourless oil, 13.6 mg, 44% yield (95% ee). $[\alpha]_D^{16}$ = -13.7 (*c* 0.38, $CHCl_3$). **^{1}H NMR** ($CDCl_3$, 400 MHz): δ 7.63-7.58 (m, 6H), 7.45 (t, 2H, *J* = 7.4 Hz), 7.37 (t, 1H, *J* = 7.4 Hz), 4.24 (q, 2H, *J* = 7.1 Hz), 3.13-3.09 (m, 1H), 2.74-2.55 (m, 3H), 1.26 (t, 3H, *J* = 7.1 Hz). **^{13}C NMR** ($CDCl_3$, 100 MHz): δ 175.1, 170.4, 141.8, 140.3, 137.1, 129.0, 127.8, 127.5, 127.2, 125.7, 87.0, 62.8, 33.6, 28.3, 14.1. **HRMS (ESI)** m/z: $[M+Na]^+$ Calcd for $C_{19}H_{18}O_4Na$ 333.1097; found 333.1103. **HPLC** (IC, 2-propanol/ n-hexane = 20/80, flow rate 1.0 mL/min, l = 220 nm) t_R = 12.3 min (major), t_R = 16.9 min (minor).

Table 2. Optimization of the ester reduction

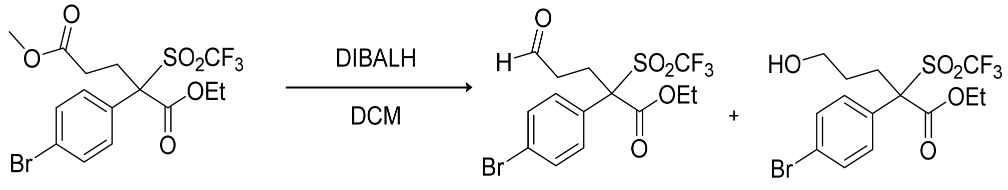

8a **11a** **11**

DIBALH (eq)	C **8a** (M)	Temperature (°C)	Time (min)	Conv **11a** (%)[a]	Conv **11** (%)[a]
2	0.7	-78	150	25	-
2	0.7	-90	15	44	22
1	0.2	-90	15	95	-
1	0.2	-90	15	90	-
2	0.2	-20	30	20	80

Unless otherwise noted reactions were conducted with **8a** (0.1 mmol), DIBALH in anhydrous DCM under nitrogen atmosphere. [a] Determined by ^{19}F-NMR spectroscopy of crude reaction mixture using α,α,α-trifluorotoluene as internal standard.

Procedure for reduction of enantioenriched 8a to alcohol

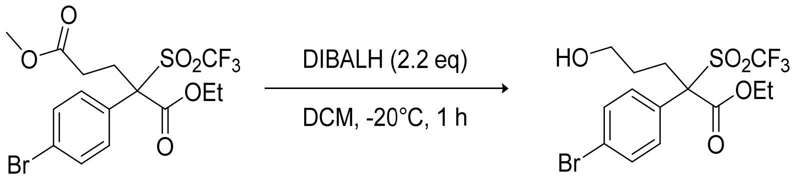

8a **11**

In an oven-dried vial enantioenriched trifluoromethylsulfonyl diesters **8a** (0.1 mmol, 93% ee) was dissolved in anhydrous CH_2Cl_2 (0.5 mL) and cooled at -20°C. To this mixture, DIBALH 1.0 M in toluene (2.2 eq, 220 µL) was added dropwise in 15 minutes, and the reaction was stirred at the same temperature for 1 hour and monitored by TLC. After completion of the reaction, the mixture was quenched by adding MeOH (1 mL) and stirring at room temperature for 15 minutes. The crude mixture was then filtered on celite pad followed by solvent evaporation. The crude mixture was

purified by flash chromatography (eluent: hexane/ethyl acetate 100/0 to 80/20) to afford product **11** in 76% yield and 93% ee.

(*R*)-Ethyl 2-(4-bromophenyl)-5-hydroxy-2-((trifluoromethyl)sulfonyl)pentanoate (11)

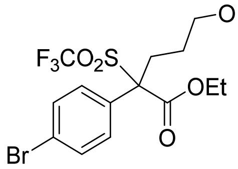

Colourless oil, 32.9 mg, 76% yield (93% ee). $[\alpha]_D^{14}$ = +5.59 (*c* 0.59, $CHCl_3$). **^{1}H NMR** ($CDCl_3$, 300 MHz): δ 7.57 (d, 2H, J = 8.8 Hz), 7.41 (d, 2H, J = 8.8 Hz), 4.45 (dq, 2H, J = 7.2 Hz, J = 3.5 Hz), 3.74-3.66 (m, 2H), 2.76-2.74 (m, 2H), 1.74-1.67 (m, 1H), 1.58-1.52 (m, 1H), 2.40-2.25 (m, 1H), 1.39 (t, 3H, J = 7.1 Hz). **^{13}C NMR** ($CDCl_3$, 150 MHz): δ 165.6, 132.1, 131.6, 127.7, 125.1, 120.8 (q, $^1J_{CF}$ = 334.1 Hz), 83.1, 63.9, 62.0, 29.4, 27.8, 14.0. **^{19}F NMR** ($CDCl_3$, 376 MHz): δ -67.5. **HRMS (ESI)** m/z: $[M+H]^+$ Calcd for $C_{14}H_{17}BrF_3O_5S$ 432.9927; found 432.9903. **HPLC** (IE-3, 2-propanol/ n-hexane = 10/90, flow rate 1.0 mL/min, l = 254-220 nm) t_R = 12.3 min (major), t_R = 10.1 min (minor).

Procedure for enantioselective one-pot Michael addition/reduction/Horner-Emmons olefination

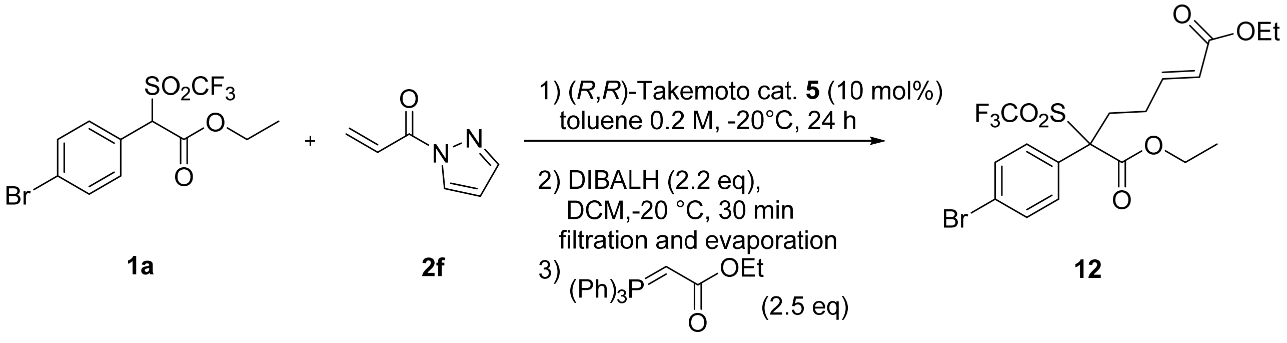

1a **2f** **12**

In an oven-dried vial, α-trifluoromethylsulfonyl ester **1a** (0.1 mmol), the acrylpyrazole **2f** (0.15 mmol) and anhydrous toluene (0.5 mL) were introduced. The reaction mixture was cooled at -20°C and Takemoto catalyst **5** was added (0.01 mmol, 4.13 mg). The reaction mixture was stirred at -20°C for 24 h and monitored by TLC, as reported in Scheme 4. After completion of the first step, the solvent was evaporated and the reaction mixture was dissolved in CH_2Cl_2 (0.5 mL). The mixture was cooled at -20°C and DIBALH 1.0 M in toluene (2.2 eq, 220 µL) was added dropwise in 15 minutes. After the addition, the reaction was stirred at the same temperature for 30 minutes and monitored by TLC. After completion, the reaction was quenched by adding MeOH (1.0 mL). Then the mixture was filtered on celite pad and the solvent was evaporated. The crude mixture was

dissolved in toluene and (carbethoxymethylene)triphenylphosphorane (2.5 eq) was added. The reaction was stirred for 2 hours at room temperature and the crude product was purified by flash chromatography (eluent: hexane/ethyl acetate 100/0 to 90/10) to afford product **12** in 50% yield and 93% ee.

(*R*)-Diethyl (E)-6-(4-bromophenyl)-6-((trifluoromethyl)sulfonyl)hept-2-enedioate (12)

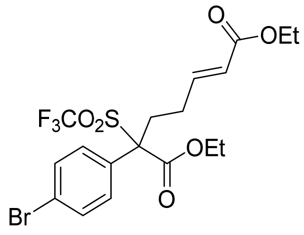

Colourless oil, 25.1 mg, 50% yield (93% ee). $[\alpha]_D^{17}$ = +23.63 (*c* 0.32, $CHCl_3$). **^{1}H NMR** ($CDCl_3$, 400 MHz): δ 7.58 (d, 2H, *J* = 8.7 Hz), 7.36 (d, 2H, *J* = 8.7 Hz), 6.87 (dt, 1H, *J* = 15.7 Hz, *J* = 6.8 Hz), 5.83 (d, 1H, *J* = 15.7 Hz), 4.46 (dq, 2H, *J* = 7.2, Hz *J* = 4.7 Hz), 4.41 (q, 2H, *J* = 7.1 Hz), 2.80 (ddd, 1H, *J* = 16.3 Hz, *J* = 11.3 Hz, *J* = 5.1 Hz), 2.67 (ddd. 1H, *J* = 15.9 Hz, *J* = 11.4 Hz, *J* = 4.4 Hz), 2.47-2.38 (m, 1H), 2.20-2.11 (m, 1H), 1.40 (t, 3H, *J* = 7.1 Hz), 1.28 (t, 3H, *J* = 7.1 Hz). **^{13}C NMR** ($CDCl_3$, 100 MHz): δ 166.2, 165.2, 145.4, 132.3, 131.1, 127.6, 125.3, 123.0, 120.7 (q, $^1J_{CF}$ = 332.7 Hz), 82.6, 64.1, 60.6, 31.3, 27.4, 14.4, 13.9. **^{19}F NMR** ($CDCl_3$, 376 MHz): δ -67.2. **HRMS (ESI)** m/z: $[M+Na]^+$ Calcd for $C_{18}H_{20}BrF_3O_6SNa$ 523.0008; found 523.0035. **HPLC** (IC, 2-propanol/ n-hexane = 10/90, flow rate 1.0 mL/min, l = 254-220 nm) t_R = 7.9 min (major), t_R = 9.9 min (minor).

X-Ray Data for the Absolute Configuration Assignment of Compound 9

Single crystals of compound **9** were obtained by slow evaporation of a solution of n-hexane/$CHCl_3$ at room temperature.

Single crystal diffraction data were collected on an Rigaku Oxford Diffraction Supernova CCD area detector diffractometer, using Mo Kα (λ = 0.71073 Å) radiation. Data reduction and absorption correction were performed using CrysalisPro. The structure was solved by direct methods using SHELXT[4] and refined by full-matrix least squares using SHELXL.[5] Hydrogen atoms were generated in calculated position. The correct absolute structure was confirmed by the very low value of the Flack parameter 0.00(5).[6]

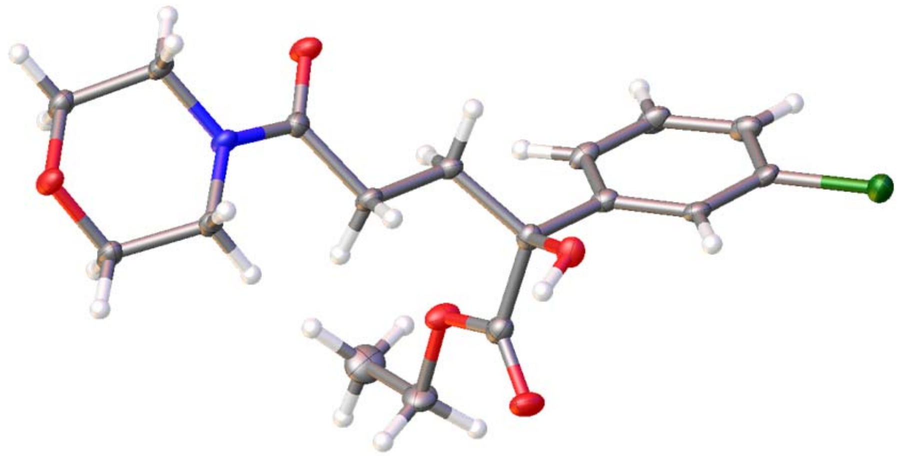

Figure 1. ORTEP drawing of compound **9**, showing 50% probability ellipsoids. The color codes used for plotting the structures are green: Cl; red: O; grey: C; blue: N; white: H.

[4] Sheldrick, G., SHELXT - Integrated space-group and crystal-structure determination. *Acta Crystallogr. Sect. A,* **2015**, *71* (1), 3.

[5] Sheldrick, G., Crystal structure refinement with SHELXL. *Acta Crystallogr. Sect. C,* **2015**, *71* (1), 3.

[6] Parsons, S.; Flack, H. D.; Wagner, T., Use of intensity quotients and differences in absolute structure refinement. *Acta Crystallogr. Sect. B,* **2013**, *69* (3), 249.

Item	Value
**Molecular formula**	$C_{17}H_{22}ClNO_5$
**Formula weight**	355.82
**Crystal system**	Monoclinic
**Space Group**	$P2_1$
**a (Å)**	5.8005(3)
**b (Å)**	9.2334(6)
**c (Å)**	16.1751(10)
**α (°)**	90
**β (°)**	99.046(5)
**γ (°)**	90
**Volume (Å^3)**	855.54(9)
**Z**	2
**T (K)**	109(1)
**ρ (g cm^{-1})**	1.381
**λ (Å)**	0.71073
**μ (mm^{-1})**	0.250
**# measured refl**	9723
**# unique refl**	4235
**R_{int}**	0.056
**# parameters**	222
**R(F^2), all refl**	0.0751
**R_w(F^2), all refl**	0.1286
**Goodness of fit**	1.005

Computational Details

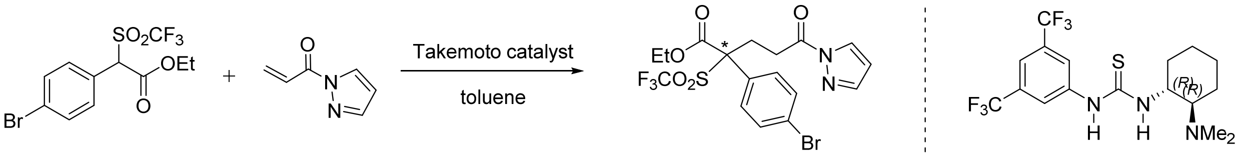

1a **2f** **8a**

Preliminary energy structures related to substrates **1a**, **2f, 8a** and Takemoto catalyst (*R*,*R*)-**5** were obtained by MonteCarlo conformational analysis performed with Molecular Mechanics calculations using the OPLS_2005 force field of the Macromodel package in the Schrodinger suite. Subsequently, the geometries obtained were fully optimized as minimum or transition state using PM6 semiempirical calculations, then with M062X functional with 6-31G(d) basis set, and then further refined at M062X / 6-31G(p,d) level of theory, employing the polarizable continuum model (PCM) for toluene implemented in Gaussian16, in order to take into account the effects of the reaction solvent. This methodology is well supported in the literature and has been shown to accurately describe the energetics of other bifunctional thiourea-catalyzed reactions. All minima and transitions states were also confirmed by frequency analysis. Default convergence and integration settings were used.

Conformational analysis of acyl pyrazole 2f

Three different conformations for compound 2f are possible, according to the different orientation of C=O and C=C double bond. The "s-cis" is the conformation where both double bonds are on the same side of the sigma bond, whereas in the "s-trans" conformation the two double bonds are oriented in a different side. In the latter case, two conformers are available (s-trans$_1$ and s-trans-$_2$) according to the orientation of the pyrazole ring. Even if the configuration s-trans$_2$ present lowest energy, the other structures have been considered according to the proposed interaction mechanisms that will be discussed in the next section.

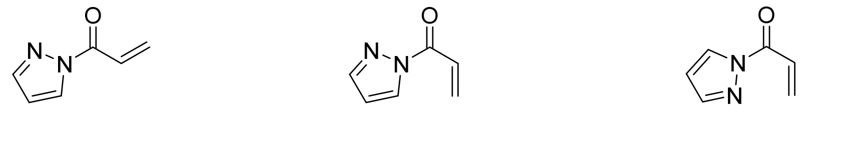

2f *s-cis* $\Delta\Delta G^0$ = 0.94 kcal mol^{-1} | **2f** *s-trans$_1$* $\Delta\Delta G^0$ = 3.05 kcal mol^{-1} | **2f** *s-trans$_2$* $\Delta\Delta G^0$ = 0 kcal mol^{-1}

Plausible modes of dual activation

The enantioselective Michael addition of triflone **1a** to acyl pyrazole **2f** was investigated by DFT calculations. Different interaction models based on dual bifunctional activation have been computationally evaluated, in order to elucidate the reaction mechanism and to rationalize the steric

outcome of the transformation. Five different coordination-models have been considered, based on literature data:

a) **Takemoto-like approach**:[7]

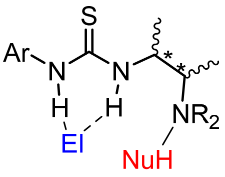

In this approach, by analogy with the mechanism originally proposed by Takemoto et al for the enantioselective Michael addition of malonates to nitroolefin, acyl pirazole (which acts as electrophile, El) interacts with the thiourea moiety of the catalyst through the formation of two hydrogen bonds whereas the neutral triflones (which acts as nucleophile, NuH) is coordinated by one hydrogen bond to the tertiary amino group. As resulting, a ternary complex is formed, wherein both substrates are activated, leading to the formation of the new C−C bond to produce the desired adduct.

b) **Papai-like approach**:[8]

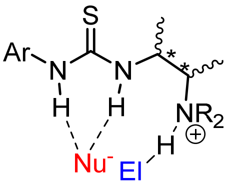

In this approach, by analogy with the mechanism originally proposed by Papai et al. for the enantioselective Michael addition of malonates to nitroolefin, the deprotonated form of triflones, (Nu^{-}) is coordinated to the thiourea moiety of the catalyst through the formation of two hydrogen bonds whereas the acyl pyrrole is coordinated by a single hydrogen bond to the protonated nitrogen of the tertiary amino group.

c) **Izzo-like approach**:[9]

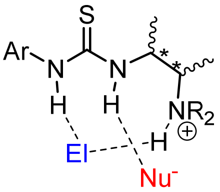

In this approach, by analogy with the mechanism originally proposed by Izzo e al for the enantioselective Michael addition of alonates to nitroolefin, the deprotonated form of triflones, (Nu^{-}) is coordinated both to the N-H group of the thiourea moiety and to the protonated nitrogen of the tertiary amino group, while the carboxylic group of the acyl pyrrole is coordinated to the N-H group of the thiourea moiety.

d) **Wong-Zong approach**:[10]

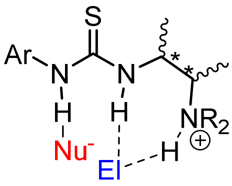

In this approach, by analogy with the mechanism originally proposed by Zhong et al for the Cinchona Alkaloid-Squaramide Catalyzed Sulfa-Michael Addition Reaction: the electrophile interacts with to the protonated nitrogen of the tertiary amino group and one of the N−H groups of the

---

[7] T. Okino, Y. Oashi, Y. Takemoto, *J. Am. Chem. Soc.* **2003**, *125*, 12672.
[8] A. Hamza, G. Schubert, T. Soós, I. Pápai, *J. Am. Chem. Soc.* **2006**, *128*, 13151
[9] J. Izzo, Y. Myshchuk, J. S. Hirschi, M. J. Vetticatt, *Org. Biomol. Chem.* **2019**, *17*, 3934.
[10] B. Tan, Y. Lu, X. Zeng, P. J. Chua, G. Zhong, *Org. Lett.* **2010**, *12*, 2682.

thiourea moiety, while the nucleophile interacts with the distal N−H group of the thiourea moiety.

e) **Alternative approach:**

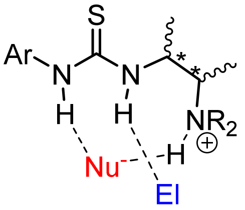

In this alternative approach, the deprotonated form of triflones, (Nu$^-$) is coordinated to the distal N−H group of the thiourea moiety and to the protonated nitrogen of the tertiary amino group, while the carboxylic group of the acyl pyrrole is coordinated to the N-H group of the thiourea moiety.

For each of these approaches, it is possible to hypothesize up to eight different transitions states, depending on the geometry orientation of the two substrates with respect to the catalyst.

Transition states calculated according to Takemoto-like approach:

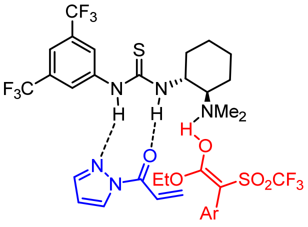

s-cis-pro(S)-1
TS1

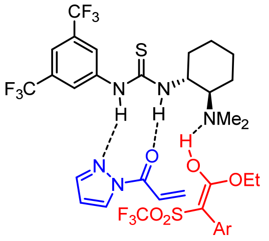

s-cis-pro(R)-1
TS2

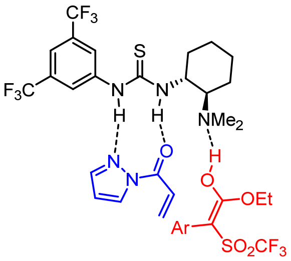

s-trans-pro(S)-1
TS3

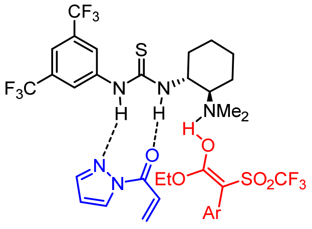

s-trans-pro(R)-1
TS4

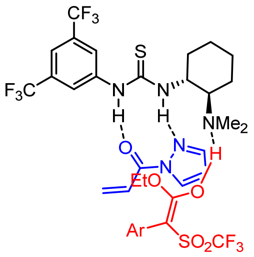

s-cis-pro(S)-2
TS5

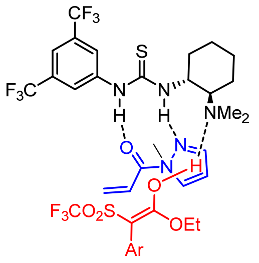

s-cis-pro(R)-2
TS6

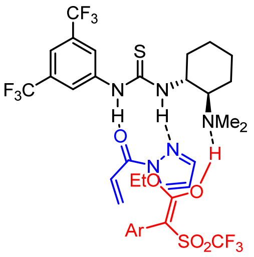

s-trans-pro(S)-2
TS7

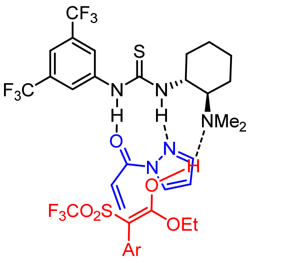

s-trans-pro(R)-2
TS8

Transition states calculated according to Papai-like approach:

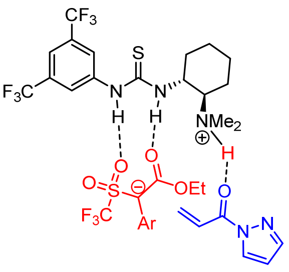

s-cis-pro(S)-1
TS9

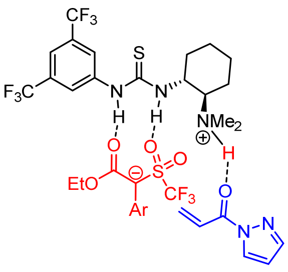

s-cis-pro(R)-1
TS10

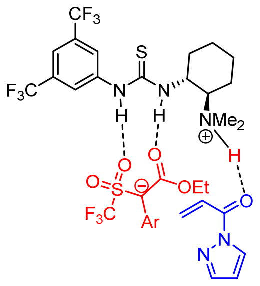

s-trans-pro(S)-1
TS11

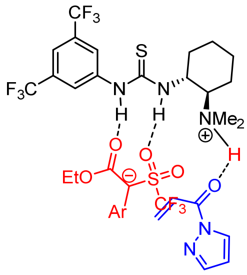

s-trans-pro(R)-1
TS12

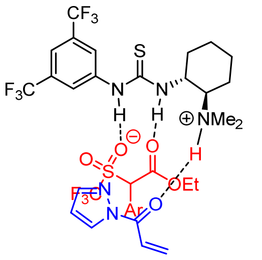

s-cis-pro(S)-2
TS13

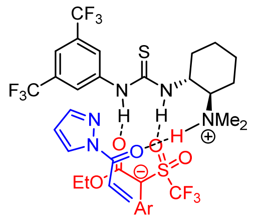

s-cis-pro(R)-2
TS14

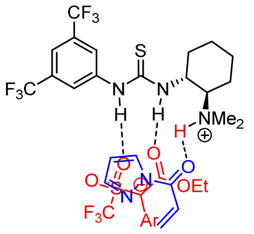

s-trans-pro(S)-2
TS15

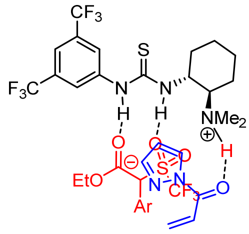

s-trans-pro(R)-2
TS16

Transition states calculated according to Izzo-like approach:
In this case, only 4 of the 8 possible transition states have been located. We were unable to locate the missing transition states.

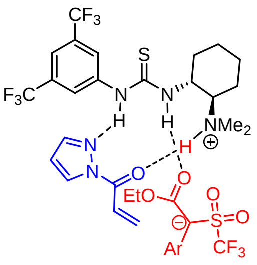

s-cis-pro(R)-1
TS17

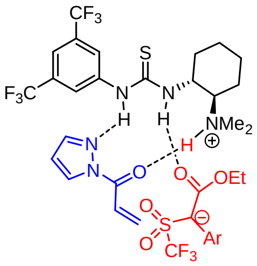

s-cis-pro(S)-1
TS18

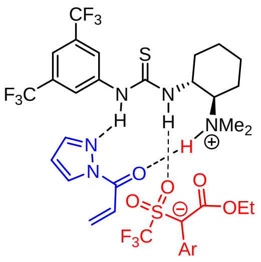

s-trans-pro(S)-1
TS19

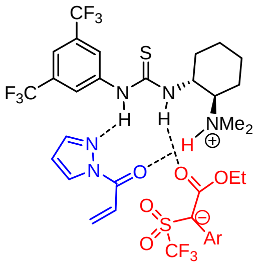

s-trans-pro(R)-1
TS20

Transition states calculated according to Wong-Zong like approach:
In this case, only 5 of the 8 possible transition states have been located. We were unable to locate the missing transition states.

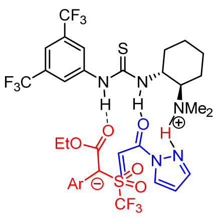

s-trans-pro(S)-1
TS21

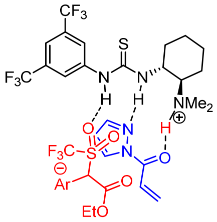

s-cis-pro(S)-2
TS22

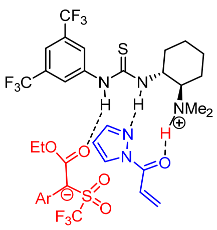

s-cis-pro(R)-2
TS23

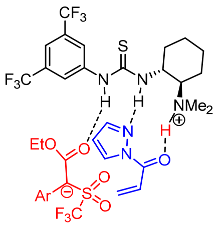

s-trans-pro(S)-2
TS24

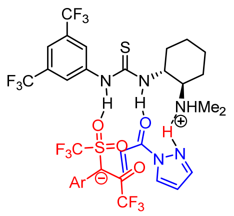

s-trans-pro(R)-1
TS25

Transition states calculated according to alternative approach (e):

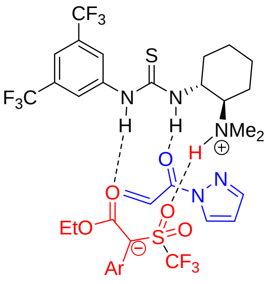

s-cis-pro(S)-1
TS26

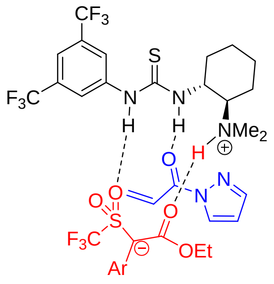

s-cis-pro(R)-1
TS27

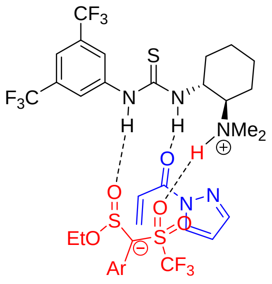

s-trans-pro(S)-1
TS28

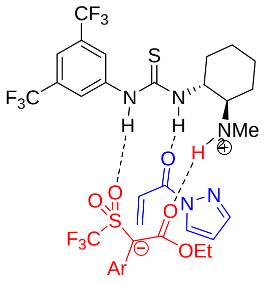

s-trans-pro(R)-1
TS29

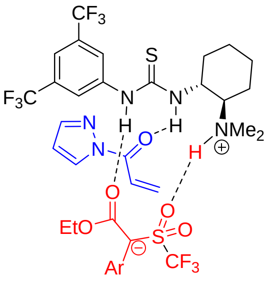

s-cis-pro(S)-2
TS30

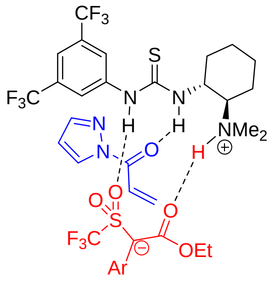

s-cis-pro(R)-2
TS31

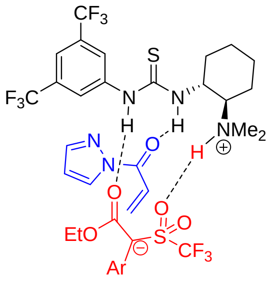

s-trans-pro(S)-2
TS32

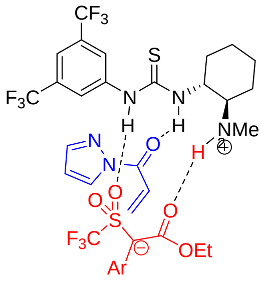

s-trans-pro(R)-2
TS33

All the energy structures from our thorough explorations are shown in Table 3 along with their relative Gibbs free energies ($\Delta\Delta G^{\ddagger}$). As result, the lowest energy transition structure akin to Papai like binding approach is **TS14** leading to the major enantiomer (*R*)-**8a**. This transition state is most sable of 1.64 kcal mol^{-1} compared and **TS13** which is responsible of the formation of the minor enantiomer (*S*)-**8a**.

Table 3. Energetic of different transition states

TS	$\Delta\Delta G^{\ddagger}$ ($kcalmol^{-1}$)	**8a** configuration
1	6,76	(*S*)
2	12,43	(*R*)
3	12,72	(*S*)
4	14,72	(*R*)
5	7,30	(*S*)
6	12,98	(*R*)
7	12,77	(*S*)
8	14,75	(*R*)
9	11,36	(*S*)
10	4,92	(*R*)
11	14,78	(*S*)
12	10,09	(*R*)
13	1,64	(*S*)

14	0,00	(*R*)
15	10,55	(*S*)
16	4,24	(*R*)
17	11,71	(*R*)
18	9,23	(*S*)
19	17,84	(*S*)
20	7,47	(*R*)
21	20,76	(*S*)
22	14,44	(*S*)
23	18,92	(*R*)
24	13,43	(*S*)
25	20,25	(*R*)
26	12,07	(*S*)
27	14,89	(*R*)
28	10,56	(*S*)
29	17,90	(*R*)
30	19,19	(*S*)
31	21,39	(*R*)
32	8,70	(*S*)
33	19,15	(*R*)

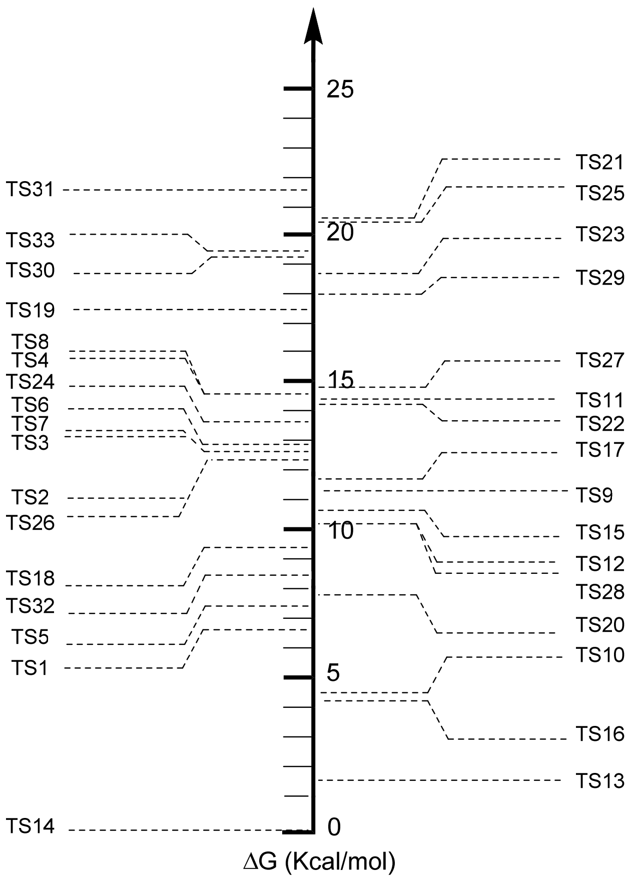

The enantiomeric excess of the Michael addition of triflone **1a** to acyl pyrazole **2f** catalyzed by (*R*,*R*)-**5** catalyst was evaluated[11], considering the Gibbs free energies of all 33 transition states previously reported. When T = 273.15 K, the predicted enantiomeric excess for *R*-**8a** enantiomer was 91%, in good agreement with the experimental data (See Table 1, entry 15).

[11] Q. Peng, F. Duarte, R. S. Paton, *Chem. Soc. Rev.* **2016**, *45*, 6093.

Geometries in XYZ file format

TS1

C	-2.87200	1.81900	-0.29500
S	-4.09200	2.92000	0.10100
N	-1.58200	2.20200	-0.52200
N	-3.06400	0.48000	-0.40700
C	-4.23000	-0.22300	-0.03900
C	-4.84100	-0.02900	1.20000
C	-4.74300	-1.17500	-0.92000
C	-5.85900	-1.92200	-0.55500
C	-5.96800	-0.77000	1.53400
C	-6.49000	-1.72500	0.66700
C	-6.64800	-0.49700	2.84500
C	-6.35800	-2.98800	-1.48600
F	-7.39300	-1.53600	3.24700
F	-5.76000	-0.23500	3.81600
F	-7.46300	0.56500	2.76700
F	-7.65800	-3.25100	-1.30000
F	-5.69900	-4.15000	-1.30000
F	-6.18700	-2.65000	-2.77000
C	-1.22600	3.53300	-0.99500
C	-1.20000	3.58600	-2.52700
C	0.14200	3.93500	-0.44100
C	-0.78800	4.97000	-3.02400
C	0.56200	5.37700	-2.43300
C	0.53800	5.33500	-0.90000
N	0.16600	3.77200	1.06800
C	-0.97800	4.39600	1.78800
C	1.47100	4.19400	1.65500
H	-4.26400	-1.32700	-1.88100
H	-4.43700	0.69700	1.89500
H	-7.36600	-2.30100	0.94000
H	-2.32400	-0.08600	-0.84200
H	-0.95200	1.46000	-0.84000
H	-1.99400	4.22500	-0.63700
H	-2.19500	3.31300	-2.89100
H	-0.49700	2.82600	-2.89400
H	-1.55200	5.70200	-2.73000
H	-0.74300	4.97800	-4.11600
H	0.90300	3.22200	-0.77900
H	0.84300	6.38100	-2.76200
H	1.52900	5.59200	-0.51800

H	-0.17600	6.08000	-0.52400
H	-1.00800	5.46000	1.54900
H	-1.91100	3.91400	1.49400
H	-0.81100	4.26700	2.85800
H	2.27100	3.83900	1.00700
H	1.56000	3.73800	2.64100
H	1.48200	5.28100	1.74000
H	1.34200	4.69200	-2.78700
H	0.12200	2.75100	1.19700
S	3.99400	1.72600	-0.62300
C	3.17000	0.23900	-0.26400
O	1.53700	-0.84400	0.96300
C	1.98500	0.36100	0.59100
O	1.42500	1.39900	0.93100
C	0.60000	-0.88600	2.05600
C	-0.83200	-0.65900	1.62400
O	3.06100	2.81700	-0.92200
C	4.82800	2.26900	0.95000
F	4.00100	2.10800	1.98400
F	5.92200	1.55500	1.16100
F	5.14600	3.55500	0.85600
O	5.09200	1.45800	-1.54500
C	4.80800	-1.53100	-1.04400
C	4.10400	-0.90500	-0.01000
C	4.33100	-1.35000	1.30100
C	5.20100	-2.39600	1.57400
C	5.87200	-3.00500	0.51900
C	5.69000	-2.57700	-0.78800
Br	7.06300	-4.43400	0.87500
H	0.90900	-0.14300	2.79700
H	0.73400	-1.88300	2.47700
H	-1.12800	-1.36600	0.84500
H	-1.49400	-0.79400	2.48500
H	-0.95900	0.35400	1.23900
H	3.81800	-0.86400	2.12600
H	5.36600	-2.73000	2.59200
H	6.22900	-3.05400	-1.59800
H	4.69100	-1.19200	-2.06600
C	2.23600	-0.06300	-2.17900
C	1.52400	-1.24900	-2.18500
C	0.17500	-1.17900	-1.76000
O	-0.42400	-0.14300	-1.44500
N	-0.58500	-2.39000	-1.70000

N	-1.89800	-2.31500	-1.39000
C	-2.32600	-3.56400	-1.39600
C	-1.28700	-4.47300	-1.71300
C	-0.18600	-3.68000	-1.90000
H	1.99900	-2.18900	-2.42600
H	-3.36400	-3.77700	-1.18000
H	-1.34100	-5.54700	-1.78900
H	0.83400	-3.93100	-2.14400
H	3.18400	0.01100	-2.70200
H	1.67500	0.86500	-2.11200

TS2

C	1.96300	-1.87600	-0.56200
S	2.65600	-3.42200	-0.55000
N	0.61500	-1.66700	-0.56300
N	2.69600	-0.73500	-0.56200
C	4.08900	-0.66300	-0.34200
C	4.67400	-1.27400	0.77000
C	4.87700	0.08700	-1.21100
C	6.23900	0.23000	-0.95600
C	6.03700	-1.13800	0.99100
C	6.83600	-0.38300	0.13700
C	6.68000	-1.83900	2.15400
C	7.05800	1.12300	-1.84200
F	7.62500	-1.07600	2.72500
F	5.78900	-2.15700	3.10300
F	7.27800	-2.97800	1.77600
F	8.34600	0.75900	-1.86900
F	7.02500	2.40000	-1.40700
F	6.60600	1.13200	-3.10100
C	-0.34600	-2.70600	-0.90100
C	-0.80700	-2.63900	-2.35900
C	-1.56600	-2.64500	0.02800
C	-1.75000	-3.80500	-2.66300
C	-2.94900	-3.79200	-1.71700
C	-2.52500	-3.80200	-0.24200
N	-1.09200	-2.57400	1.47000
C	-0.13800	-3.64500	1.87600
C	-2.21800	-2.48500	2.43700
H	4.41900	0.57000	-2.06700
H	4.06300	-1.85200	1.45200
H	7.89800	-0.27100	0.32500
H	2.23800	0.15000	-0.82000

H	0.30300	-0.70000	-0.68200
H	0.16400	-3.66300	-0.76100
H	0.08000	-2.66800	-2.99900
H	-1.31300	-1.67900	-2.53700
H	-1.19900	-4.74800	-2.55200
H	-2.08900	-3.75500	-3.70200
H	-2.08900	-1.69000	-0.11200
H	-3.61000	-4.64300	-1.90400
H	-3.42200	-3.71500	0.38000
H	-2.04100	-4.75900	-0.00900
H	-0.62400	-4.61300	1.75800
H	0.76700	-3.59700	1.27000
H	0.11100	-3.48800	2.92600
H	-2.90800	-1.72400	2.08300
H	-1.81100	-2.20000	3.40800
H	-2.70700	-3.45600	2.50900
H	-3.54000	-2.88800	-1.90300
H	-0.61500	-1.66100	1.50900
C	-2.86100	1.74200	0.39100
O	-0.99900	2.28600	1.69700
C	-1.69800	1.29300	1.13200
O	-1.34600	0.11300	1.22300
C	-0.00100	1.90800	2.66500
C	1.35000	1.66500	2.02500
H	-0.35500	1.02000	3.19400
H	0.03200	2.74800	3.36000
H	1.74200	2.58500	1.58600
H	2.05900	1.32100	2.78300
H	1.27500	0.90400	1.24300
C	-1.83900	2.41700	-1.50200
C	-0.67100	3.09700	-1.26700
C	0.51800	2.32700	-1.14700
O	0.59500	1.09900	-1.25700
N	1.73700	3.02800	-0.89200
N	2.88700	2.32600	-0.84800
C	3.81600	3.20100	-0.51200
C	3.27000	4.49500	-0.32100
C	1.93300	4.34300	-0.57300
H	-0.67800	4.16400	-1.10200
H	4.84800	2.88600	-0.43000
H	3.78500	5.40100	-0.04200
H	1.12000	5.05100	-0.53700
H	-2.73100	2.95700	-1.79400

H	-1.76900	1.37700	-1.80800
C	-3.86600	0.66200	0.12300
C	-4.21400	0.27800	-1.17500
C	-4.43000	-0.04200	1.19500
C	-5.01600	-0.83700	-1.40400
H	-3.84800	0.83800	-2.02800
C	-5.22500	-1.16300	0.98700
H	-4.21700	0.29000	2.20800
C	-5.47200	-1.57400	-0.31800
H	-5.25900	-1.14900	-2.41400
H	-5.63100	-1.72500	1.82200
Br	-6.38500	-3.20300	-0.61800
S	-3.54000	3.23000	1.03500
O	-4.01100	3.07900	2.41500
O	-2.75700	4.40900	0.67900
C	-5.11200	3.42400	0.05800
F	-6.05500	2.59100	0.47500
F	-5.52500	4.67000	0.23700
F	-4.90700	3.22400	-1.24600

TS3

C	-3.24400	1.81000	-0.05400
S	-4.43000	2.87800	0.50300
N	-1.96500	2.19400	-0.31300
N	-3.42200	0.48700	-0.28600
C	-4.53900	-0.33400	-0.06900
C	-5.47200	-0.15400	0.95900
C	-4.65100	-1.44800	-0.90500
C	-5.66300	-2.37700	-0.70100
C	-6.49300	-1.08400	1.11900
C	-6.60300	-2.20700	0.30600
C	-7.52800	-0.85000	2.18400
C	-5.68000	-3.60200	-1.57000
F	-8.56400	-0.13700	1.71700
F	-7.02800	-0.17600	3.22900
F	-8.02800	-2.00500	2.64900
F	-6.79800	-4.32000	-1.41100
F	-4.64300	-4.41000	-1.28500
F	-5.57600	-3.29000	-2.86900
C	-1.54900	3.57300	-0.50100
C	-1.56100	3.98400	-1.97800
C	-0.13900	3.72900	0.06800
C	-1.04500	5.41300	-2.14600

C	0.35300	5.55900	-1.54500
C	0.37300	5.15900	-0.06400
N	-0.10900	3.21200	1.49900
C	-1.18100	3.74400	2.38600
C	1.23300	3.37800	2.12300
H	-3.93200	-1.58800	-1.70600
H	-5.39400	0.69400	1.62500
H	-7.39200	-2.93300	0.46000
H	-2.62600	-0.02500	-0.69100
H	-1.41500	1.53500	-0.87500
H	-2.25100	4.21400	0.04200
H	-2.58700	3.88600	-2.34600
H	-0.94300	3.27600	-2.54300
H	-1.73000	6.11100	-1.64800
H	-1.03100	5.68400	-3.20600
H	0.54600	3.05100	-0.45700
H	0.71500	6.58600	-1.64700
H	1.39500	5.23700	0.31600
H	-0.25900	5.84800	0.51100
H	-1.11200	4.83100	2.40900
H	-2.15900	3.43000	2.01900
H	-1.01600	3.34400	3.38700
H	1.27000	2.75800	3.01800
H	1.98600	3.06000	1.40100
H	1.36700	4.42500	2.39300
H	1.05400	4.91300	-2.08900
H	-0.25400	2.19800	1.38800
C	3.20500	0.07000	-0.45600
O	0.78500	0.75900	0.47500
O	2.72200	2.43500	-0.59400
C	5.58600	-0.20300	0.31300
C	4.39000	-0.78600	-0.13500
C	4.33400	-2.18300	-0.23000
C	5.43600	-2.97000	0.09200
C	6.60900	-2.35900	0.51100
C	6.69400	-0.97600	0.62600
Br	8.11200	-3.42200	0.95200
H	3.41300	-2.67400	-0.52200
H	5.37900	-4.05000	0.01900
H	7.61200	-0.51300	0.97000
H	5.65200	0.87500	0.41800
C	2.40900	-0.61000	-2.24500
C	1.32000	0.12200	-2.72100

C	-0.03100	-0.11000	-2.37100
O	-0.95800	0.70100	-2.44500
N	-0.38600	-1.43300	-1.89400
N	-1.38000	-1.54900	-0.99500
C	-1.52600	-2.85000	-0.79900
C	-0.63400	-3.59800	-1.59700
C	0.07600	-2.64900	-2.29100
H	1.49000	1.07300	-3.21600
H	-2.27700	-3.20900	-0.10700
H	-0.54100	-4.67000	-1.66800
H	0.82900	-2.73400	-3.05800
H	3.38000	-0.46200	-2.71000
H	2.25200	-1.60600	-1.84900
C	3.42100	1.47100	-0.85400
C	4.76400	2.85700	-2.22500
C	5.54800	3.68300	-1.22400
H	5.34800	2.65700	-3.12400
H	3.82900	3.34800	-2.50300
H	5.81900	4.64600	-1.66400
H	4.94700	3.86800	-0.33100
H	6.46600	3.16500	-0.93800
O	4.46700	1.55400	-1.69700
S	1.86600	-0.22300	0.62400
C	2.54300	0.10500	2.32700
O	1.53000	-1.64100	0.62200
F	3.33900	1.17600	2.28900
F	3.23200	-0.93000	2.77100
F	1.53200	0.35500	3.15700

TS4

C	2.91700	1.94400	-0.33200
S	3.98400	2.39100	-1.57000
N	1.88200	2.71700	0.08100
N	3.02600	0.77600	0.35500
C	3.78100	-0.34900	-0.02700
C	3.75800	-0.83500	-1.33400
C	4.48600	-1.04700	0.95400
C	5.15700	-2.21800	0.62000
C	4.44200	-2.00400	-1.64700
C	5.14800	-2.71100	-0.68000
C	4.31800	-2.55500	-3.03800
C	5.86100	-2.99300	1.69600

F	5.31400	-3.39900	-3.33500
F	4.31100	-1.58700	-3.96200
F	3.16700	-3.23800	-3.18500
F	5.03500	-3.88800	2.27600
F	6.31400	-2.19700	2.67200
F	6.90000	-3.68600	1.21400
C	1.78800	4.13500	-0.21100
C	2.33200	4.97700	0.95100
C	0.34800	4.55600	-0.53300
C	2.27900	6.46700	0.62200
C	0.86200	6.88600	0.23700
C	0.31700	6.03600	-0.91700
N	-0.26500	3.65500	-1.59200
C	0.62000	3.34200	-2.75000
C	-1.57200	4.17100	-2.09400
H	4.49700	-0.67500	1.97200
H	3.21100	-0.29800	-2.10100
H	5.68400	-3.61700	-0.93500
H	2.50300	0.67000	1.23000
H	1.36200	2.37800	0.89900
H	2.41500	4.32200	-1.08800
H	3.35800	4.65100	1.14300
H	1.74500	4.76000	1.85300
H	2.96200	6.67700	-0.21100
H	2.63000	7.05400	1.47600
H	-0.29600	4.39200	0.34000
H	0.83400	7.94100	-0.05000
H	-0.70200	6.35700	-1.14600
H	0.92800	6.20200	-1.81500
H	0.90900	4.27400	-3.23900
H	1.50400	2.80200	-2.41700
H	0.04600	2.72500	-3.44100
H	-2.09400	3.34900	-2.58300
H	-2.16900	4.51700	-1.25400
H	-1.38100	4.97700	-2.80200
H	0.19900	6.77300	1.10400
H	-0.46100	2.75400	-1.11100
S	-3.19100	1.82500	-0.09500
C	-2.65400	0.15400	0.09800
O	-0.85100	-1.24700	-0.34800
C	-1.27700	0.01100	-0.41900
O	-0.54500	0.91500	-0.80200
C	0.50700	-1.48600	-0.77500

C	0.77600	-2.96000	-0.59100
O	-3.83300	2.10900	-1.38200
C	-4.55300	2.05800	1.17000
F	-5.33700	3.02400	0.71800
F	-5.27300	0.96000	1.36300
F	-4.02300	2.43300	2.32600
O	-2.17000	2.76600	0.37700
C	-4.83900	-0.70600	-0.92400
C	-3.65500	-0.92800	-0.20100
C	-3.42800	-2.23200	0.27200
C	-4.33700	-3.25900	0.04800
C	-5.50700	-2.99500	-0.65100
C	-5.76100	-1.72400	-1.14300
Br	-6.76600	-4.38000	-0.93300
H	1.16700	-0.86900	-0.16200
H	0.60400	-1.17300	-1.82000
H	0.63500	-3.24200	0.45600
H	0.10900	-3.55900	-1.21600
H	1.80900	-3.17800	-0.87600
H	-2.51400	-2.47300	0.79900
H	-4.13700	-4.25700	0.42200
H	-6.66800	-1.52400	-1.70000
H	-5.05400	0.26800	-1.34400
C	-2.22300	0.03600	2.13100
C	-1.22400	0.91000	2.56300
C	0.15800	0.61500	2.55000
O	1.08700	1.43100	2.46700
N	0.55300	-0.76800	2.72200
N	1.75600	-1.15900	2.26000
C	1.90500	-2.40500	2.67900
C	0.79600	-2.83100	3.44700
C	-0.04600	-1.74700	3.46000
H	-1.45300	1.95900	2.70500
H	2.80200	-2.95400	2.42300
H	0.65000	-3.78100	3.93700
H	-0.98300	-1.56900	3.96300
H	-3.25300	0.21100	2.42500
H	-1.98500	-1.01600	2.03000

TS5

C	-4.22500	0.58100	-0.05400
S	-4.77100	1.26700	-1.49000

N	-4.43800	-0.71100	0.28500
N	-3.51200	1.25000	0.90800
C	-2.82500	2.47100	0.75700
C	-1.50800	2.51700	1.21100
C	-3.41900	3.62200	0.23000
C	-2.66400	4.78200	0.12900
C	-0.77600	3.69400	1.09900
C	-1.33700	4.83700	0.55000
C	0.67600	3.66600	1.48300
C	-3.27300	6.03600	-0.43600
F	1.42900	3.14600	0.49600
F	1.15100	4.88900	1.74200
F	0.88700	2.90400	2.56700
F	-3.26000	7.03100	0.46500
F	-4.54100	5.85800	-0.82100
F	-2.58200	6.47400	-1.50000
C	-5.04100	-1.70900	-0.57600
C	-6.42800	-2.14200	-0.09400
C	-4.12400	-2.93900	-0.66100
C	-7.01800	-3.19700	-1.03100
C	-6.07700	-4.39400	-1.18300
C	-4.68300	-3.95600	-1.65000
N	-2.70300	-2.52500	-0.94100
C	-2.49800	-1.84900	-2.25500
C	-1.72600	-3.63700	-0.75200
H	-4.44900	3.60600	-0.09700
H	-1.04500	1.62500	1.62500
H	-0.76100	5.75100	0.45900
H	-3.02200	0.62200	1.53800
H	-4.02000	-1.06800	1.14800
H	-5.14800	-1.25600	-1.56600
H	-7.06600	-1.25500	-0.04500
H	-6.34400	-2.54300	0.92600
H	-7.19000	-2.74400	-2.01600
H	-7.99300	-3.52600	-0.66000
H	-4.07200	-3.40100	0.33200
H	-6.48800	-5.11700	-1.89400
H	-4.02400	-4.82600	-1.72400
H	-4.76000	-3.50800	-2.64800
H	-2.74000	-2.54800	-3.05600
H	-3.13200	-0.96200	-2.30700
H	-1.44500	-1.56500	-2.30400
H	-1.92000	-4.11100	0.21300

H	-0.72100	-3.20800	-0.76600
H	-1.84300	-4.35900	-1.56100
H	-5.98500	-4.91200	-0.22000
H	-2.43900	-1.81500	-0.22300
S	2.43400	-2.65600	0.49400
C	2.45600	-1.07300	-0.21900
O	1.57400	0.39600	-1.78300
C	1.47500	-0.85500	-1.28400
O	0.60900	-1.63300	-1.65400
C	0.55200	0.80700	-2.70500
C	-0.62400	1.39500	-1.94800
O	1.09000	-3.23200	0.57100
C	3.33000	-3.76400	-0.69000
F	2.75400	-3.70600	-1.88700
F	4.59600	-3.38200	-0.80200
F	3.28600	-5.01500	-0.24600
O	3.26100	-2.67500	1.70000
C	4.52800	-0.16300	0.90500
C	3.79400	-0.41500	-0.26300
C	4.37700	-0.05900	-1.48900
C	5.62500	0.54400	-1.55000
C	6.32200	0.78100	-0.37200
C	5.78700	0.42700	0.85800
Br	8.03700	1.58600	-0.45700
H	0.26000	-0.04600	-3.32100
H	1.03300	1.55500	-3.33800
H	-0.29900	2.27100	-1.37900
H	-1.41600	1.70500	-2.63600
H	-1.03800	0.66400	-1.24500
H	3.84500	-0.25400	-2.41300
H	6.06100	0.81500	-2.50500
H	6.34200	0.61100	1.77000
H	4.12600	-0.46300	1.86600
C	-0.79600	-1.17600	1.64600
O	-1.43800	-0.66200	0.71500
N	-1.42300	-2.27900	2.31100
C	-0.84000	-3.31900	2.96800
C	-1.85500	-4.15000	3.37700
H	0.23400	-3.40200	3.02900
C	-3.03400	-3.52200	2.91400
H	-1.76400	-5.07800	3.91800
H	-4.06100	-3.84200	3.03400
N	-2.76900	-2.40000	2.26100

C	0.47000	-0.77200	2.13300
H	0.87700	-1.24500	3.01700
C	1.24700	0.05800	1.35400
H	2.17000	0.47300	1.74200
H	0.78800	0.61800	0.54400

TS6

C	3.37500	0.99100	-0.27900
S	4.63600	0.84400	-1.39800
N	2.63400	2.11200	-0.13600
N	2.96300	-0.00400	0.55700
C	3.30600	-1.36200	0.53600
C	2.27000	-2.27200	0.76200
C	4.61000	-1.83900	0.38100
C	4.84200	-3.21000	0.42100
C	2.52800	-3.63500	0.80900
C	3.81600	-4.12500	0.63100
C	1.37400	-4.57600	1.00300
C	6.24400	-3.70400	0.20300
F	0.48400	-4.09000	1.88200
F	0.70400	-4.78700	-0.14400
F	1.77400	-5.77500	1.44800
F	7.14700	-2.91200	0.80000
F	6.55900	-3.74000	-1.10100
F	6.41600	-4.94500	0.68300
C	2.83900	3.34300	-0.86500
C	3.52300	4.41000	-0.00500
C	1.49100	3.87300	-1.37500
C	3.72800	5.69900	-0.80000
C	2.39800	6.20400	-1.35900
C	1.69900	5.13500	-2.20900
N	0.72800	2.78500	-2.11200
C	1.50800	2.00800	-3.11800
C	-0.52300	3.29900	-2.74100
H	5.42900	-1.14600	0.24000
H	1.25700	-1.90400	0.88900
H	4.01700	-5.18800	0.67500
H	2.08200	0.19400	1.03500
H	1.96900	2.14500	0.64800
H	3.48800	3.11700	-1.71700
H	4.47700	4.00100	0.34000
H	2.90000	4.59600	0.88000

H	4.42200	5.50800	-1.62900
H	4.18900	6.46400	-0.16900
H	0.85100	4.09500	-0.50900
H	2.54700	7.10400	-1.96300
H	0.74500	5.52800	-2.56800
H	2.31700	4.90400	-3.08700
H	1.88000	2.69000	-3.88400
H	2.33400	1.48800	-2.63500
H	0.82500	1.28000	-3.55800
H	-1.03200	3.95000	-2.03400
H	-1.15000	2.43900	-2.97600
H	-0.25800	3.83800	-3.65100
H	1.73600	6.48200	-0.52800
H	0.43900	2.11500	-1.37700
S	-0.99400	-0.29800	-1.20500
C	-2.23100	0.14300	-0.01100
O	-3.30400	2.07000	0.74500
C	-2.23700	1.61200	0.09400
O	-1.31800	2.33900	-0.25600
C	-3.29800	3.47500	1.07100
C	-3.88700	4.28300	-0.06700
O	0.34200	0.10600	-0.74900
C	-0.91000	-2.16700	-1.13500
F	-1.09400	-2.58300	0.11400
F	-1.79900	-2.73600	-1.92900
F	0.30600	-2.49600	-1.53600
O	-1.34900	0.03900	-2.59100
C	-4.38800	-0.63700	0.98400
C	-3.55500	-0.55100	-0.14300
C	-4.03900	-1.06500	-1.35400
C	-5.28200	-1.68600	-1.42800
C	-6.07100	-1.77000	-0.29100
C	-5.63900	-1.23700	0.91800
Br	-7.77200	-2.59500	-0.40200
H	-2.27500	3.78100	1.30000
H	-3.90800	3.54600	1.97400
H	-4.90200	3.94800	-0.29300
H	-3.92200	5.34100	0.20600
H	-3.27500	4.17600	-0.96600
H	-3.45700	-0.97200	-2.26300
H	-5.63900	-2.08300	-2.37100
H	-6.27200	-1.28900	1.79600
H	-4.07000	-0.20000	1.92300

C	-0.13700	1.73600	2.41900
O	0.92000	1.36900	1.89600
N	-0.19200	3.11900	2.83400
C	-0.96500	3.69600	3.79300
C	-0.72000	5.04800	3.74400
H	-1.59300	3.10200	4.43900
C	0.22500	5.19100	2.70200
H	-1.14300	5.81400	4.37400
H	0.68400	6.09700	2.32900
N	0.54200	4.02500	2.16000
C	-1.29400	0.95600	2.61900
H	-2.16600	1.38800	3.09400
C	-1.38900	-0.22500	1.87300
H	-2.13400	-0.97600	2.11100
H	-0.44500	-0.61800	1.50900

TS7

C	3.32800	1.51800	-0.40100
S	4.26800	1.90600	-1.75000
N	2.27900	2.28800	0.01500
N	3.49000	0.41500	0.37200
C	4.24500	-0.75300	0.21200
C	3.84500	-1.82300	1.02700
C	5.33000	-0.92800	-0.65200
C	5.96100	-2.16900	-0.70500
C	4.48900	-3.04700	0.94600
C	5.55700	-3.24300	0.07700
C	4.01400	-4.15600	1.84000
C	7.07900	-2.35800	-1.69300
F	4.41100	-3.98100	3.10900
F	2.67100	-4.22100	1.86300
F	4.46800	-5.35200	1.44200
F	6.61000	-2.67000	-2.91200
F	7.90300	-3.35100	-1.32600
F	7.81700	-1.24900	-1.83100
C	2.12200	3.67200	-0.40000
C	2.76500	4.63000	0.61000
C	0.65200	4.05400	-0.62200
C	2.66700	6.07900	0.13500
C	1.21600	6.45400	-0.15500
C	0.57300	5.48600	-1.15500
N	-0.05000	3.05300	-1.52200

C	0.73800	2.58900	-2.70000
C	-1.38200	3.53500	-1.98500
H	5.67400	-0.11300	-1.27200
H	3.00400	-1.68700	1.70300
H	6.05600	-4.20200	0.01300
H	2.81900	0.34000	1.14300
H	1.83500	2.06400	0.91200
H	2.65200	3.77700	-1.35200
H	3.81000	4.32800	0.73400
H	2.27000	4.50300	1.58200
H	3.26400	6.19900	-0.77800
H	3.09300	6.75200	0.88500
H	0.09800	3.97700	0.32100
H	1.14800	7.47300	-0.54700
H	-0.46600	5.78200	-1.31800
H	1.09500	5.55600	-2.12000
H	1.00800	3.45200	-3.31100
H	1.63300	2.06600	-2.37200
H	0.10300	1.91200	-3.27100
H	-1.23800	4.25900	-2.78600
H	-1.91900	3.97300	-1.14700
H	-1.92800	2.67000	-2.35600
H	0.63800	6.43300	0.77800
H	-0.24200	2.21100	-0.94400
S	-3.14900	1.34500	0.55400
C	-2.67300	-0.30000	0.21100
O	-1.23200	-1.75100	-0.86300
C	-1.45600	-0.46000	-0.59100
O	-0.65100	0.40800	-0.90400
C	-0.04500	-2.04800	-1.62600
C	1.19000	-2.08600	-0.74800
O	-2.03300	2.29000	0.50900
C	-4.24400	1.82300	-0.88000
F	-3.73500	1.32700	-2.01400
F	-5.46900	1.35700	-0.72000
F	-4.28600	3.14900	-0.98000
O	-4.04900	1.37700	1.70400
C	-4.79200	-1.44900	0.95500
C	-3.85700	-1.17600	-0.05300
C	-4.08700	-1.70600	-1.33000
C	-5.19400	-2.50100	-1.59200
C	-6.10100	-2.75700	-0.56900
C	-5.91400	-2.23400	0.70200

Br	-7.62200	-3.82500	-0.93600
H	0.06100	-1.29900	-2.41400
H	-0.25000	-3.02000	-2.07500
H	1.12100	-2.88100	-0.00000
H	2.07500	-2.27500	-1.36300
H	1.32200	-1.12800	-0.23800
H	-3.38900	-1.49800	-2.13400
H	-5.36000	-2.90900	-2.58200
H	-6.63400	-2.43400	1.48700
H	-4.66100	-1.02200	1.94200
C	-1.88900	-0.90100	2.09500
C	-0.50200	-0.89400	2.19500
C	0.33500	0.21900	2.42800
O	1.56500	0.22300	2.29700
N	-0.24400	1.47600	2.84000
N	0.32600	2.61200	2.39500
C	-0.35400	3.58800	2.97500
C	-1.36300	3.09000	3.82600
C	-1.25700	1.72600	3.71200
H	0.04600	-1.78300	1.90500
H	-0.09700	4.61900	2.76800
H	-2.05500	3.64500	4.43900
H	-1.77700	0.92600	4.21500
H	-2.50800	-0.17200	2.60400
H	-2.36100	-1.87200	1.97600

TS8

C	-3.40100	-1.28700	-0.31800
S	-4.53100	-1.24900	-1.57800
N	-2.56500	-2.33300	-0.09300
N	-3.20900	-0.27800	0.56700
C	-3.55300	1.07700	0.46700
C	-4.77400	1.56800	-0.00000
C	-2.58500	1.97300	0.93000
C	-2.82400	3.33800	0.90700
C	-4.98700	2.94400	-0.01700
C	-4.02800	3.84500	0.42900
C	-6.32800	3.45800	-0.46200
C	-1.72800	4.27200	1.33000
F	-7.21000	3.47500	0.55100
F	-6.24900	4.71200	-0.93100
F	-6.85900	2.69600	-1.42700

F	-2.21000	5.44100	1.77400
F	-0.89600	4.55600	0.31200
F	-0.97500	3.74500	2.30800
C	-2.61000	-3.58500	-0.81900
C	-3.22700	-4.70800	0.02300
C	-1.21200	-4.01300	-1.29600
C	-3.32400	-6.00300	-0.78300
C	-1.95700	-6.40400	-1.33500
C	-1.32200	-5.27300	-2.15500
N	-0.48800	-2.87600	-1.99600
C	-1.28000	-2.13400	-3.01900
C	0.80600	-3.31000	-2.59900
H	-1.63300	1.58200	1.27900
H	-5.53800	0.88700	-0.35000
H	-4.21300	4.91300	0.40600
H	-2.48100	-0.44900	1.26900
H	-1.98800	-2.32400	0.75300
H	-3.24900	-3.42000	-1.69200
H	-4.21400	-4.37400	0.35400
H	-2.61100	-4.85400	0.92100
H	-4.02300	-5.85600	-1.61600
H	-3.73300	-6.80500	-0.16200
H	-0.57900	-4.20800	-0.42200
H	-2.03700	-7.29900	-1.95700
H	-0.33800	-5.59300	-2.50500
H	-1.94100	-5.06900	-3.03800
H	-1.57200	-2.82800	-3.80900
H	-2.16200	-1.68700	-2.56500
H	-0.63300	-1.35200	-3.41800
H	0.59200	-3.83300	-3.53200
H	1.33100	-3.95300	-1.89700
H	1.39100	-2.41100	-2.78900
H	-1.28700	-6.65400	-0.50100
H	-0.26200	-2.20300	-1.24200
C	2.35200	-0.19300	0.24200
O	1.64500	-2.46100	-0.10600
C	4.53300	0.09900	-0.98200
C	3.57300	0.61100	-0.09500
C	3.78800	1.88700	0.43700
C	4.92400	2.62400	0.11300
C	5.86300	2.07500	-0.74900
C	5.67600	0.81500	-1.30600
Br	7.42100	3.05900	-1.17900

H	3.05700	2.33600	1.09800
H	5.07700	3.61200	0.53100
H	6.41100	0.40600	-1.99000
H	4.38200	-0.88000	-1.42700
C	1.87700	0.00300	2.31100
C	0.52600	-0.01200	2.64100
C	-0.31700	-1.14500	2.61700
O	-1.55000	-1.13600	2.56200
N	0.28200	-2.46500	2.64600
N	-0.31100	-3.45100	1.95100
C	0.38000	-4.54200	2.24400
C	1.41900	-4.27200	3.16400
C	1.32100	-2.92000	3.39600
H	-0.01400	0.92800	2.69000
H	0.10700	-5.48900	1.79600
H	2.12100	-4.96400	3.60400
H	1.85700	-2.26200	4.06200
H	2.51300	-0.85400	2.49500
H	2.40000	0.94600	2.42000
C	2.49100	-1.65800	0.26100
C	3.91400	-3.44700	0.89600
C	4.87900	-3.66300	2.04100
H	2.97400	-3.97700	1.05300
H	4.34300	-3.75700	-0.06300
H	5.13600	-4.72200	2.11500
H	5.79600	-3.09000	1.89400
H	4.41800	-3.35000	2.98200
O	3.64800	-2.03400	0.82500
S	0.96300	0.17100	-0.78200
O	1.22900	-0.06300	-2.20900
O	-0.28400	-0.39000	-0.23900
C	0.70400	2.00500	-0.64200
F	1.62500	2.68200	-1.30200
F	0.71000	2.36900	0.63900
F	-0.48500	2.25800	-1.16300

TS9

C	3.32200	-0.11300	-1.13800
S	4.95800	0.04500	-0.77700
N	2.72000	-1.30000	-1.39800

N	2.44200	0.92300	-1.24700
C	2.40700	2.20500	-0.69200
C	3.40200	2.80400	0.08400
C	1.21500	2.90500	-0.92600
C	1.00200	4.14800	-0.35800
C	3.16800	4.06900	0.62100
C	1.97700	4.75600	0.42600
H	0.44200	2.45400	-1.54200
H	4.33800	2.29400	0.26600
C	4.25500	4.66800	1.47000
H	1.81500	5.73200	0.86500
C	-0.34800	4.77600	-0.54800
H	1.56900	0.67300	-1.70600
F	3.99000	5.93800	1.80700
F	4.42200	3.98000	2.61000
F	5.43600	4.65700	0.83600
F	-1.28200	4.14100	0.18800
F	-0.75700	4.70200	-1.82300
F	-0.36600	6.06300	-0.18400
C	3.42100	-2.51900	-1.74400
H	1.70800	-1.30100	-1.54300
C	3.45400	-2.73900	-3.26100
C	2.76500	-3.71900	-1.05400
H	4.45100	-2.41200	-1.38900
C	4.20200	-4.02600	-3.60900
H	3.93300	-1.86700	-3.71700
H	2.42400	-2.78200	-3.63800
C	3.58300	-5.22200	-2.88800
H	5.25400	-3.92500	-3.31100
H	4.19200	-4.18600	-4.69000
C	3.53400	-5.00200	-1.37000
H	1.73000	-3.81100	-1.40700
N	2.61400	-3.46100	0.42700
H	4.14400	-6.13600	-3.10000
H	3.05900	-5.86500	-0.89700
H	4.55700	-4.92700	-0.98000
C	3.85000	-3.03200	1.14400
C	1.99200	-4.60800	1.13800
H	4.22100	-2.10300	0.71000
H	4.59700	-3.82500	1.07500
H	3.55900	-2.85100	2.17900
H	1.09900	-4.91500	0.59300
H	1.74100	-4.26500	2.14300

H	2.70900	-5.42700	1.19300
H	2.56200	-5.38100	-3.25700
C	-1.73100	-0.68700	-0.47100
H	1.96200	-2.65500	0.53400
O	-2.01800	-1.38000	-3.07600
C	-0.32600	0.10600	2.02400
H	-1.07500	0.35400	2.76400
O	1.57900	-1.21000	1.52400
C	0.70200	-0.83300	2.30400
N	0.71800	-1.42000	3.62200
C	0.04100	-1.04200	4.74400
C	0.36000	-1.93900	5.73000
H	-0.58300	-0.16200	4.76100
C	1.24800	-2.84400	5.10200
H	0.01500	-1.94100	6.75200
H	1.73400	-3.71200	5.52600
N	1.46300	-2.52500	3.83700
C	-3.19000	-0.46800	-0.17700
C	-4.13000	-1.42200	-0.59500
C	-3.65200	0.67400	0.48100
C	-5.48700	-1.24700	-0.36400
H	-3.78700	-2.30900	-1.12000
C	-5.01000	0.86400	0.72200
H	-2.95800	1.43700	0.81200
C	-5.91400	-0.09900	0.29600
H	-6.20800	-1.98700	-0.69400
H	-5.36100	1.75300	1.23200
Br	-7.76300	0.15500	0.61500
C	-0.42700	0.56600	0.72000
H	0.41800	0.35700	0.07200
H	-0.98400	1.46600	0.49200
O	-0.13500	-2.48900	-0.47700
C	-1.13000	-1.94000	-0.00900
C	-1.18600	-3.47200	1.80000
C	-1.58000	-4.78700	1.15500
H	-0.10500	-3.33700	1.83900
H	-1.58200	-3.38600	2.81400
H	-1.16400	-5.62400	1.72200
H	-2.66700	-4.89000	1.13600
H	-1.20800	-4.83400	0.12800
O	-1.75600	-2.36300	1.09400
S	-1.36800	-0.44000	-2.16500
O	0.06900	-0.17500	-2.37700

C	-2.15500	1.20400	-2.54300
F	-1.91000	2.08200	-1.56700
F	-1.59800	1.64900	-3.65900
F	-3.45800	1.08700	-2.71200

TS10

C	3.06400	0.03200	-1.31800
S	4.60200	0.48700	-0.80300
N	2.67300	-1.24000	-1.54700
N	2.08200	0.93700	-1.60800
C	1.94700	2.22900	-1.05900
C	2.17600	2.45600	0.30300
C	1.45600	3.25500	-1.85900
C	1.16400	4.49500	-1.29000
C	1.89700	3.70100	0.84000
C	1.38200	4.73600	0.05800
H	1.30300	3.09100	-2.92100
H	2.51600	1.63800	0.93000
C	2.15900	3.92900	2.30300
H	1.14500	5.69800	0.49500
C	0.56200	5.54800	-2.17300
H	1.27000	0.57500	-2.10600
F	3.40800	4.36800	2.52000
F	1.32800	4.84700	2.81700
F	2.01100	2.80100	3.01400
F	1.25300	5.68700	-3.31500
F	0.51200	6.74400	-1.57800
F	-0.69700	5.22700	-2.52800
C	3.52200	-2.41300	-1.50700
H	1.67800	-1.40100	-1.70700
C	3.48100	-3.15600	-2.84900
C	3.09400	-3.38000	-0.38700
H	4.54200	-2.05900	-1.32500
C	4.36200	-4.40500	-2.82700
H	3.79900	-2.46300	-3.63200
H	2.44000	-3.43500	-3.06300
C	3.97200	-5.33300	-1.67600
H	5.41100	-4.10500	-2.71000
H	4.28600	-4.93000	-3.78300
C	4.02500	-4.59000	-0.33700
H	2.07100	-3.70800	-0.59900
N	2.98900	-2.66800	0.93800
H	4.63700	-6.20000	-1.63800

H	3.74100	-5.26300	0.47800
H	5.05400	-4.26000	-0.15400
C	4.29300	-2.25500	1.52600
C	2.20100	-3.43400	1.94600
H	4.86100	-1.68600	0.79100
H	4.83400	-3.14100	1.85800
H	4.06400	-1.61000	2.37200
H	1.23000	-3.67900	1.51900
H	2.06700	-2.78900	2.81500
H	2.75000	-4.33200	2.22800
H	2.95600	-5.71500	-1.83800
C	-1.76100	-0.66800	-0.47200
H	2.44200	-1.79200	0.79500
O	-0.34100	-0.54900	-2.38900
C	-0.42300	0.25600	2.06800
H	-1.14900	0.39600	2.85500
O	1.87600	-0.30200	1.69400
C	0.87100	-0.16400	2.42100
N	1.09800	-0.38600	3.82300
C	2.32000	-0.35900	4.42700
C	2.10600	-0.60200	5.76100
H	3.20400	-0.12200	3.85700
C	0.70200	-0.76200	5.86600
H	2.84200	-0.64200	6.54700
H	0.11400	-0.97300	6.74900
N	0.10100	-0.62500	4.69700
C	-3.20400	-0.57700	-0.10700
C	-4.19400	-0.41100	-1.08500
C	-3.59900	-0.67900	1.23200
C	-5.53600	-0.31500	-0.73700
H	-3.91500	-0.35400	-2.13200
C	-4.93900	-0.59600	1.59200
H	-2.85000	-0.84500	1.99900
C	-5.89500	-0.40600	0.60200
H	-6.29600	-0.18200	-1.49800
H	-5.23700	-0.67600	2.63100
Br	-7.72300	-0.28000	1.08400
C	-0.68400	0.62000	0.74500
H	0.18800	0.64400	0.09500
H	-1.44600	1.36700	0.54200
C	-1.27000	-0.09300	-1.72000
C	-1.49300	1.71700	-3.23700
C	-2.35300	1.17800	-4.36200

H	-1.65500	2.78100	-3.05800
H	-0.43400	1.54500	-3.43900
H	-2.10300	1.67900	-5.30000
H	-2.17900	0.10500	-4.48600
H	-3.41100	1.34700	-4.15000
O	-1.85100	1.07800	-1.99700
C	-1.28700	-3.27000	-1.70900
F	-2.21900	-2.73000	-2.48500
F	-0.15100	-3.38000	-2.38900
F	-1.67800	-4.47900	-1.33500
S	-1.02500	-2.22600	-0.16300
O	0.43500	-2.10900	-0.08200
O	-1.73100	-2.92700	0.90000

TS11

C	-2.78700	1.34200	-1.06300
S	-4.26700	2.07500	-0.71000
N	-1.66400	2.02000	-1.38100
N	-2.60800	-0.00700	-1.08300
C	-3.41500	-0.96600	-0.43600
C	-3.82100	-0.77900	0.89000
C	-3.72500	-2.14600	-1.10300
C	-4.45700	-3.13400	-0.44700
C	-4.56700	-1.76700	1.51100
C	-4.89400	-2.95400	0.85500
H	-3.40300	-2.28800	-2.13000
H	-3.52000	0.12200	1.41700
C	-5.05900	-1.55900	2.91600
H	-5.46700	-3.72500	1.35800
C	-4.73800	-4.41500	-1.17900
H	-1.76000	-0.34700	-1.53200
F	-5.05900	-2.70700	3.61100
F	-4.30300	-0.67900	3.58600
F	-6.31800	-1.09400	2.93600
F	-5.17700	-4.18200	-2.42500
F	-5.65900	-5.16100	-0.55500
F	-3.63100	-5.16600	-1.29600
C	-1.61700	3.39100	-1.84700
H	-0.78100	1.50400	-1.42300
C	-0.90700	3.46400	-3.20500
C	-0.85600	4.27800	-0.85400
H	-2.65100	3.74000	-1.94700

C	-0.79800	4.90500	-3.70500
H	-1.45500	2.83900	-3.91600
H	0.09400	3.02300	-3.09000
C	-0.10000	5.79700	-2.67700
H	-1.80400	5.29800	-3.90000
H	-0.25800	4.92700	-4.65500
C	-0.81300	5.72900	-1.32200
H	0.16100	3.87700	-0.77800
N	-1.42300	4.11800	0.53800
H	-0.07200	6.83400	-3.02300
H	-0.29800	6.36200	-0.59200
H	-1.83300	6.11700	-1.43600
C	-2.68700	4.86500	0.79200
C	-0.40200	4.44100	1.57000
H	-3.39600	4.65200	-0.00600
H	-2.46200	5.92900	0.86100
H	-3.09700	4.50200	1.73400
H	0.45800	3.78700	1.41800
H	-0.83500	4.25800	2.55200
H	-0.11500	5.48900	1.47000
H	0.94100	5.47000	-2.55500
C	2.16100	1.22900	-0.56900
C	2.26300	-0.24500	-0.66000
O	1.12500	1.87400	-0.66500
C	0.73900	-2.53600	-1.64600
H	-1.63500	3.10700	0.66800
O	0.09600	-0.09300	-2.22900
C	-0.16400	-0.17100	1.12500
H	-1.01300	-0.57300	0.59000
O	-1.54500	1.60500	1.85300
C	-0.44700	1.02600	1.81600
N	0.57300	1.61300	2.66700
C	0.29900	2.22200	3.85100
C	1.48600	2.72500	4.32700
H	-0.70900	2.23400	4.23700
C	2.43400	2.37200	3.34100
H	1.65200	3.25400	5.25200
H	3.49500	2.58000	3.31700
N	1.87700	1.70000	2.34200
C	3.57600	-0.90600	-0.38200
C	4.17700	-1.88800	-1.18300
C	4.22200	-0.57400	0.82400
C	5.35800	-2.51500	-0.79700

H	3.74400	-2.17300	-2.13300
C	5.40400	-1.18600	1.21300
H	3.79400	0.19300	1.46300
C	5.96300	-2.16400	0.39900
H	5.80300	-3.27200	-1.43200
H	5.88400	-0.91300	2.14600
Br	7.56200	-3.03000	0.93400
O	3.34200	1.82100	-0.34900
C	3.31300	3.24200	-0.18900
C	4.73100	3.68200	0.09900
H	2.63400	3.48800	0.63400
H	2.92100	3.69400	-1.10700
H	4.76900	4.76600	0.22600
H	5.09900	3.21000	1.01400
H	5.39500	3.40300	-0.72200
F	-0.40700	-2.44900	-0.97000
F	0.51000	-3.15700	-2.79100
F	1.60900	-3.23900	-0.93100
S	1.36000	-0.82200	-2.05300
O	2.19100	-1.02600	-3.23700
C	1.05900	-0.81300	1.06400
H	1.09000	-1.85900	0.78400
H	1.85800	-0.50900	1.72700

TS12

C	-2.90000	1.25200	-0.87400
S	-4.47800	1.75900	-0.56000
N	-1.87600	2.08800	-1.17000
N	-2.52000	-0.04800	-0.91000
C	-3.24300	-1.16600	-0.46000
C	-3.84800	-1.20000	0.80200
C	-3.23700	-2.30000	-1.26300
C	-3.80600	-3.48500	-0.79600
C	-4.45000	-2.37500	1.22100
C	-4.42800	-3.53300	0.43900
H	-2.78000	-2.25800	-2.24800
H	-3.80500	-0.32100	1.43600
C	-5.10500	-2.45900	2.57100
H	-4.87900	-4.45200	0.79700
C	-3.67400	-4.70600	-1.65700
H	-1.57500	-0.25100	-1.23500
F	-4.48800	-3.35900	3.35500

F	-5.09700	-1.28900	3.21600
F	-6.38300	-2.85800	2.46500
F	-4.12400	-4.48900	-2.90100
F	-4.33100	-5.75700	-1.16000
F	-2.37800	-5.06300	-1.78100
C	-2.02100	3.49800	-1.45900
H	-0.95000	1.68000	-1.30200
C	-1.85500	3.80000	-2.95300
C	-1.00600	4.31700	-0.65300
H	-3.03700	3.77400	-1.16400
C	-2.02000	5.29500	-3.23000
H	-2.59800	3.21200	-3.50000
H	-0.86200	3.46300	-3.27800
C	-1.05000	6.11800	-2.38300
H	-3.05000	5.59400	-2.99700
H	-1.86200	5.49800	-4.29300
C	-1.22100	5.81100	-0.89000
H	0.00400	4.03100	-0.97000
N	-1.05000	3.92200	0.80700
H	-1.20300	7.18700	-2.55100
H	-0.51500	6.40900	-0.30700
H	-2.23500	6.09300	-0.58100
C	-2.32800	4.23800	1.50600
C	0.12700	4.45200	1.55300
H	-3.15600	3.77500	0.96700
H	-2.44200	5.31900	1.57400
H	-2.26600	3.78800	2.49600
H	1.03200	4.04800	1.10100
H	0.05600	4.12200	2.58800
H	0.11000	5.54000	1.51300
H	-0.01800	5.89200	-2.68000
C	2.14400	-0.38700	-0.13200
H	-0.98500	2.88900	0.82900
O	0.25100	-0.46600	-1.56800
C	-0.19300	-0.43400	1.57400
H	-0.94600	-0.98500	1.02000
O	-1.78800	1.27300	1.97400
C	-0.65600	0.77900	2.10400
N	0.22600	1.55300	2.98100
C	-0.22700	2.18100	4.10000
C	0.86000	2.79000	4.68500
H	-1.27000	2.12200	4.37200
C	1.93800	2.47400	3.83000

H	0.87700	3.36900	5.59500
H	2.97600	2.76500	3.91100
N	1.54900	1.72200	2.80800
C	3.52800	-0.91900	0.03400
C	4.11400	-1.76200	-0.91800
C	4.27600	-0.56700	1.16600
C	5.39400	-2.27000	-0.73200
H	3.56600	-2.03400	-1.81200
C	5.56200	-1.05900	1.35700
H	3.85600	0.12300	1.89200
C	6.10500	-1.91600	0.40800
H	5.83800	-2.92900	-1.46900
H	6.13500	-0.78200	2.23400
Br	7.84600	-2.61600	0.66300
C	1.14100	-0.86400	1.55800
H	1.32700	-1.91900	1.36500
H	1.84200	-0.41300	2.24800
C	1.17400	-1.05000	-1.00800
C	0.22300	-3.14900	-1.57700
C	0.25000	-3.15900	-3.09100
H	0.37000	-4.14800	-1.16700
H	-0.71900	-2.75100	-1.19100
H	-0.54600	-3.81400	-3.45700
H	0.09400	-2.15600	-3.49100
H	1.20600	-3.54400	-3.45500
O	1.30400	-2.37800	-1.01300
C	2.23100	1.62400	-2.24300
F	2.72400	0.54400	-2.83800
F	1.04200	1.91200	-2.76300
F	3.04400	2.64800	-2.46000
S	2.09500	1.36900	-0.37900
O	0.78200	1.95700	-0.08200
O	3.29300	2.00400	0.15000

TS13

C	2.66400	1.37100	1.29200
S	4.18800	2.03800	1.58000
N	1.55300	2.10900	1.07800
N	2.38500	0.03600	1.26600
C	3.16700	-1.07700	0.95100
C	4.56100	-1.10900	0.85400

C	2.43700	-2.24000	0.66400
C	3.08500	-3.37900	0.21200
C	5.18000	-2.27200	0.40600
C	4.46700	-3.41400	0.06500
H	1.35300	-2.23500	0.76400
H	5.14600	-0.23400	1.10000
C	6.66400	-2.23700	0.18000
H	4.96900	-4.30400	-0.29500
C	2.23700	-4.54000	-0.21600
H	1.39000	-0.18200	1.27600
F	7.29800	-1.49600	1.09700
F	7.20700	-3.46200	0.19800
F	6.95700	-1.69900	-1.02100
F	1.58300	-4.25100	-1.36500
F	1.30000	-4.84200	0.68800
F	2.95900	-5.64200	-0.44600
C	1.44800	3.55200	1.18400
H	0.66400	1.62900	0.91400
C	0.48500	3.92900	2.31800
C	0.94200	4.10700	-0.15500
H	2.44400	3.94300	1.41000
C	0.22600	5.43400	2.36500
H	0.92000	3.57200	3.25600
H	-0.45700	3.39100	2.16300
C	-0.29800	5.93000	1.01700
H	1.15400	5.96600	2.61100
H	-0.49100	5.66100	3.15900
C	0.70200	5.61200	-0.09900
H	0.00500	3.58300	-0.37600
N	1.85800	3.70300	-1.28600
H	-0.48700	7.00600	1.04300
H	0.32200	5.97500	-1.06000
H	1.64300	6.13800	0.10600
C	3.24500	4.23300	-1.21100
C	1.26700	3.99300	-2.62000
H	3.71700	3.89000	-0.29000
H	3.22400	5.32100	-1.27000
H	3.79200	3.82700	-2.06300
H	0.21700	3.70900	-2.60700
H	1.79500	3.38100	-3.35300
H	1.39000	5.05100	-2.85000
H	-1.25400	5.43500	0.79600
C	-2.54600	-0.01900	-0.05100

H	1.95000	2.66200	-1.25800
O	-0.40500	-0.10800	1.50500
C	-0.08300	-0.94000	-1.64800
H	0.37900	-1.88700	-1.39800
O	0.28600	1.39800	-1.78200
C	0.69700	0.23200	-1.79800
N	2.11300	0.04200	-1.97800
C	2.79100	-1.07500	-2.37000
C	4.12100	-0.74100	-2.42000
H	2.27800	-2.00300	-2.57000
C	4.15800	0.61700	-2.02900
H	4.95300	-1.38200	-2.66700
H	5.01800	1.26200	-1.90800
N	2.94600	1.08400	-1.78000
C	-3.94900	-0.50900	-0.21300
C	-4.23900	-1.86600	-0.42000
C	-5.03200	0.38100	-0.11500
C	-5.55000	-2.31500	-0.54000
H	-3.43600	-2.59300	-0.45400
C	-6.34400	-0.05300	-0.24900
H	-4.84900	1.43400	0.06900
C	-6.59300	-1.40400	-0.46200
H	-5.75600	-3.36800	-0.69400
H	-7.16600	0.64900	-0.17200
Br	-8.37800	-2.01400	-0.62700
C	-1.46000	-0.81700	-1.74800
H	-1.86700	0.02500	-2.30000
H	-2.05800	-1.72100	-1.75800
O	-1.59500	2.15700	0.46500
C	-2.27400	1.42400	-0.23000
C	-2.62100	3.16300	-1.84200
C	-2.35500	3.06100	-3.33000
H	-3.50200	3.77600	-1.62600
H	-1.77000	3.58400	-1.30400
H	-2.16800	4.05300	-3.75200
H	-1.48100	2.42600	-3.49900
H	-3.21200	2.62200	-3.84600
O	-2.87800	1.84100	-1.36100
S	-1.68800	-0.77500	1.24800
C	-2.66400	-0.45300	2.79400
F	-3.75800	-1.20300	2.80400
F	-1.92200	-0.74000	3.85400
F	-3.01100	0.82900	2.83000

O	-1.68900	-2.22700	1.09400

TS14

C	-2.65200	1.35900	-0.87400
S	-4.15100	1.96700	-1.36800
N	-1.54500	2.11800	-0.74300
N	-2.41600	0.04900	-0.57500
C	-3.30000	-1.03900	-0.57800
C	-4.62500	-0.96800	-0.14000
C	-2.77600	-2.27600	-0.95700
C	-3.55200	-3.42600	-0.86200
C	-5.38500	-2.12900	-0.07100
C	-4.86700	-3.37200	-0.42100
H	-1.75600	-2.32900	-1.32800
H	-5.04400	-0.01900	0.16600
C	-6.78300	-2.00800	0.46400
H	-5.47100	-4.26900	-0.35000
C	-2.91400	-4.73500	-1.22200
H	-1.43500	-0.21800	-0.47900
F	-7.50800	-1.12500	-0.23300
F	-7.43500	-3.17700	0.44700
F	-6.77700	-1.57400	1.74000
F	-3.77500	-5.75600	-1.16900
F	-1.89700	-5.02600	-0.38500
F	-2.38800	-4.71000	-2.45700
C	-1.42800	3.50000	-1.18200
H	-0.65800	1.65900	-0.51800
C	-0.65700	3.60600	-2.50600
C	-0.75700	4.33900	-0.08700
H	-2.44300	3.86700	-1.35600
C	-0.48600	5.06400	-2.92900
H	-1.21200	3.03800	-3.25800
H	0.32500	3.13600	-2.38600
C	0.20800	5.86200	-1.82600
H	-1.46800	5.50900	-3.13900
H	0.09100	5.11500	-3.85700
C	-0.59300	5.79300	-0.52100
H	0.22200	3.89800	0.13400
N	-1.51800	4.20000	1.21100
H	0.33300	6.90800	-2.12000
H	-0.08300	6.36600	0.26000
H	-1.57600	6.25200	-0.68300

C	-2.95000	4.59100	1.16200
C	-0.82000	4.85400	2.34800
H	-3.48100	3.94100	0.46500
H	-3.03700	5.63700	0.86700
H	-3.36100	4.45300	2.16300
H	0.23600	4.59600	2.30200
H	-1.24800	4.45600	3.26900
H	-0.96800	5.93300	2.29700
H	1.21100	5.45000	-1.65500
C	2.58400	-0.15800	0.36600
H	-1.50600	3.18500	1.44900
O	0.41700	-0.61300	-0.56000
C	0.49600	-0.20100	2.46500
H	0.09300	-1.19900	2.56600
O	-0.00600	2.07500	2.05800
C	-0.35100	0.91900	2.31700
N	-1.77200	0.69400	2.48200
C	-2.44800	-0.44100	2.81600
C	-3.78600	-0.13500	2.79800
H	-1.93800	-1.36100	3.04800
C	-3.82700	1.23000	2.43000
H	-4.61600	-0.79700	2.99000
H	-4.69100	1.86500	2.28300
N	-2.61200	1.71900	2.25000
C	3.97500	-0.70900	0.45500
C	4.43900	-1.66600	-0.45700
C	4.86100	-0.23900	1.43300
C	5.73400	-2.16400	-0.38000
H	3.77800	-2.03000	-1.23700
C	6.16300	-0.72100	1.51500
H	4.53800	0.53000	2.12600
C	6.58400	-1.68600	0.61100
H	6.08400	-2.90700	-1.08700
H	6.84100	-0.35100	2.27500
Br	8.35000	-2.36000	0.72700
C	1.86800	-0.00600	2.33200
H	2.25000	1.00200	2.47800
H	2.53700	-0.80800	2.62900
C	1.48700	-0.99900	-0.11000
C	0.69100	-3.23100	-0.16800
C	0.70700	-3.51900	-1.65700
H	0.93100	-4.12200	0.41200
H	-0.27300	-2.84200	0.17100

H	-0.03500	-4.28100	-1.90400
H	0.48700	-2.61400	-2.22900
H	1.69200	-3.88900	-1.95500
O	1.71900	-2.28900	0.18000
C	3.47600	1.40100	-1.85400
F	3.01400	0.35600	-2.53700
F	3.21700	2.51300	-2.53300
F	4.78500	1.27900	-1.69800
S	2.60700	1.49300	-0.21300
O	1.29300	2.01200	-0.58700
O	3.47200	2.30200	0.63800

TS15

C	-2.73900	1.69100	-0.80000
S	-4.18700	2.45900	-1.21500
N	-1.57000	2.36600	-0.63500
N	-2.59300	0.35700	-0.59100
C	-3.48800	-0.72100	-0.61000
C	-4.87900	-0.64300	-0.69400
C	-2.88500	-1.98500	-0.48800
C	-3.66000	-3.13000	-0.44800
C	-5.63100	-1.81700	-0.66200
C	-5.04900	-3.06800	-0.53900
H	-1.80400	-2.05700	-0.39800
H	-5.37100	0.31500	-0.78700
C	-7.12400	-1.68000	-0.76500
H	-5.65200	-3.96800	-0.51800
C	-2.98900	-4.45500	-0.22100
H	-1.63200	0.05300	-0.43500
F	-7.61600	-0.92700	0.23200
F	-7.48600	-1.08300	-1.91000
F	-7.74600	-2.86600	-0.71600
F	-2.86800	-4.72700	1.09400
F	-1.75500	-4.48900	-0.73800
F	-3.68800	-5.46400	-0.75800
C	-1.38600	3.76500	-0.98300
H	-0.70600	1.83400	-0.49700
C	-0.66400	3.93100	-2.32500
C	-0.61300	4.49700	0.12200
H	-2.38300	4.20300	-1.07900
C	-0.49400	5.41100	-2.67100
H	-1.25200	3.41400	-3.08800

H	0.31300	3.43600	-2.26200
C	0.24400	6.14900	-1.55400
H	-1.48100	5.86800	-2.81600
H	0.04700	5.51500	-3.61600
C	-0.47000	5.98100	-0.20900
H	0.38000	4.03800	0.19700
N	-1.24600	4.25300	1.46800
H	0.33600	7.21400	-1.78600
H	0.09400	6.49500	0.57600
H	-1.46200	6.44900	-0.26700
C	-2.69800	4.56700	1.55700
C	-0.50900	4.90600	2.58000
H	-3.25500	3.94200	0.85800
H	-2.86100	5.62400	1.34100
H	-3.01900	4.34300	2.57500
H	0.56400	4.80300	2.41300
H	-0.78700	4.39900	3.50500
H	-0.77400	5.96300	2.63700
H	1.26400	5.75200	-1.46600
C	2.46700	1.61400	-0.27300
C	2.64200	0.21900	0.07400
O	1.38900	2.15400	-0.51200
C	1.70100	-2.47100	-0.06700
H	-1.18200	3.20500	1.68900
O	0.05700	-0.40800	-0.05700
C	0.97600	1.34700	2.34900
H	1.10900	2.42400	2.31000
O	-1.32300	1.83500	2.41600
C	-0.39800	0.99300	2.44600
N	-0.83900	-0.33900	2.62300
C	-2.15500	-0.71600	2.57100
C	-2.17600	-2.08000	2.67100
H	-2.92900	0.02500	2.45500
C	-0.80900	-2.44900	2.77100
H	-3.03900	-2.72600	2.65100
H	-0.38600	-3.44100	2.85800
N	-0.01300	-1.39900	2.74600
C	3.99500	-0.40600	-0.08800
C	4.64200	-0.35100	-1.33000
C	4.63300	-1.06000	0.96900
C	5.89400	-0.92100	-1.51500
H	4.14700	0.14100	-2.16300
C	5.89000	-1.63600	0.80000

H	4.15200	-1.12900	1.93800
C	6.50700	-1.55800	-0.44000
H	6.38900	-0.87800	-2.47800
H	6.38300	-2.14100	1.62300
Br	8.21800	-2.33300	-0.67600
O	3.62100	2.30600	-0.20000
C	3.53700	3.71800	-0.42600
C	3.21600	4.44000	0.86900
H	2.78100	3.91200	-1.19100
H	4.51800	4.00300	-0.80900
H	3.11100	5.51500	0.69600
H	2.27800	4.05500	1.27700
H	4.00300	4.27800	1.60900
F	0.56200	-3.14000	-0.20300
F	2.63100	-3.02700	-0.82700
F	2.08600	-2.52600	1.20100
S	1.36600	-0.73100	-0.64700
O	1.45300	-0.82400	-2.10300
C	2.08700	0.53900	2.19400
H	3.06500	0.99500	2.32600
H	2.01500	-0.52100	2.39400

TS16

C	2.62000	1.67600	0.89900
S	4.16600	2.32700	1.11100
N	1.49500	2.41600	0.77000
N	2.36300	0.34400	0.87200
C	3.25200	-0.73500	0.78900
C	4.42300	-0.72200	0.02600
C	2.85300	-1.91700	1.41000
C	3.58100	-3.08600	1.22500
C	5.14800	-1.89700	-0.11900
C	4.74000	-3.09500	0.46200
H	1.96300	-1.91600	2.03200
H	4.73400	0.18700	-0.47200
C	6.30600	-1.89500	-1.07200
H	5.30600	-4.00700	0.31600
C	3.02800	-4.34800	1.81700
H	1.37900	0.06400	0.90300
F	7.14000	-2.92200	-0.86000
F	5.87300	-2.00100	-2.34700
F	7.02000	-0.76500	-1.00300

F	1.89000	-4.71300	1.18900
F	2.70700	-4.19500	3.11200
F	3.87900	-5.37500	1.72500
C	1.43400	3.86100	0.89700
H	0.59600	1.92900	0.71100
C	0.59400	4.28200	2.11100
C	0.86500	4.48400	-0.38700
H	2.45900	4.21000	1.05200
C	0.48900	5.80400	2.21100
H	1.06500	3.86000	3.00300
H	-0.40600	3.84300	2.02300
C	-0.08700	6.39100	0.92200
H	1.48400	6.23200	2.39100
H	-0.13500	6.07700	3.06600
C	0.78100	6.00400	-0.28100
H	-0.13900	4.07000	-0.53900
N	1.65200	4.03200	-1.59600
H	-0.15300	7.48000	0.98700
H	0.36500	6.43000	-1.20000
H	1.78400	6.42500	-0.14300
C	3.06800	4.49300	-1.64800
C	0.96600	4.35800	-2.87600
H	3.58600	4.18300	-0.74000
H	3.09500	5.57500	-1.77400
H	3.53500	4.00600	-2.50400
H	-0.10300	4.16700	-2.77500
H	1.38400	3.71300	-3.65000
H	1.13300	5.40500	-3.12800
H	-1.10500	6.01200	0.77000
C	-2.47500	0.03500	-0.18000
O	-3.63600	2.33000	-0.69700
H	1.71900	2.97200	-1.58700
O	-0.38400	-0.13200	0.95300
C	-0.23300	1.04500	-1.98200
H	-0.32700	2.12400	-1.93400
O	2.07900	1.42900	-2.09200
C	1.12200	0.62900	-2.01800
N	1.48100	-0.74400	-2.06600
C	2.68800	-1.21400	-2.49800
C	2.65100	-2.57700	-2.36300
H	3.44600	-0.53400	-2.85200
C	1.37000	-2.84200	-1.81800
H	3.43800	-3.27500	-2.59800

H	0.94600	-3.79600	-1.53400
N	0.66700	-1.73600	-1.64400
C	-3.79400	-0.64100	-0.40500
C	-4.34700	-1.47700	0.57700
C	-4.54400	-0.40800	-1.56300
C	-5.58100	-2.08700	0.39800
H	-3.80200	-1.65300	1.49900
C	-5.78500	-1.00600	-1.75600
H	-4.17700	0.27000	-2.32400
C	-6.28900	-1.84700	-0.77400
H	-5.99500	-2.73200	1.16400
H	-6.35500	-0.81400	-2.65700
Br	-7.98000	-2.66500	-1.02100
C	-1.40700	0.32100	-2.09400
H	-2.27400	0.86500	-2.45200
H	-1.39600	-0.74800	-2.27200
C	-1.39400	-0.66500	0.50200
C	-0.55300	-2.80400	1.08700
C	-0.58000	-2.76800	2.60400
H	-0.76300	-3.80700	0.71200
H	0.40500	-2.47800	0.68400
H	0.18700	-3.44000	2.99900
H	-0.38700	-1.75700	2.97000
H	-1.55100	-3.09900	2.98100
O	-1.58600	-1.98800	0.51700
S	-2.66800	1.71700	0.21000
O	-1.40300	2.42000	0.44600
C	-3.47800	1.74000	1.88200
F	-2.87100	0.85700	2.67300
F	-3.36100	2.95100	2.41200
F	-4.76100	1.42300	1.78400

TS17

S	-3.82600	1.87900	0.08500
O	-4.19500	2.37500	1.41300
C	-5.44200	1.73400	-0.82400
F	-6.38000	1.16400	-0.08600
F	-5.29000	1.04300	-1.95500
F	-5.82100	2.96800	-1.12800
C	-4.25300	-0.80000	0.32800
C	-4.72600	-1.62700	-0.69300
C	-4.78100	-0.96400	1.61600

C	-5.69900	-2.59100	-0.44700
C	-5.75400	-1.92000	1.88000
C	-6.20300	-2.72500	0.83900
Br	-7.53400	-4.03200	1.17500
C	-0.02100	-0.82900	-1.82600
N	0.74600	-2.02700	-1.77800
C	0.32000	-3.32900	-1.80900
C	1.42400	-4.11400	-1.61800
C	2.49200	-3.19200	-1.46700
N	2.07600	-1.94400	-1.55800
C	-2.22300	0.12200	-2.03200
H	-4.33300	-1.52200	-1.69800
H	-4.42900	-0.31900	2.41600
H	-6.06300	-3.23000	-1.24400
H	-6.16400	-2.03800	2.87700
H	-0.71700	-3.58900	-1.93800
H	1.46500	-5.19100	-1.58500
H	3.54200	-3.39800	-1.30600
H	-1.78700	1.08100	-1.78100
H	-3.20800	0.14900	-2.48200
C	-1.40000	-0.96200	-2.17800
O	0.57500	0.22500	-1.56900
H	-1.78300	-1.91300	-2.52200
C	-3.21300	0.24500	0.06400
C	-1.94300	0.14900	0.75300
O	-1.13000	1.06400	0.88700
O	-3.05100	2.78300	-0.78100
O	-1.69900	-1.09700	1.19300
C	-0.60500	-1.26200	2.11500
C	0.66400	-1.69100	1.41000
H	-0.94900	-2.02700	2.81400
H	-0.45700	-0.32400	2.65400
H	1.46900	-1.82200	2.13800
H	0.52000	-2.63500	0.87800
H	0.97700	-0.93100	0.69200
N	0.39100	3.10700	-1.90400
C	-0.66100	3.28600	-2.94700
C	-0.07600	3.58600	-0.53700
C	1.04100	3.41600	0.49700
C	0.49500	3.72500	1.89400
C	-0.09300	5.13600	1.93700
C	-1.16900	5.31200	0.86500
C	-0.62300	5.00700	-0.53700

N	1.59100	2.07500	0.38800
C	2.90900	1.78500	0.38300
N	3.17200	0.49000	0.02600
C	4.41800	-0.15000	0.14400
S	4.10700	2.92800	0.71300
C	1.68600	3.68200	-2.36600
C	4.90000	-0.90100	-0.92600
C	6.09600	-1.60300	-0.79600
C	6.84200	-1.54600	0.37100
C	6.35400	-0.78000	1.42900
C	5.15200	-0.09700	1.33400
C	6.54400	-2.47300	-1.93400
C	7.12900	-0.76200	2.71600
F	5.90800	-3.66200	-1.91700
F	6.27800	-1.91600	-3.12300
F	7.85500	-2.73400	-1.88600
F	6.93000	-1.88800	3.42200
F	8.44900	-0.67700	2.49200
F	6.78100	0.26300	3.50200
H	1.85800	4.11400	0.28800
H	-0.27500	2.98400	2.13900
H	1.31700	3.61500	2.60800
H	-0.51200	5.33500	2.92700
H	0.70800	5.87000	1.77600
H	-2.00600	4.63000	1.06300
H	-1.56900	6.33000	0.88200
H	0.16200	5.72900	-0.79600
H	-1.43400	5.10200	-1.26400
H	-0.89100	2.90100	-0.28800
H	2.48400	0.01500	-0.56600
H	0.90800	1.31600	0.39600
H	1.89400	3.28700	-3.36000
H	1.59000	4.76700	-2.41200
H	2.48800	3.40400	-1.68300
H	-0.43900	2.60900	-3.77300
H	-1.63500	3.06100	-2.51200
H	-0.64000	4.31800	-3.29600
H	0.52100	2.08300	-1.79100
H	7.78400	-2.07500	0.45800
H	4.78400	0.48400	2.17100
H	4.33500	-0.93700	-1.85100

TS18

C	-3.98600	-1.29900	1.74800
O	-4.57500	-0.77700	2.84500
C	-4.58100	-1.60600	4.01700
C	-5.05300	0.68100	0.52600
C	-4.83000	1.97100	0.03000
C	-6.36700	0.31800	0.85600
C	-5.87500	2.87500	-0.12300
C	-7.42000	1.21300	0.71700
C	-7.16200	2.48800	0.22700
Br	-8.58700	3.71900	0.02500
C	-0.22100	0.75000	0.15800
N	0.36100	1.30900	-1.03800
C	-0.29000	1.89600	-2.07900
C	0.65700	2.19000	-3.03100
C	1.86900	1.72800	-2.47600
N	1.68200	1.19200	-1.27800
C	-2.23900	0.76400	1.51300
C	-3.24800	-1.53000	4.73600
H	-3.82800	2.26400	-0.25900
H	-6.56900	-0.68000	1.23100
H	-5.69300	3.87100	-0.50900
H	-8.43200	0.92200	0.97500
H	-1.36600	1.98700	-2.07200
H	0.49800	2.65000	-3.99300
H	2.86500	1.76100	-2.89900
H	-1.83800	-0.11200	2.02200
H	-2.96800	1.34200	2.07400
H	-5.38900	-1.20600	4.63100
H	-4.81800	-2.63200	3.72900
H	-3.29100	-2.09200	5.67300
H	-2.46100	-1.95700	4.11100
H	-2.99800	-0.49100	4.96500
C	-1.40400	1.38800	0.59700
O	0.37500	-0.20300	0.67300
H	-1.67200	2.33800	0.15400
C	-3.92900	-0.29000	0.68100
O	-2.03400	-1.63500	-0.60600
O	-3.49000	-2.41200	1.74100
N	0.82100	-2.87200	1.16000
C	-0.46900	-2.93500	1.90500
C	0.62600	-3.10500	-0.32500
C	1.96100	-2.96300	-1.05900

C	1.71500	-3.02900	-2.57000
C	0.99800	-4.32600	-2.94700
C	-0.31200	-4.47700	-2.17100
C	-0.07800	-4.41400	-0.65600
N	2.56100	-1.70500	-0.65600
C	3.87700	-1.47200	-0.44900
N	4.10700	-0.14900	-0.20600
C	5.28900	0.56300	0.03200
S	5.03600	-2.69700	-0.48100
C	1.87200	-3.71100	1.79500
C	5.22800	1.94100	-0.21000
C	6.33400	2.74500	0.02200
C	7.52500	2.20900	0.49800
C	7.56900	0.84300	0.74800
C	6.47500	0.01300	0.53000
C	6.24200	4.20600	-0.31500
C	8.81200	0.24600	1.34700
F	5.05600	4.71900	0.04300
F	7.19600	4.92100	0.29500
F	6.37100	4.41500	-1.63500
F	9.90700	0.95100	1.02700
F	8.74300	0.21900	2.68800
F	9.00600	-1.01600	0.94200
H	2.64900	-3.76700	-0.78100
H	1.10800	-2.16200	-2.86500
H	2.68100	-2.94400	-3.07700
H	0.80500	-4.34500	-4.02400
H	1.65300	-5.17800	-2.72500
H	-1.00600	-3.67300	-2.44600
H	-0.80000	-5.42300	-2.42100
H	0.52800	-5.27300	-0.33800
H	-1.04100	-4.46200	-0.14000
H	-0.02600	-2.27700	-0.62000
H	3.28500	0.44800	-0.33900
H	1.94100	-0.89400	-0.68000
H	2.84300	-3.49100	1.34800
H	1.89400	-3.47700	2.85900
H	1.61800	-4.76200	1.65500
H	-0.31500	-2.46400	2.87600
H	-1.23200	-2.40700	1.33100
H	-0.75500	-3.97800	2.04100
H	1.10900	-1.88400	1.20500
H	8.38900	2.83800	0.67400

H	6.54300	-1.04600	0.73600
H	4.30500	2.38200	-0.57800
S	-3.23300	-0.82300	-0.82000
C	-4.43400	-1.99200	-1.61200
F	-5.54800	-1.34200	-1.92300
F	-3.88800	-2.48800	-2.71700
F	-4.72900	-2.98600	-0.78500
O	-3.16800	0.30100	-1.75400

TS19

C	1.07996	-0.02833	2.50672
O	-1.15026	0.55096	1.89936
C	3.32405	0.32254	0.49275
C	3.39517	1.59959	1.19903
O	2.53891	2.46542	1.07291
O	2.68364	1.35380	-1.92682
N	-0.87190	3.31020	1.70895
C	0.07992	3.53392	2.83336
C	-0.23076	3.56666	0.35843
C	-1.22834	3.29876	-0.77607
C	-0.50986	3.41639	-2.12539
C	0.15121	4.78652	-2.27391
C	1.11476	5.04580	-1.11613
C	0.39808	4.94945	0.23675
N	-1.81887	1.98473	-0.60096
C	-3.13643	1.68881	-0.62595
N	-3.35834	0.36869	-0.38505
C	-4.56454	-0.33440	-0.28133
S	-4.34351	2.84665	-0.88064
C	-2.14987	4.03214	1.94814
H	-2.04466	4.02702	-0.74864
H	0.25123	2.62825	-2.18922
H	-1.24583	3.23981	-2.91562
H	0.68169	4.84096	-3.22883
H	-0.62134	5.56711	-2.29098
H	1.91857	4.29850	-1.13574
H	1.58060	6.03069	-1.21212
H	-0.36751	5.73385	0.30779
H	1.12424	5.10449	1.03936
H	0.56562	2.81969	0.30946
H	-2.53887	-0.17486	-0.08733
H	-1.15274	1.21616	-0.53156

H	-2.49172	3.78892	2.95435
H	-1.97031	5.10550	1.87049
H	-2.89893	3.71789	1.22077
H	-0.31415	3.01784	3.70995
H	1.05494	3.13448	2.54997
H	0.15084	4.60272	3.03678
H	-1.07088	2.28397	1.72500
H	1.14903	0.82322	3.17418
C	-4.59060	-1.40011	0.62305
C	-5.72524	-2.19406	0.73429
C	-6.85831	-1.93910	-0.02679
C	-6.82041	-0.87312	-0.92035
C	-5.69102	-0.07813	-1.06999
H	-3.71898	-1.59634	1.24137
C	-5.68359	-3.36677	1.67091
H	-7.74395	-2.55588	0.06760
C	-8.04769	-0.54301	-1.72306
H	-5.67945	0.72926	-1.78928
F	-4.94859	-4.37197	1.16620
F	-5.12742	-3.04044	2.84766
F	-6.90482	-3.85557	1.92208
F	-8.80552	-1.62913	-1.93865
F	-8.82208	0.35466	-1.09431
F	-7.73675	-0.02265	-2.91783
O	4.33711	1.83192	2.13542
C	5.41784	0.95939	2.49363
C	5.92860	1.44060	3.83524
H	6.19730	1.01289	1.72804
H	5.07706	-0.07727	2.55219
H	6.78156	0.83359	4.14697
H	6.24723	2.48290	3.76849
H	5.14605	1.36340	4.59337
S	2.18421	0.49549	-0.85161
O	0.80067	0.68630	-0.40434
C	2.12240	-1.20760	-1.60188
F	1.06004	-1.23455	-2.39199
F	1.97753	-2.14051	-0.65921
F	3.20430	-1.47038	-2.31773
C	4.56335	-0.38807	0.03111
C	5.49791	0.28892	-0.76448
C	4.80538	-1.72809	0.34966
C	6.65408	-0.34070	-1.21071
H	5.30684	1.32190	-1.04147

C	5.95605	-2.37310	-0.09095
H	4.09833	-2.28161	0.95763
C	6.87128	-1.66964	-0.86444
H	7.37479	0.18927	-1.82271
H	6.14281	-3.41020	0.16258
Br	8.44510	-2.53980	-1.45408
C	-0.19360	-0.23003	1.90867
N	-0.42054	-1.50302	1.26549
C	-0.04182	-2.74747	1.66942
C	-0.58215	-3.64141	0.77822
H	0.54222	-2.88512	2.56607
C	-1.28937	-2.83555	-0.14135
H	-0.49382	-4.71585	0.79192
H	-1.86436	-3.13764	-1.00587
N	-1.19891	-1.55229	0.16912
C	2.22738	-0.69470	2.11182
H	2.15196	-1.59928	1.52387
H	3.11102	-0.62833	2.73836

TS20

C	1.21600	0.40000	2.59500
O	-1.05100	0.94500	2.15100
C	3.07200	0.02800	0.20600
C	1.75100	-0.12100	-0.35200
O	0.89900	0.76800	-0.36900
O	2.82300	2.54400	1.13800
N	-0.62300	3.61300	1.56500
C	0.32900	3.99000	2.64600
C	0.00600	3.72100	0.19000
C	-1.00100	3.32300	-0.89500
C	-0.28400	3.26700	-2.24900
C	0.36200	4.61500	-2.57100
C	1.32300	5.03800	-1.45900
C	0.61500	5.08800	-0.09800
N	-1.61400	2.05400	-0.54700
C	-2.93100	1.77100	-0.61500
N	-3.21400	0.52400	-0.13400
C	-4.42000	-0.18500	-0.21300
S	-4.09000	2.86600	-1.17200
C	-1.91600	4.33000	1.72700
H	-1.80500	4.06300	-0.95900
H	0.48100	2.48200	-2.20500

H	-1.01800	2.98600	-3.01000
H	0.89300	4.55500	-3.52500
H	-0.42200	5.37500	-2.68800
H	2.14900	4.31800	-1.39600
H	1.75900	6.01700	-1.67600
H	-0.16300	5.86400	-0.11300
H	1.34300	5.35100	0.67500
H	0.80700	2.97500	0.21600
H	-2.52100	0.09300	0.48500
H	-0.95600	1.29300	-0.37400
H	-2.25300	4.18900	2.75400
H	-1.76200	5.39100	1.53100
H	-2.65800	3.92300	1.04000
H	-0.04000	3.56700	3.58100
H	1.31300	3.58700	2.40100
H	0.37400	5.07600	2.72500
H	-0.81100	2.59100	1.70800
H	1.37900	1.41200	2.94400
C	-4.74100	-1.02600	0.85400
C	-5.86000	-1.84800	0.78100
C	-6.70000	-1.82500	-0.32300
C	-6.38100	-0.96500	-1.37100
C	-5.25000	-0.16000	-1.33900
H	-4.10600	-1.04000	1.73300
C	-6.10000	-2.81900	1.90000
H	-7.57400	-2.46400	-0.37200
C	-7.30000	-0.90200	-2.55900
H	-5.00700	0.47400	-2.18200
F	-5.28800	-3.89000	1.79300
F	-5.85100	-2.27100	3.09700
F	-7.35600	-3.28000	1.91200
F	-6.67600	-0.44500	-3.65200
F	-7.80100	-2.11200	-2.85300
F	-8.34600	-0.09400	-2.33300
O	1.48400	-1.37700	-0.73800
C	0.19200	-1.60900	-1.32400
C	0.01400	-3.10700	-1.42500
H	0.16200	-1.12100	-2.30400
H	-0.57900	-1.16300	-0.69100
H	-0.96300	-3.33300	-1.85800
H	0.78800	-3.55100	-2.05500
H	0.06700	-3.55900	-0.43100
S	3.56300	1.71400	0.18000

O	3.74800	2.23400	-1.17700
C	5.27400	1.67900	0.90400
F	5.60300	2.93800	1.15900
F	6.15800	1.16100	0.06800
F	5.29000	0.99300	2.04800
C	4.14700	-0.92500	-0.21200
C	4.89300	-1.64700	0.72300
C	4.43800	-1.09500	-1.57300
C	5.90500	-2.51400	0.32100
H	4.69500	-1.53100	1.78300
C	5.44400	-1.95500	-1.99100
H	3.87300	-0.53100	-2.30900
C	6.17100	-2.65500	-1.03400
H	6.48200	-3.07000	1.05000
H	5.67000	-2.07700	-3.04400
Br	7.55900	-3.81500	-1.59300
C	-0.14200	0.11300	2.27100
N	-0.53200	-1.26200	2.13100
C	-0.04500	-2.36500	2.77500
C	-0.86700	-3.41300	2.45400
H	0.80300	-2.29500	3.43700
C	-1.85200	-2.84500	1.61000
H	-0.79000	-4.43400	2.79200
H	-2.70400	-3.32400	1.14500
N	-1.65100	-1.55100	1.43400
C	2.31600	-0.36900	2.28200
H	2.22800	-1.40900	1.98200
H	3.27500	-0.08100	2.69700

TS21

S	-2.68353	1.27680	-0.87630
O	-2.21819	2.56430	-0.35993
C	-4.53158	1.38549	-0.70552
F	-5.14152	0.36283	-1.27940
F	-4.88924	2.50609	-1.31446
F	-4.88744	1.45818	0.57564
C	-3.11733	-1.23228	0.10770
C	-4.18425	-1.40063	0.99711
C	-2.92296	-2.19007	-0.89655
C	-5.02357	-2.50483	0.90631
C	-3.75083	-3.30254	-0.99463
C	-4.79304	-3.45050	-0.08570

Br	-5.91224	-4.97010	-0.19235
C	-0.44323	2.67149	2.08603
N	-1.43272	3.72243	2.06093
C	-2.58380	3.82128	2.77870
C	-3.19297	4.99343	2.40265
C	-2.31746	5.54301	1.43890
N	-1.25498	4.77843	1.24615
C	-2.12922	0.80366	2.07037
H	-4.37376	-0.66269	1.76852
H	-2.11406	-2.06171	-1.61101
H	-5.84487	-2.63348	1.60152
H	-3.59256	-4.04511	-1.76786
H	-2.83795	3.08049	3.52065
H	-4.11616	5.40405	2.77977
H	-2.41988	6.46805	0.88756
C	-0.87498	1.35468	2.37394
O	0.72786	3.02486	1.90421
H	-0.05504	0.68293	2.61446
C	-2.20361	-0.04949	0.21678
C	-0.76010	-0.22040	0.02745
O	-0.05196	0.63925	-0.48572
O	-2.47182	0.96731	-2.29162
O	-0.10987	-1.26570	0.56065
C	-0.66565	-2.27971	1.42617
C	0.41962	-2.67729	2.40299
H	-0.97858	-3.12253	0.80356
H	-1.54126	-1.88878	1.94426
H	0.03175	-3.43998	3.08241
H	1.28154	-3.09133	1.87713
H	0.74221	-1.81556	2.99219
N	0.39254	3.38456	-1.68633
C	-0.79617	4.14075	-2.18118
C	1.32153	4.26516	-0.85116
C	2.65858	3.56531	-0.58008
C	3.48326	4.40209	0.40398
C	3.73659	5.79921	-0.15901
C	2.41763	6.48366	-0.51187
C	1.57567	5.63686	-1.47558
N	2.45597	2.21285	-0.09599
C	3.25693	1.17986	-0.41746
N	2.81105	-0.00724	0.10679
C	3.46135	-1.24864	0.02646
S	4.61447	1.31764	-1.40994

C	1.03293	2.66746	-2.82651
C	2.71882	-2.37020	-0.33154
C	3.30954	-3.63172	-0.31670
C	4.64488	-3.79501	0.01430
C	5.38307	-2.65975	0.35050
C	4.80955	-1.39824	0.37256
C	2.45969	-4.80665	-0.70269
C	6.81376	-2.83601	0.77465
F	3.04363	-5.97394	-0.40976
F	1.27343	-4.77618	-0.06284
F	2.18123	-4.81394	-2.01462
F	7.44397	-3.74200	0.01161
F	7.50507	-1.69272	0.70627
F	6.89845	-3.27513	2.04193
H	3.22335	3.48737	-1.51533
H	2.93510	4.45637	1.35271
H	4.42354	3.87228	0.58389
H	4.29624	6.40178	0.56233
H	4.35944	5.71782	-1.05953
H	1.83594	6.64527	0.40458
H	2.59560	7.46634	-0.95797
H	2.09747	5.53426	-2.43703
H	0.63256	6.15610	-1.65913
H	0.77097	4.39765	0.08621
H	1.80502	-0.04849	0.26252
H	1.80350	2.12337	0.69122
H	0.26177	2.05895	-3.29988
H	1.42020	3.40335	-3.53236
H	1.83418	2.02598	-2.46711
H	-1.49872	3.42261	-2.59951
H	-1.25242	4.65463	-1.33566
H	-0.46246	4.83660	-2.94983
H	0.03443	2.65595	-1.04373
H	5.10368	-4.77721	0.01493
H	5.39522	-0.53226	0.65409
H	1.67496	-2.25562	-0.60448
H	-2.96924	1.47284	1.91521
H	-2.40342	-0.12063	2.57346

TS22

S	1.69358	-2.85576	-1.79714
O	0.69121	-3.91900	-1.90087

C	3.28531	-3.79814	-1.60111
F	3.34758	-4.37169	-0.39843
F	4.34374	-3.02282	-1.77039
F	3.28860	-4.74585	-2.52705
C	2.87122	-1.29320	0.06341
C	3.74042	-1.74885	1.05802
C	3.22147	-0.14280	-0.65741
C	4.91342	-1.06162	1.35157
C	4.38693	0.55917	-0.37203
C	5.22019	0.09107	0.63691
Br	6.79472	1.04995	1.05906
C	-1.20263	-2.55490	2.06415
N	-1.86323	-1.89543	3.16457
C	-1.58486	-1.93450	4.49558
C	-2.45223	-1.06500	5.11163
C	-3.22403	-0.53190	4.05409
N	-2.86574	-1.03925	2.88549
C	0.82610	-3.47964	1.17819
H	3.49305	-2.63564	1.63146
H	2.57176	0.20818	-1.45546
H	5.57954	-1.41064	2.13214
H	4.63598	1.45995	-0.91974
H	-0.82428	-2.59071	4.88961
H	-2.52852	-0.85164	6.16590
H	-4.01625	0.20266	4.09966
C	0.14979	-2.93523	2.26163
O	-1.86444	-2.68964	1.03570
H	0.66311	-2.66689	3.17622
C	1.57230	-1.98395	-0.24940
C	0.35303	-1.18190	-0.28336
O	-0.52795	-1.29134	-1.12830
O	1.92696	-1.97993	-2.94329
O	0.09513	-0.31468	0.73031
C	1.00527	0.13315	1.76104
C	0.17631	0.89123	2.77557
H	1.75762	0.78661	1.30923
H	1.50259	-0.72831	2.20573
H	0.82939	1.24814	3.57544
H	-0.30057	1.76152	2.31533
H	-0.59767	0.25593	3.21139
N	-3.03549	-2.50182	-1.42691
C	-2.52523	-3.89199	-1.58628
C	-4.31858	-2.44442	-0.63373

C	-4.75015	-0.99015	-0.41308
C	-5.93106	-0.95164	0.56068
C	-7.09694	-1.77847	0.01472
C	-6.66455	-3.21312	-0.29388
C	-5.46051	-3.24955	-1.24521
N	-3.61889	-0.20829	0.04355
C	-3.29037	1.00811	-0.44780
N	-2.09859	1.44855	0.05681
C	-1.43434	2.65141	-0.23259
S	-4.20926	1.84853	-1.58309
C	-3.06015	-1.79486	-2.73583
C	-0.06168	2.59441	-0.47362
C	0.66870	3.76495	-0.64758
C	0.05000	5.00536	-0.62369
C	-1.32601	5.04829	-0.40548
C	-2.07115	3.89597	-0.19482
C	2.15473	3.64080	-0.81910
C	-2.02900	6.37722	-0.41865
F	2.73212	3.14923	0.29367
F	2.47418	2.79004	-1.81088
F	2.73696	4.81249	-1.08929
F	-2.46959	6.69158	-1.64506
F	-3.09407	6.38340	0.39529
F	-1.21468	7.36973	-0.02848
H	-5.08027	-0.55474	-1.36139
H	-5.60130	-1.34043	1.53347
H	-6.22141	0.09376	0.70207
H	-7.92564	-1.77885	0.72856
H	-7.46858	-1.30734	-0.90440
H	-6.39251	-3.72113	0.64016
H	-7.49207	-3.77657	-0.73391
H	-5.74762	-2.82187	-2.21422
H	-5.15763	-4.28678	-1.41466
H	-4.03570	-2.87467	0.33325
H	-1.48266	0.70723	0.38941
H	-3.17231	-0.53297	0.91076
H	-2.06584	-1.88752	-3.17173
H	-3.80502	-2.26094	-3.38142
H	-3.29004	-0.74034	-2.58211
H	-1.47589	-3.82862	-1.87649
H	-2.61129	-4.39906	-0.62618
H	-3.10878	-4.40214	-2.35304
H	-2.32258	-2.01883	-0.85123

H	0.61914	5.91695	-0.76030
H	-3.13336	3.95669	0.00546
H	0.43304	1.63019	-0.52817
H	0.23558	-3.90781	0.37340
H	1.80494	-3.92259	1.31951

TS23

S	1.28402	0.51118	0.40874
O	1.25663	1.50716	-0.66299
C	1.98110	1.45513	1.85244
F	2.43125	0.63907	2.80606
F	0.98480	2.17955	2.34021
F	2.95959	2.25985	1.47277
C	3.83561	-0.18213	-0.08577
C	4.75167	-0.03917	0.96221
C	4.21304	0.26215	-1.36150
C	6.01305	0.50411	0.74432
C	5.47334	0.79838	-1.59759
C	6.36503	0.90999	-0.53671
Br	8.08511	1.64124	-0.83192
C	-0.00477	-2.53013	2.08646
N	-1.11922	-2.31892	2.97855
C	-1.12009	-2.16161	4.32910
C	-2.42659	-1.97610	4.71216
C	-3.15586	-2.02896	3.50384
N	-2.36063	-2.24252	2.46792
C	2.33200	-2.22839	1.69276
H	4.48174	-0.33373	1.96976
H	3.50233	0.19435	-2.18116
H	6.71678	0.61335	1.56135
H	5.75586	1.13708	-2.58773
H	-0.20651	-2.22082	4.89956
H	-2.80287	-1.82916	5.71193
H	-4.21963	-1.91189	3.34785
C	1.25184	-2.08365	2.55166
O	-0.25252	-3.05658	0.99902
H	1.30644	-1.44449	3.42140
C	2.46832	-0.77924	0.11559
C	1.93367	-1.59850	-0.98883
O	0.80016	-1.50408	-1.43314
O	0.01081	0.00547	0.93164
O	2.70463	-2.56982	-1.52344

C	4.02350	-2.94668	-1.09152
C	4.14179	-4.44157	-1.30151
H	4.18058	-2.68167	-0.04525
H	4.75105	-2.39402	-1.69307
H	5.14910	-4.77528	-1.04248
H	3.42484	-4.97546	-0.67163
H	3.94822	-4.69696	-2.34585
N	-1.09780	-3.58222	-1.55764
C	0.02338	-4.55990	-1.56843
C	-2.33524	-4.13339	-0.88260
C	-3.44204	-3.07690	-0.87987
C	-4.63556	-3.57993	-0.06050
C	-5.15881	-4.89627	-0.63506
C	-4.04715	-5.94384	-0.70051
C	-2.83716	-5.43677	-1.49712
N	-2.90902	-1.82535	-0.37991
C	-3.31837	-0.61197	-0.82251
N	-2.71023	0.40710	-0.15130
C	-2.94158	1.78765	-0.28684
S	-4.40631	-0.40209	-2.09506
C	-1.32070	-3.01244	-2.91516
C	-1.83409	2.63184	-0.27592
C	-2.00985	4.01056	-0.30227
C	-3.27576	4.57341	-0.36082
C	-4.37437	3.71632	-0.38176
C	-4.22665	2.33648	-0.33325
C	-0.77555	4.86614	-0.28108
C	-5.75550	4.29791	-0.48654
F	0.02537	4.54127	0.74688
F	-0.04977	4.71308	-1.39703
F	-1.06897	6.16997	-0.16750
F	-6.14662	4.42407	-1.76380
F	-6.66805	3.53055	0.12739
F	-5.82066	5.52119	0.06254
H	-3.78863	-2.90129	-1.90315
H	-4.31717	-3.71437	0.98183
H	-5.40722	-2.80429	-0.07920
H	-5.99219	-5.26711	-0.03136
H	-5.55165	-4.71728	-1.64430
H	-3.72163	-6.19199	0.31784
H	-4.41472	-6.86972	-1.15175
H	-3.13365	-5.27166	-2.54084
H	-2.05235	-6.19812	-1.48946

H	-2.00552	-4.30254	0.14913
H	-1.83769	0.16994	0.32206
H	-2.51679	-1.88451	0.57076
H	-0.36026	-2.64324	-3.26936
H	-1.70567	-3.79337	-3.57176
H	-2.02399	-2.18174	-2.85045
H	0.92918	-4.01839	-1.84077
H	0.12342	-4.97309	-0.56498
H	-0.18932	-5.34154	-2.29780
H	-0.77123	-2.80707	-0.95474
H	-3.40921	5.64833	-0.37841
H	-5.09607	1.69167	-0.32258
H	-0.83289	2.21511	-0.26312
H	2.25990	-3.03765	0.97005
H	3.33338	-2.02273	2.05503

TS24

S	1.75689	-1.69539	-1.18789
O	0.50306	-2.42618	-0.97976
C	2.96336	-3.09376	-1.41844
F	2.37969	-3.95908	-2.24287
F	3.21796	-3.71854	-0.26744
F	4.10030	-2.68636	-1.95483
C	3.68288	-0.30686	0.15693
C	3.88455	1.02352	-0.24301
C	4.80823	-1.09339	0.42131
C	5.16044	1.55618	-0.35517
C	6.09578	-0.57664	0.30274
C	6.25797	0.74658	-0.07918
Br	8.00322	1.46208	-0.24120
C	-0.37412	-2.66429	1.99653
N	-0.72877	-1.60696	2.92158
C	-0.14492	-1.26457	4.10156
C	-0.94232	-0.30471	4.68049
C	-2.00548	-0.13571	3.76460
N	-1.87317	-0.93201	2.71474
C	2.06671	-2.13340	1.96337
H	3.02966	1.65401	-0.46619
H	4.69542	-2.13199	0.71075
H	5.30549	2.58488	-0.66419
H	6.96102	-1.19592	0.50842
H	0.75134	-1.76023	4.44157
H	-0.79141	0.18839	5.62787

H	-2.85296	0.53337	3.82533
C	0.99014	-3.00338	1.83838
O	-1.32468	-3.22448	1.44221
H	1.13095	-3.91732	1.27229
C	2.29513	-0.85686	0.27162
C	1.22908	0.03213	0.73111
O	0.04794	-0.07966	0.42966
O	1.91564	-0.88761	-2.39427
O	1.66962	0.91365	1.64054
C	0.68911	1.77874	2.24492
C	1.27393	2.27138	3.54911
H	-0.23573	1.21785	2.40135
H	0.48962	2.60187	1.55240
H	0.56289	2.93917	4.04179
H	1.47417	1.42789	4.21453
H	2.20483	2.81617	3.37740
N	-2.03765	-3.64344	-1.15849
C	-1.19928	-4.87342	-1.19106
C	-3.37015	-3.87747	-0.47857
C	-4.17975	-2.57909	-0.41000
C	-5.42703	-2.80085	0.45200
C	-6.28661	-3.91853	-0.13850
C	-5.47794	-5.20542	-0.30306
C	-4.20603	-4.97632	-1.13118
N	-3.36532	-1.46727	0.04426
C	-3.54920	-0.20414	-0.41679
N	-2.69519	0.69213	0.14854
C	-2.59083	2.05719	-0.18872
S	-4.68195	0.20606	-1.60060
C	-2.11875	-3.01091	-2.50370
C	-1.35103	2.55108	-0.58035
C	-1.19072	3.91520	-0.81477
C	-2.25690	4.79225	-0.69609
C	-3.50027	4.27814	-0.32697
C	-3.67697	2.92822	-0.06238
C	0.16640	4.40088	-1.23421
C	-4.66617	5.22120	-0.23482
F	0.46144	4.03765	-2.48864
F	0.27138	5.73312	-1.16440
F	1.13248	3.87896	-0.44914
F	-5.14601	5.53294	-1.44770
F	-5.67835	4.70445	0.47370
F	-4.31376	6.37838	0.34863

H	-4.52321	-2.32057	-1.41660
H	-5.11834	-3.04977	1.47593
H	-5.97998	-1.85782	0.48896
H	-7.15985	-4.10009	0.49459
H	-6.66384	-3.59678	-1.11771
H	-5.19051	-5.58396	0.68615
H	-6.08210	-5.98327	-0.77821
H	-4.48181	-4.68594	-2.15280
H	-3.64164	-5.91087	-1.19023
H	-3.09001	-4.18160	0.53385
H	-1.86850	0.30130	0.60134
H	-2.84044	-1.57697	0.91972
H	-2.66970	-3.67054	-3.17481
H	-2.61434	-2.04300	-2.42455
H	-1.09897	-2.86620	-2.85764
H	-0.19306	-4.57605	-1.48147
H	-1.18708	-5.29743	-0.18725
H	-1.61463	-5.57595	-1.91267
H	-1.52476	-2.99720	-0.53399
H	-2.12613	5.85348	-0.87514
H	-4.64151	2.54649	0.24877
H	-0.51839	1.86471	-0.69303
H	3.06398	-2.55006	1.88421
H	2.01402	-1.25771	2.60353

TS25

S	-0.69253	-1.56062	0.38940
O	-0.45771	-2.05547	-0.96834
C	-1.43186	-3.01937	1.28106
F	-1.99092	-2.64319	2.43107
F	-0.42909	-3.84209	1.53790
F	-2.33077	-3.64200	0.53818
C	-3.30516	-0.93603	0.11893
C	-4.22984	-1.19674	1.13445
C	-3.63762	-1.27707	-1.19954
C	-5.46663	-1.76340	0.84401
C	-4.87033	-1.83953	-1.50453
C	-5.77550	-2.07495	-0.47434
Br	-7.46058	-2.83858	-0.86992
C	-0.47072	2.67013	2.07286
N	-1.68200	3.47069	1.95230
C	-2.71083	3.55796	2.83938

C	-3.54167	4.55936	2.39342
C	-2.91244	5.03335	1.22122
N	-1.78573	4.38235	0.96795
C	-1.79555	0.57302	2.33074
H	-3.99274	-0.95578	2.16493
H	-2.91464	-1.10238	-1.99102
H	-6.18474	-1.96024	1.63150
H	-5.12517	-2.10040	-2.52521
H	-2.73775	2.92852	3.71575
H	-4.45207	4.91122	2.85215
H	-3.23887	5.82138	0.55561
C	-0.62930	1.32047	2.46873
O	0.58948	3.25564	1.85165
H	0.29638	0.80915	2.70263
C	-1.97111	-0.31923	0.41380
C	-1.50196	0.81243	-0.36644
O	-0.29976	1.04504	-0.51689
O	0.45684	-1.20897	1.22587
O	-2.35455	1.70604	-0.90247
C	-3.66604	2.02162	-0.39026
C	-4.24675	3.07175	-1.31179
H	-3.56183	2.40907	0.62793
H	-4.30014	1.13618	-0.37781
H	-5.23827	3.36022	-0.95471
H	-3.61478	3.96154	-1.33057
H	-4.34338	2.67814	-2.32676
N	0.47042	3.43106	-1.86481
C	-0.84250	3.99174	-2.28433
C	1.23141	4.38362	-0.96010
C	2.58258	3.77309	-0.57147
C	3.26808	4.65718	0.47399
C	3.45875	6.07276	-0.07182
C	2.12600	6.66834	-0.52722
C	1.42748	5.77296	-1.56029
N	2.39575	2.40883	-0.11681
C	3.20322	1.37677	-0.44636
N	2.77914	0.18978	0.06474
C	3.46157	-1.04234	-0.04292
S	4.56584	1.53664	-1.43520
C	1.23410	2.93504	-3.04301
C	2.78476	-2.13983	-0.55800
C	3.40077	-3.38944	-0.56744
C	4.69702	-3.55300	-0.10848

C	5.37580	-2.43352	0.37512
C	4.77099	-1.18732	0.42519
C	2.59891	-4.55436	-1.07052
C	6.79270	-2.60699	0.84039
F	1.50639	-4.75546	-0.31653
F	2.16865	-4.35258	-2.32484
F	3.30471	-5.69443	-1.06580
F	7.23125	-1.55201	1.53878
F	6.92120	-3.69114	1.62265
F	7.63391	-2.77927	-0.19178
H	3.23100	3.72955	-1.45245
H	2.65259	4.66964	1.38117
H	4.22966	4.19818	0.72284
H	3.91584	6.71148	0.68944
H	4.15327	6.04251	-0.92153
H	1.46435	6.78348	0.34057
H	2.27331	7.66452	-0.95388
H	2.03983	5.71661	-2.46979
H	0.46584	6.21792	-1.83037
H	0.59771	4.45677	-0.06856
H	1.80744	0.12349	0.37117
H	1.75134	2.30523	0.67149
H	0.58354	2.26272	-3.60222
H	1.51532	3.78261	-3.66896
H	2.12205	2.39451	-2.71510
H	-1.43660	3.17998	-2.70240
H	-1.33418	4.38353	-1.39289
H	-0.67986	4.76906	-3.03067
H	0.24956	2.59270	-1.28193
H	5.17067	-4.52839	-0.11737
H	5.29974	-0.33199	0.82841
H	1.77804	-2.02489	-0.94527
H	-2.75860	1.06498	2.23154
H	-1.84164	-0.38523	2.83697

TS26

S	-1.05471	-0.23250	-0.93471
O	-0.00124	0.78066	-0.74416
C	-2.36906	0.71952	-1.83138
F	-2.86121	1.68459	-1.06211
F	-3.34452	-0.04782	-2.27644
F	-1.76055	1.28539	-2.87956

C	-3.20682	-1.24082	0.36290
C	-4.31264	-0.42053	0.59741
C	-3.42202	-2.54634	-0.09989
C	-5.60747	-0.88452	0.38281
C	-4.70722	-3.02488	-0.31601
C	-5.78868	-2.18383	-0.07122
Br	-7.54614	-2.82546	-0.36932
C	-1.30251	3.12110	1.49957
N	-1.77816	4.47830	1.61728
C	-2.84483	4.95202	2.32697
C	-2.84968	6.31501	2.18593
C	-1.72887	6.58459	1.36505
N	-1.08442	5.47991	1.03502
C	-1.70409	0.82517	2.11392
H	-4.15888	0.59745	0.93740
H	-2.56811	-3.18792	-0.29694
H	-6.46451	-0.24647	0.56471
H	-4.87194	-4.03374	-0.67602
H	-3.48573	4.29646	2.89349
H	-3.54628	7.01593	2.61757
H	-1.37144	7.54039	1.00714
H	-0.62298	0.71041	2.11941
H	-2.27182	0.12598	2.72185
C	-2.19754	2.10281	1.92448
O	-0.15447	2.96788	1.07933
H	-3.23334	2.33811	2.12929
C	-1.81567	-0.72616	0.56639
C	-0.85693	-1.54740	1.29939
O	0.35902	-1.39661	1.24868
O	-0.74561	-1.35570	-1.82332
O	-1.44330	-2.39987	2.14710
C	-0.55548	-3.16411	2.98826
C	-0.08679	-2.33805	4.17016
H	0.28224	-3.51569	2.38238
H	-1.15684	-4.01664	3.30605
H	0.52709	-2.95286	4.83301
H	0.51264	-1.49248	3.82784
H	-0.94300	-1.96581	4.73812
N	0.71430	3.45349	-1.60375
C	-0.52898	4.18288	-1.97956
C	1.64232	4.31196	-0.76914
C	2.88055	3.50911	-0.35993
C	3.67134	4.30781	0.67941

C	4.08563	5.65970	0.09292
C	2.87578	6.43687	-0.43181
C	2.06287	5.61367	-1.44231
N	2.52067	2.17308	0.08409
C	3.25121	1.07170	-0.22125
N	2.68072	-0.08883	0.19157
C	3.20362	-1.38822	0.02202
S	4.70965	1.14711	-1.07163
C	1.33886	2.80819	-2.79125
C	2.37134	-2.36336	-0.51994
C	2.79908	-3.68642	-0.56761
C	4.06551	-4.04964	-0.13494
C	4.90286	-3.05406	0.36575
C	4.48175	-1.73459	0.46562
C	1.81832	-4.70995	-1.05948
C	6.29636	-3.41858	0.79090
F	2.34644	-5.94053	-1.10609
F	0.73927	-4.76863	-0.25520
F	1.36345	-4.41715	-2.28504
F	6.76625	-2.58852	1.73297
F	6.35086	-4.66487	1.28745
F	7.15859	-3.37373	-0.23644
H	3.52778	3.37571	-1.23291
H	3.04993	4.44766	1.57332
H	4.54761	3.71965	0.96634
H	4.61500	6.25078	0.84555
H	4.79088	5.48721	-0.73025
H	2.21899	6.70107	0.40698
H	3.19452	7.37343	-0.89765
H	2.67202	5.41260	-2.33283
H	1.18607	6.18859	-1.75390
H	1.03847	4.54645	0.11168
H	1.68683	-0.07556	0.42257
H	1.69421	2.07633	0.67345
H	0.57954	2.18836	-3.26662
H	1.67839	3.58437	-3.47767
H	2.17284	2.18106	-2.47727
H	-1.26344	3.45079	-2.30895
H	-0.88458	4.72483	-1.10253
H	-0.30531	4.87017	-2.79523
H	0.41608	2.69114	-0.97162
H	4.39224	-5.08203	-0.17420
H	5.13002	-0.97982	0.89329

H	1.38344	-2.09376	-0.88029

TS27

S	-0.50798	-1.82074	0.35947
O	-0.25430	-2.47559	-0.92516
C	-1.18792	-3.17466	1.43846
F	-2.07036	-3.92046	0.79564
F	-1.75364	-2.66392	2.53212
F	-0.15737	-3.92444	1.79341
C	-3.13581	-1.31458	0.06333
C	-4.08187	-1.44519	1.08423
C	-3.42162	-1.87353	-1.18951
C	-5.29144	-2.09352	0.86176
C	-4.62661	-2.52462	-1.42849
C	-5.55272	-2.62430	-0.39617
Br	-7.20233	-3.49563	-0.70556
C	-1.58872	2.81407	1.63511
N	-2.28821	4.07580	1.68068
C	-3.35080	4.43984	2.45371
C	-3.69445	5.71688	2.08870
C	-2.77194	6.04782	1.06900
N	-1.92635	5.05926	0.83034
C	-1.61116	0.46957	2.16239
H	-3.88395	-1.02223	2.06322
H	-2.68151	-1.80559	-1.98222
H	-6.02532	-2.18645	1.65407
H	-4.84261	-2.95406	-2.40009
H	-3.74859	3.78374	3.21149
H	-4.48104	6.32791	2.50208
H	-2.69605	6.96637	0.50305
H	-0.53451	0.47271	2.02219
H	-1.99467	-0.35820	2.74827
C	-2.26584	1.69261	2.18334
O	-0.47383	2.81623	1.11277
H	-3.29128	1.77697	2.51891
C	-1.81913	-0.62740	0.29199
C	-1.38380	0.46939	-0.56927
O	-0.20372	0.65807	-0.87911
O	0.63207	-1.31667	1.13335
O	-2.25407	1.40304	-0.98868
C	-3.67511	1.41596	-0.73748
C	-4.16607	2.81960	-1.01936

H	-3.86525	1.13635	0.29784
H	-4.15624	0.68911	-1.39720
H	-5.24006	2.87057	-0.82506
H	-3.66153	3.54779	-0.37833
H	-3.99626	3.09158	-2.06423
N	0.24538	3.34066	-1.93116
C	-1.08924	3.78211	-2.41743
C	0.86036	4.36332	-0.99899
C	2.20380	3.86111	-0.46405
C	2.64879	4.77847	0.67732
C	2.78994	6.21420	0.16603
C	1.49847	6.70218	-0.49489
C	1.02900	5.75151	-1.60675
N	2.12177	2.46442	-0.08113
C	3.08535	1.55515	-0.35767
N	2.74116	0.28943	-0.00634
C	3.55945	-0.85666	-0.05835
S	4.54379	1.95801	-1.11542
C	1.11403	2.91565	-3.06249
C	3.01809	-2.02827	-0.57823
C	3.75460	-3.20874	-0.53601
C	5.04209	-3.23493	-0.02319
C	5.58145	-2.04652	0.46695
C	4.85287	-0.86562	0.47077
C	3.09170	-4.45955	-1.03542
C	6.99387	-2.04630	0.97652
F	3.92486	-5.51075	-1.02755
F	2.03032	-4.78427	-0.28008
F	2.64189	-4.31274	-2.29063
F	7.29466	-3.19760	1.59883
F	7.88095	-1.90446	-0.02151
F	7.21572	-1.04850	1.84332
H	2.95869	3.91900	-1.25508
H	1.90523	4.72263	1.48308
H	3.59976	4.40490	1.06787
H	3.06788	6.88149	0.98697
H	3.60805	6.25062	-0.56510
H	0.70412	6.76545	0.26005
H	1.63473	7.70607	-0.90697
H	1.76431	5.73649	-2.42197
H	0.08143	6.11788	-2.01226
H	0.13981	4.42390	-0.18035
H	1.75432	0.09924	0.17001

H	1.31838	2.18778	0.48347
H	0.58940	2.13258	-3.60979
H	1.29476	3.77140	-3.71396
H	2.05697	2.52290	-2.68166
H	-1.59482	2.91815	-2.84568
H	-1.65015	4.16067	-1.56057
H	-0.95608	4.55849	-3.17052
H	0.07860	2.48327	-1.36162
H	5.61136	-4.15655	0.00392
H	5.27711	0.04026	0.88500
H	2.01721	-2.02599	-0.99842

TS28

C	-3.36502	-0.44638	0.54379
C	-4.41824	0.46971	0.44281
C	-3.61964	-1.79123	0.23318
C	-5.69271	0.05940	0.06411
C	-4.88856	-2.21388	-0.13684
C	-5.91738	-1.28118	-0.21564
Br	-7.64593	-1.84652	-0.73591
C	-0.15542	3.01475	2.10726
N	-1.06121	4.06473	1.67333
C	-2.20883	4.48036	2.27564
C	-2.67194	5.55925	1.56229
C	-1.71588	5.73020	0.53695
N	-0.74174	4.83737	0.62047
C	-2.01746	1.38013	2.37244
H	-4.24428	1.52387	0.62440
H	-2.81401	-2.51673	0.27647
H	-6.50075	0.77692	-0.01736
H	-5.07657	-3.25440	-0.37485
H	-2.56629	4.00083	3.17338
H	-3.55360	6.14763	1.76086
H	-1.69984	6.46930	-0.25295
H	-2.49039	0.66021	3.03828
H	-2.72105	2.05077	1.88991
C	-0.74529	1.87685	2.70142
O	1.05019	3.23541	1.95569
H	-0.04465	1.23734	3.22688
C	-1.97887	0.00588	0.89688
O	0.16339	1.11984	-0.31942
O	0.15133	-0.85179	1.61161

N	1.47477	3.13005	-1.88409
C	0.19528	3.72573	-2.36477
C	2.19013	4.02506	-0.88832
C	3.37100	3.27163	-0.25922
C	3.98219	4.12960	0.85343
C	4.41411	5.49219	0.30941
C	3.24260	6.21177	-0.36017
C	2.63382	5.35687	-1.47995
N	2.91276	1.98632	0.23561
C	3.42479	0.78419	-0.10094
N	2.70206	-0.25257	0.40596
C	2.91746	-1.62364	0.16699
S	4.79242	0.58369	-1.08003
C	2.30408	2.64966	-3.02326
C	1.82295	-2.37894	-0.25407
C	1.93796	-3.75461	-0.39017
C	3.14924	-4.39716	-0.15770
C	4.24219	-3.62727	0.22586
C	4.13884	-2.25111	0.40692
C	0.73692	-4.56772	-0.78071
C	5.57227	-4.29495	0.43570
F	0.85072	-5.07847	-2.01460
F	0.55990	-5.60567	0.05337
F	-0.39324	-3.83930	-0.75464
F	6.29455	-4.32387	-0.69473
F	6.30963	-3.65883	1.35616
F	5.42788	-5.56683	0.83885
H	4.14154	3.07505	-1.01073
H	3.23377	4.25234	1.64457
H	4.83292	3.58456	1.27328
H	4.81966	6.10493	1.11966
H	5.22158	5.35572	-0.42210
H	2.46446	6.41706	0.38573
H	3.56295	7.17394	-0.76968
H	3.38258	5.19988	-2.26706
H	1.78279	5.88260	-1.92376
H	1.43602	4.20420	-0.11314
H	1.76168	-0.04353	0.74836
H	2.17745	2.04540	0.94859
H	1.68811	1.97807	-3.62192
H	2.60850	3.50519	-3.62622
H	3.17387	2.10826	-2.64819
H	-0.36910	2.94343	-2.87256

H	-0.35981	4.08736	-1.49805
H	0.41835	4.53355	-3.06175
H	1.19863	2.30202	-1.32932
H	3.23790	-5.47381	-0.25577
H	4.99085	-1.67219	0.74149
H	0.87994	-1.88392	-0.44480
C	-1.06454	-0.92060	1.58963
C	-0.97880	-2.59672	3.26251
C	-0.37978	-3.77832	2.52673
H	-0.20361	-1.98771	3.73361
H	-1.69839	-2.91540	4.01790
H	0.09401	-4.45625	3.24123
H	-1.15122	-4.33150	1.98553
H	0.37894	-3.43823	1.82062
O	-1.75203	-1.75979	2.37935
S	-1.29533	0.94497	-0.41611
O	-2.11154	2.12872	-0.66546
C	-1.48290	-0.09974	-1.94418
F	-2.73247	-0.12020	-2.36769
F	-0.70448	0.42069	-2.89354
F	-1.07958	-1.34043	-1.68395

TS29

S	-0.84493	-1.56293	1.42958
O	0.51874	-1.07330	1.59944
C	-0.72108	-2.88747	0.12825
F	-0.55330	-2.31770	-1.06745
F	-1.81284	-3.63236	0.10311
F	0.32760	-3.64576	0.40695
C	-3.26010	-1.01170	0.42502
C	-4.16582	-1.37472	1.42835
C	-3.58136	-1.31183	-0.90676
C	-5.36708	-2.00027	1.11297
C	-4.78252	-1.92746	-1.23500
C	-5.66725	-2.26640	-0.21742
Br	-7.29243	-3.12991	-0.64998
C	-0.69653	2.93449	2.00141
N	-1.87812	3.73297	1.69798
C	-3.00667	3.87230	2.44580
C	-3.77044	4.84668	1.84679
C	-3.00038	5.25481	0.73523
N	-1.85599	4.58890	0.65932

C	-2.05376	0.86592	2.39517
H	-3.92567	-1.18933	2.46881
H	-2.87930	-1.06716	-1.69920
H	-6.06181	-2.28441	1.89476
H	-5.02295	-2.15773	-2.26656
H	-3.14354	3.30051	3.35072
H	-4.72604	5.22432	2.17421
H	-3.23427	6.00466	-0.00858
H	-2.19279	0.03879	3.08653
H	-2.98768	1.32829	2.08607
C	-0.90580	1.65502	2.55596
O	0.38930	3.48279	1.79204
H	-0.01107	1.19905	2.96496
C	-1.95305	-0.35558	0.75430
C	-1.37061	0.59764	-0.19412
O	-0.16924	0.76904	-0.36710
O	-1.51147	-2.21473	2.55124
O	-2.18063	1.43473	-0.87365
C	-3.51793	1.85131	-0.51629
C	-4.32009	1.97798	-1.79319
H	-3.42045	2.81780	-0.01704
H	-3.98672	1.14349	0.16495
H	-5.31973	2.35135	-1.55899
H	-3.84477	2.68134	-2.48151
H	-4.41897	1.00947	-2.28887
N	0.44202	3.20742	-1.92510
C	-0.88234	3.62672	-2.46235
C	1.09437	4.30460	-1.09824
C	2.44656	3.82584	-0.55271
C	2.99152	4.86198	0.43495
C	3.14427	6.21938	-0.25310
C	1.82245	6.67159	-0.87534
C	1.26495	5.62224	-1.84838
N	2.30765	2.50616	0.03189
C	3.13593	1.46898	-0.21542
N	2.72684	0.31179	0.37255
C	3.38174	-0.93006	0.20323
S	4.51334	1.58038	-1.19275
C	1.29365	2.65203	-3.01405
C	4.65954	-1.14006	0.71479
C	5.26684	-2.37858	0.54352
C	4.61623	-3.41421	-0.11902
C	3.33846	-3.19052	-0.61830

C	2.71934	-1.95522	-0.46675
C	6.66870	-2.59447	1.03815
C	2.63701	-4.31445	-1.32660
F	6.86114	-3.86250	1.43297
F	7.57542	-2.34499	0.08095
F	6.96100	-1.79693	2.07444
F	1.53760	-3.89693	-1.97153
F	3.43809	-4.89626	-2.23315
F	2.25820	-5.28118	-0.47666
H	3.16320	3.73234	-1.37474
H	2.30225	4.93274	1.28385
H	3.95386	4.49940	0.80804
H	3.49797	6.96604	0.46369
H	3.90834	6.14356	-1.03780
H	1.08313	6.83729	-0.08168
H	1.94876	7.62193	-1.40173
H	1.95737	5.50024	-2.69163
H	0.30866	5.97450	-2.24480
H	0.39833	4.44885	-0.26479
H	1.74324	0.24877	0.63994
H	1.63646	2.44667	0.80360
H	0.73053	1.85984	-3.50771
H	1.51721	3.44602	-3.72728
H	2.21604	2.24264	-2.60200
H	-1.38264	2.73957	-2.84701
H	-1.45930	4.05697	-1.64187
H	-0.72712	4.35027	-3.26195
H	0.26079	2.42188	-1.26639
H	5.09828	-4.37827	-0.24323
H	1.73351	-1.77148	-0.87787
H	5.16818	-0.33932	1.23899

TS30

S	-0.30914	-0.81495	1.44714
O	1.11034	-1.04200	1.73249
C	-1.13615	-1.47292	2.97797
F	-0.43889	-1.02037	4.01010
F	-1.10030	-2.80527	2.98366
F	-2.38629	-1.06138	3.08225
C	-2.44746	-1.93593	0.25419
C	-3.11267	-3.03328	0.80909
C	-3.21103	-0.85710	-0.21246

C	-4.50203	-3.06949	0.86974
C	-4.59870	-0.87884	-0.16170
C	-5.23128	-1.99411	0.37642
Br	-7.12027	-2.04371	0.44705
C	2.32975	-3.19447	-0.62943
N	3.10683	-2.95991	-1.82713
C	2.63752	-2.63004	-3.06225
C	3.73532	-2.43335	-3.86453
C	4.83760	-2.66525	-3.00908
N	4.45357	-2.97043	-1.77866
C	0.12848	-3.66036	0.22891
H	-2.55075	-3.86927	1.21067
H	-2.71431	0.02050	-0.61534
H	-5.01368	-3.92418	1.29713
H	-5.17364	-0.03590	-0.52663
H	1.57809	-2.50691	-3.23605
H	3.74526	-2.14840	-4.90473
H	5.89292	-2.62060	-3.24409
H	-0.75828	-4.28618	0.21359
H	0.54705	-3.47723	1.21387
C	1.04834	-3.75872	-0.82033
O	2.84944	-2.88051	0.44339
H	0.75582	-4.11796	-1.79930
C	-0.94711	-1.87206	0.15215
C	-0.36279	-1.40345	-1.10853
O	0.65170	-0.71150	-1.15564
O	-0.78890	0.55941	1.30984
O	-0.83130	-1.83520	-2.28066
C	-1.80972	-2.87821	-2.45414
C	-1.57470	-3.46510	-3.82900
H	-2.80891	-2.44406	-2.36882
H	-1.69014	-3.63209	-1.67293
H	-2.32023	-4.23745	-4.03089
H	-1.65926	-2.68993	-4.59333
H	-0.58071	-3.91517	-3.89509
N	3.97485	-1.02351	2.05516
C	4.29845	-2.28037	2.78695
C	4.90482	-0.78161	0.88207
C	4.59700	0.56129	0.21819
C	5.39712	0.68061	-1.08340
C	6.89484	0.56186	-0.80073
C	7.20931	-0.73175	-0.04948
C	6.38693	-0.84898	1.24215

N	3.16994	0.74520	0.00910
C	2.57674	1.97337	0.05191
N	1.29276	1.93569	-0.38053
C	0.28102	2.89612	-0.47506
S	3.38589	3.34778	0.60177
C	3.83428	0.11465	3.00353
C	-0.90282	2.43184	-1.07254
C	-2.00230	3.25858	-1.19920
C	-1.96192	4.58222	-0.76380
C	-0.78414	5.03543	-0.19324
C	0.33592	4.21814	-0.03347
C	-3.28052	2.69594	-1.74819
C	-0.66614	6.46287	0.26295
F	-3.06308	1.65902	-2.57349
F	-4.07589	2.23259	-0.76246
F	-3.98854	3.61167	-2.41880
F	-1.84143	7.10599	0.21593
F	-0.21352	6.54228	1.52297
F	0.19706	7.15137	-0.50002
H	4.91459	1.36997	0.88368
H	5.07354	-0.11194	-1.77136
H	5.15856	1.64580	-1.54042
H	7.45812	0.60177	-1.73763
H	7.21584	1.42193	-0.19884
H	6.96946	-1.59186	-0.68756
H	8.27472	-0.79059	0.19035
H	6.65516	-0.03569	1.92970
H	6.62170	-1.79676	1.73391
H	4.66669	-1.60390	0.19809
H	0.96363	1.00640	-0.63887
H	2.65093	-0.02021	-0.42068
H	3.12380	-0.18200	3.77503
H	4.80703	0.32663	3.44950
H	3.45393	0.99029	2.47950
H	3.42322	-2.55280	3.37661
H	4.50367	-3.06075	2.05782
H	5.15286	-2.10285	3.43969
H	3.04981	-1.19344	1.62569
H	-2.82082	5.23387	-0.86943
H	1.23137	4.60593	0.43093
H	-0.94565	1.41070	-1.43921

TS31

S	-0.21569	-1.13864	-1.44428
O	-1.06902	0.04464	-1.58486
C	-0.74674	-2.22387	-2.87717
F	-0.61715	-3.52468	-2.62857
F	0.03391	-1.90722	-3.89952
F	-2.00688	-1.97125	-3.18109
C	-2.08336	-2.26510	0.12367
C	-2.74845	-3.33950	-0.47711
C	-2.85165	-1.30893	0.80500
C	-4.13195	-3.46378	-0.39066
C	-4.23159	-1.42108	0.90445
C	-4.86027	-2.50586	0.30279
Br	-6.74060	-2.67427	0.41895
C	2.76825	-2.98221	-0.36023
N	3.86479	-2.71656	-1.26019
C	3.81182	-2.57520	-2.61337
C	5.09270	-2.31613	-3.03688
C	5.85932	-2.32092	-1.84856
N	5.11293	-2.55229	-0.77899
C	0.56885	-3.82220	-0.06042
H	-2.19549	-4.08973	-1.02963
H	-2.36849	-0.45342	1.27003
H	-4.63967	-4.29769	-0.86133
H	-4.80683	-0.66296	1.42262
H	2.87005	-2.61563	-3.13884
H	5.42581	-2.13504	-4.04647
H	6.92382	-2.16265	-1.73383
H	-0.24317	-4.48661	-0.33652
H	0.77269	-3.76143	1.00339
C	1.69161	-3.72993	-0.88048
O	2.84007	-2.51571	0.78144
H	1.68752	-4.06459	-1.90775
C	-0.59344	-2.07542	0.02044
C	0.03651	-1.36420	1.15675
O	0.71252	-0.34306	1.04310
O	1.22586	-0.95484	-1.63547
O	-0.09184	-1.85025	2.39581
C	-0.88829	-2.98604	2.78700
C	-0.12779	-3.70310	3.88161
H	-1.07824	-3.64337	1.93836
H	-1.85010	-2.60340	3.14076
H	-0.71223	-4.55174	4.24393

H	0.82921	-4.07268	3.50310
H	0.06272	-3.02919	4.72006
N	3.25993	-0.20529	2.38967
C	3.42587	-1.37578	3.30008
C	4.43347	-0.03365	1.44549
C	4.24334	1.18930	0.53889
C	5.32169	1.17758	-0.54820
C	6.71400	1.19890	0.08611
C	6.88955	0.05797	1.08979
C	5.78230	0.06044	2.15352
N	2.89997	1.24859	-0.01021
C	2.13171	2.36338	-0.00436
N	0.90462	2.17022	-0.56247
C	-0.22709	2.99618	-0.38039
S	2.62881	3.84080	0.64907
C	2.89295	1.00432	3.17569
C	-1.36186	2.41190	0.17680
C	-2.52429	3.15659	0.32970
C	-2.56874	4.49352	-0.04347
C	-1.42407	5.06666	-0.59041
C	-0.25746	4.33274	-0.77619
C	-3.74471	2.46918	0.86844
C	-1.43206	6.52299	-0.96023
F	-4.32679	1.68765	-0.05313
F	-3.43963	1.66059	1.90572
F	-4.66834	3.33741	1.29712
F	-0.96351	7.29052	0.03582
F	-0.66610	6.76608	-2.03272
F	-2.67046	6.95724	-1.23900
H	4.36714	2.10131	1.13115
H	5.19640	0.27921	-1.16703
H	5.16901	2.05157	-1.18855
H	7.48040	1.13568	-0.69235
H	6.85801	2.15956	0.59748
H	6.85080	-0.90309	0.56311
H	7.86546	0.12553	1.57898
H	5.84053	0.97931	2.75219
H	5.92610	-0.78953	2.82640
H	4.41121	-0.95100	0.84847
H	0.66161	1.20554	-0.76345
H	2.52070	0.40913	-0.45093
H	2.01433	0.75576	3.77125
H	3.72534	1.26736	3.82935

H	2.66056	1.83451	2.50998
H	2.43438	-1.64697	3.66508
H	3.84475	-2.20254	2.73207
H	4.06989	-1.08805	4.13094
H	2.44271	-0.41987	1.78708
H	-3.47444	5.07525	0.08170
H	0.61772	4.78589	-1.22478
H	-1.32167	1.37177	0.47909

TS32

C	3.27195	-1.03023	0.24030
C	4.02869	-2.18094	-0.01670
C	3.90317	0.21784	0.11963
C	5.37661	-2.09813	-0.34966
C	5.25069	0.31208	-0.20067
C	5.97873	-0.85050	-0.42653
Br	7.81681	-0.73126	-0.86396
C	-1.10560	-2.56075	2.19859
N	-1.11229	-1.78440	3.39883
C	-2.16992	-1.04196	3.82724
C	-1.78454	-0.44033	4.99792
C	-0.45627	-0.88855	5.19924
N	-0.05591	-1.69412	4.23011
C	1.39599	-2.68122	1.85149
H	3.55927	-3.15779	0.00421
H	3.33737	1.12875	0.27520
H	5.95083	-2.99533	-0.54945
H	5.72874	1.28090	-0.28812
H	-3.08920	-1.02180	3.26454
H	-2.36951	0.21625	5.62216
H	0.21676	-0.65741	6.01412
H	1.67894	-2.10674	2.72621
H	2.17844	-3.32277	1.45980
C	0.08117	-3.13693	1.70728
O	-2.22593	-2.71501	1.66019
H	-0.10826	-3.83520	0.90012
C	1.80800	-1.13502	0.54518
O	-0.51219	-1.36412	-0.82797
O	-0.08917	0.01187	1.45686
N	-2.47442	-3.40365	-1.18174
C	-1.80366	-4.71946	-1.00413
C	-3.90395	-3.40774	-0.68029

C	-4.49459	-1.99641	-0.76730
C	-5.86048	-1.97741	-0.07456
C	-6.80182	-2.99001	-0.72838
C	-6.19253	-4.39294	-0.72700
C	-4.80787	-4.41046	-1.39042
N	-3.55984	-1.03883	-0.21141
C	-3.37603	0.21329	-0.68918
N	-2.34946	0.85418	-0.06534
C	-1.91918	2.17551	-0.26647
S	-4.29472	0.86643	-1.94327
C	-2.30656	-2.89833	-2.57350
C	-0.55032	2.39741	-0.37421
C	-0.05199	3.69533	-0.43648
C	-0.90398	4.78717	-0.43986
C	-2.27795	4.54984	-0.36740
C	-2.79526	3.26729	-0.26767
C	1.43832	3.85872	-0.48990
C	-3.19463	5.74059	-0.36673
F	1.81517	5.14006	-0.46998
F	2.02650	3.25007	0.56600
F	1.96663	3.29308	-1.58446
F	-4.48145	5.39435	-0.24907
F	-2.90666	6.57369	0.64746
F	-3.06811	6.45570	-1.49501
H	-4.65030	-1.72442	-1.81705
H	-5.72243	-2.21140	0.98951
H	-6.26006	-0.96194	-0.14501
H	-7.76499	-2.99744	-0.21052
H	-6.99908	-2.67922	-1.76246
H	-6.09424	-4.74761	0.30678
H	-6.84851	-5.09834	-1.24441
H	-4.91006	-4.14678	-2.45026
H	-4.38904	-5.41944	-1.33473
H	-3.80064	-3.68413	0.37394
H	-1.65073	0.26225	0.39150
H	-3.05841	-1.33152	0.63119
H	-1.24935	-2.96372	-2.82796
H	-2.89807	-3.51769	-3.24775
H	-2.61908	-1.85501	-2.62190
H	-0.73495	-4.57249	-1.17340
H	-1.98301	-5.05656	0.01744
H	-2.20357	-5.43081	-1.72593
H	-1.96886	-2.74739	-0.56006

H	-0.51571	5.79831	-0.49189
H	-3.86268	3.10913	-0.18404
H	0.12234	1.54995	-0.40164
C	1.12199	-0.11921	1.35948
C	1.34515	1.52298	3.02299
C	2.46522	2.26464	3.71524
H	0.71379	2.19245	2.43247
H	0.70486	0.97375	3.71826
H	2.04901	2.98989	4.41821
H	3.10466	1.57193	4.26690
H	3.07542	2.80046	2.98448
O	1.96496	0.57461	2.13023
S	0.91926	-1.69567	-0.86430
O	1.28742	-3.07143	-1.20810
C	1.55223	-0.68504	-2.29594
F	2.75093	-1.08822	-2.67909
F	0.69582	-0.83357	-3.29819
F	1.60642	0.59756	-1.94802

TS33

S	1.07875	0.34046	0.69049
O	1.14731	1.09334	-0.55892
C	2.47861	1.01445	1.70824
F	3.58364	1.16733	0.99914
F	2.72704	0.21843	2.75000
F	2.07850	2.19706	2.15524
C	2.91139	-1.53809	0.03070
C	3.93019	-1.86126	0.93215
C	3.24850	-1.32999	-1.31403
C	5.24723	-1.99906	0.50625
C	4.55837	-1.46899	-1.75527
C	5.54669	-1.80691	-0.83688
Br	7.33194	-1.99283	-1.43233
C	-1.59087	-2.46890	2.17887
N	-1.78488	-1.27590	2.97260
C	-1.21185	-0.93075	4.15339
C	-1.74602	0.28080	4.52307
C	-2.64946	0.59322	3.48561
N	-2.67589	-0.35752	2.56294
C	0.90179	-2.30885	2.27650
H	3.70510	-1.99618	1.98425
H	2.47221	-1.04697	-2.01978

H	6.03331	-2.25050	1.20887
H	4.81311	-1.30661	-2.79621
H	-0.50867	-1.58954	4.63905
H	-1.52810	0.85037	5.41225
H	-3.27995	1.46549	3.37506
H	0.89926	-1.37412	2.82410
H	1.82432	-2.87529	2.36667
C	-0.28944	-3.03155	2.14790
O	-2.59547	-2.91248	1.61713
H	-0.26221	-4.02350	1.70427
C	1.48500	-1.38453	0.47190
C	0.41687	-1.96162	-0.34960
O	-0.68057	-1.43470	-0.47645
O	-0.06477	0.50105	1.59228
O	0.56417	-3.18569	-0.89358
C	1.62613	-4.10195	-0.56868
C	1.09170	-5.50025	-0.79371
H	1.93301	-3.95994	0.46988
H	2.48489	-3.89566	-1.21257
H	1.88620	-6.23005	-0.62289
H	0.26921	-5.71849	-0.10732
H	0.73547	-5.61344	-1.82079
N	-2.99243	-2.96479	-1.16041
C	-2.57122	-4.38080	-0.99070
C	-4.41263	-2.72308	-0.70208
C	-4.75683	-1.23507	-0.81479
C	-6.11780	-0.97928	-0.16051
C	-7.19749	-1.82816	-0.83474
C	-6.82993	-3.31315	-0.81454
C	-5.45042	-3.56330	-1.43999
N	-3.69127	-0.42816	-0.25690
C	-3.27149	0.73826	-0.81325
N	-2.28945	1.33232	-0.09058
C	-1.56425	2.51407	-0.32781
S	-3.93642	1.35654	-2.24172
C	-2.67607	-2.47618	-2.53023
C	-1.02280	3.13859	0.80400
C	-0.18128	4.22795	0.66103
C	0.11216	4.75256	-0.59552
C	-0.44864	4.13973	-1.70460
C	-1.27095	3.02058	-1.59162
C	0.44428	4.86871	1.86506
C	-0.18332	4.68712	-3.07764

F	0.01610	6.13143	2.03429
F	0.17632	4.20216	2.99605
F	1.78030	4.93442	1.74530
F	0.90341	5.47344	-3.10333
F	0.00599	3.70786	-3.97476
F	-1.21176	5.42602	-3.52489
H	-4.83922	-0.96366	-1.87164
H	-6.05183	-1.21794	0.90941
H	-6.34262	0.08751	-0.25022
H	-8.16235	-1.67191	-0.34392
H	-7.31243	-1.49650	-1.87483
H	-6.81758	-3.67135	0.22268
H	-7.58199	-3.90240	-1.34661
H	-5.47631	-3.28753	-2.50187
H	-5.20819	-4.62810	-1.37582
H	-4.38963	-3.00707	0.35424
H	-1.94664	0.80898	0.71625
H	-3.41180	-0.61370	0.71510
H	-2.86404	-1.40380	-2.58896
H	-1.61696	-2.66612	-2.70295
H	-3.28582	-3.01723	-3.25459
H	-1.48619	-4.40506	-1.09163
H	-2.85758	-4.70349	0.00885
H	-3.03913	-4.99234	-1.76200
H	-2.38663	-2.41621	-0.52296
H	0.77475	5.60407	-0.70365
H	-1.65169	2.53378	-2.47825
H	-1.21926	2.72814	1.78839

NMR Spectra

^{1}H NMR in $CDCl_3$ (400 MHz)

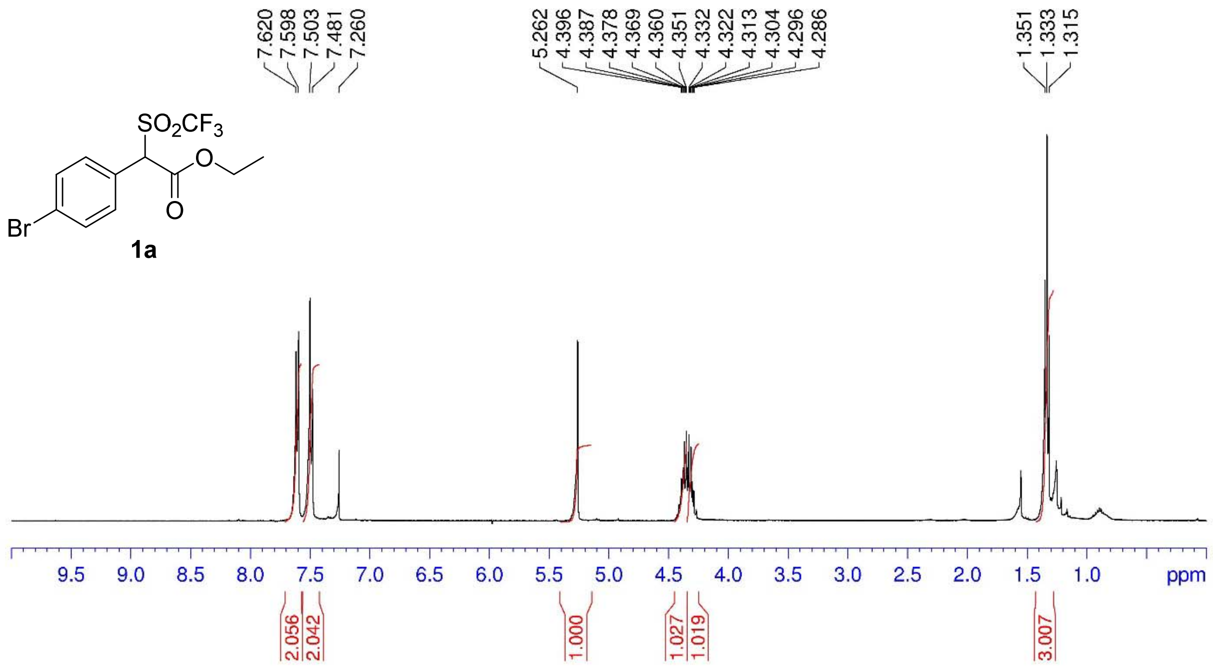

^{13}C NMR in $CDCl_3$ (100 MHz)

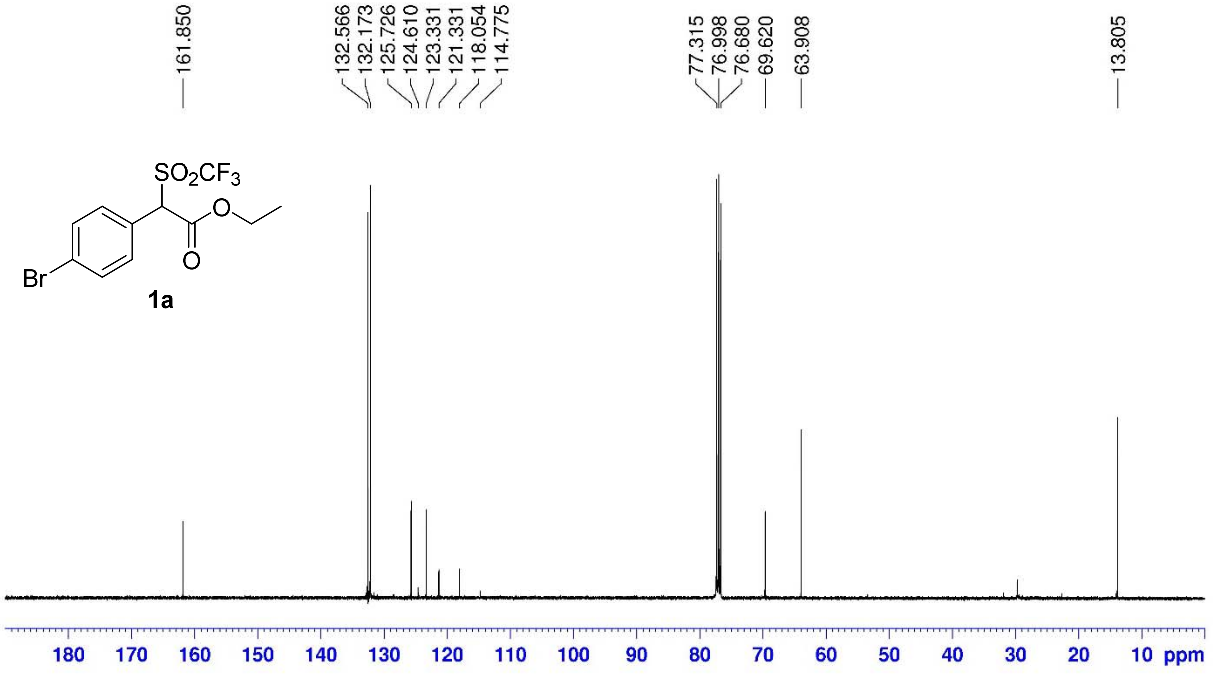

^{19}F NMR in $CDCl_3$ (376 MHz)

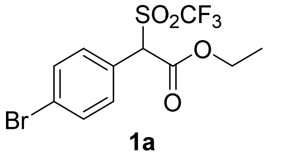

1a

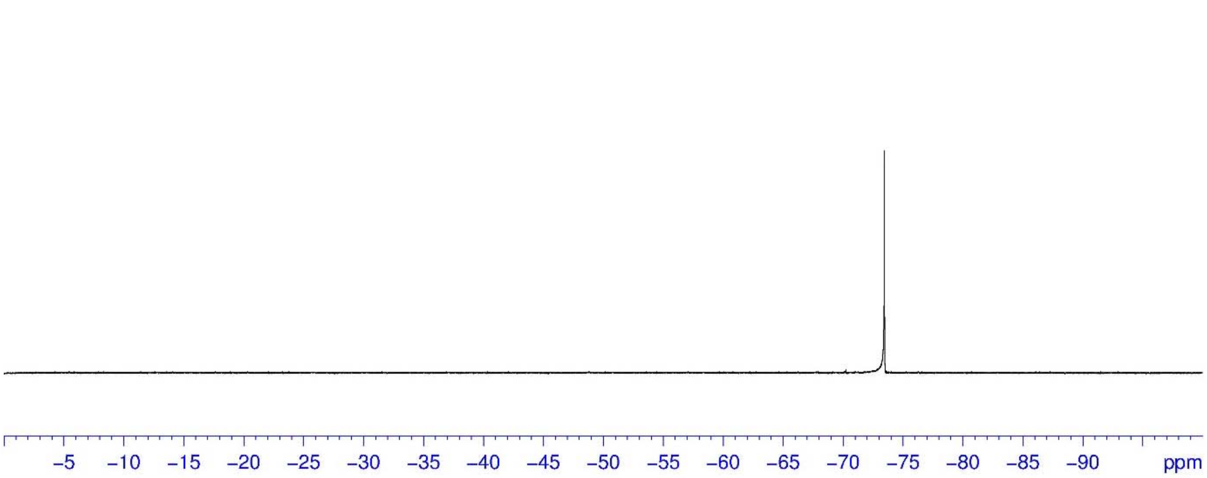

^1H NMR in $CDCl_3$ (600 MHz)

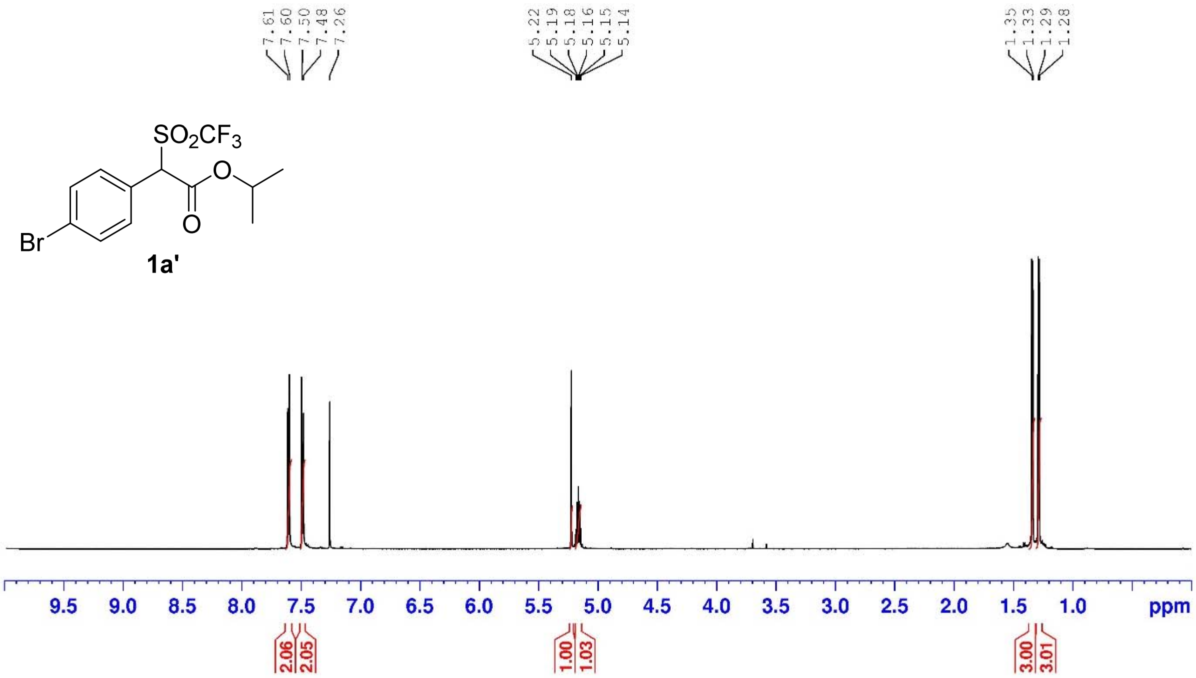

^{13}C NMR in $CDCl_3$ (150 MHz)

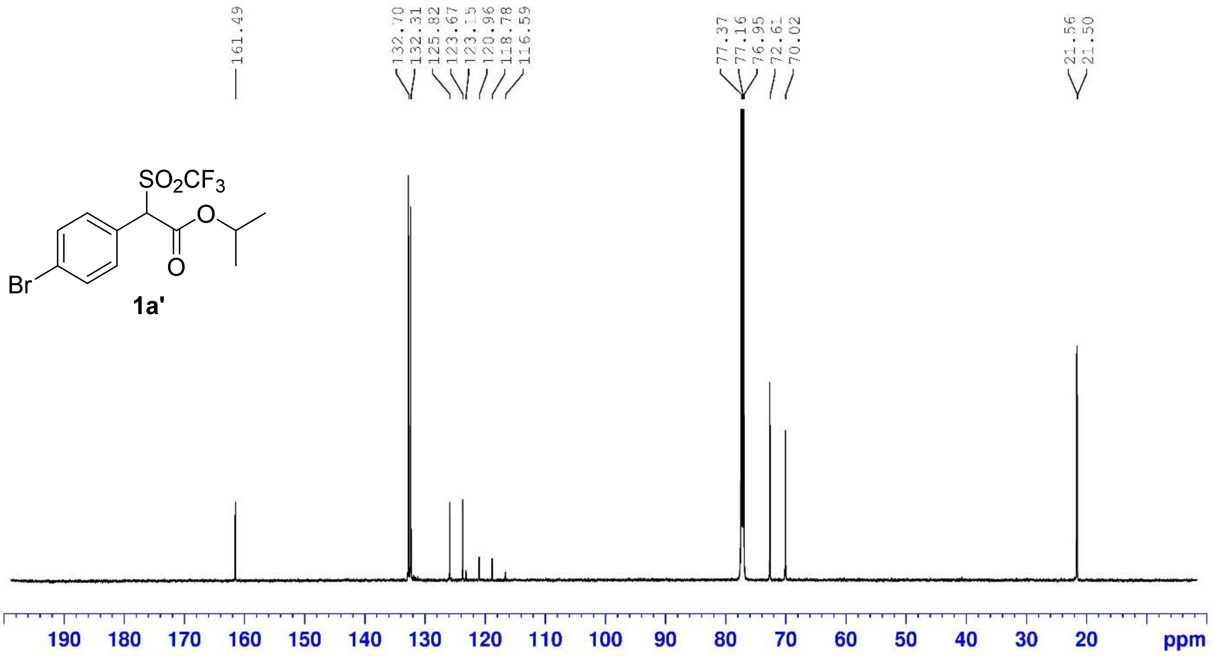

^{19}F NMR in $CDCl_3$ (376 MHz)

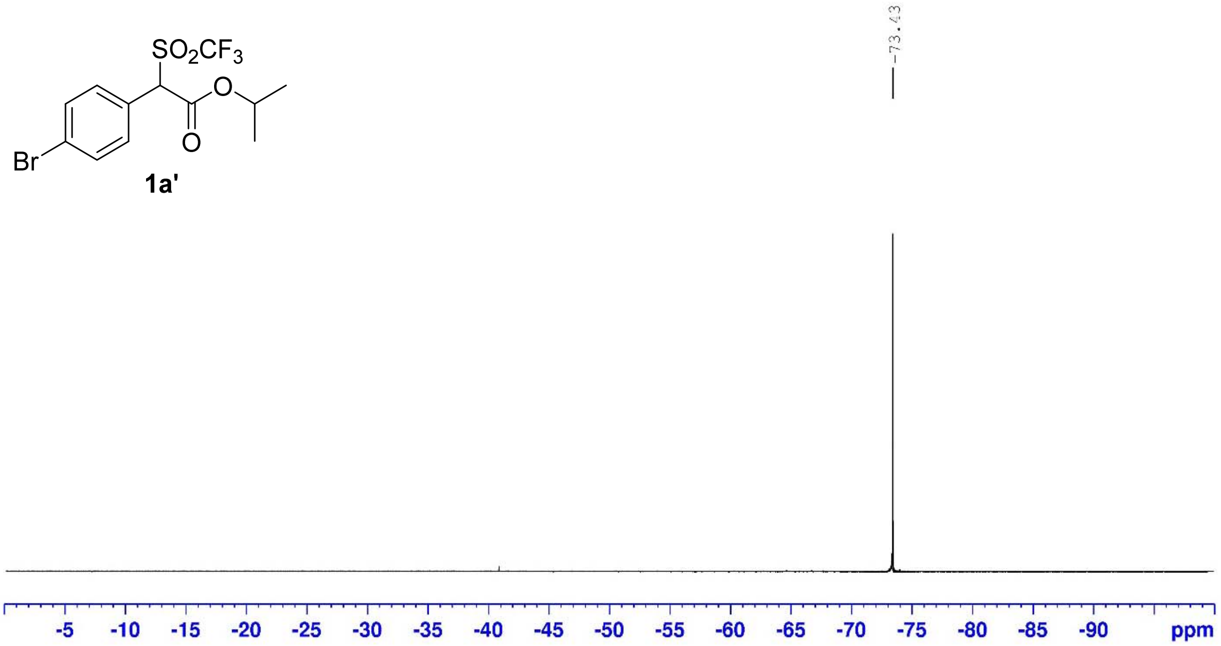

^{1}H NMR in $CDCl_3$ (300 MHz)

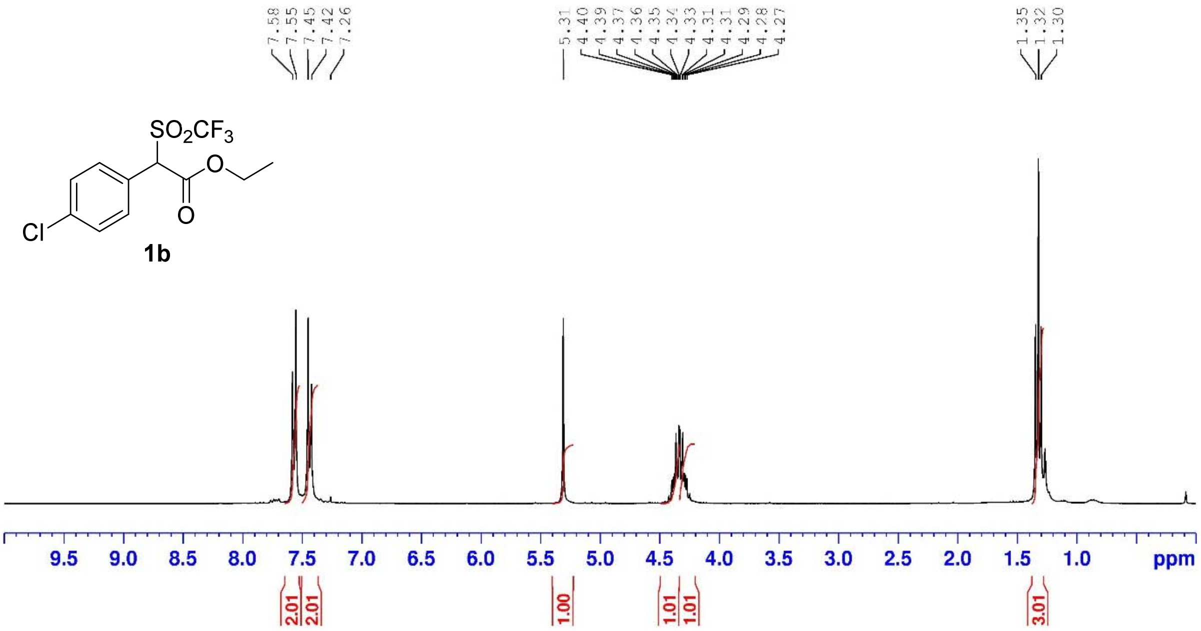

^{13}C NMR in $CDCl_3$ (75MHz)

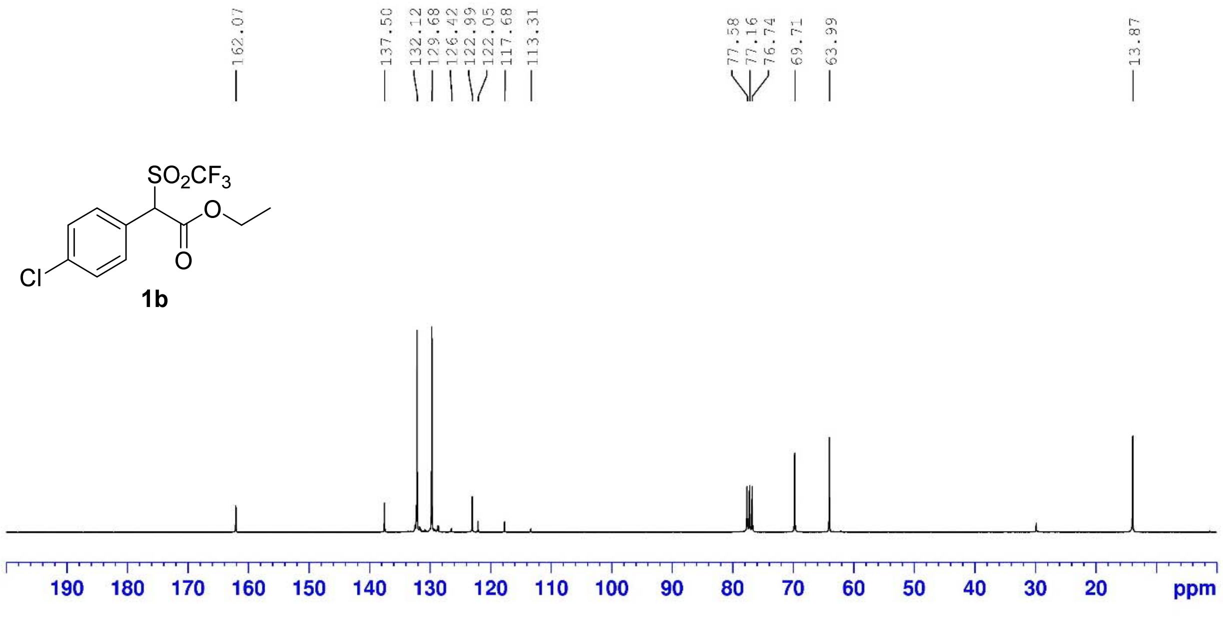

^{19}F NMR in $CDCl_3$ (282 MHz)

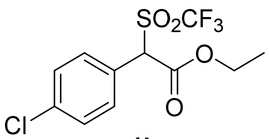

1b

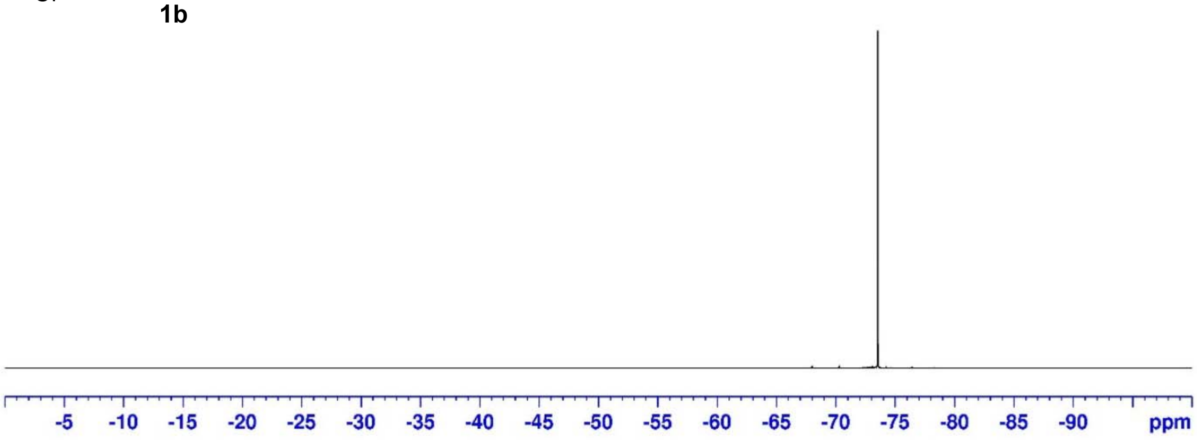

^{1}H NMR in $CDCl_3$ (300 MHz)

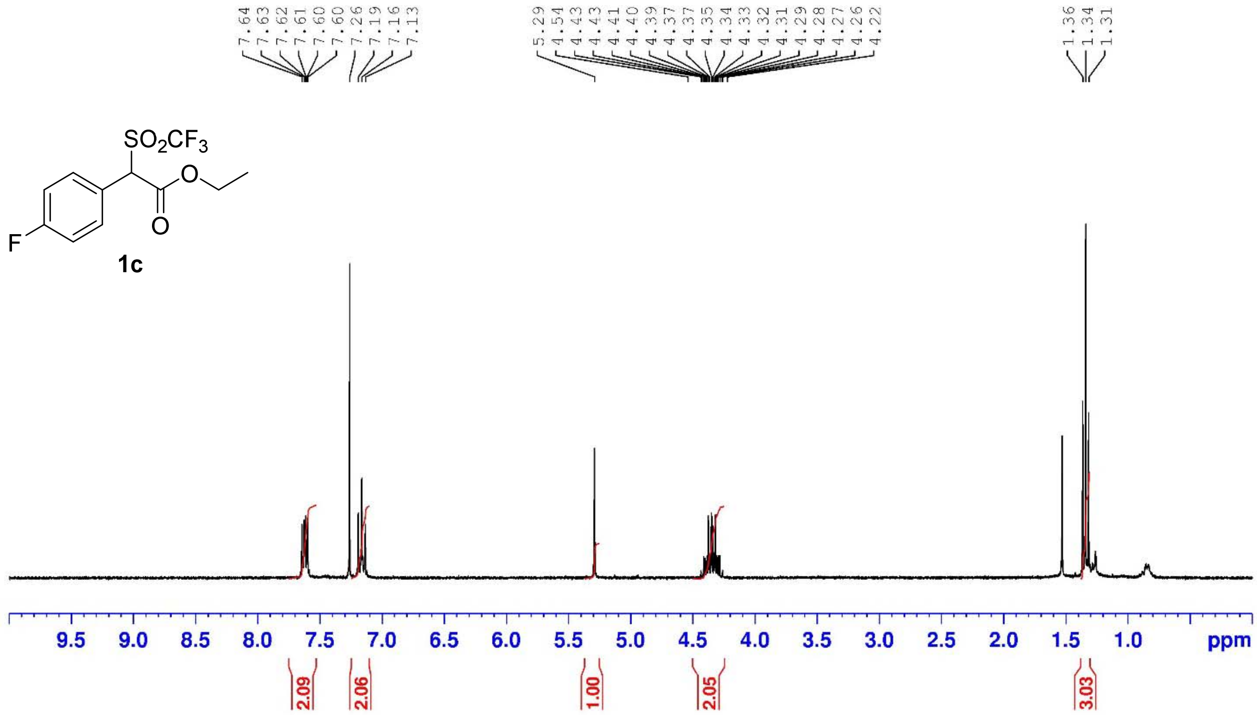

^{13}C NMR in $CDCl_3$ (62.5 MHz)

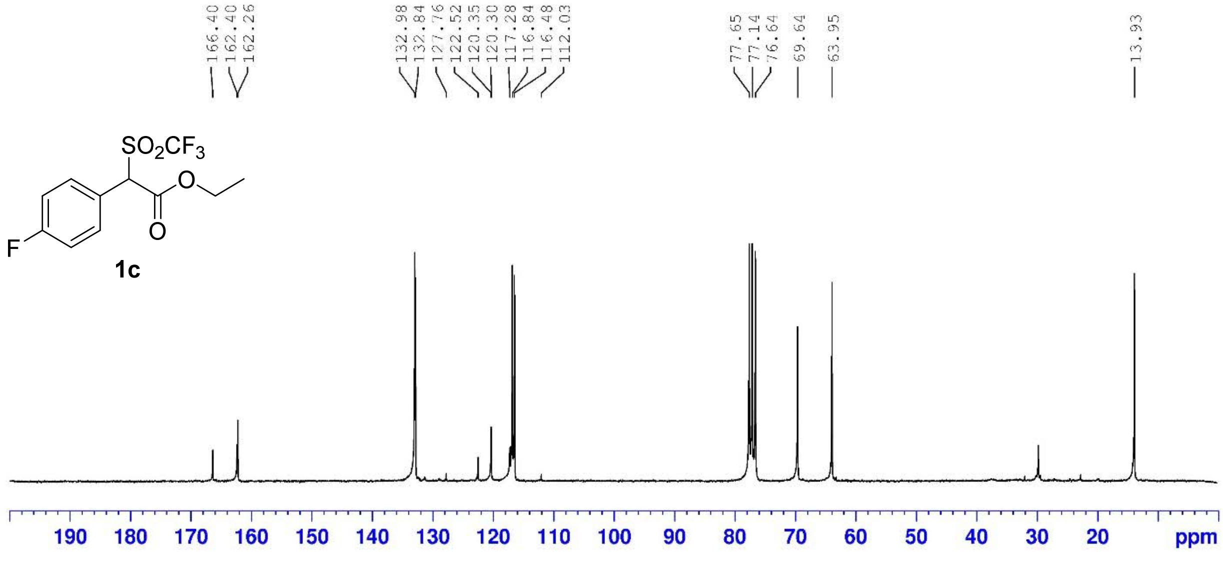

^{19}F NMR in $CDCl_3$ (282 MHz)

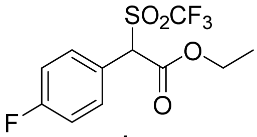

1c

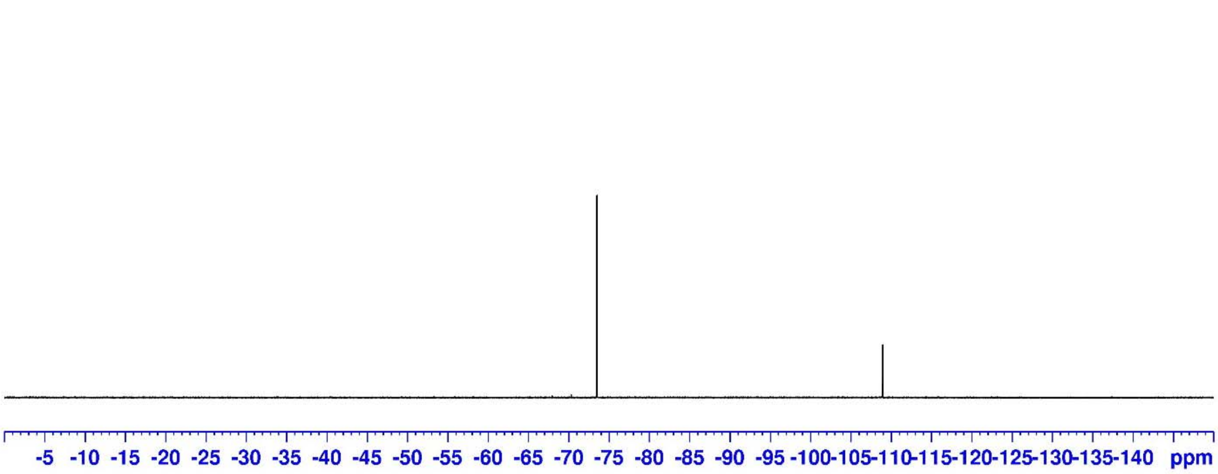

^{1}H NMR in $CDCl_3$ (300 MHz)

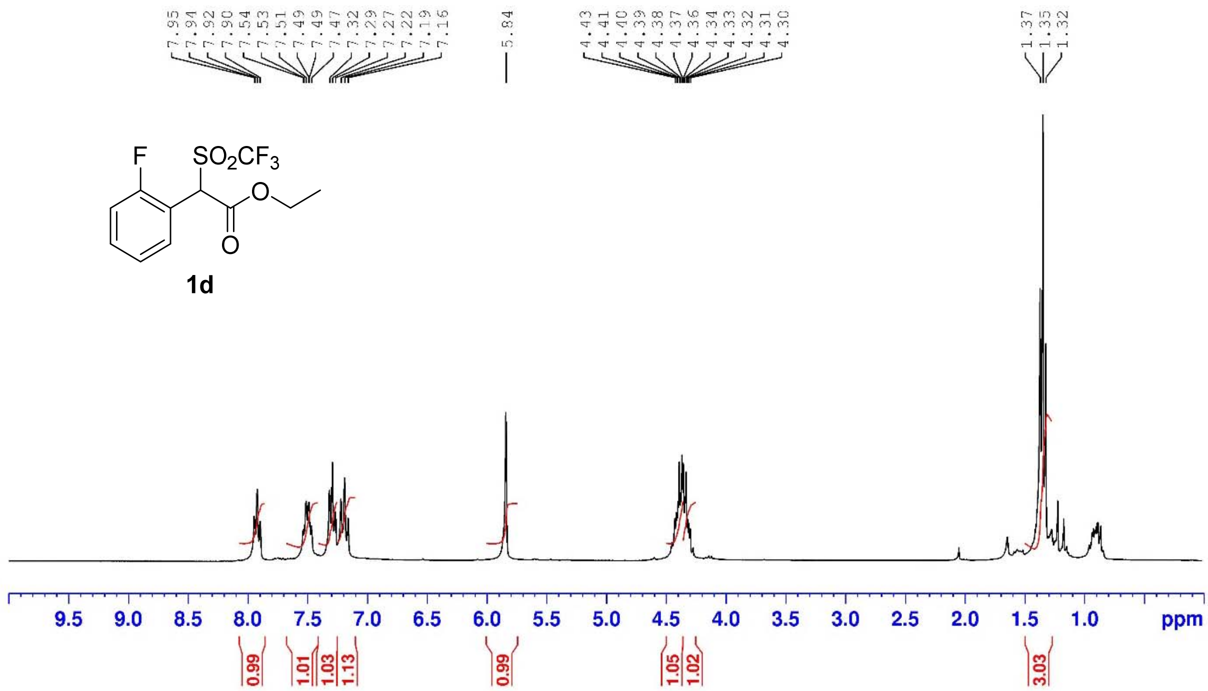

^{13}C NMR in $CDCl_3$ (62.5MHz)

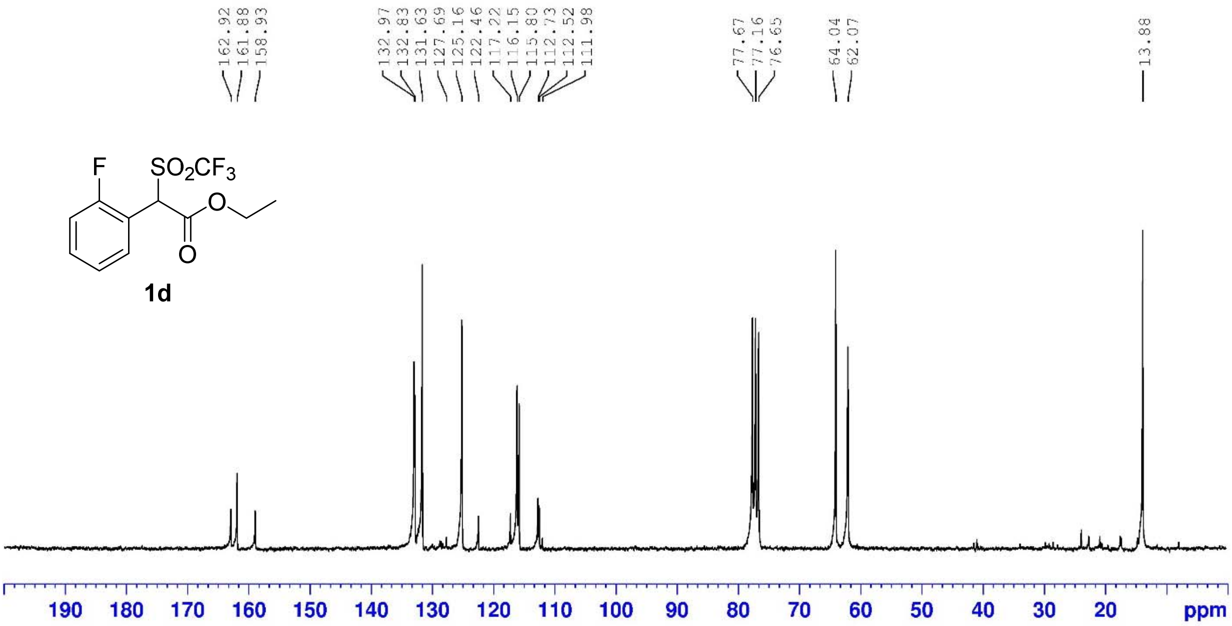

^{19}F NMR in $CDCl_3$ (376 MHz)

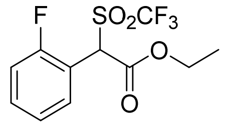

1d

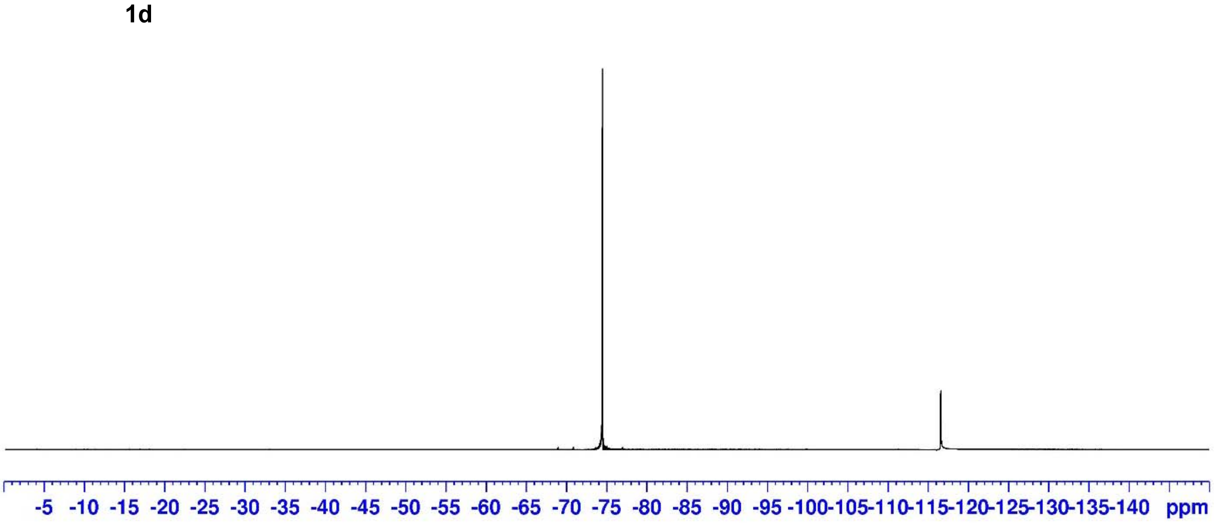

^{1}H NMR in $CDCl_3$ (300MHz)

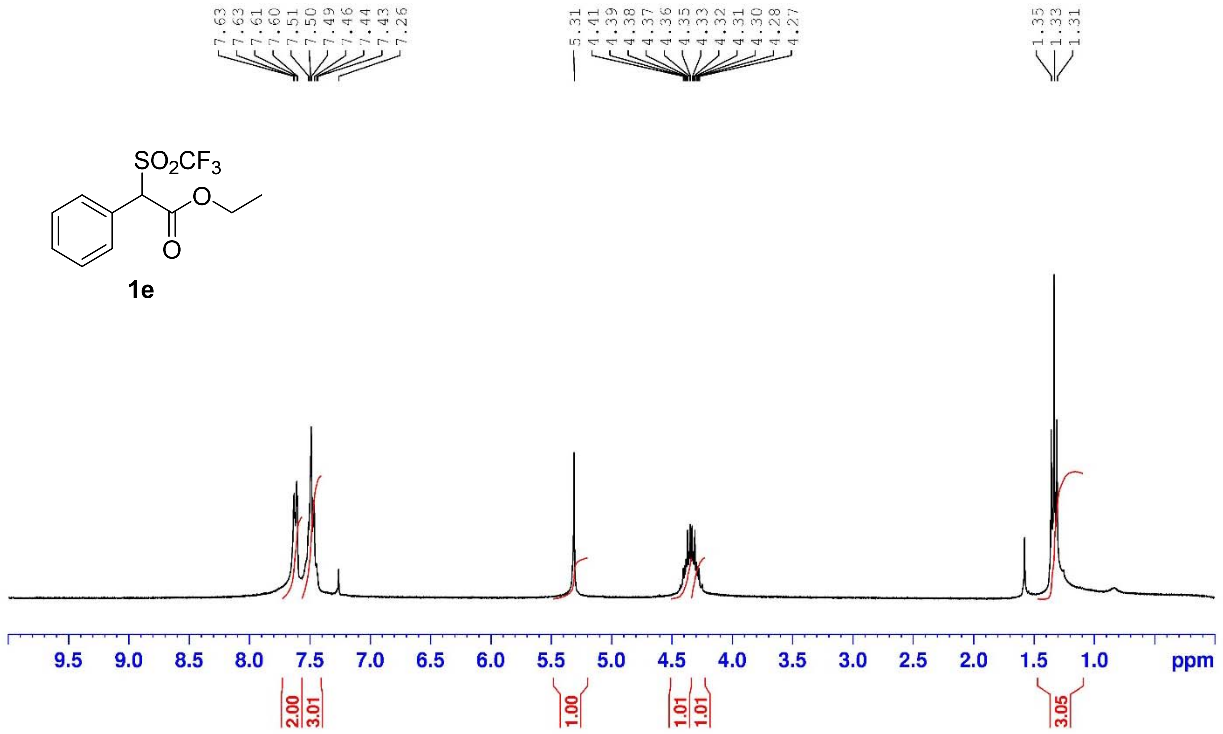

^{13}C NMR in $CDCl_3$ (75 MHz)

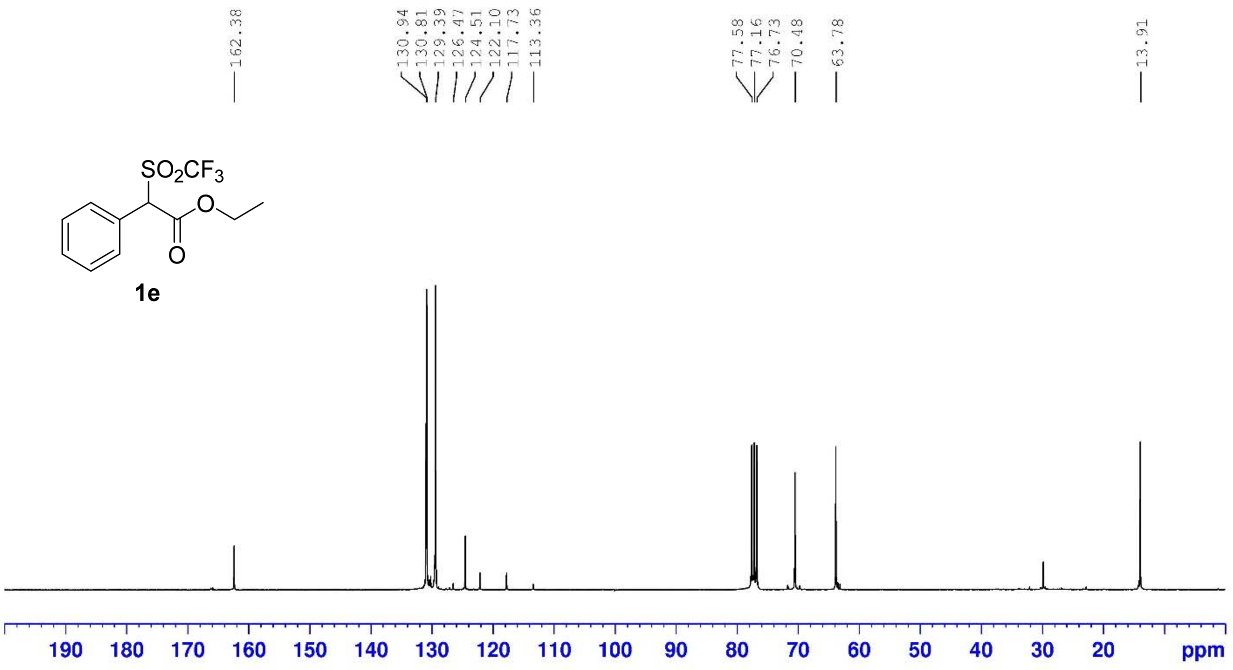

^{19}F NMR in $CDCl_3$ (282 MHz)

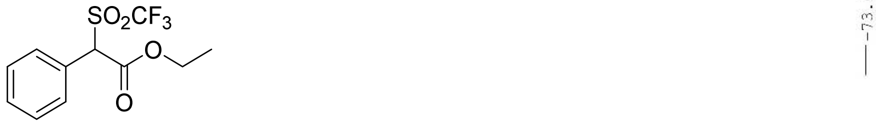

1e

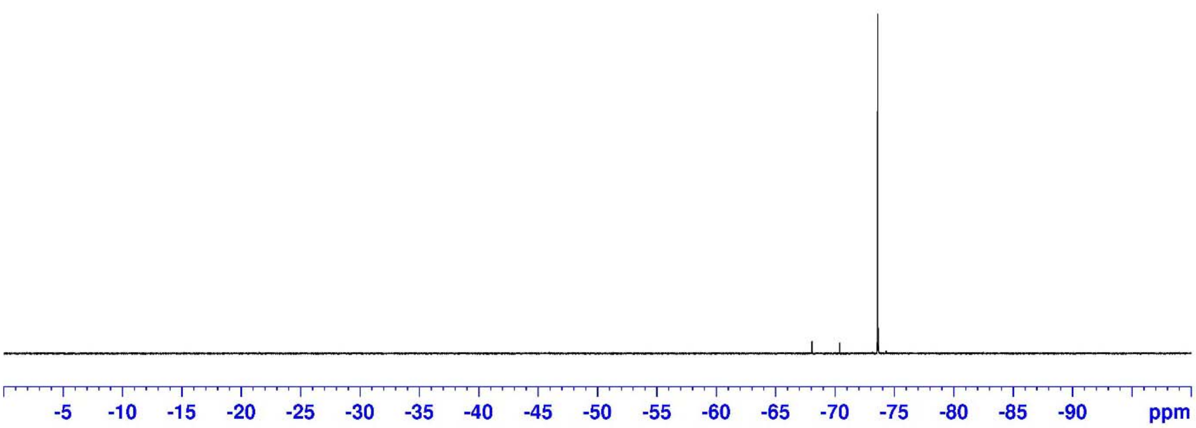

^{1}H NMR in $CDCl_3$ (300 MHz)

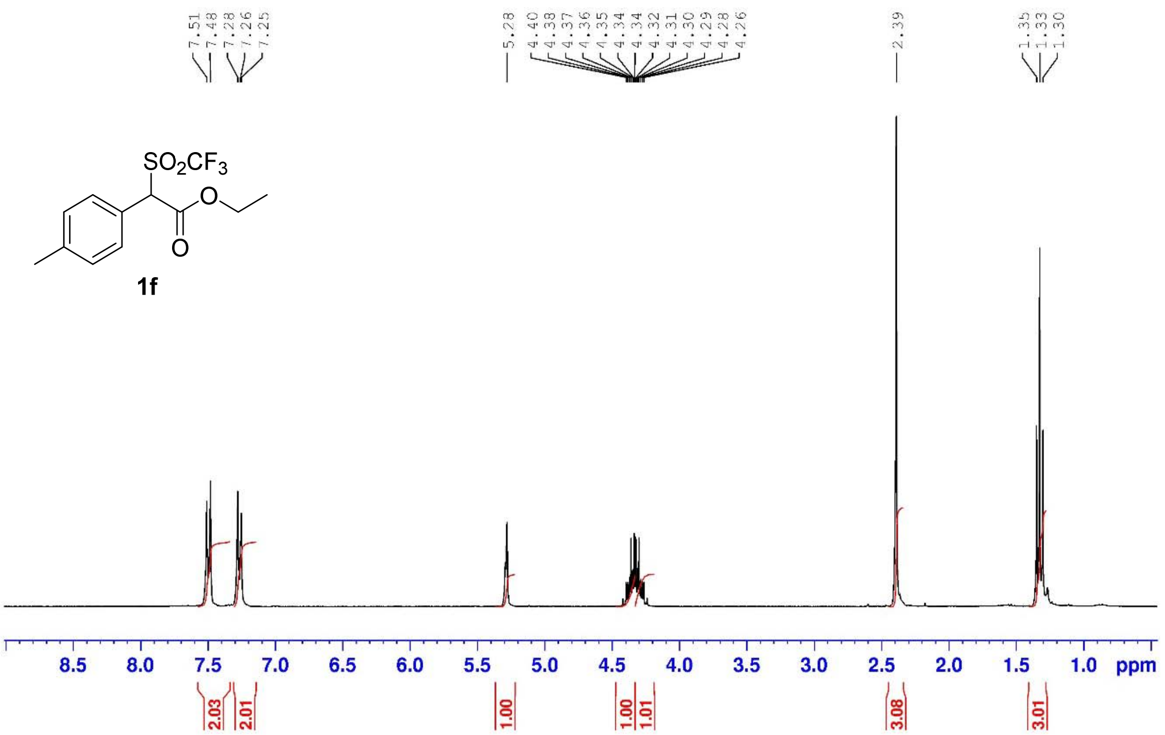

^{13}C NMR in $CDCl_3$ (75 MHz)

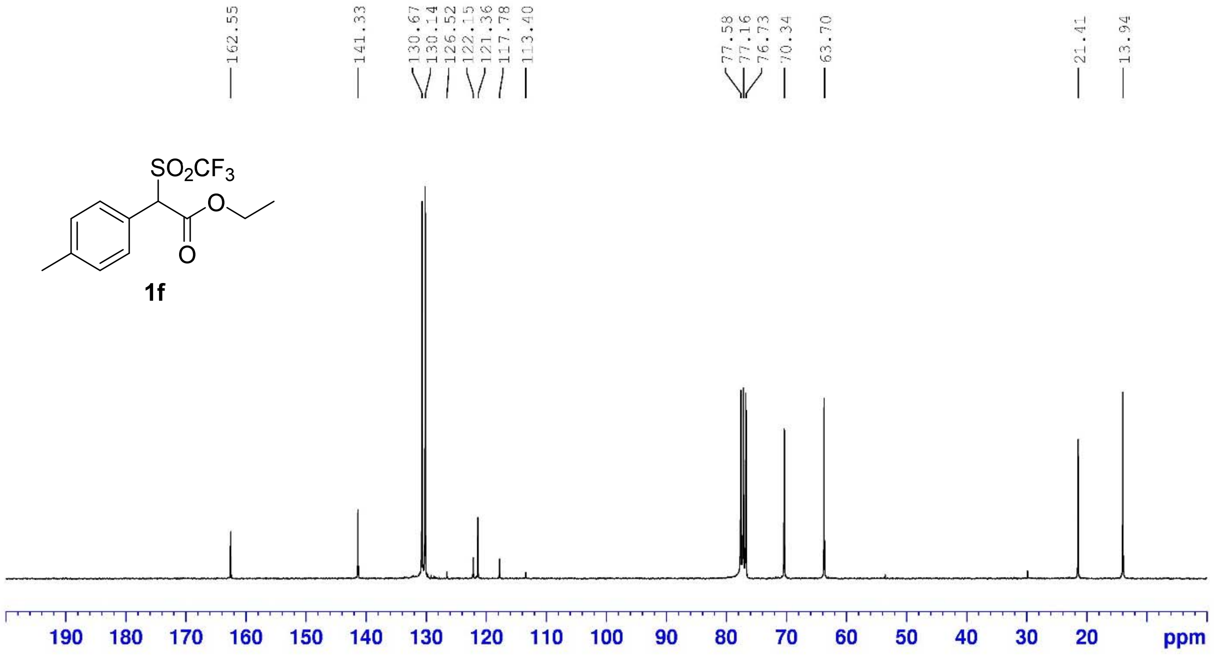

^{19}F NMR in $CDCl_3$ (282 MHz)

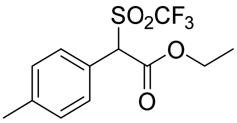

1f

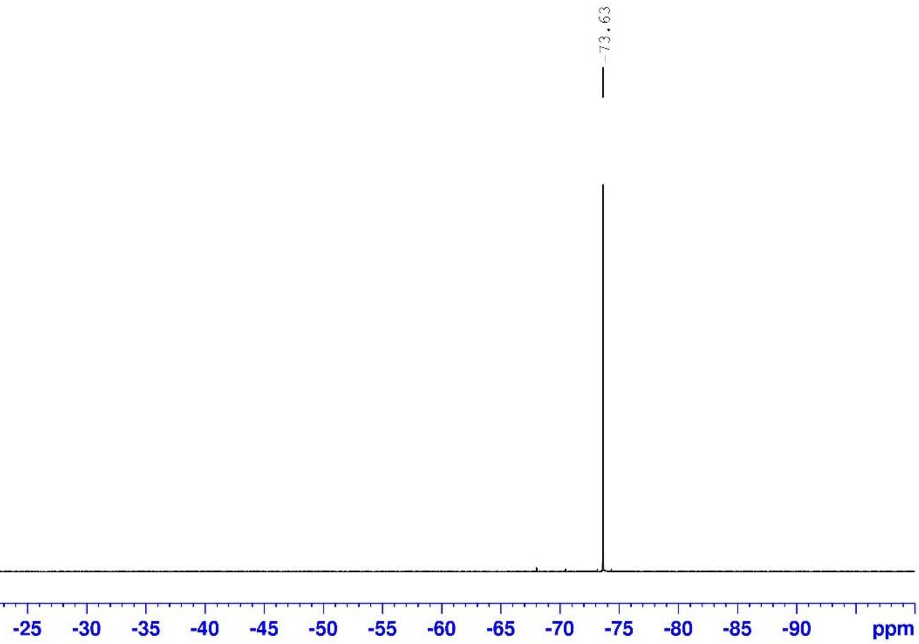

^{1}H NMR in $CDCl_3$ (300 MHz)

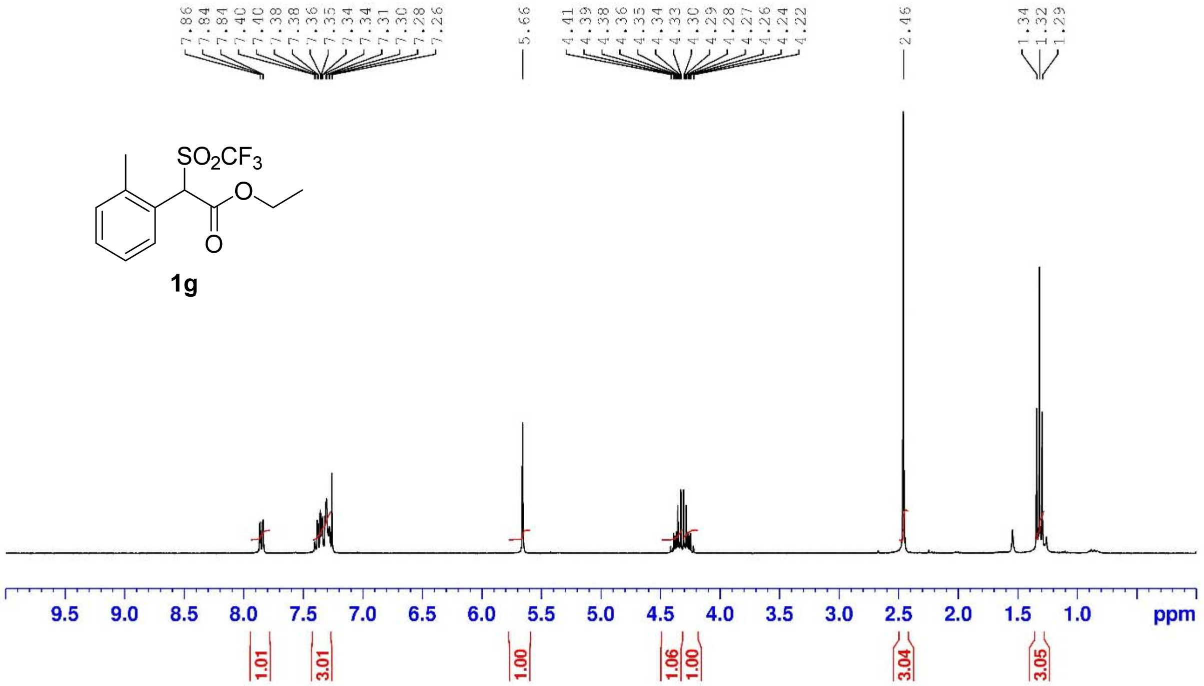

^{13}C NMR in $CDCl_3$ (75 MHz)

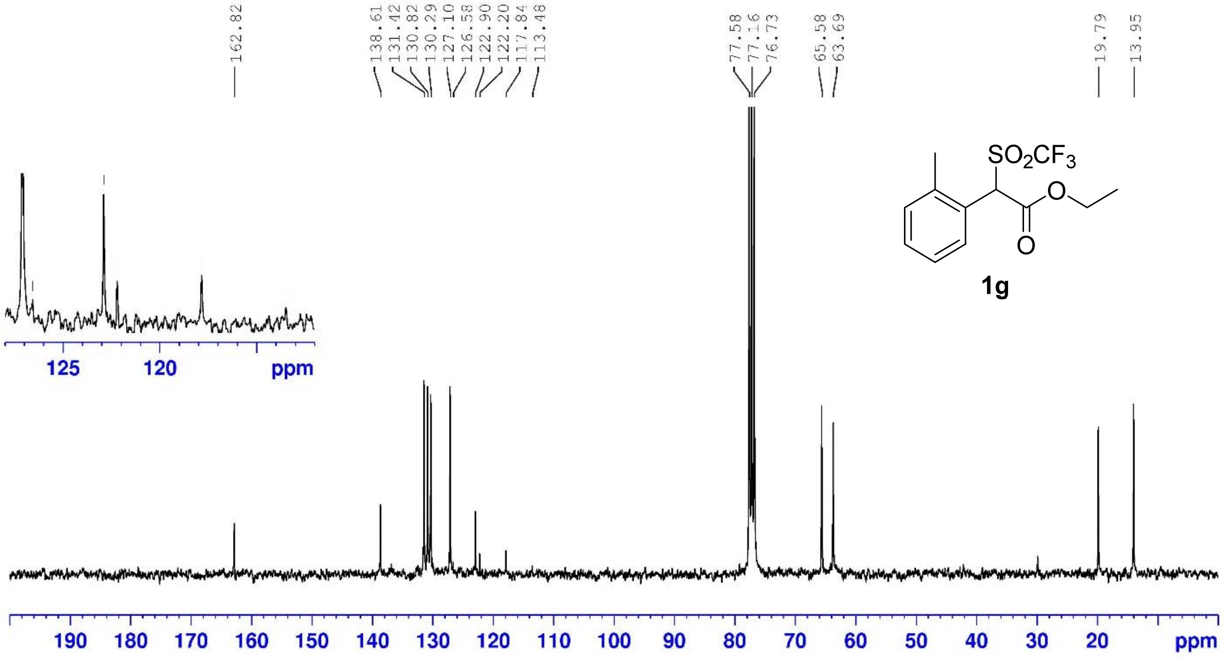

^{19}F NMR in $CDCl_3$ (376 MHz)

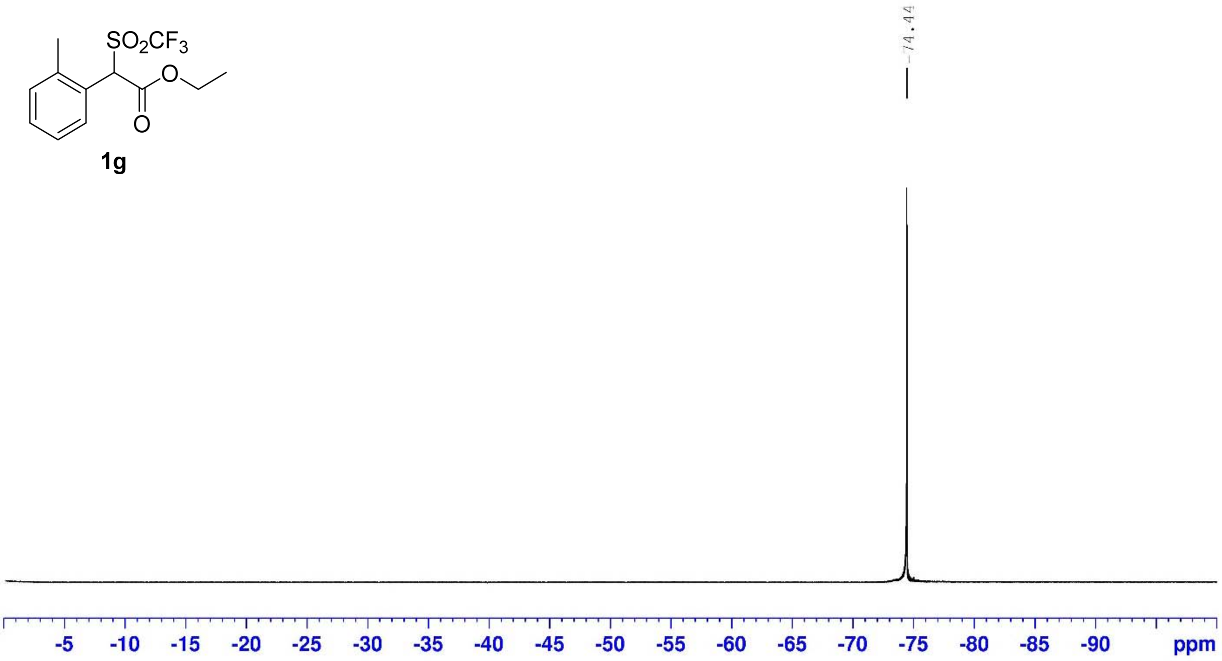

^{1}H NMR in $CDCl_3$ (400 MHz)

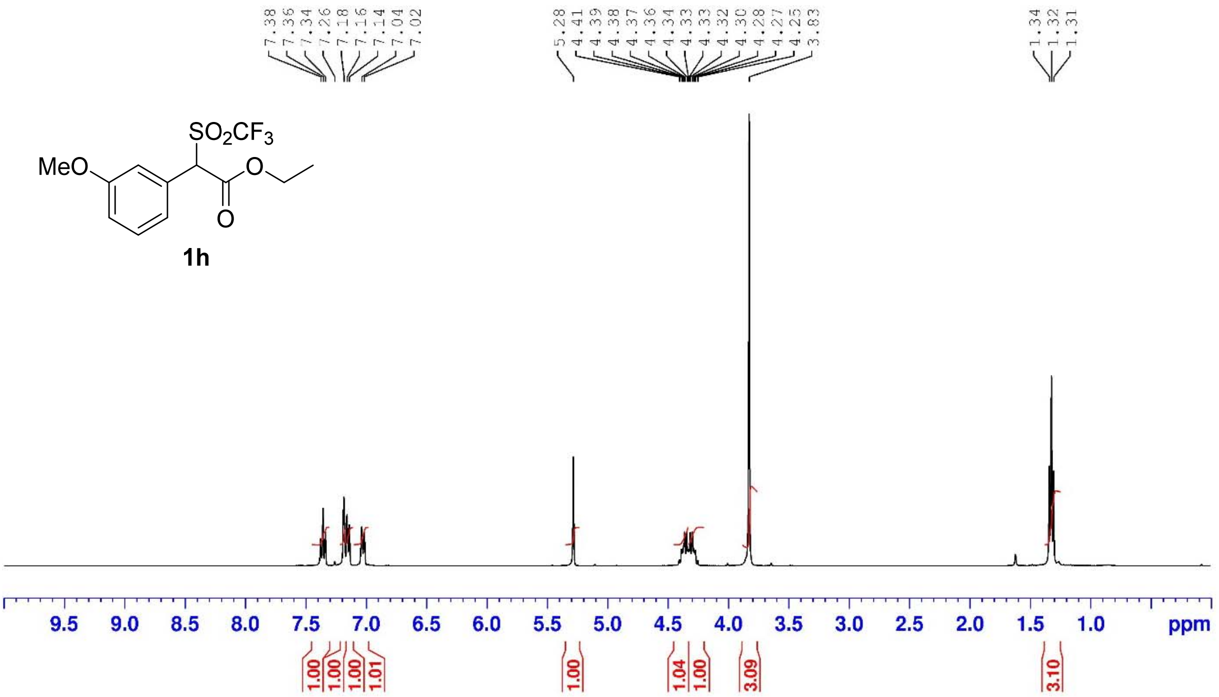

^{13}C NMR in $CDCl_3$ (100 MHz)

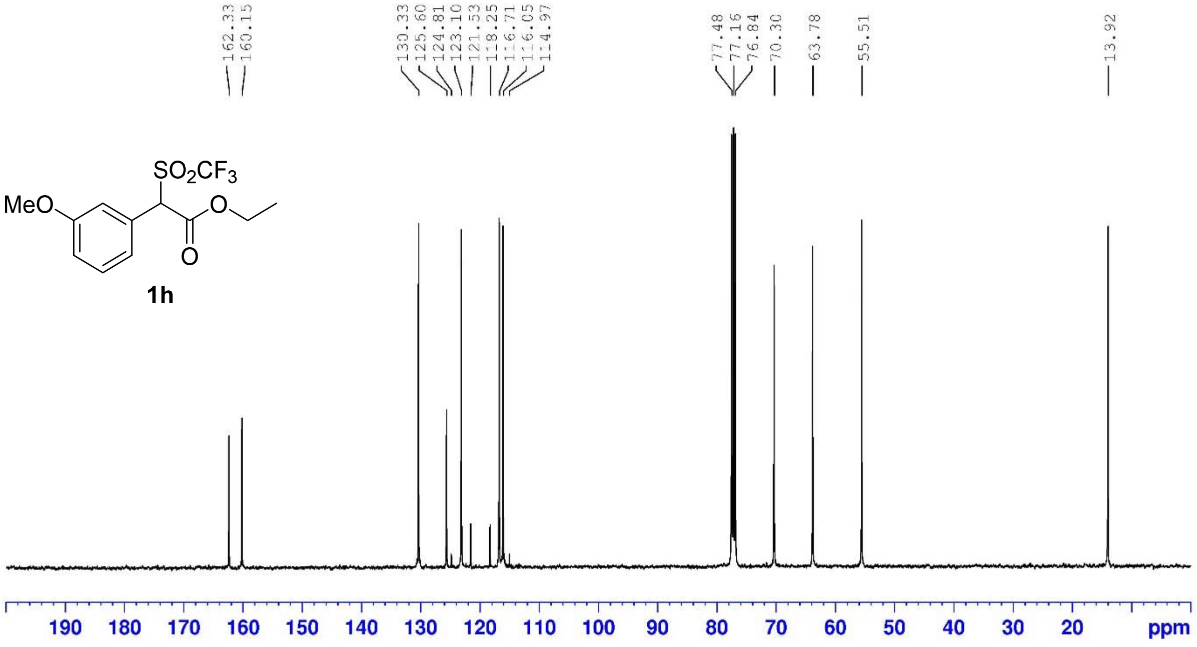

^{19}F NMR in $CDCl_3$ (376 MHz)

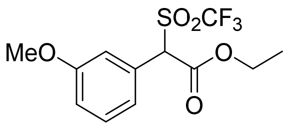

1h

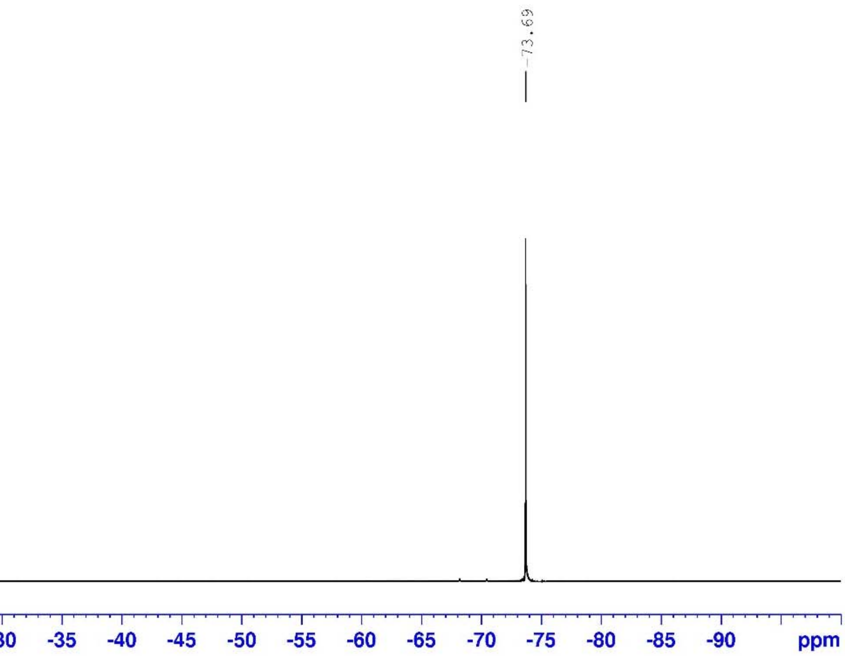

^{1}H NMR in $CDCl_3$ (300 MHz)

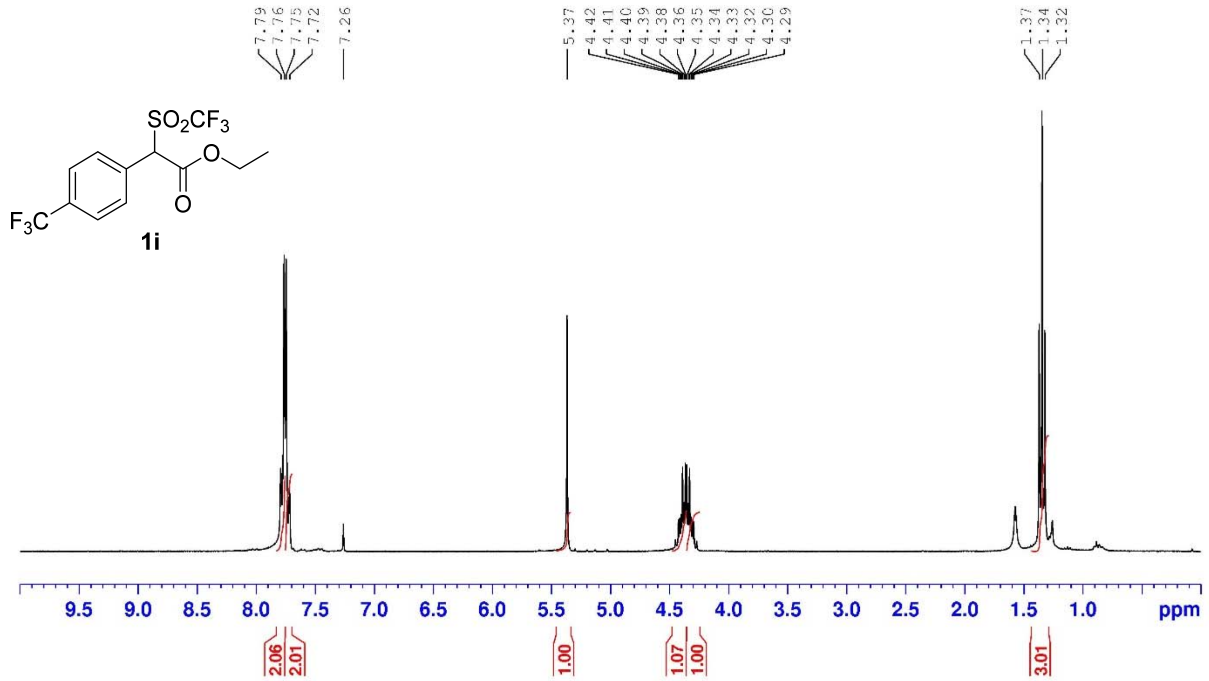

^{13}C NMR in $CDCl_3$ (75 MHz)

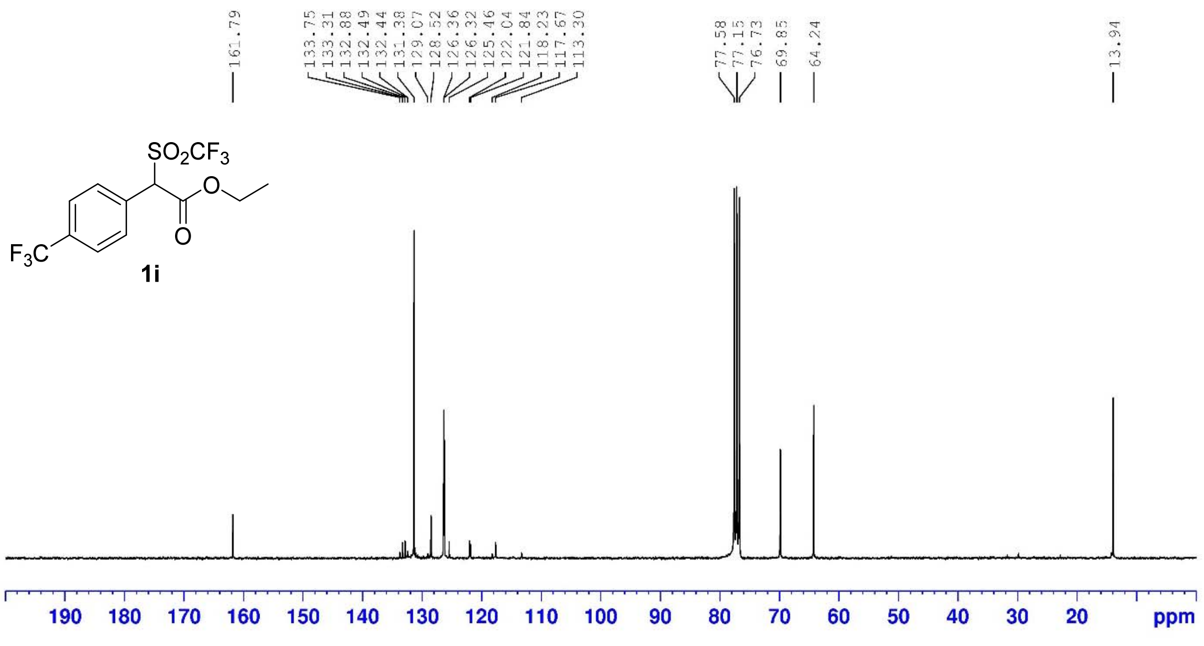

^{19}F NMR in $CDCl_3$ (376 MHz)

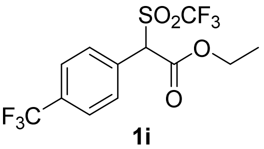

1i

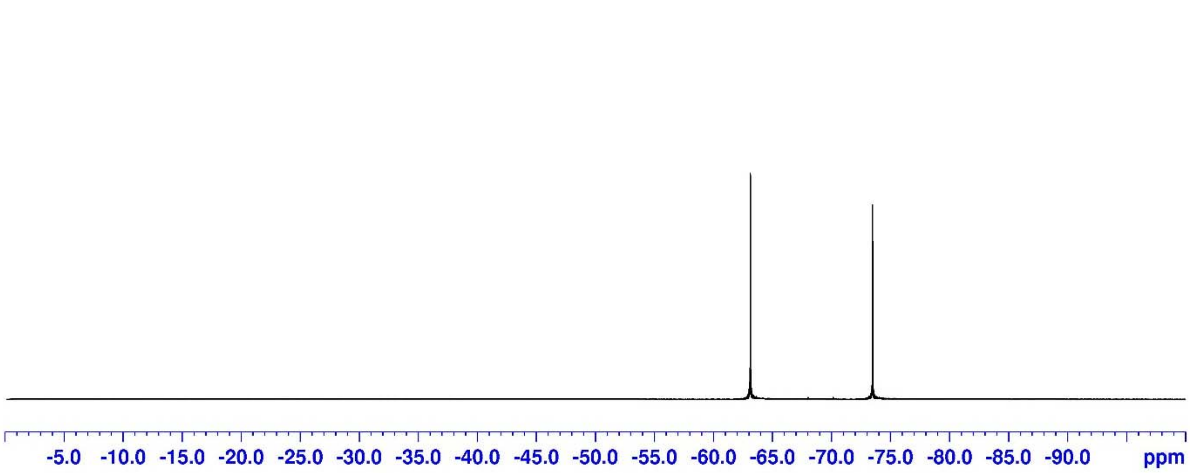

^{1}H NMR in $CDCl_3$ (400 MHz)

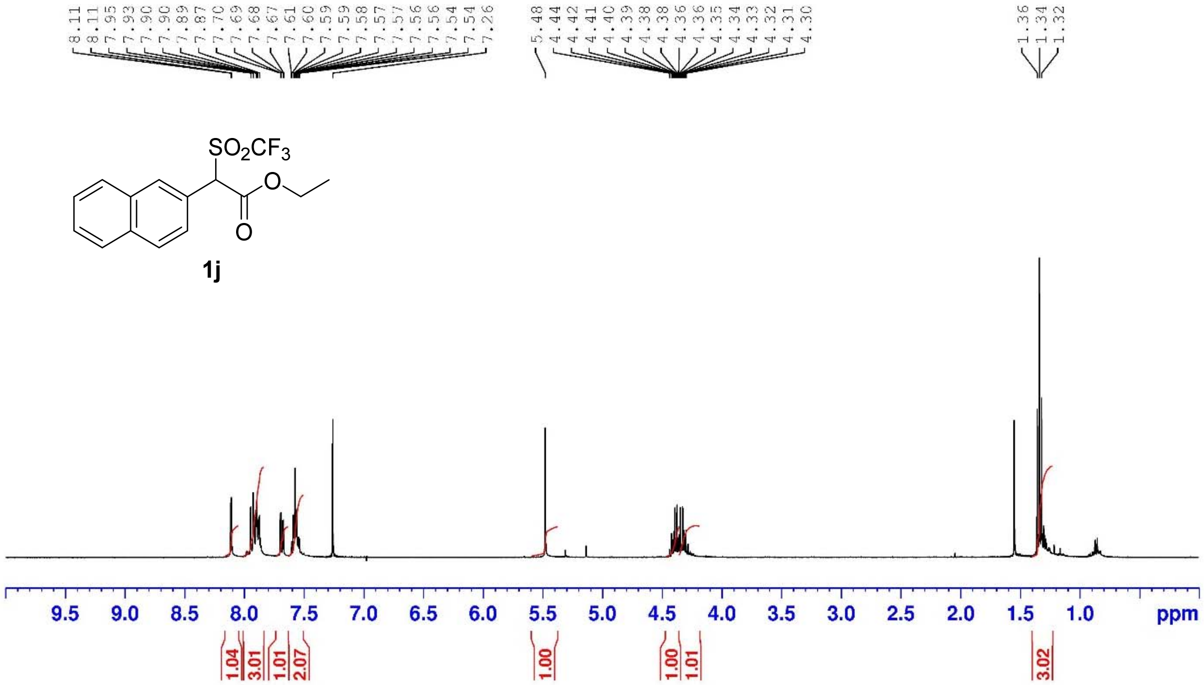

^{13}C NMR in $CDCl_3$ (100 MHz)

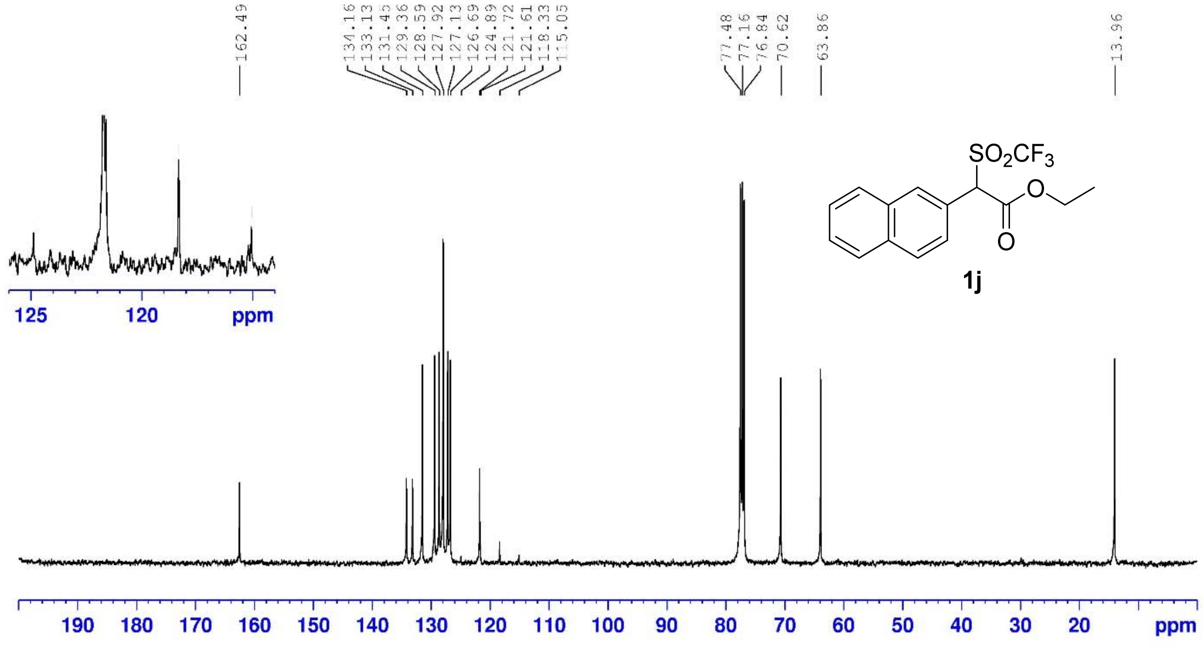

^{19}F NMR in $CDCl_3$ (376 MHz)

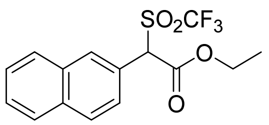

1j

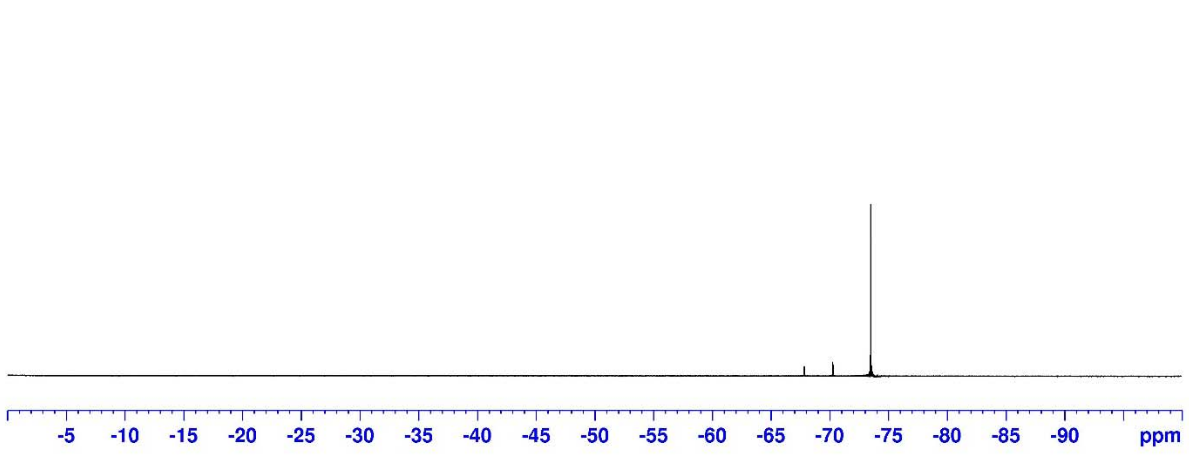

^{1}H NMR in $CDCl_3$ (400 MHz)

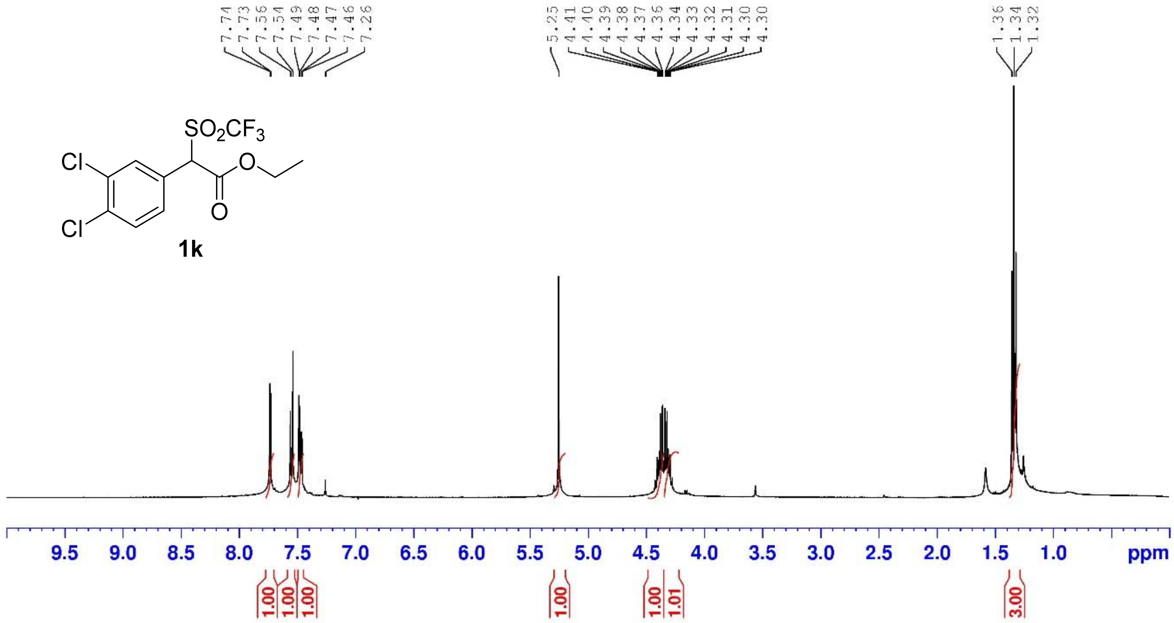

^{13}C NMR in $CDCl_3$ (100 MHz)

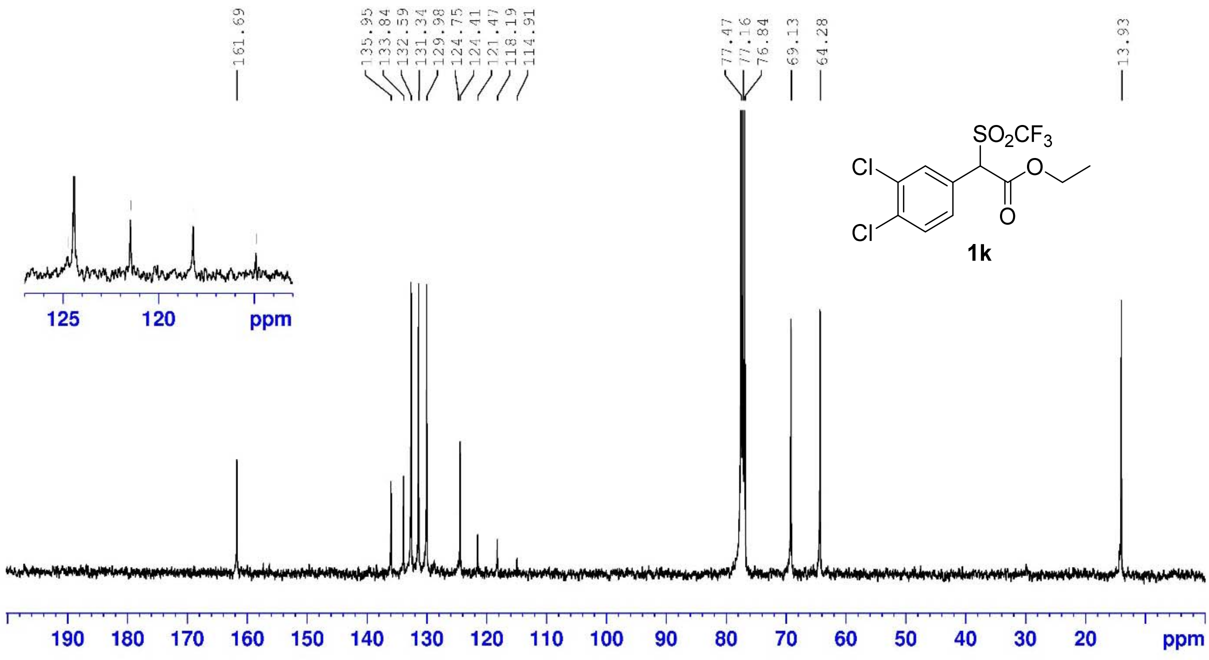

^{19}F NMR in $CDCl_3$ (376 MHz)

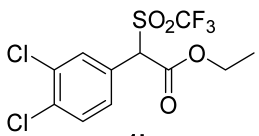

1k

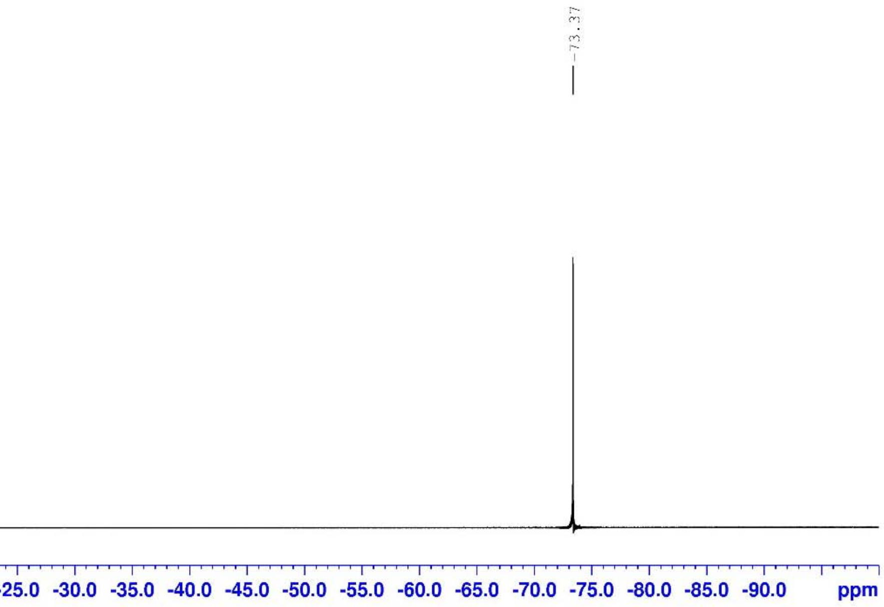

^{1}H NMR in $CDCl_3$ (300 MHz)

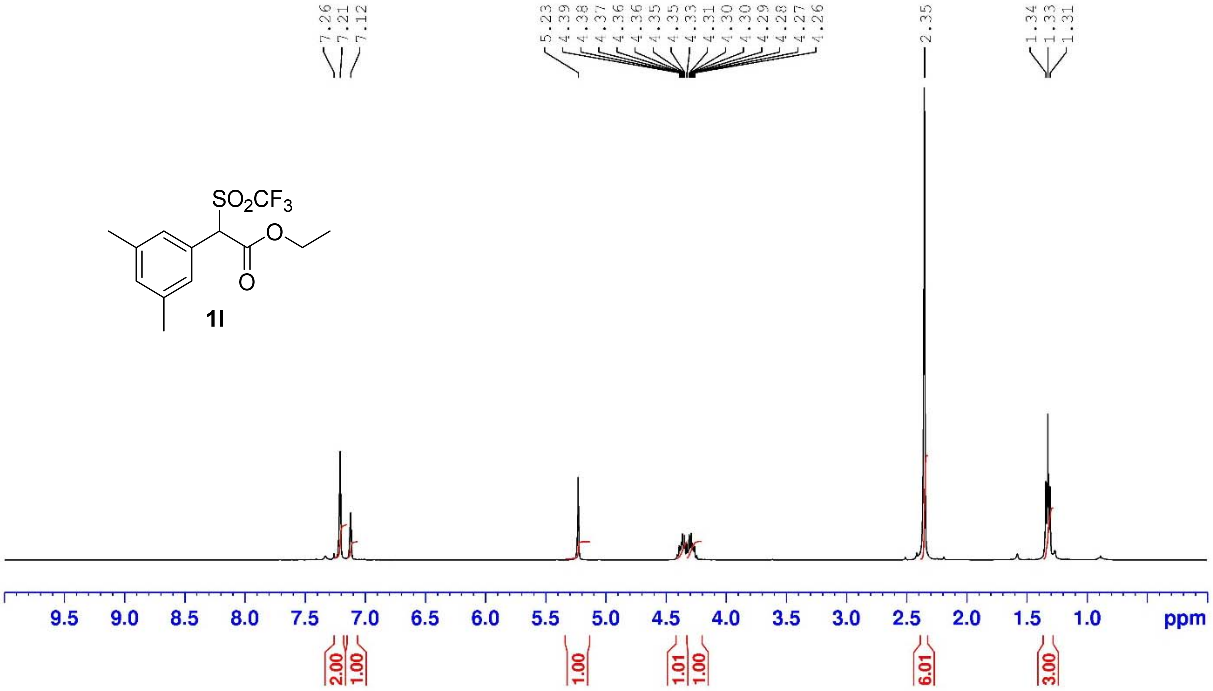

^{13}C NMR in $CDCl_3$ (100 MHz)

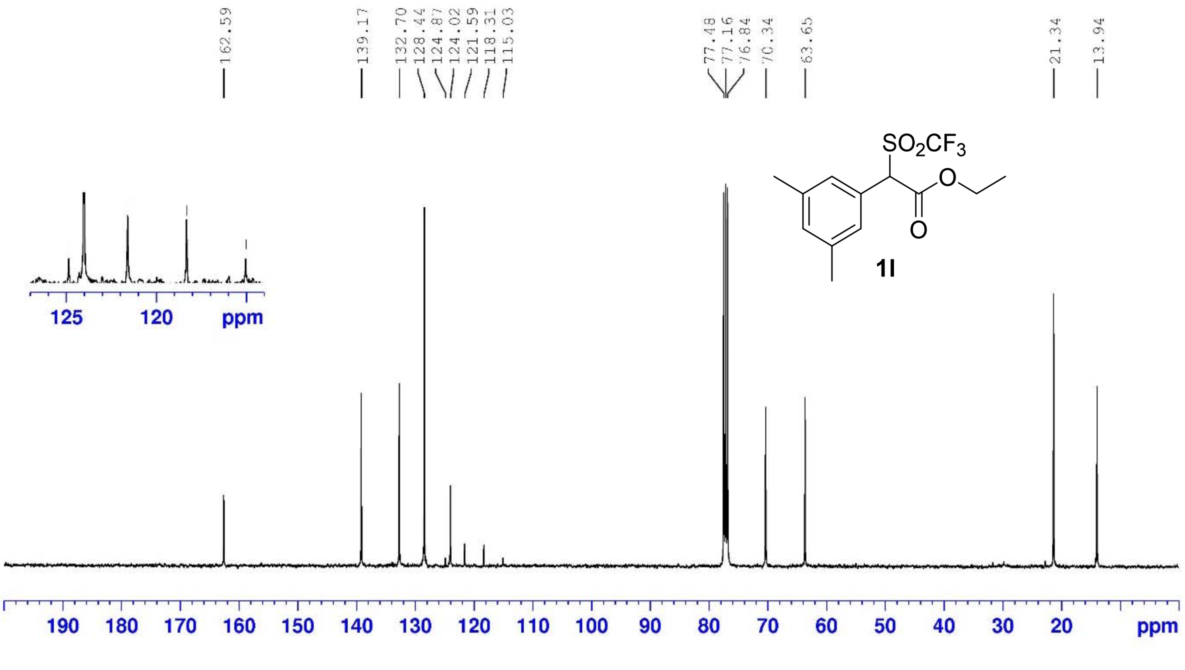

^{19}F NMR in $CDCl_3$ (376 MHz)

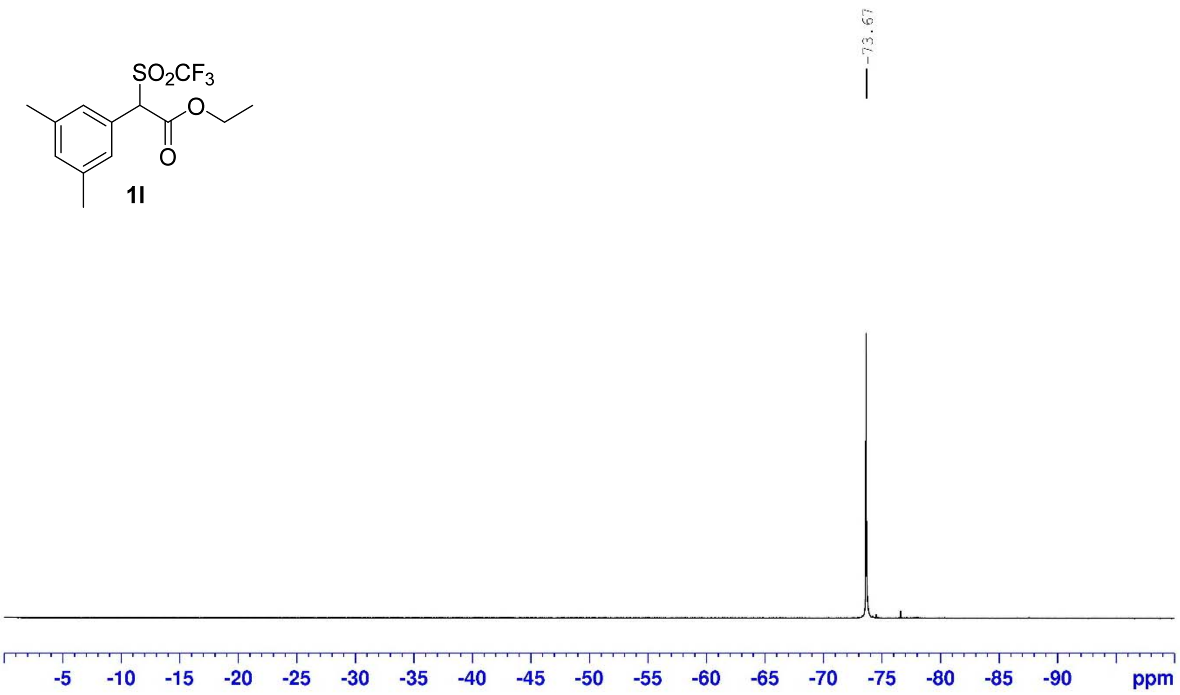

^{1}H NMR in $CDCl_3$ (300 MHz)

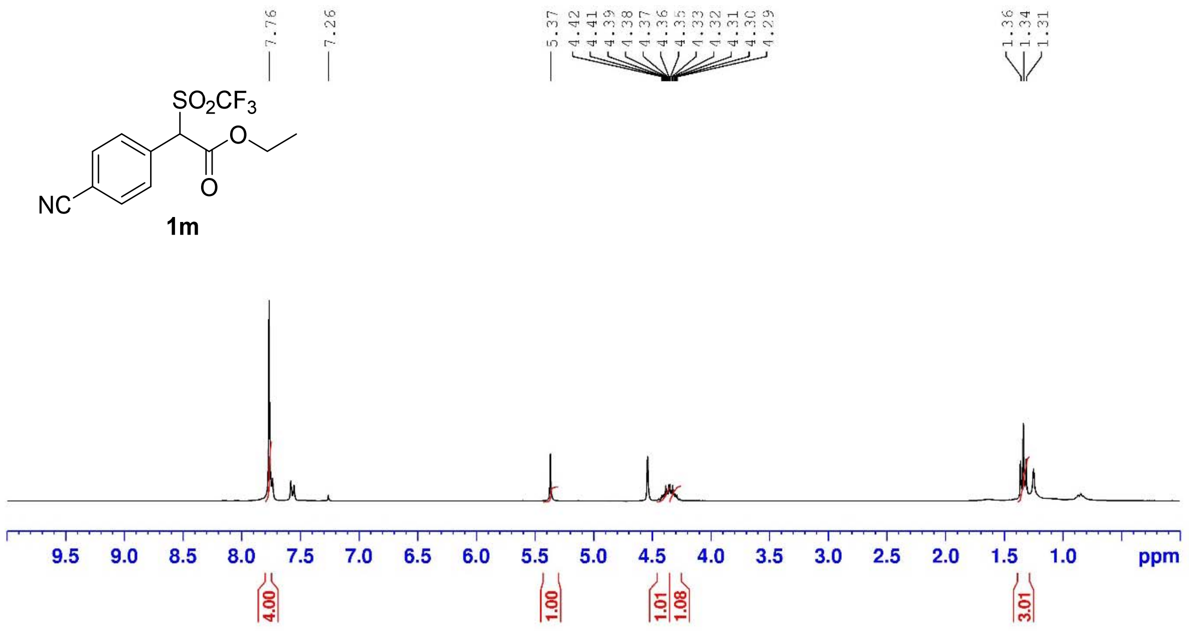

^{13}C NMR in $CDCl_3$ (150 MHz)

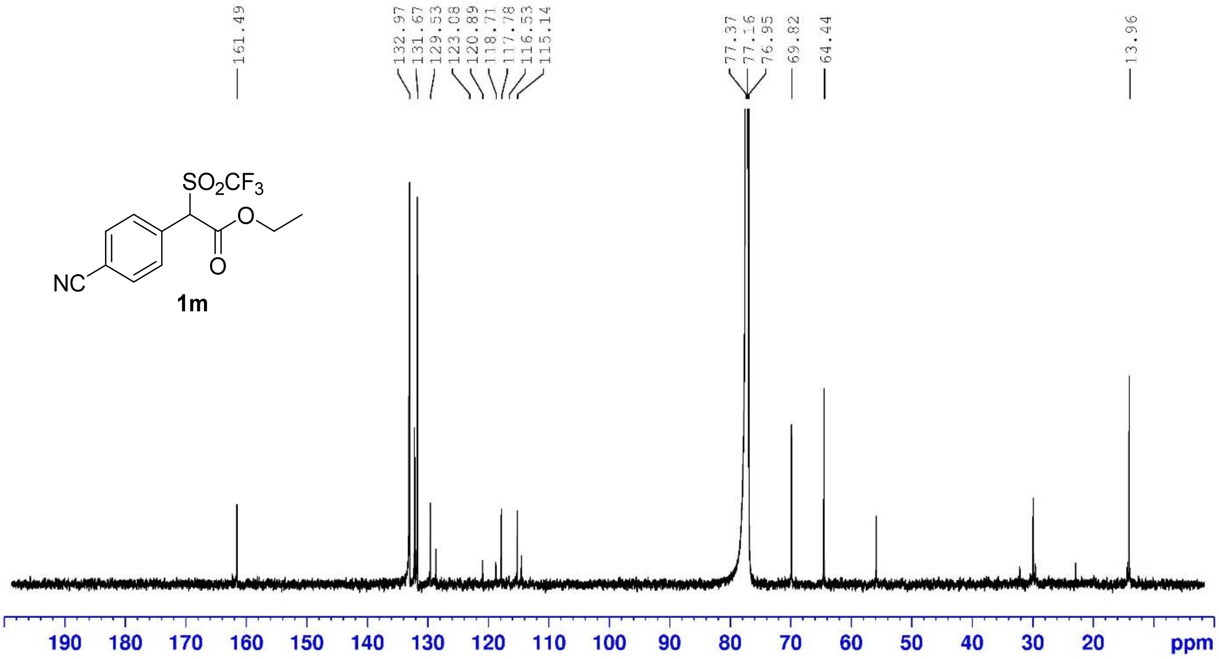

^{19}F NMR in $CDCl_3$ (376 MHz)

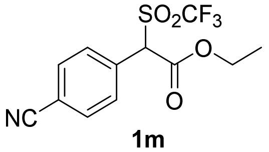

1m

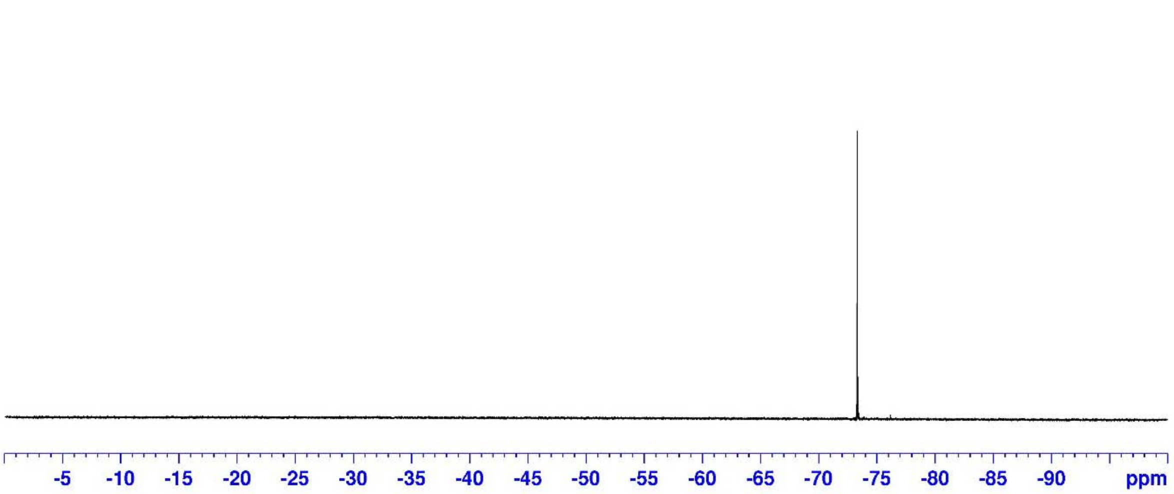

^{1}H NMR in $CDCl_3$ (300 MHz)

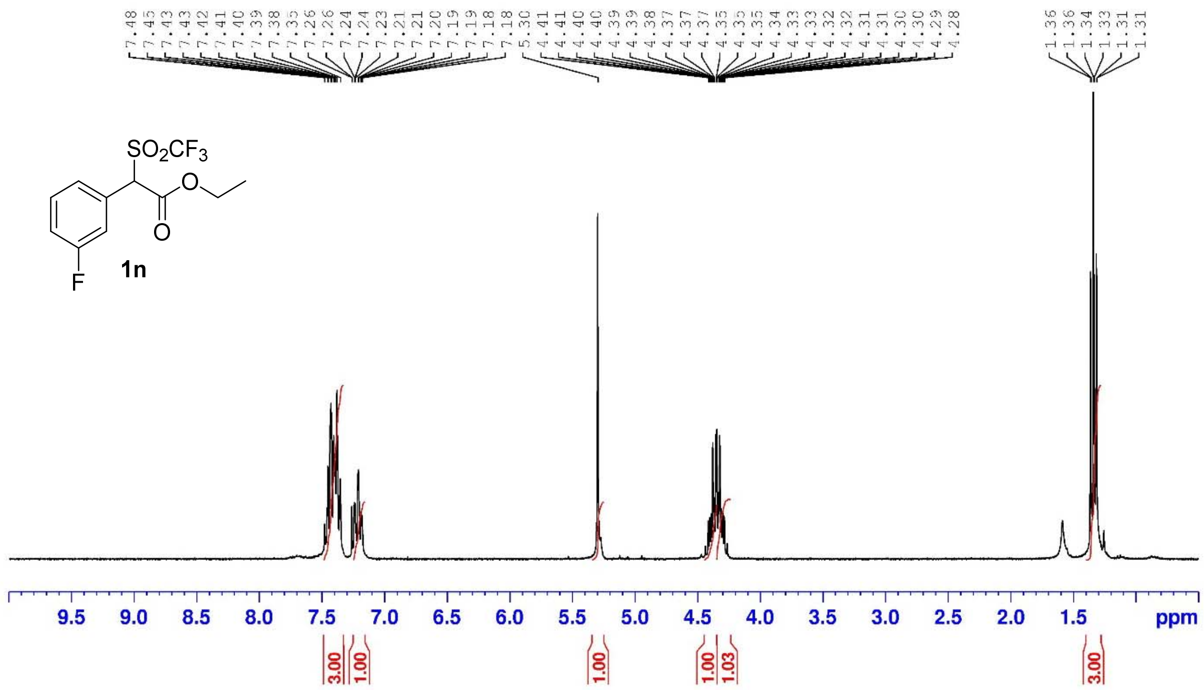

^{13}C NMR in $CDCl_3$ (75 MHz)

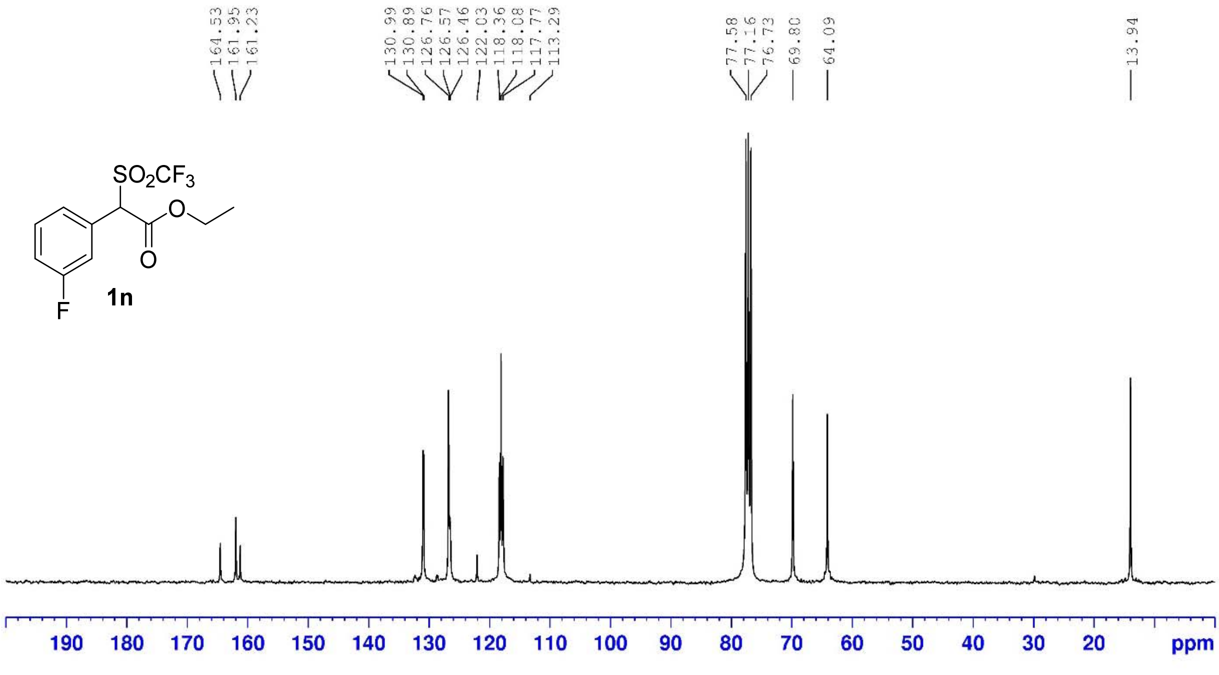

^{19}F NMR in $CDCl_3$ (376 MHz)

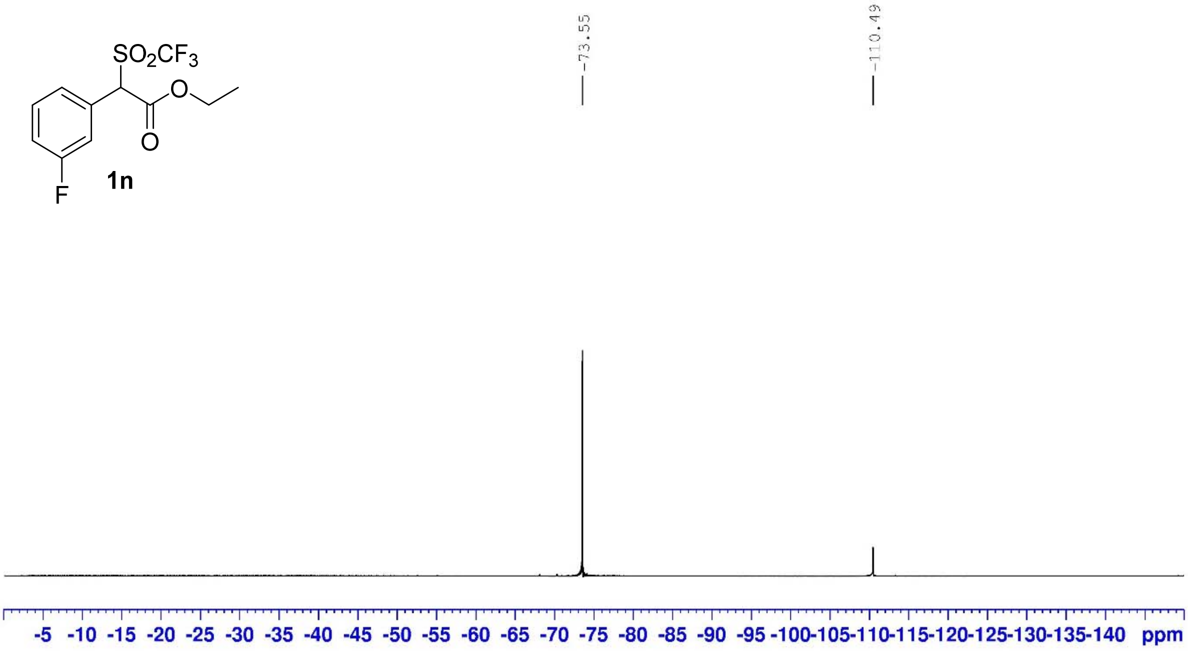

^{1}H NMR in $CDCl_3$ (400 MHz)

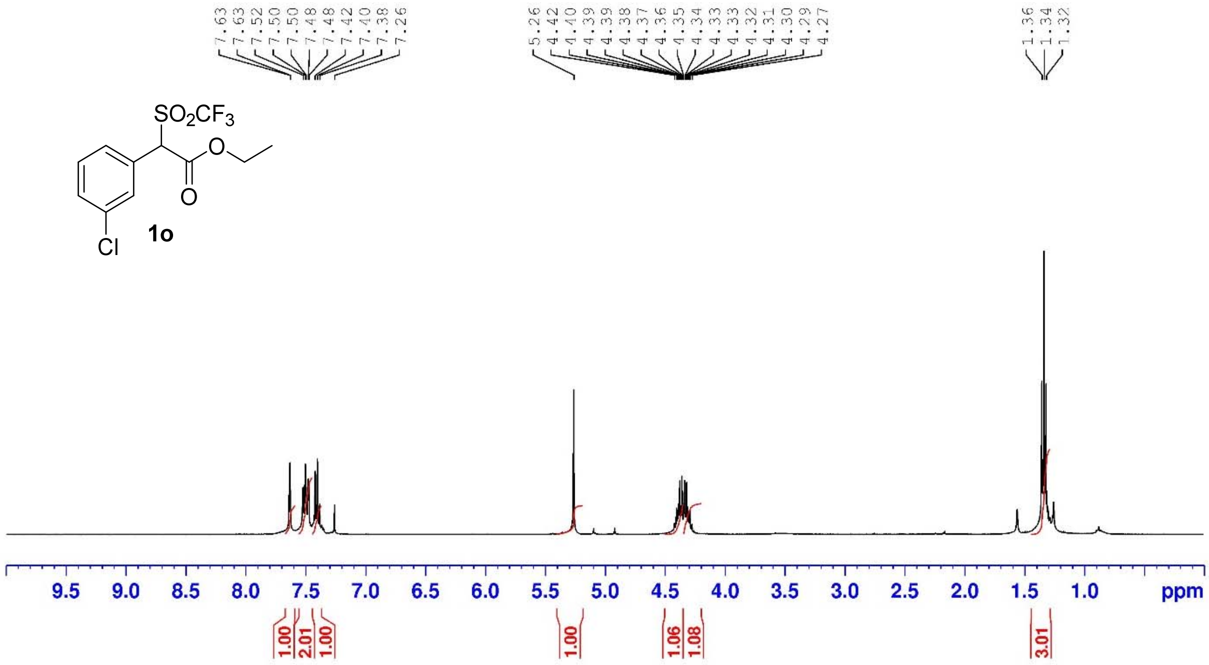

^{13}C NMR in $CDCl_3$ (100 MHz)

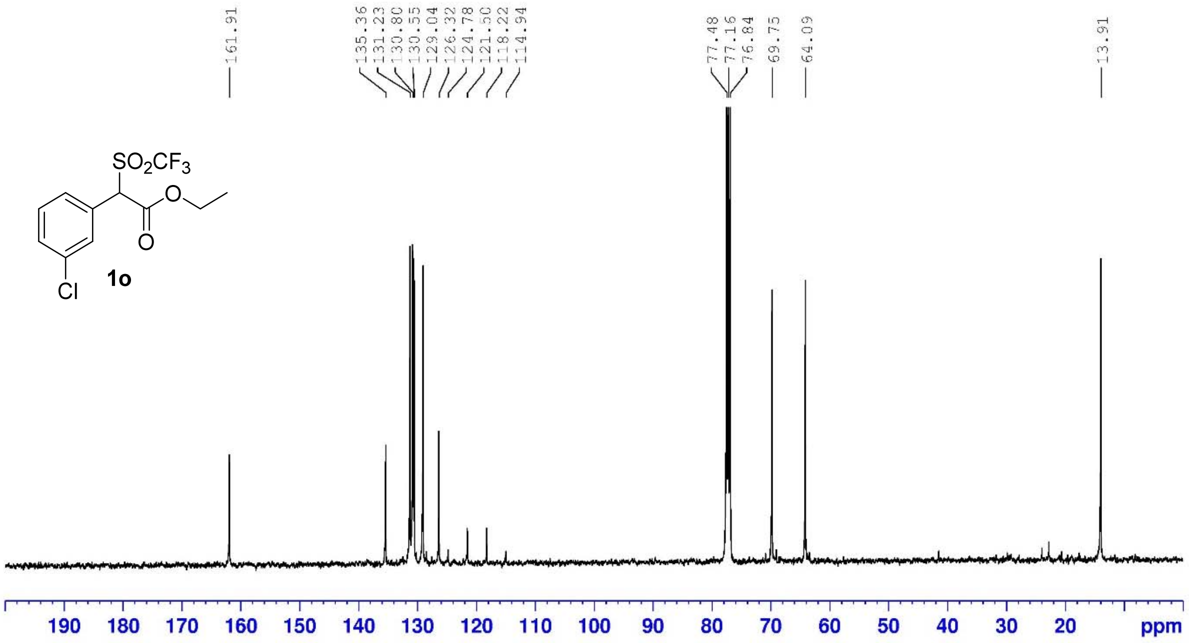

^{19}F NMR in $CDCl_3$ (376 MHz)

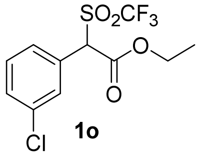

1o

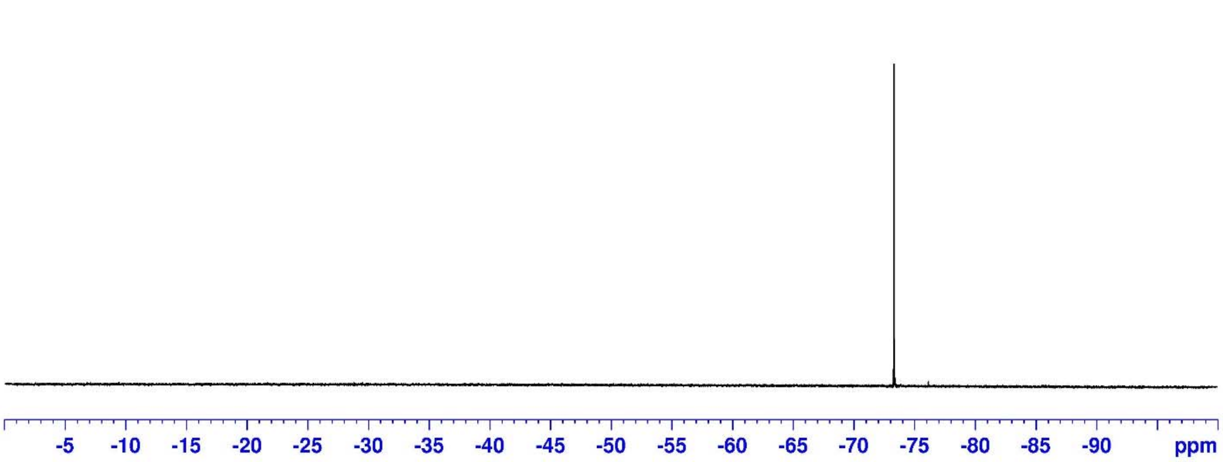

^{1}H NMR in $CDCl_3$ (300 MHz)

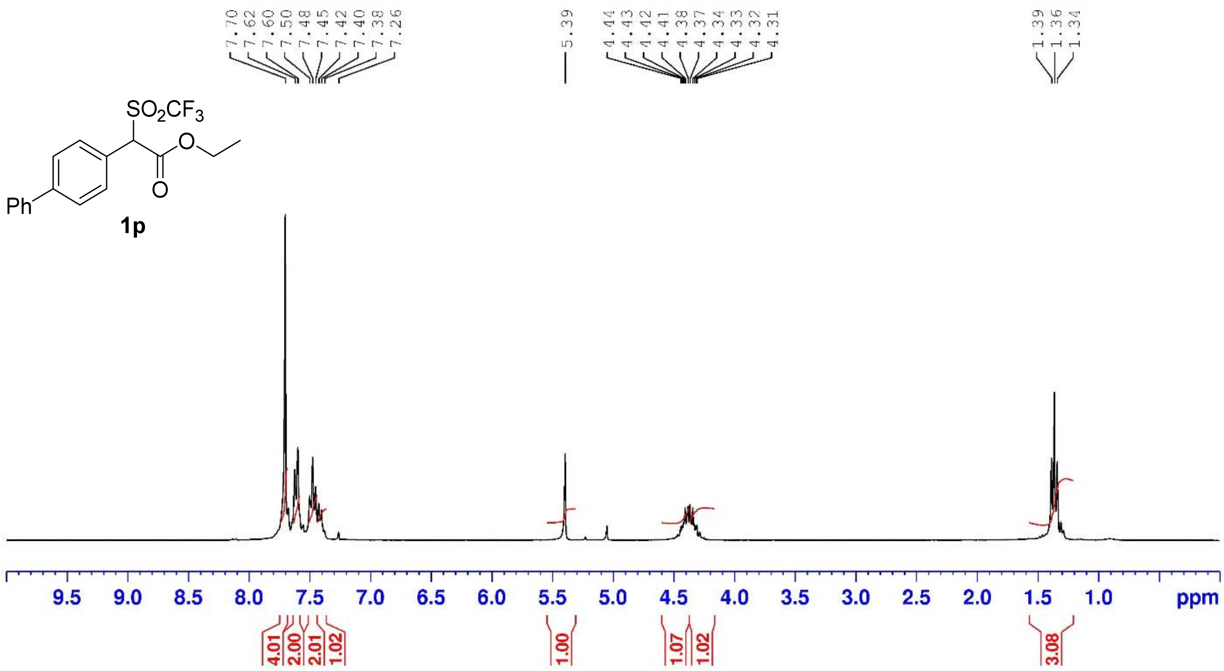

^{13}C NMR in $CDCl_3$ (75 MHz)

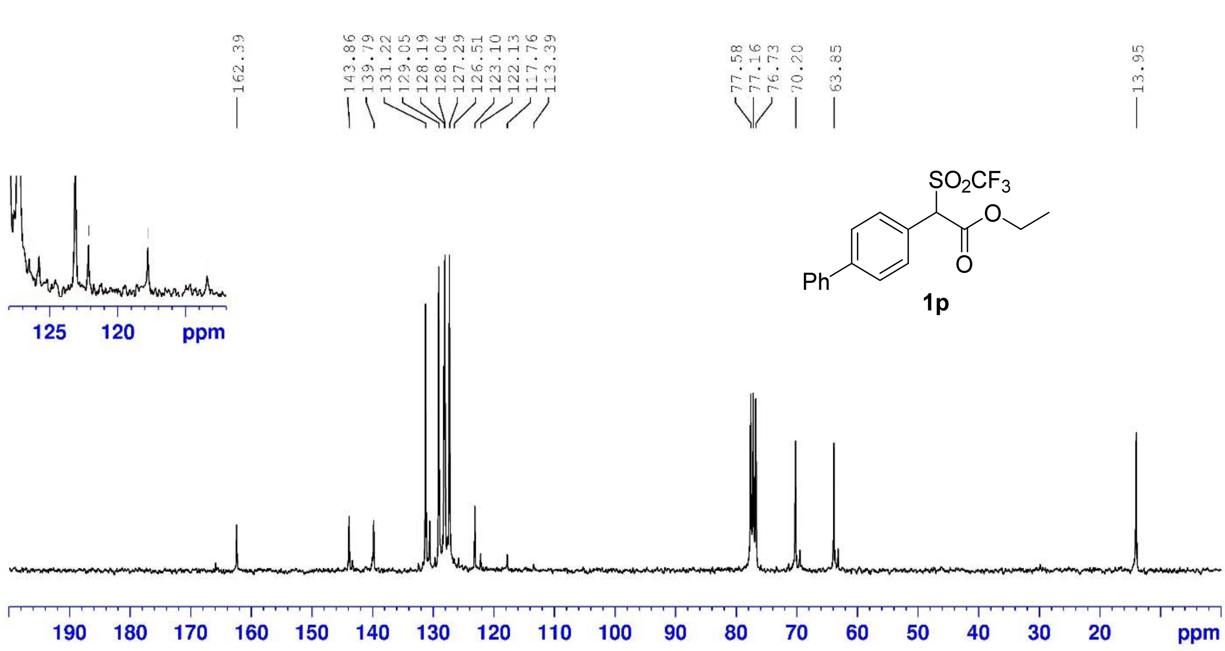

^{19}F NMR in $CDCl_3$ (376 MHz)

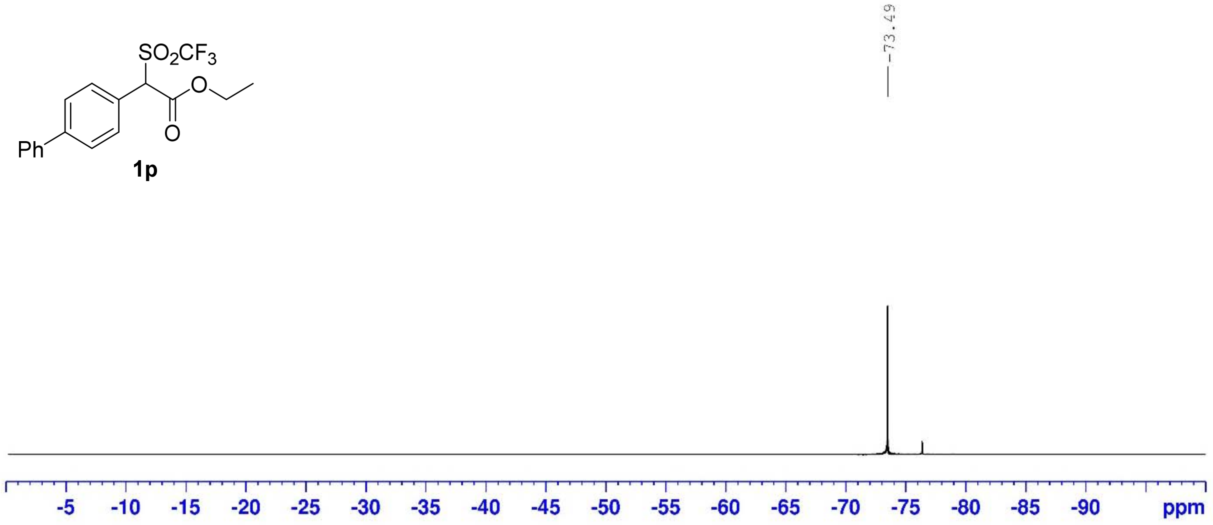

^{1}H NMR in $CDCl_3$ (600 MHz)

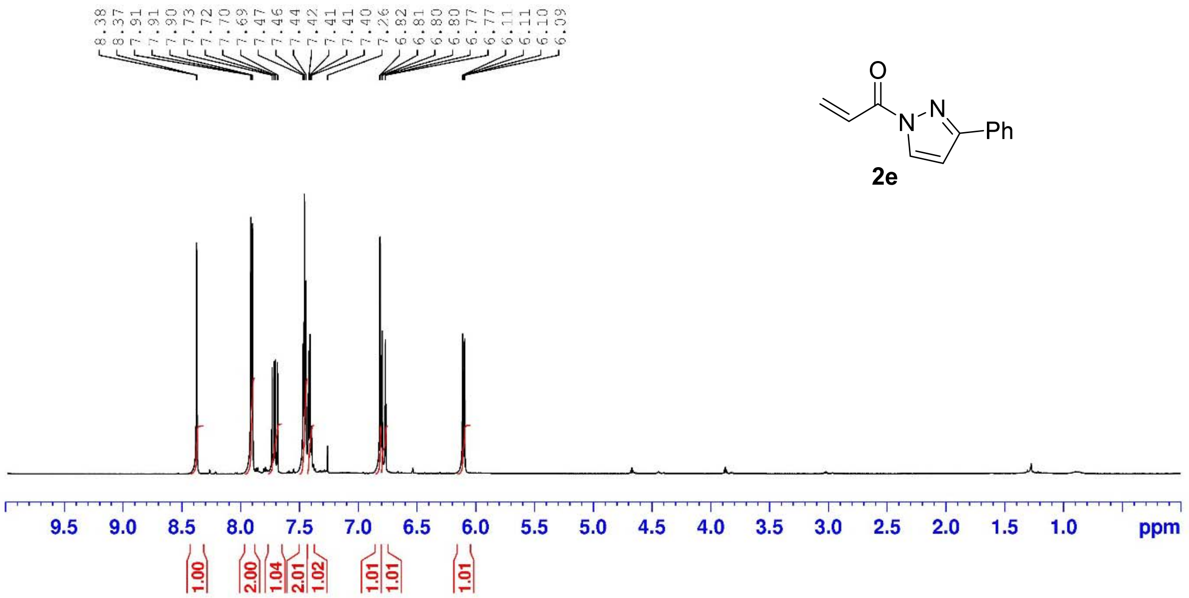

^{13}C NMR in $CDCl_3$ (150 MHz)

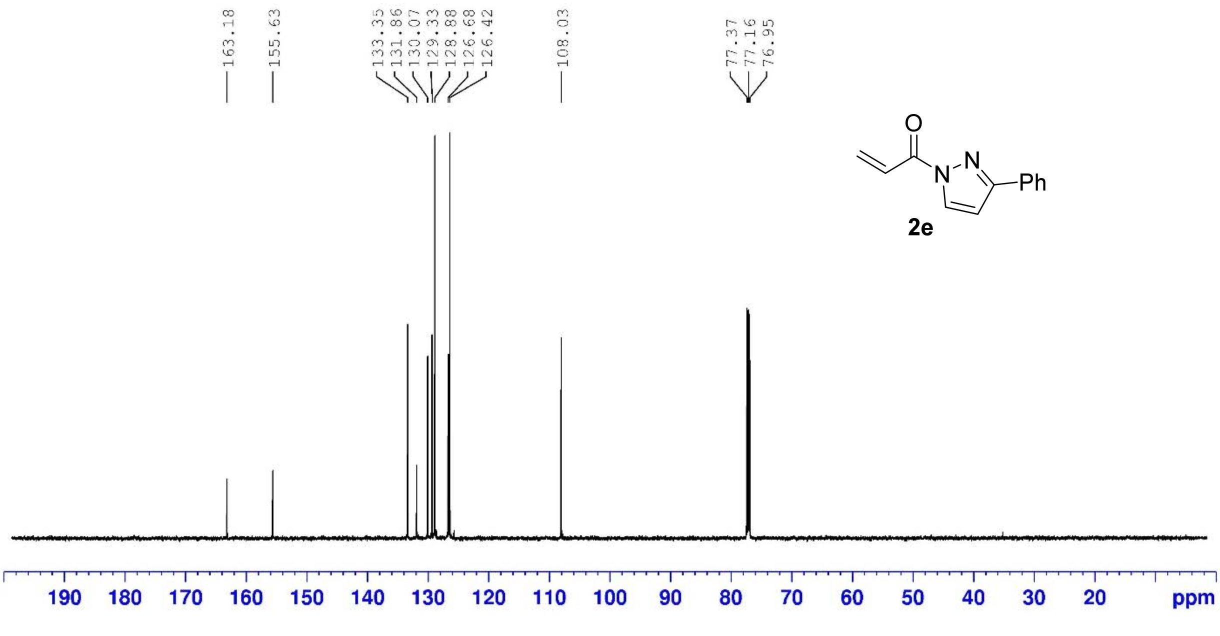

^{1}H NMR in $CDCl_3$ (300 MHz)

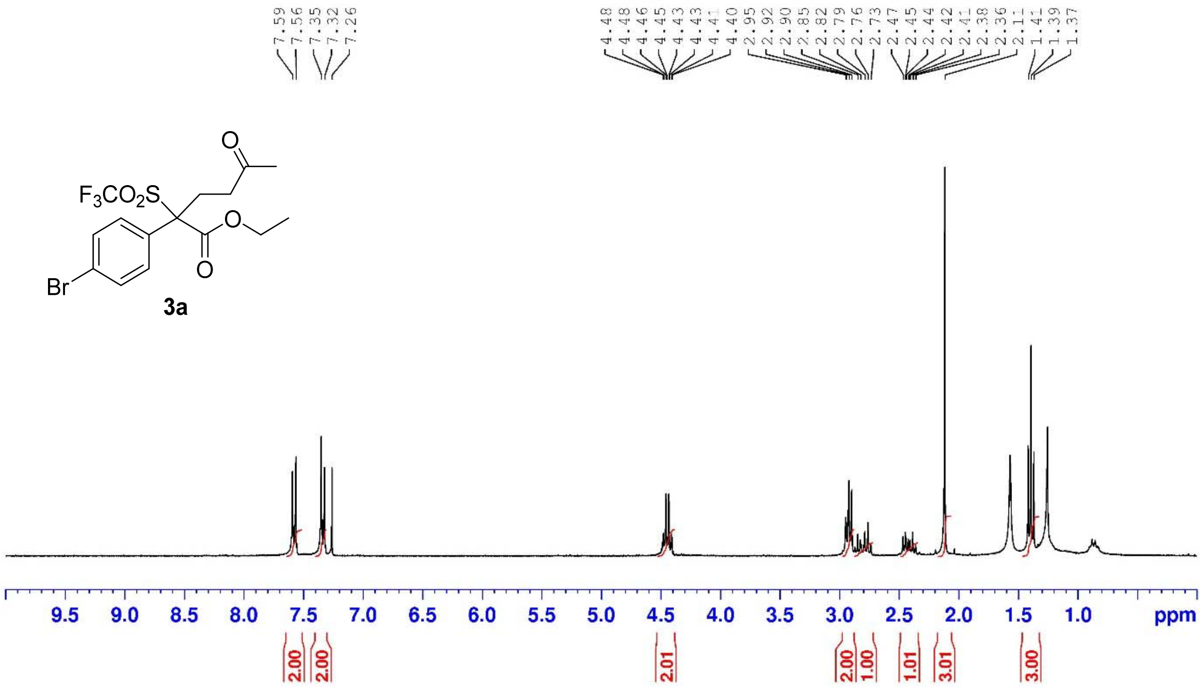

^{13}C NMR in $CDCl_3$ (75 MHz)

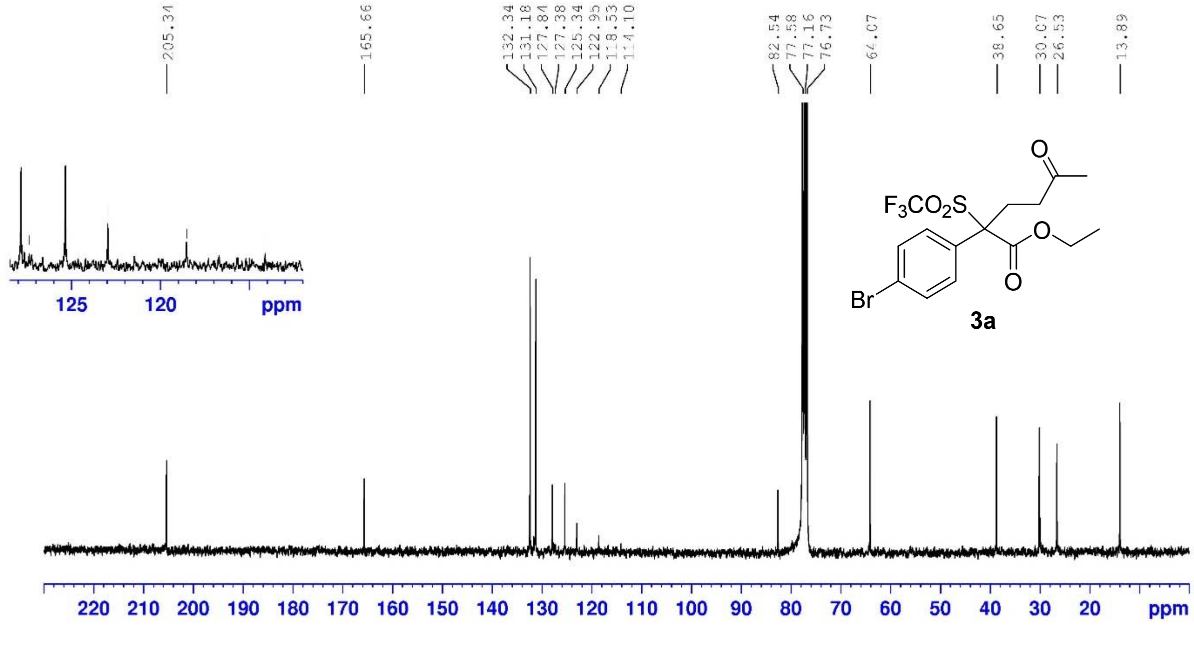

^{19}F NMR in $CDCl_3$ (376 MHz)

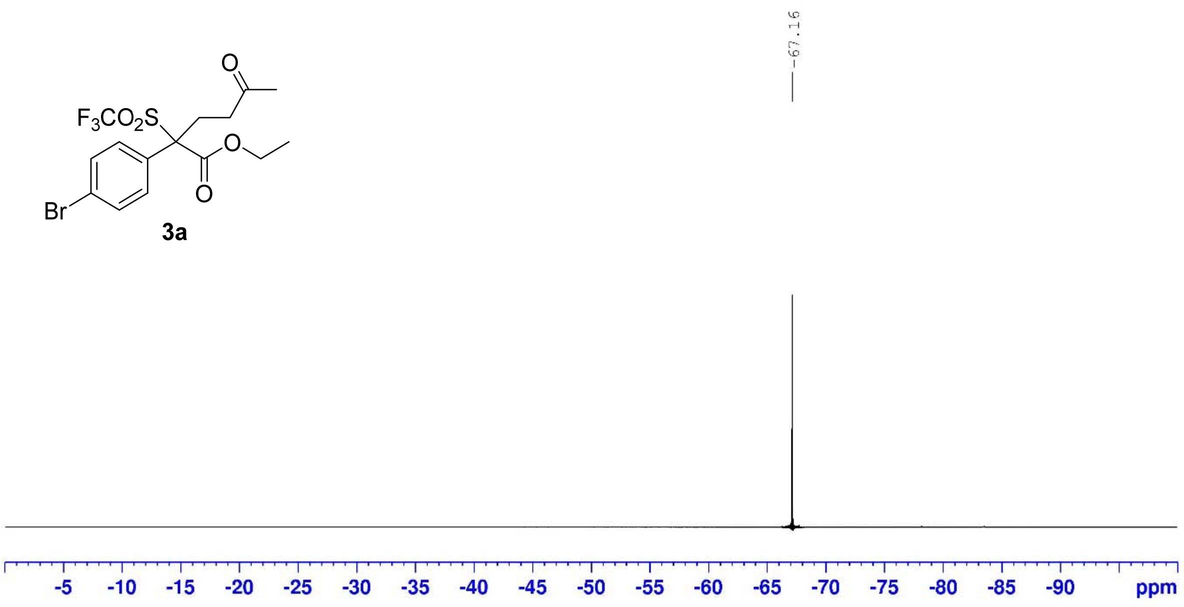

3a

^{1}H NMR in $CDCl_3$ (300 MHz)

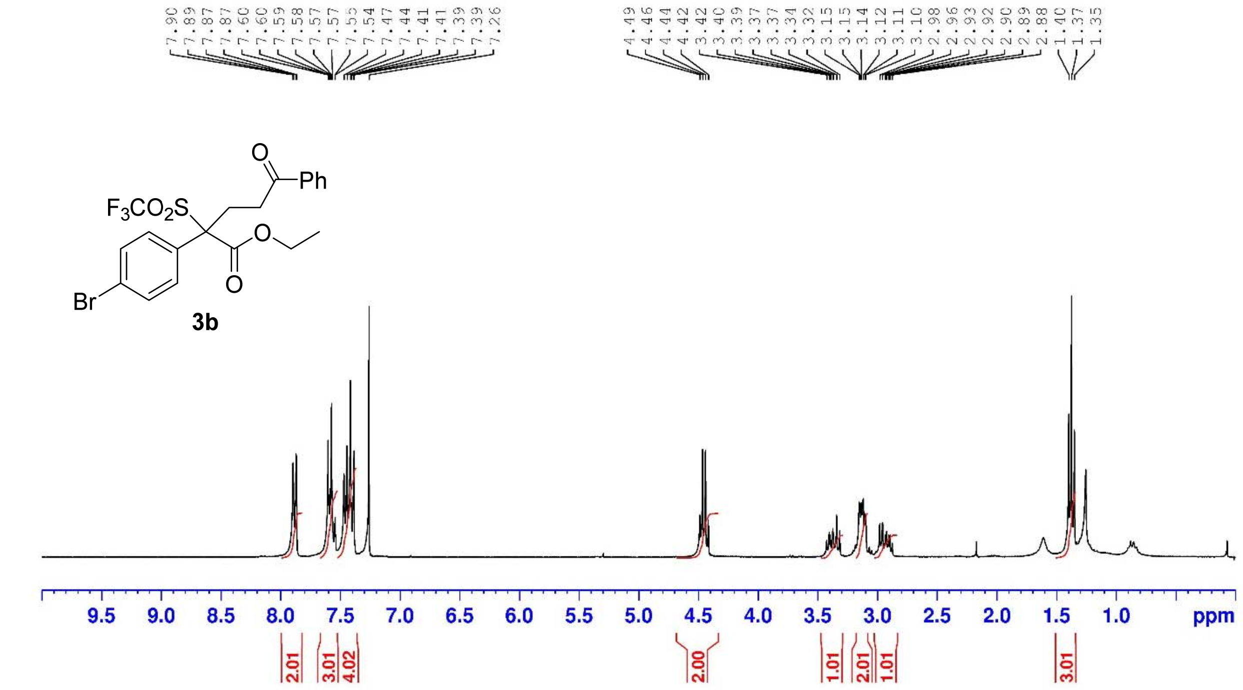

^{13}C NMR in $CDCl_3$ (75 MHz)

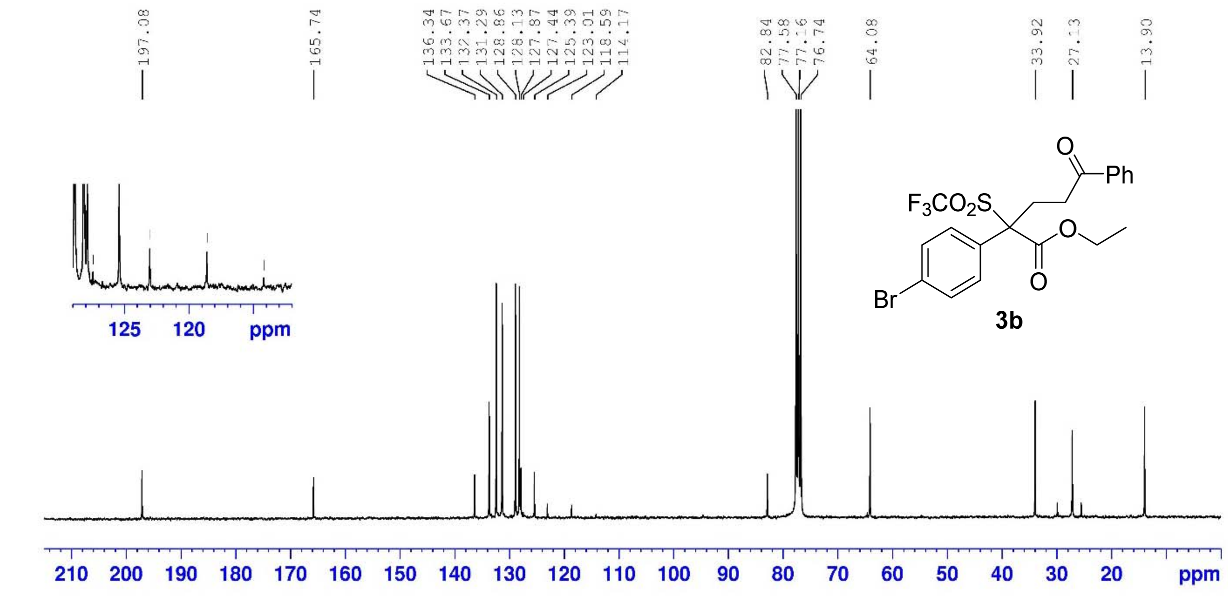

^{19}F NMR in $CDCl_3$ (282 MHz)

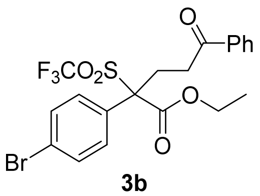

3b

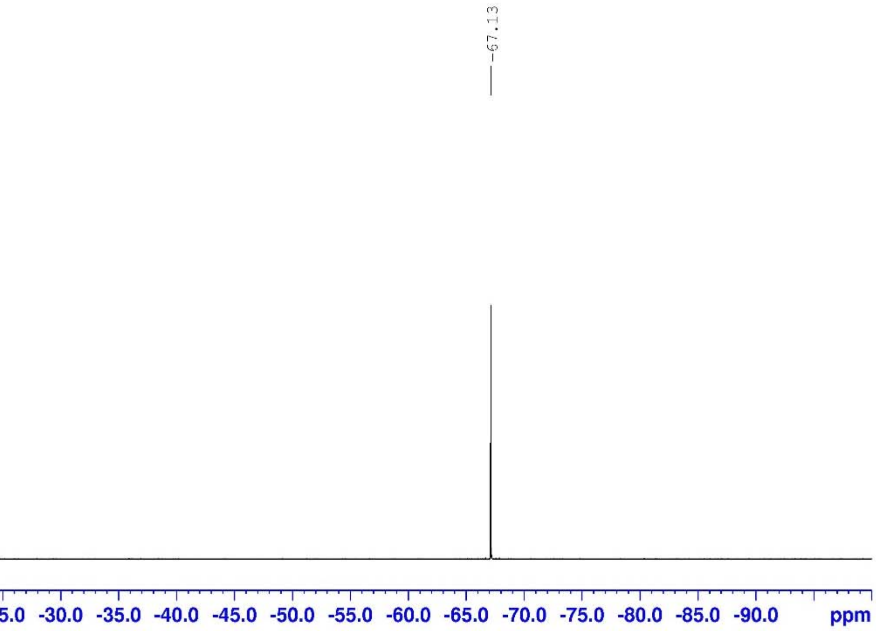

^{1}H NMR in $CDCl_3$ (400 MHz)

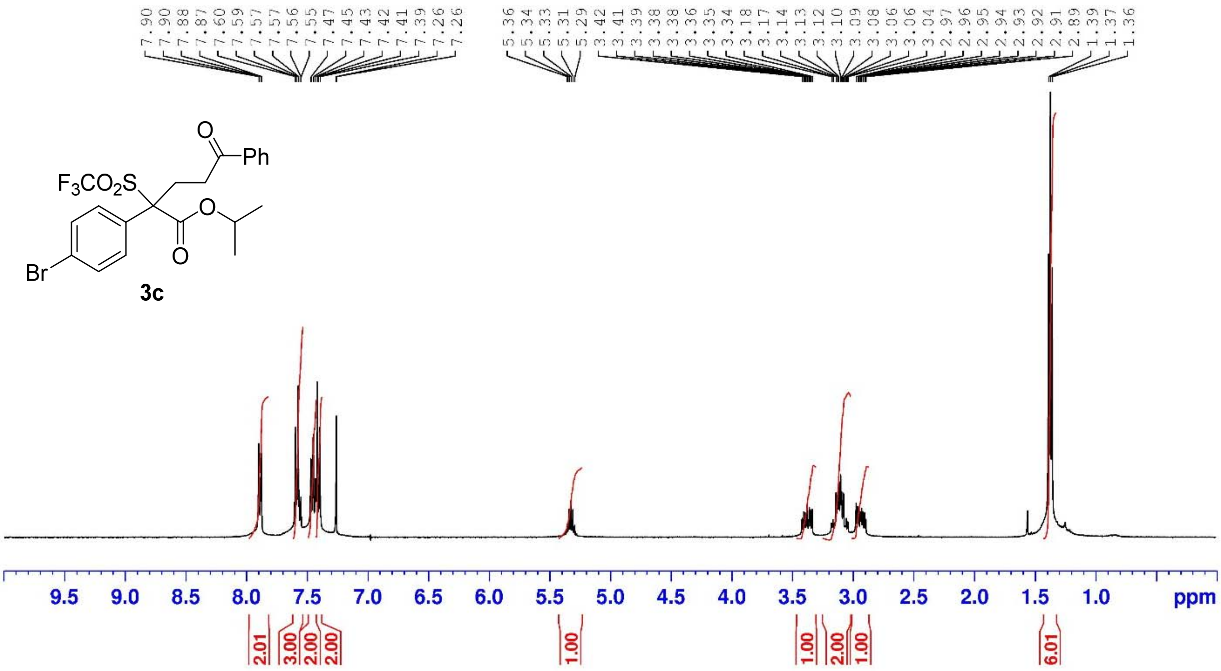

^{13}C NMR in $CDCl_3$ (100 MHz)

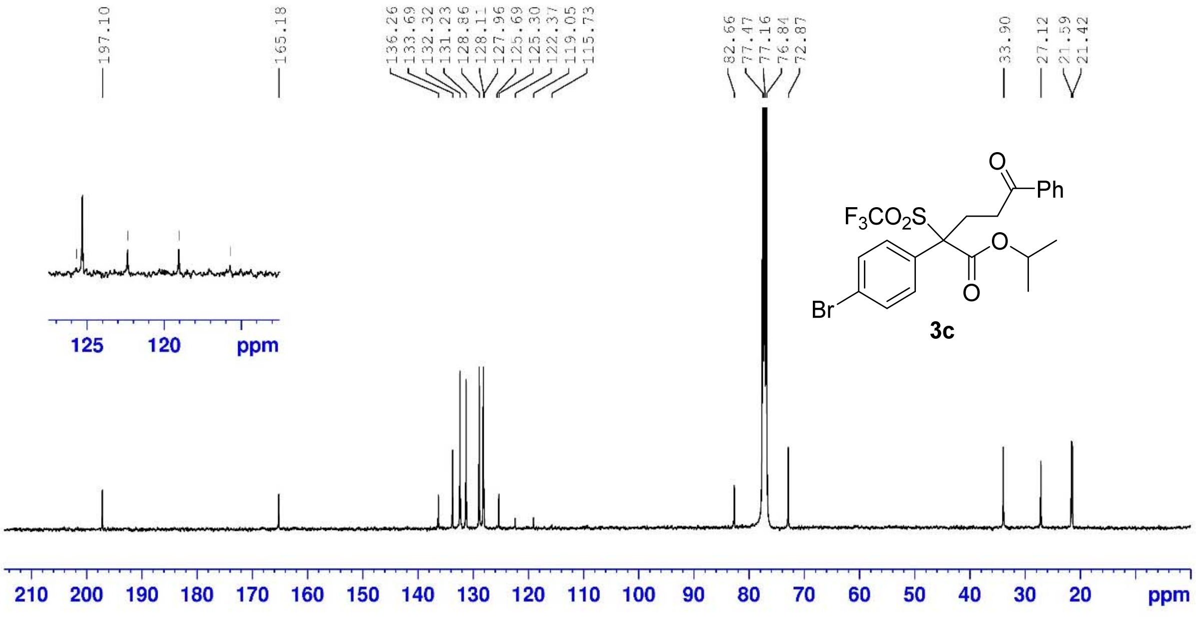

^{19}F NMR in $CDCl_3$ (376 MHz)

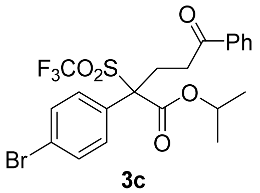

3c

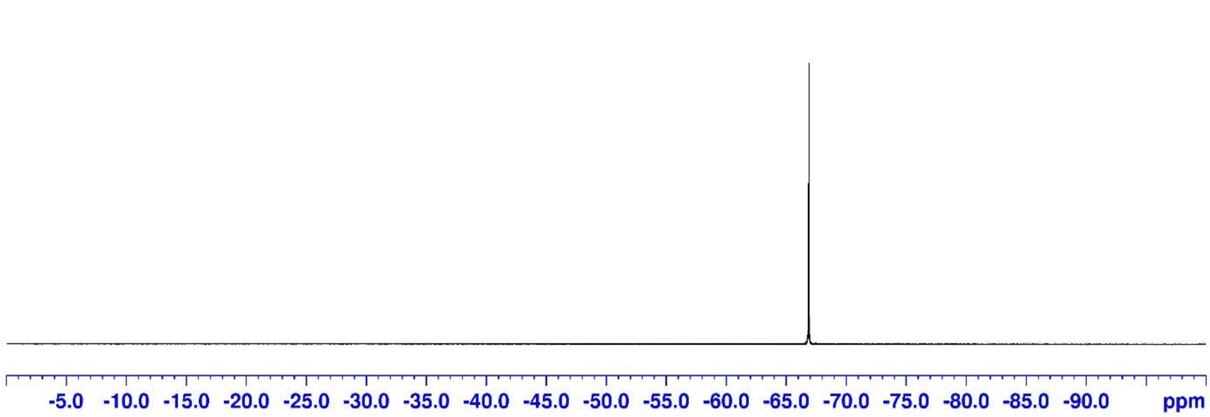

^{1}H NMR in $CDCl_3$ (400 MHz)

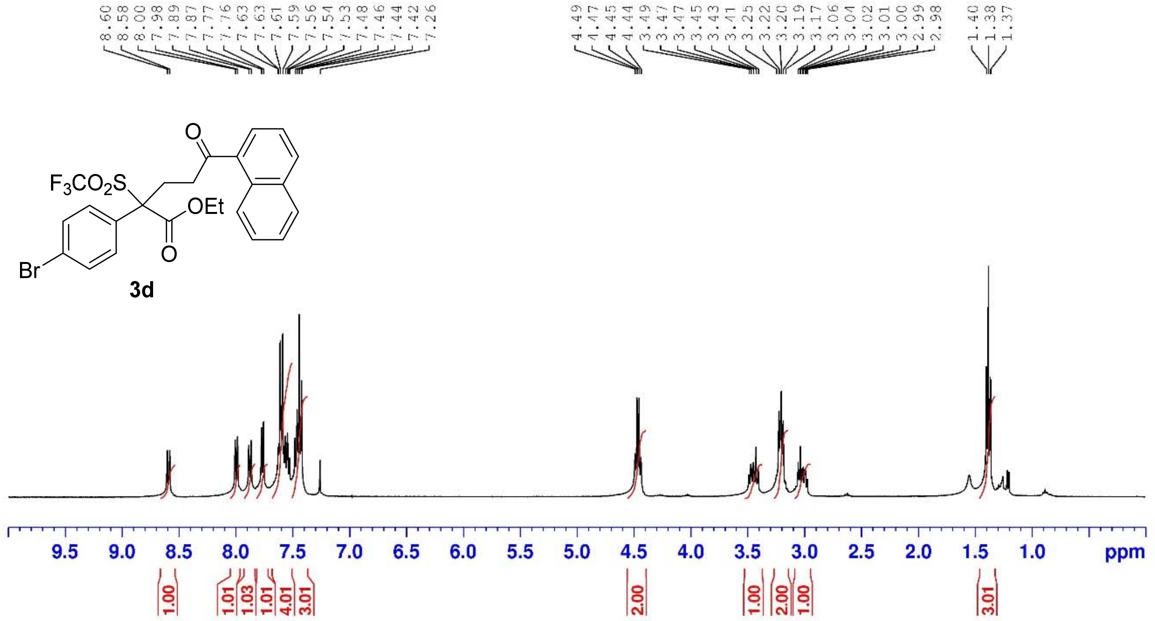

^{13}C NMR in $CDCl_3$ (100 MHz)

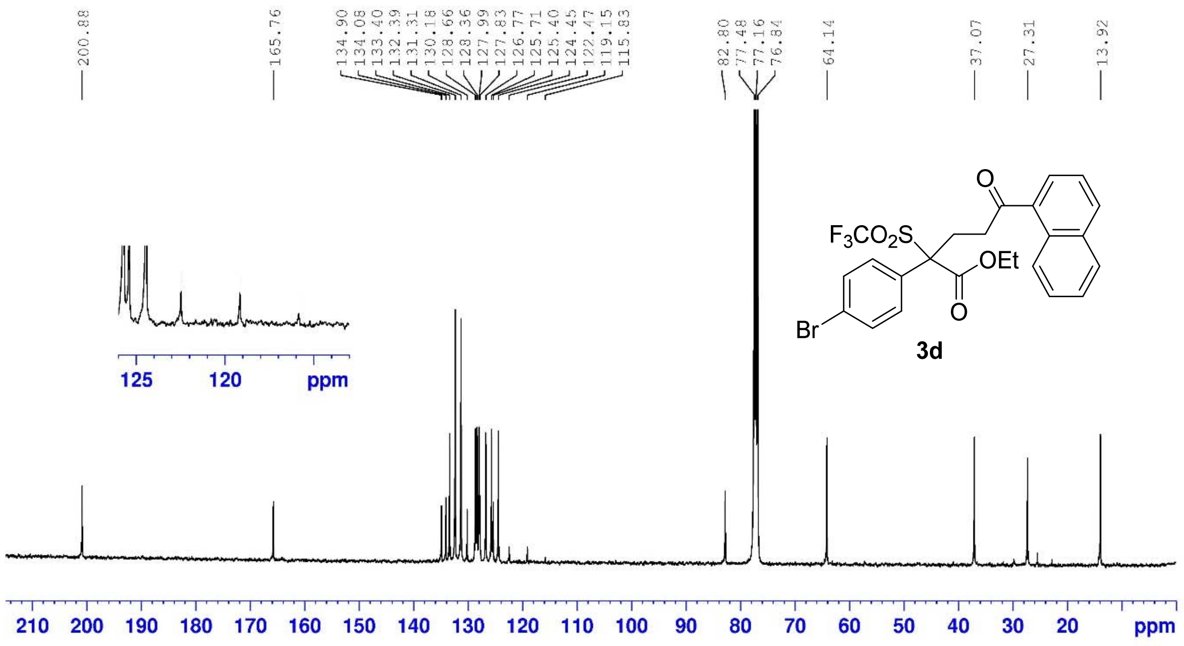

^{19}F NMR in $CDCl_3$ (376 MHz)

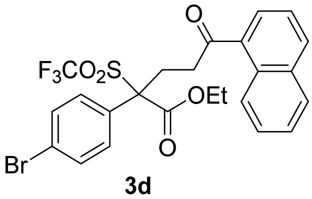

3d

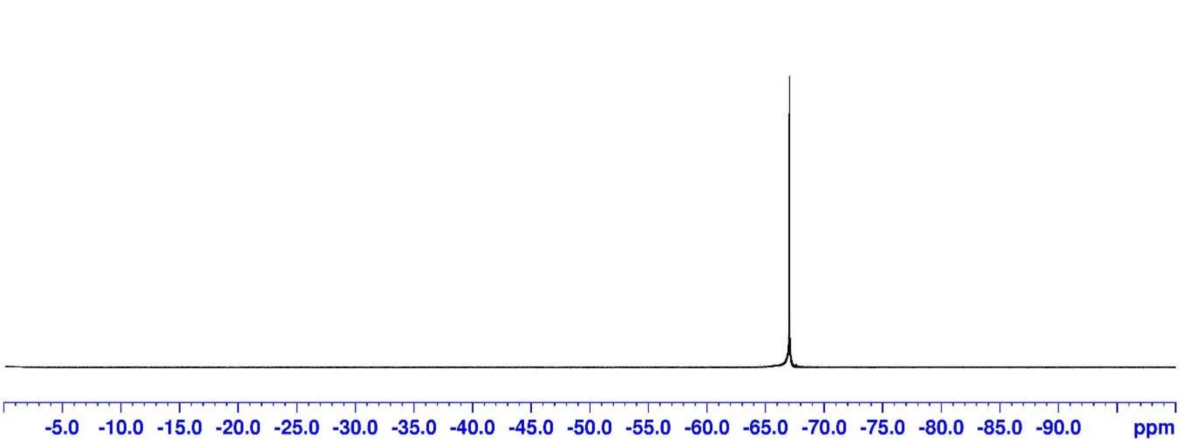

^{1}H NMR in $CDCl_3$ (300 MHz)

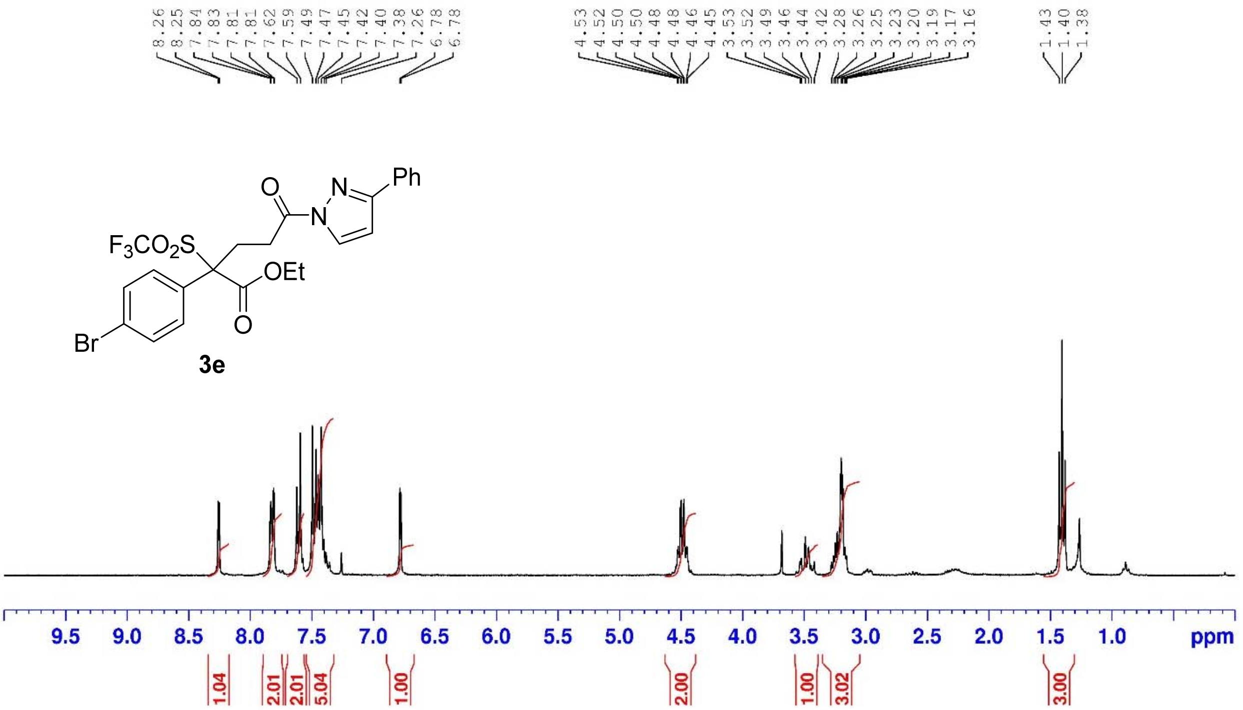

^{13}C NMR in $CDCl_3$ (75 MHz)

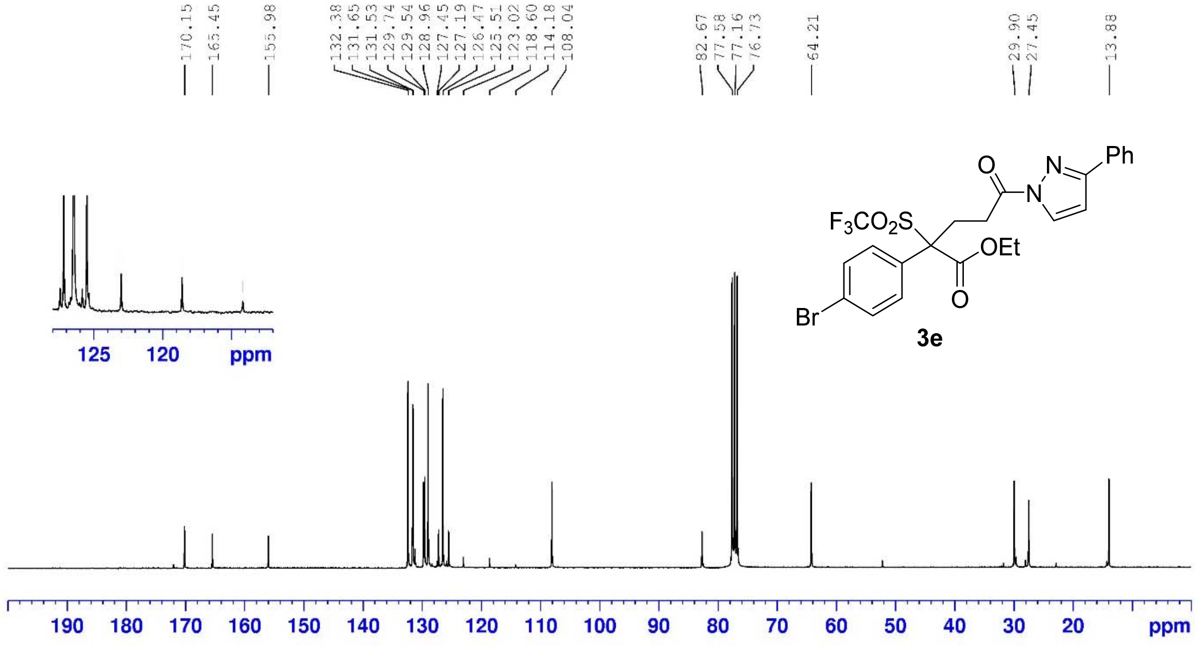

^{19}F NMR in $CDCl_3$ (282 MHz)

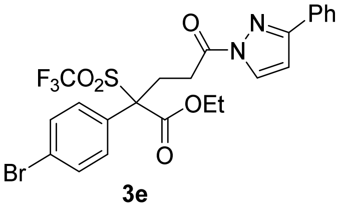

3e

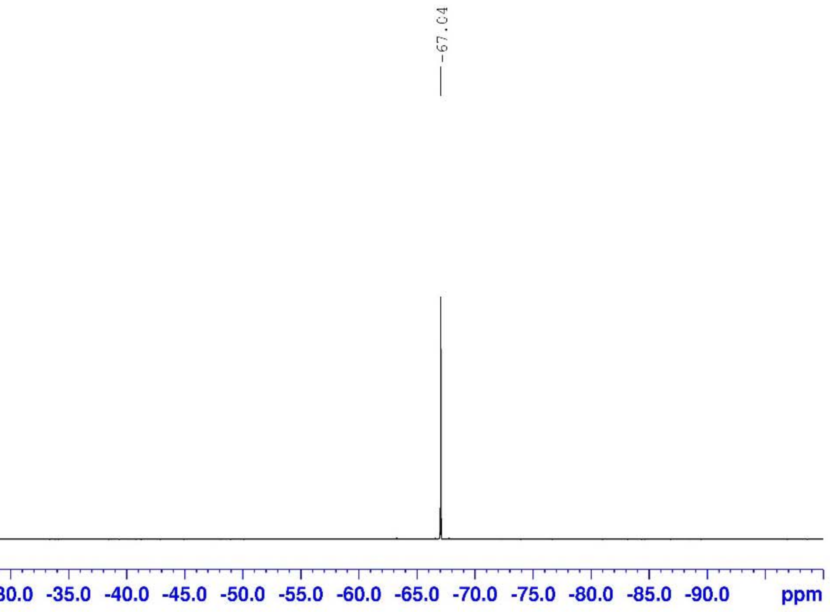

^{1}H NMR in $CDCl_3$ (300 MHz)

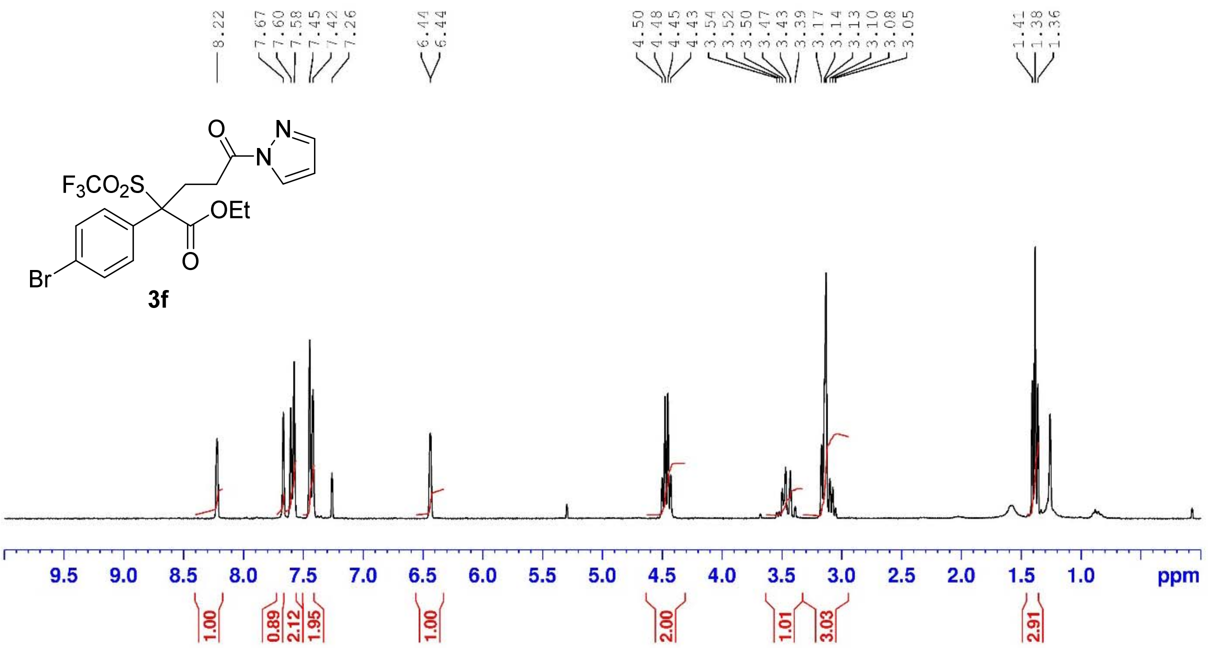

^{13}C NMR in $CDCl_3$ (75 MHz)

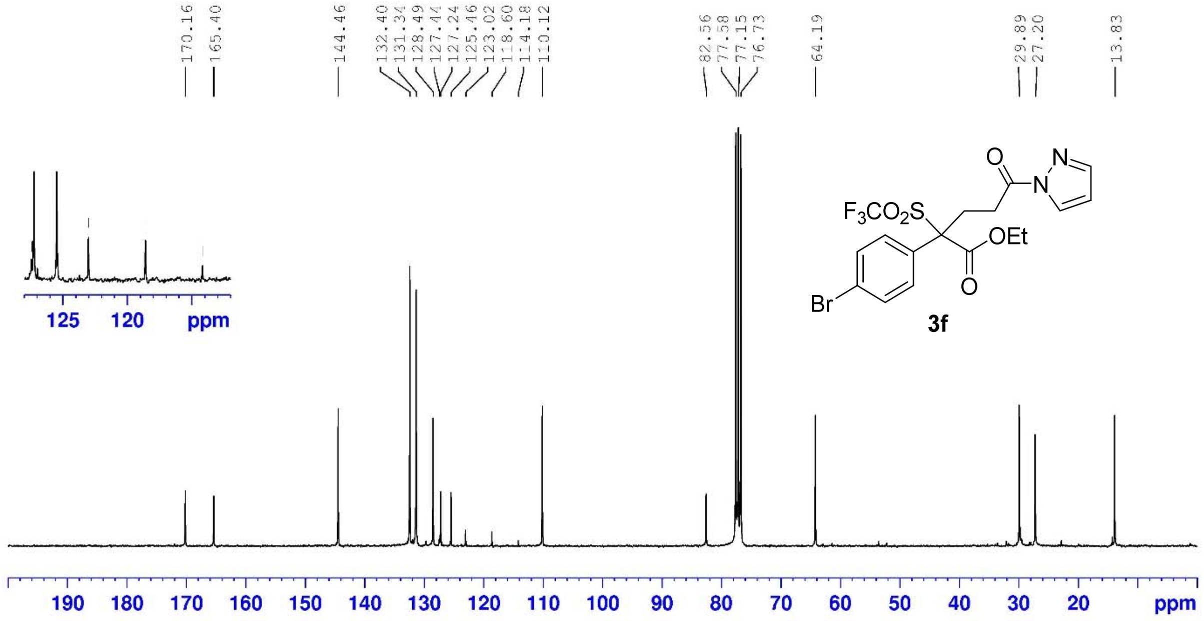

^{19}F NMR in $CDCl_3$ (282 MHz)

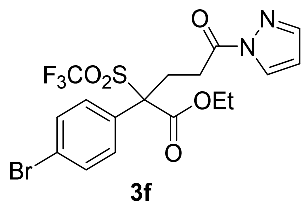

3f

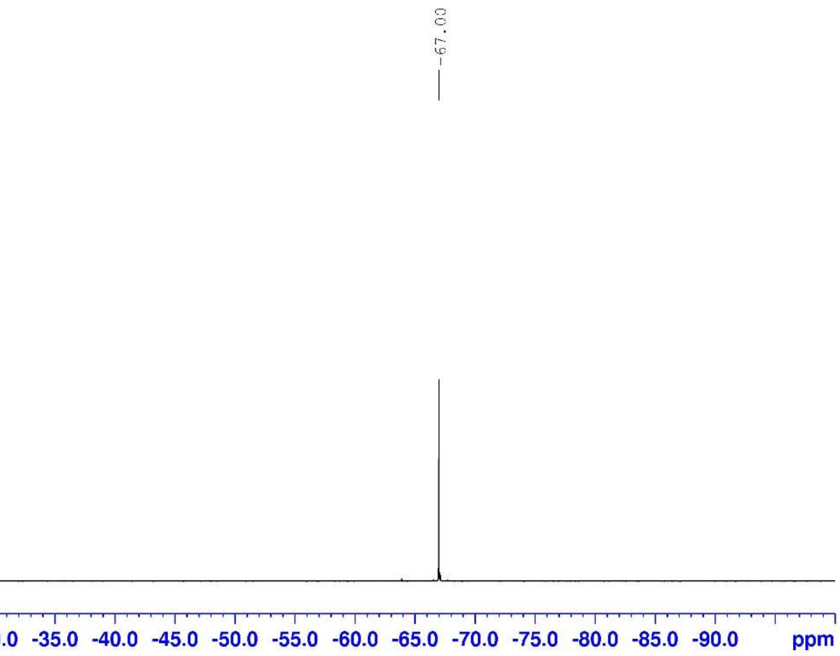

^1H NMR in $CDCl_3$ (250 MHz)

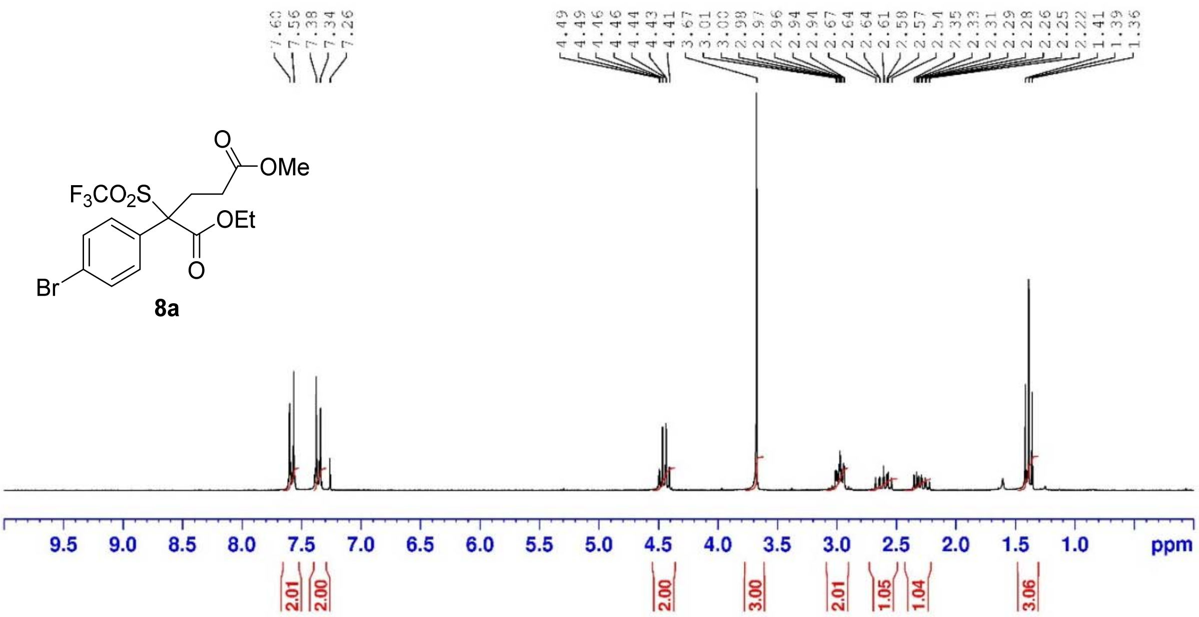

^{13}C NMR in $CDCl_3$ (75 MHz)

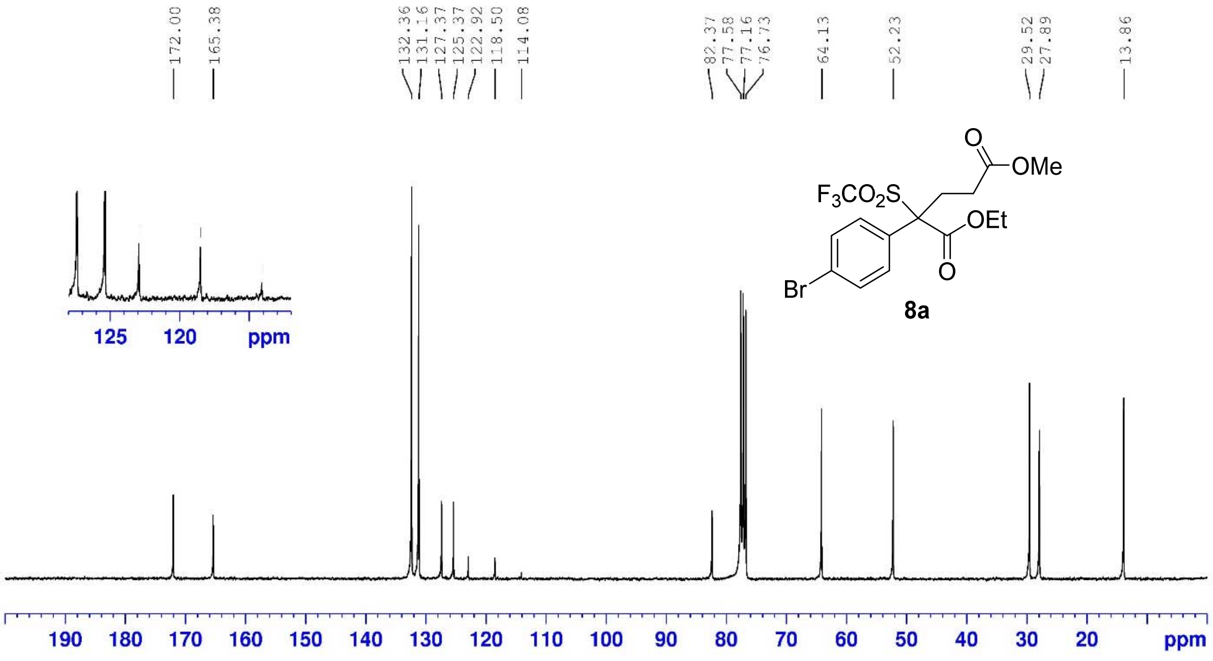

^{19}F NMR in $CDCl_3$ (376 MHz)

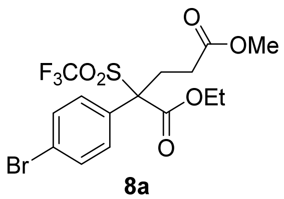

8a

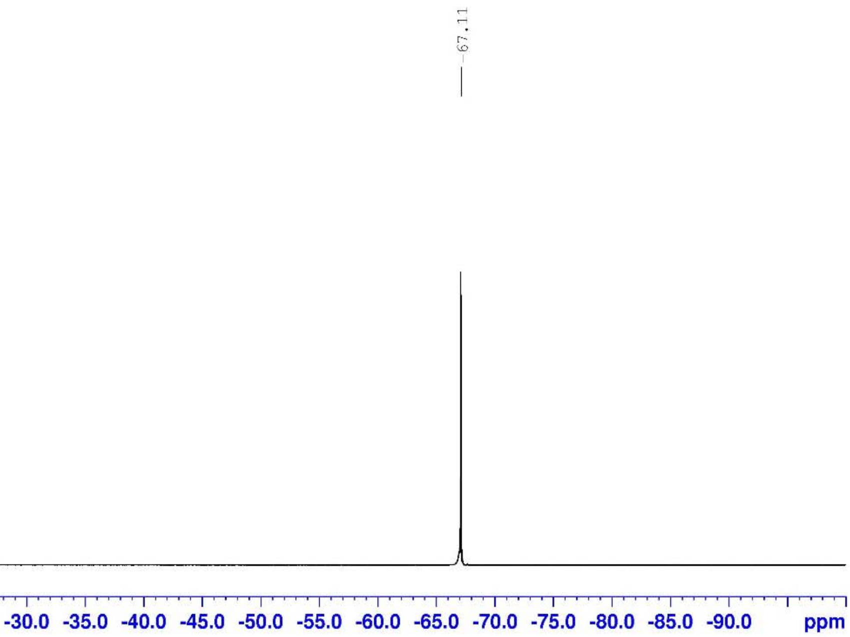

^{1}H NMR in $CDCl_3$ (400 MHz)

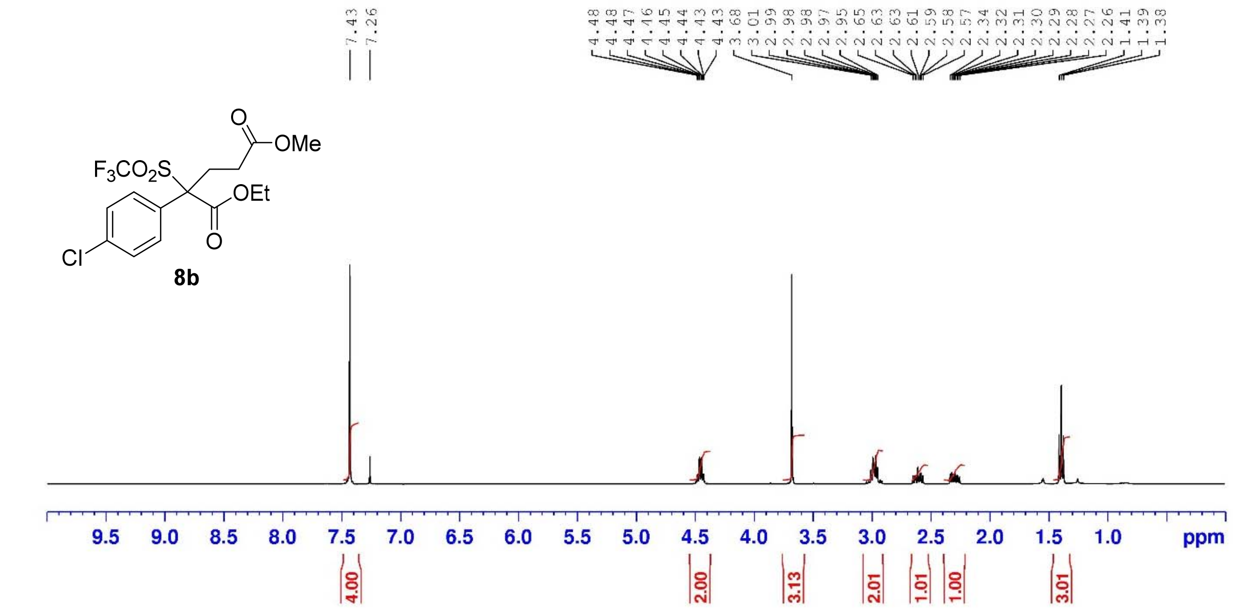

^{13}C NMR in $CDCl_3$ (100 MHz)

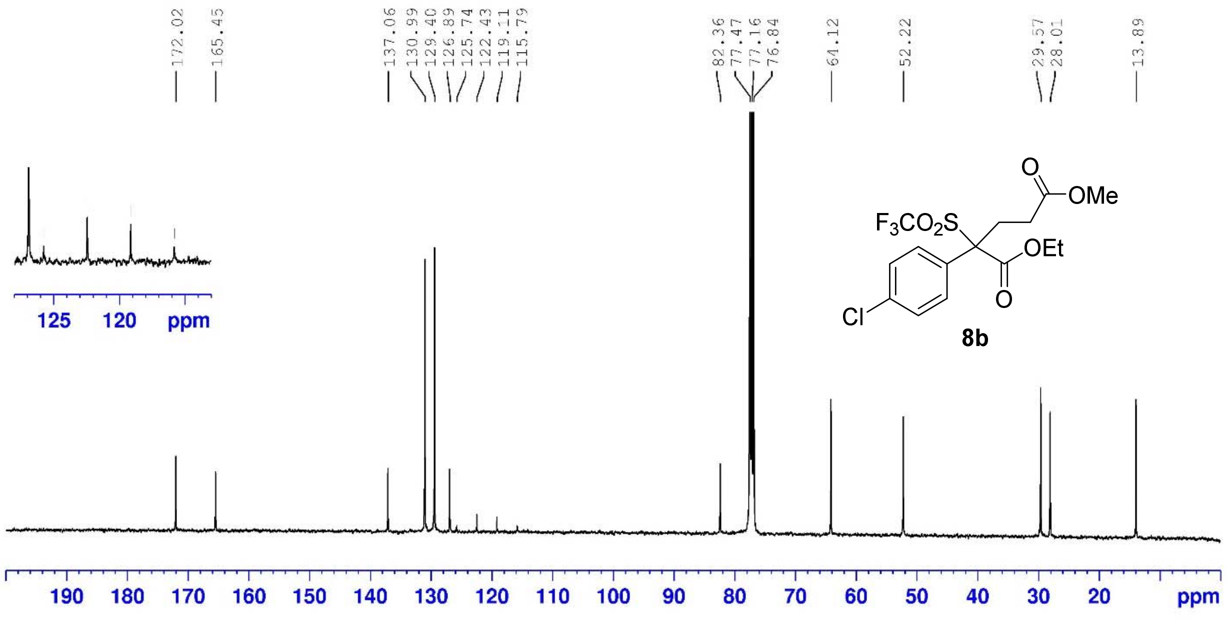

^{19}F NMR in $CDCl_3$ (376 MHz)

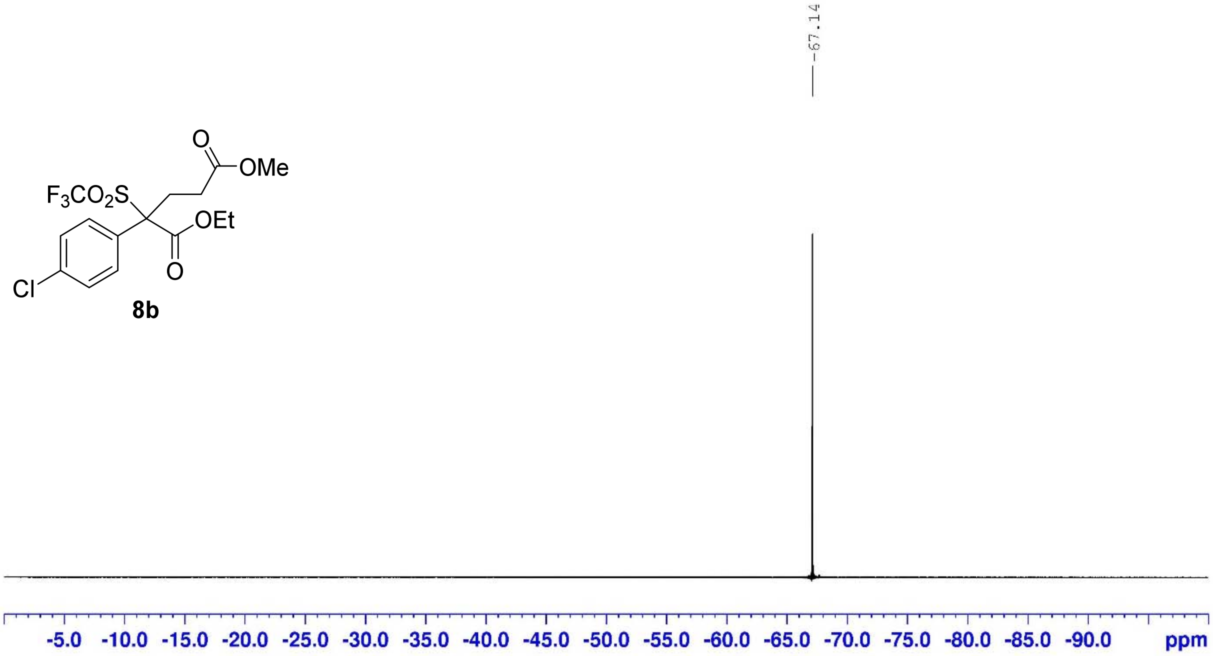

^{1}H NMR in $CDCl_3$ (600 MHz)

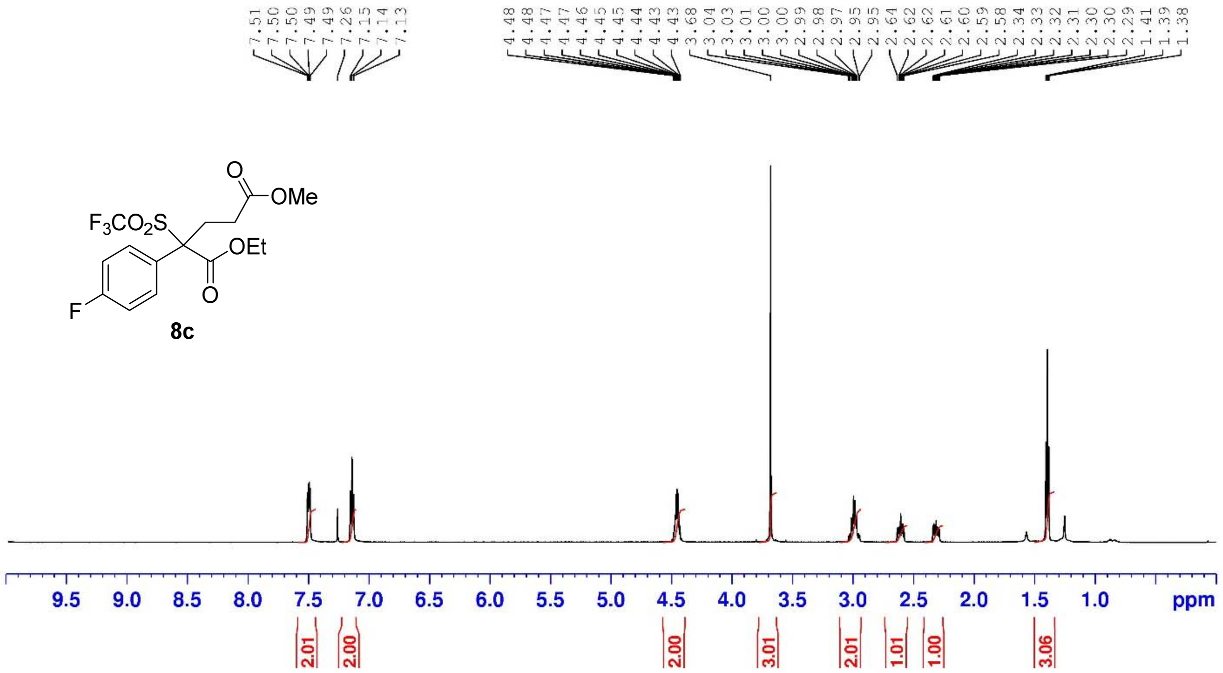

^{13}C NMR in $CDCl_3$ (150 MHz)

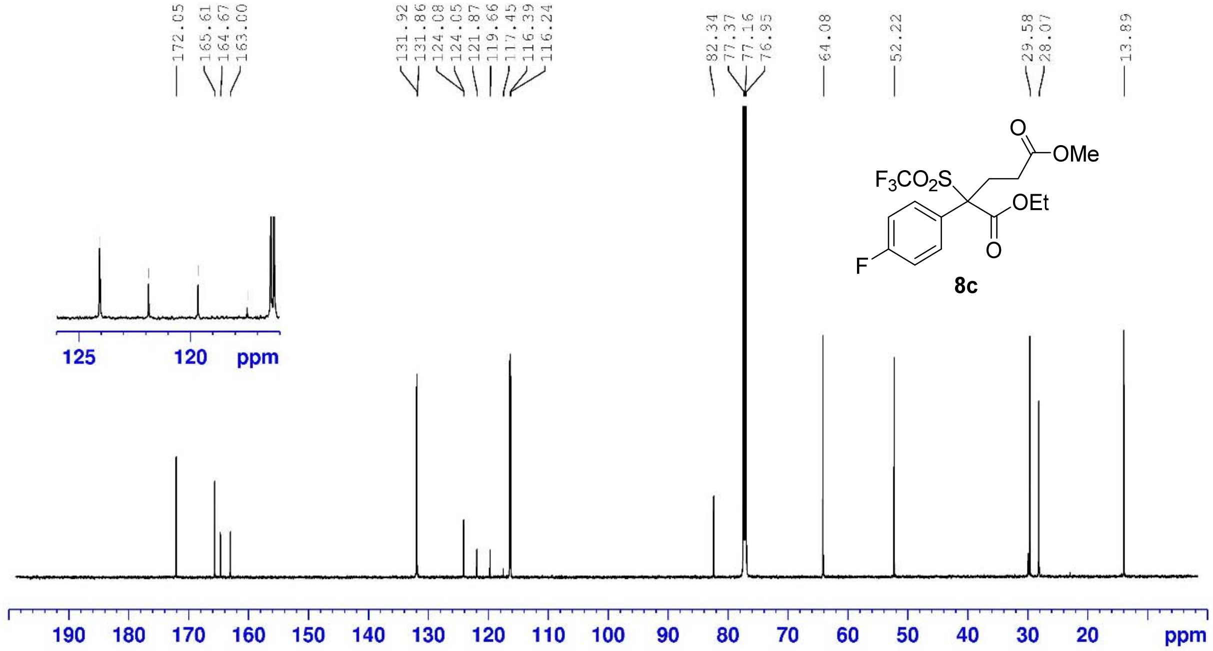

^{19}F NMR in $CDCl_3$ (376 MHz)

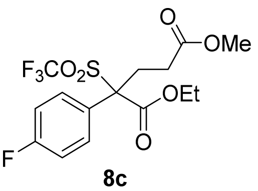

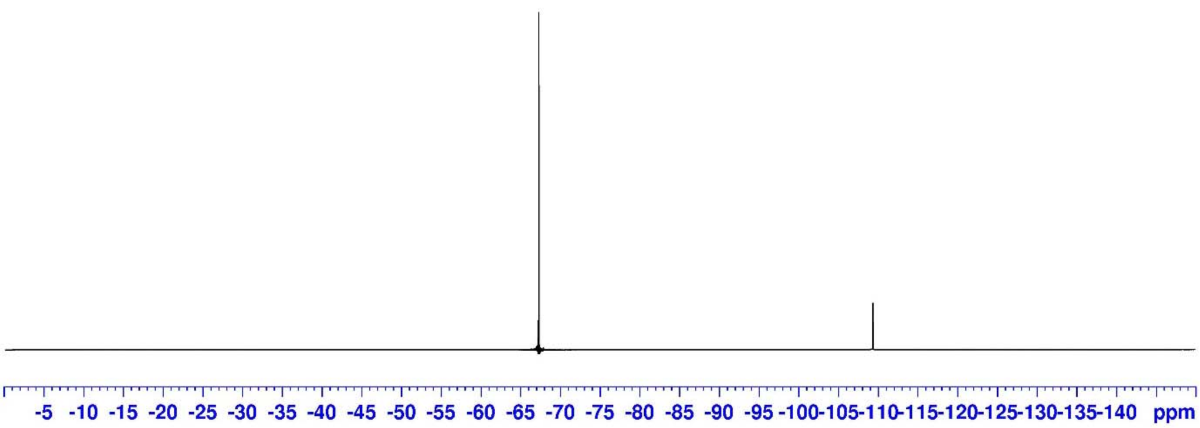

^{1}H NMR in $CDCl_3$ (300 MHz)

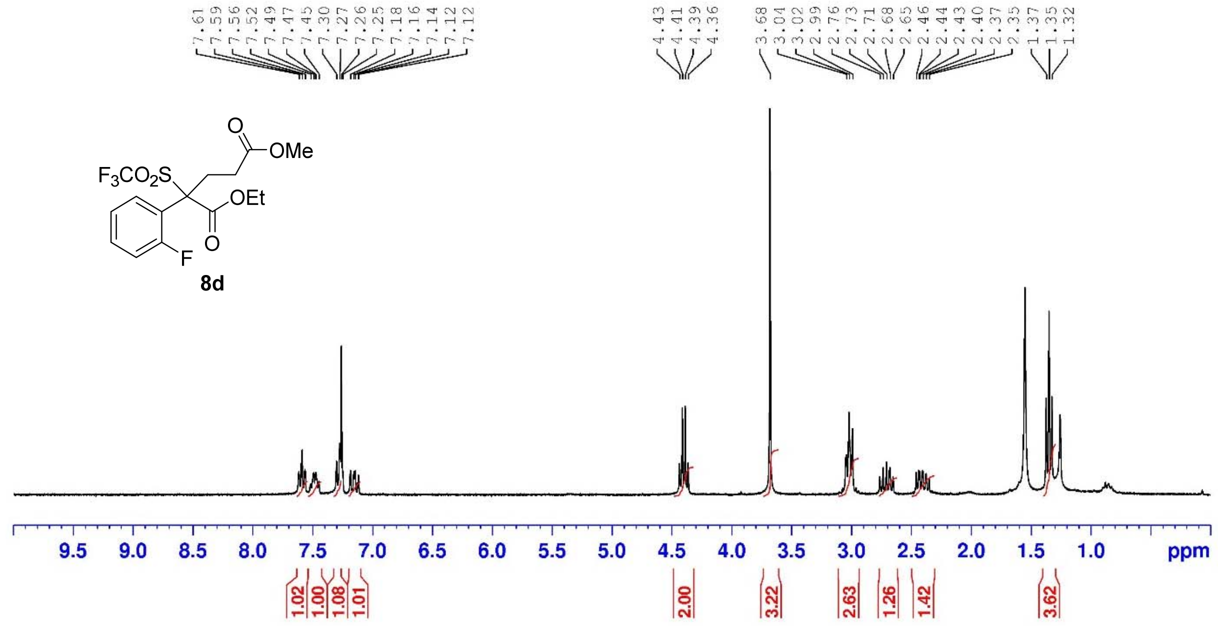

^{13}C NMR in $CDCl_3$ (62.5 MHz)

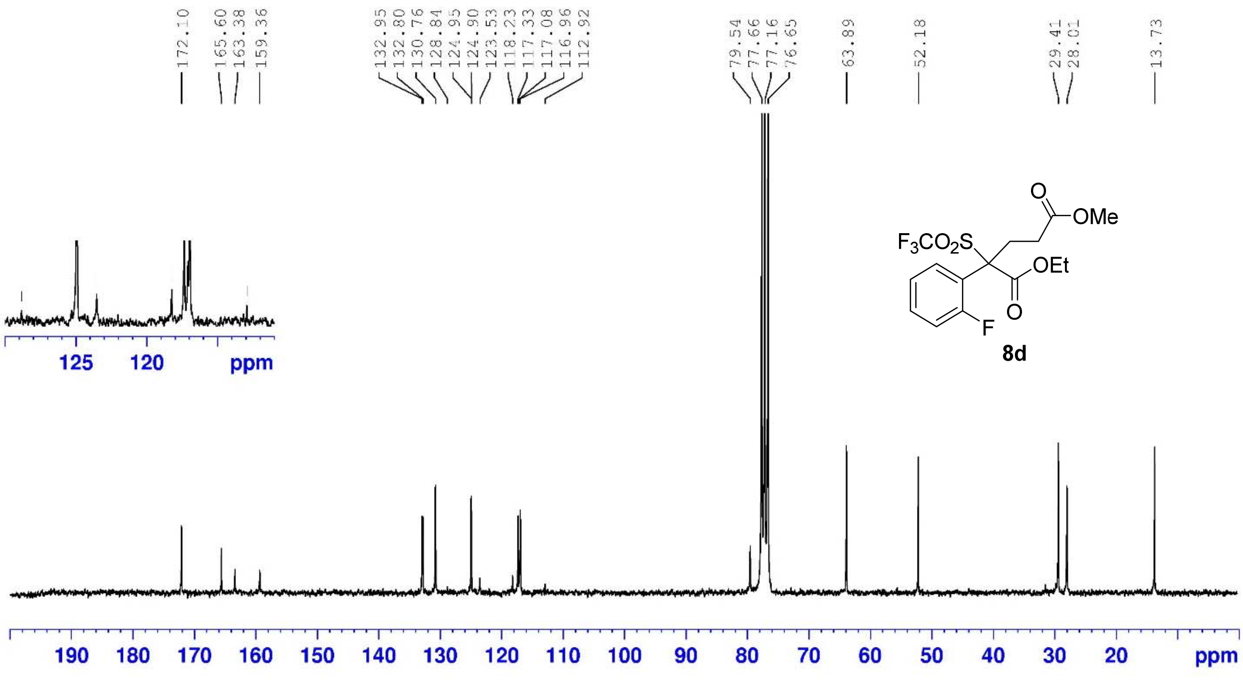

^{19}F NMR in $CDCl_3$ (376 MHz)

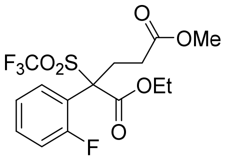

8d

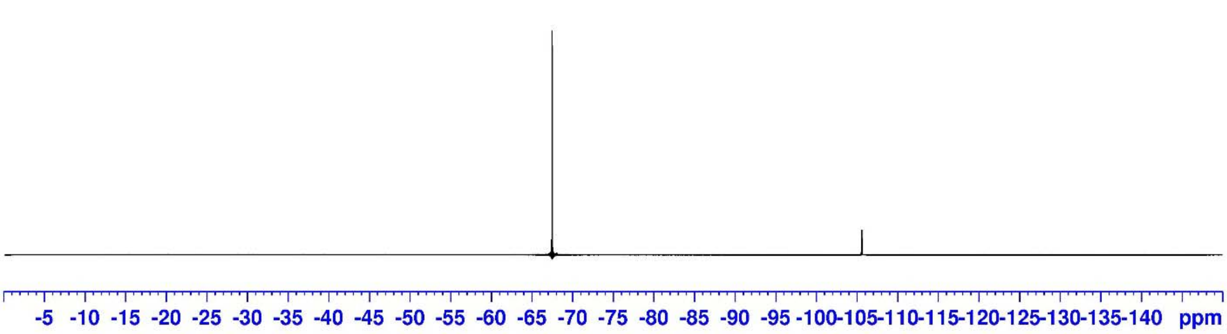

^{1}H NMR in $CDCl_3$ (400 MHz)

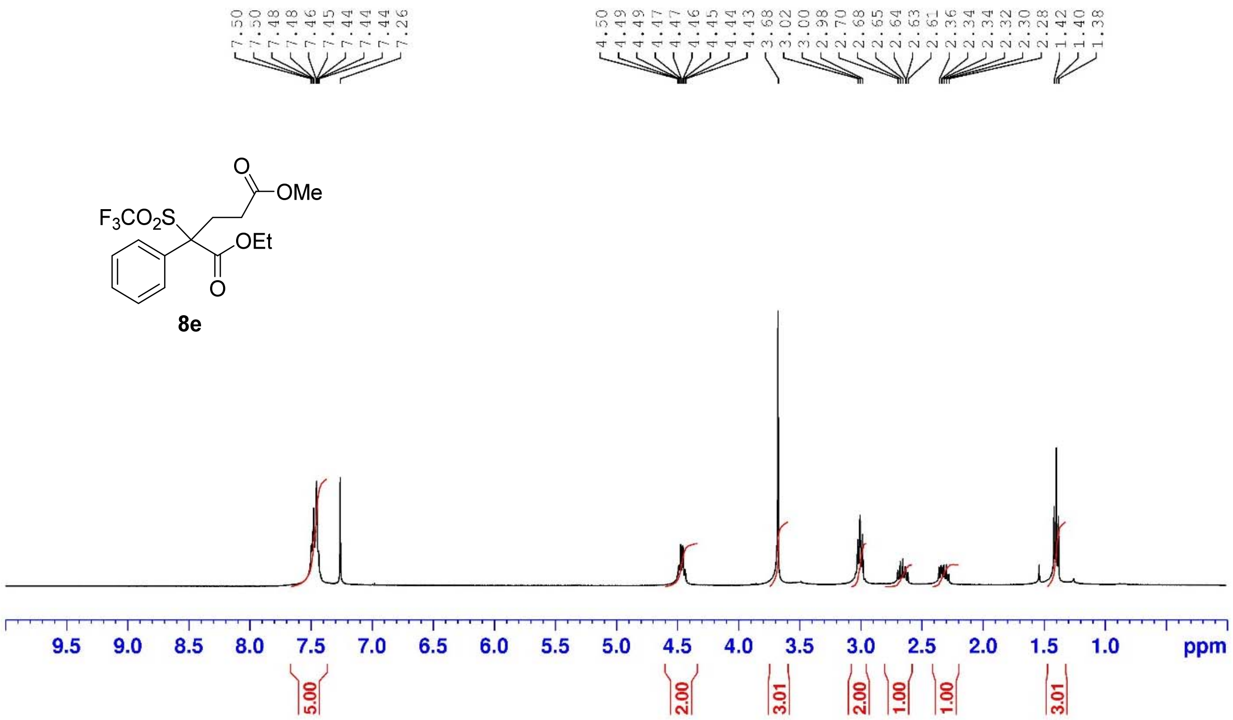

^{13}C NMR in $CDCl_3$ (100 MHz)

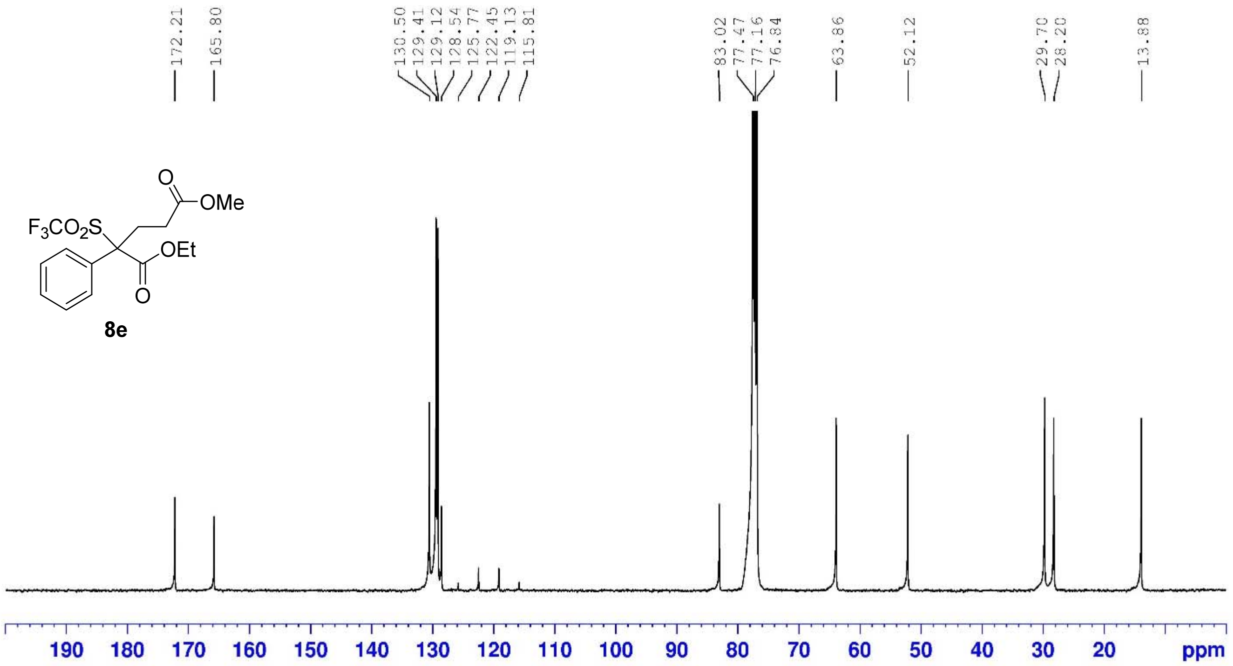

^{19}F NMR in $CDCl_3$ (376 MHz)

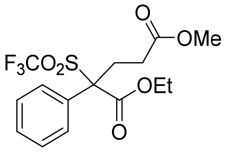

8e

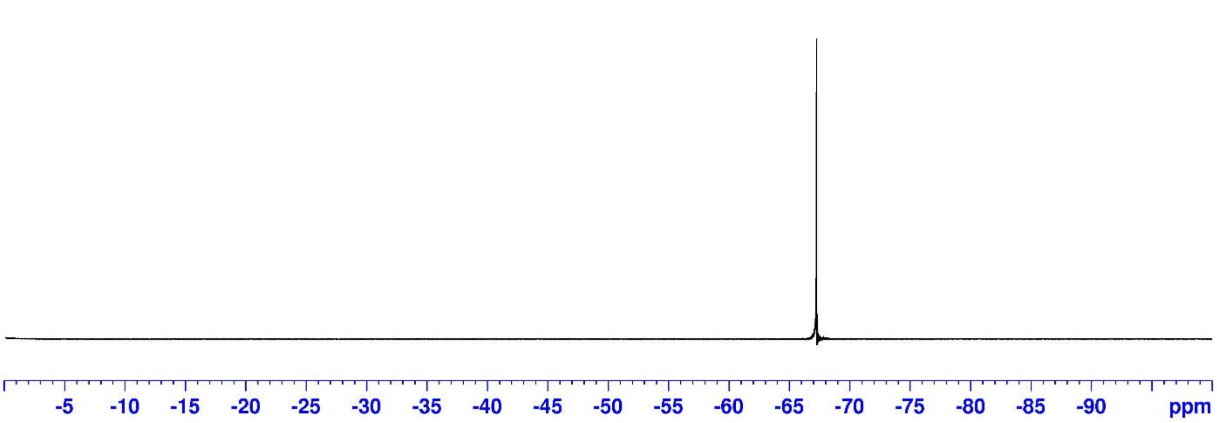

^{1}H NMR in $CDCl_3$ (400 MHz)

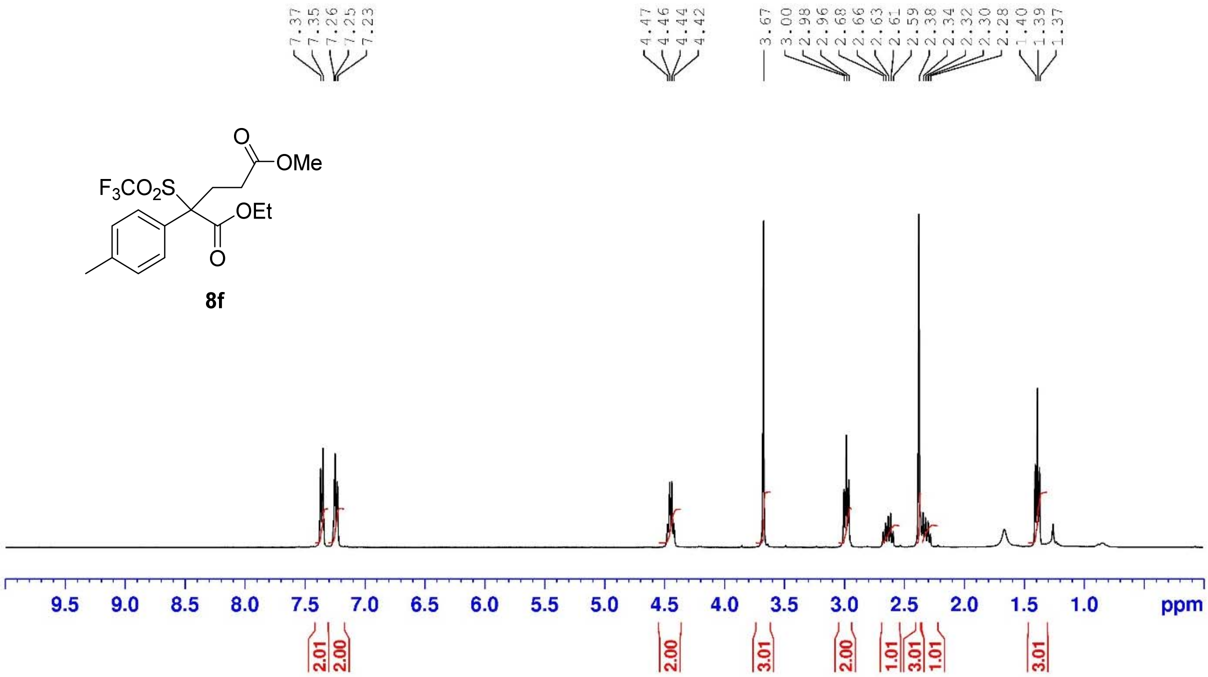

^{13}C NMR in $CDCl_3$ (62.5 MHz)

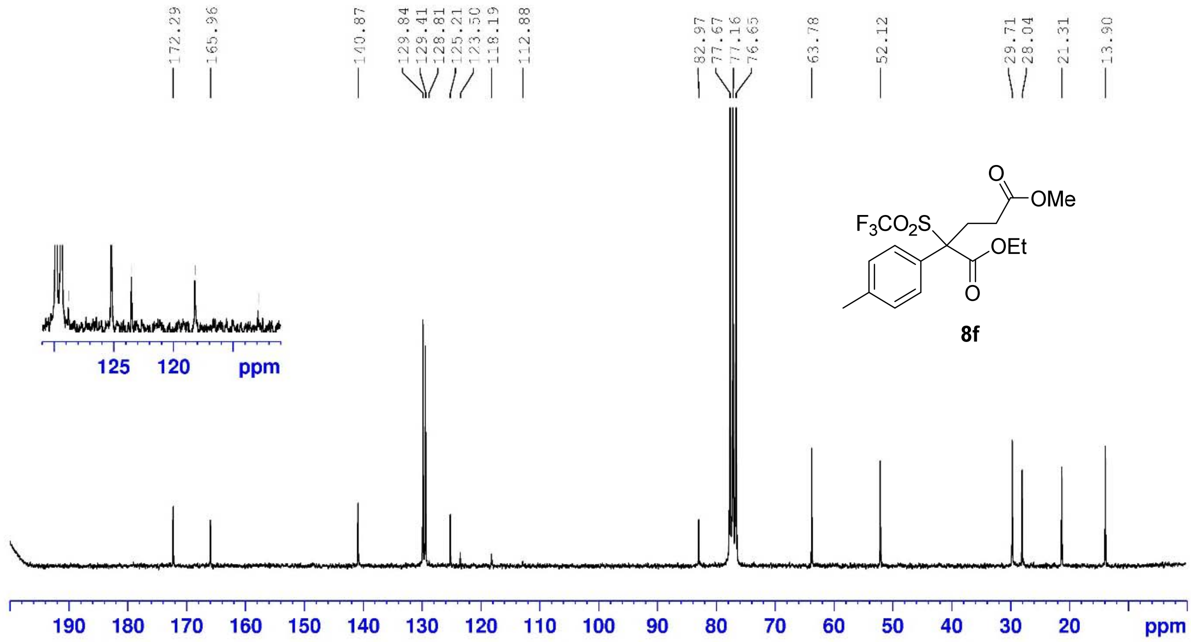

^{19}F NMR in $CDCl_3$ (376 MHz)

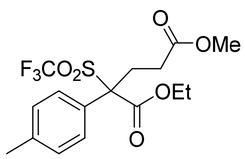

8f

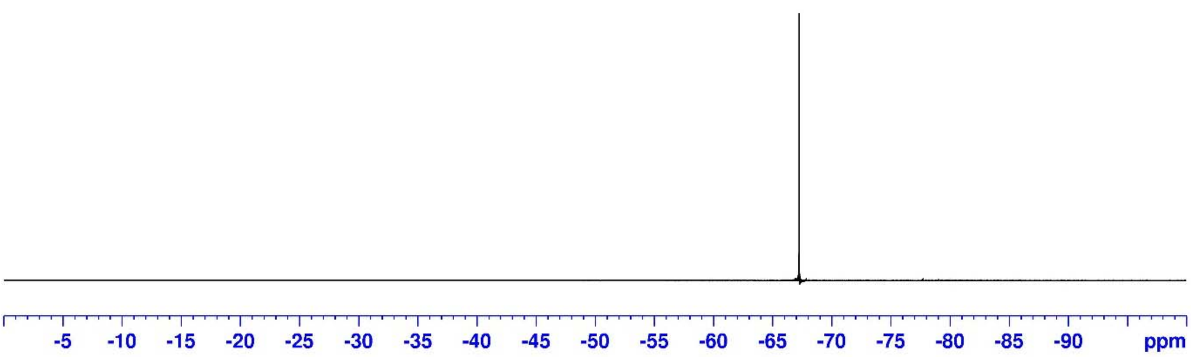

^{1}H NMR in $CDCl_3$ (400 MHz)

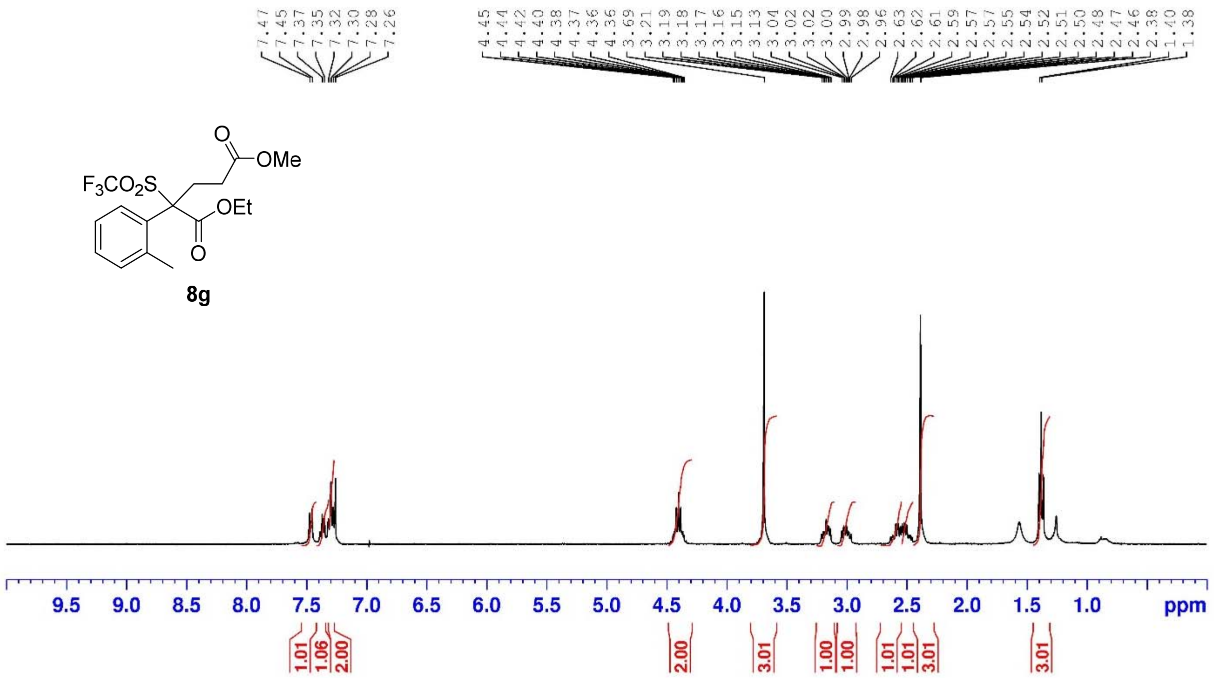

^{13}C NMR in $CDCl_3$ (75 MHz)

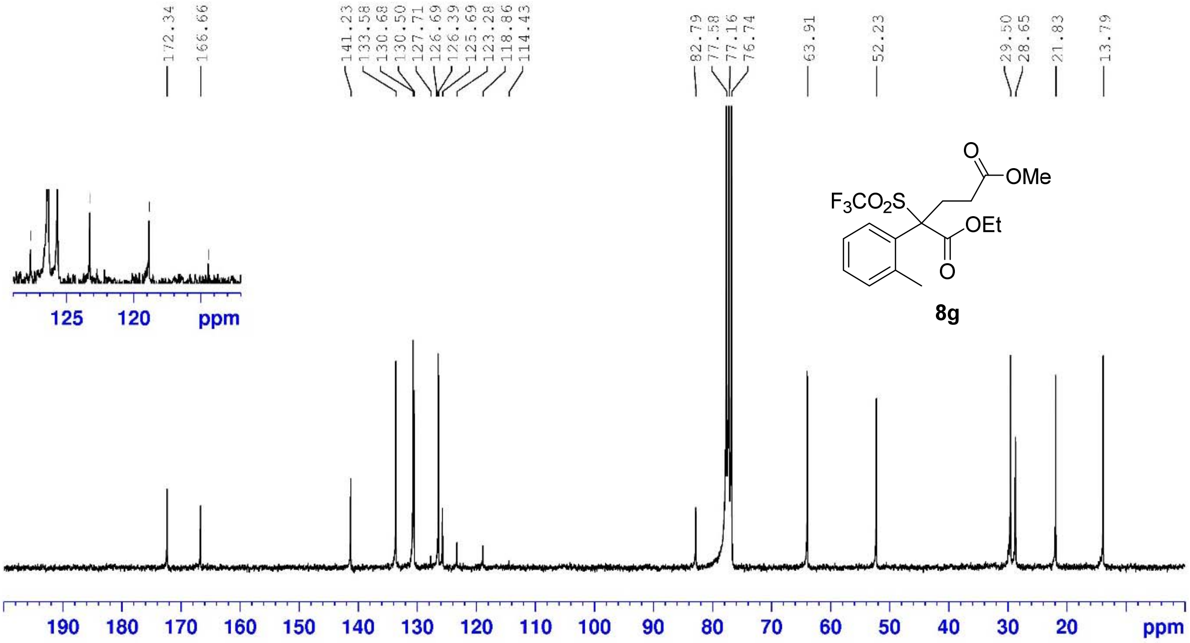

^{19}F NMR in $CDCl_3$ (376 MHz)

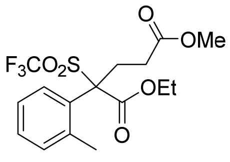

8g

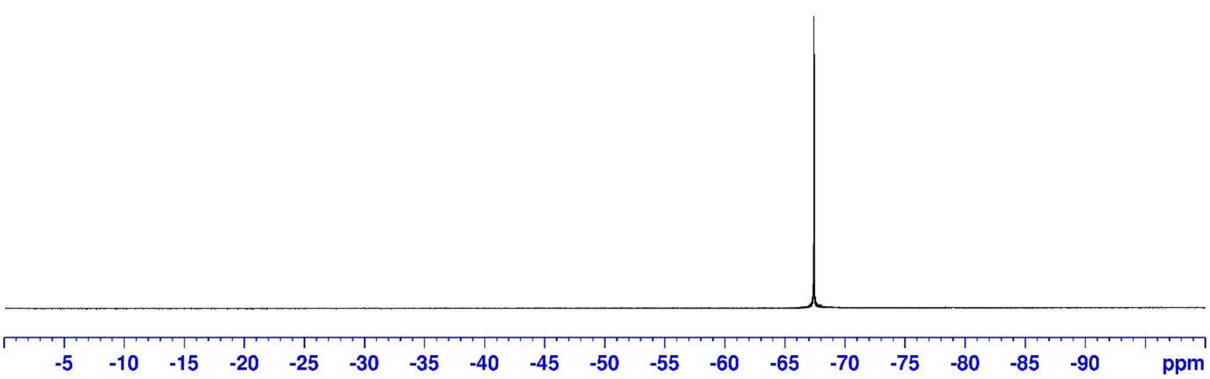

^{1}H NMR in $CDCl_3$ (400 MHz)

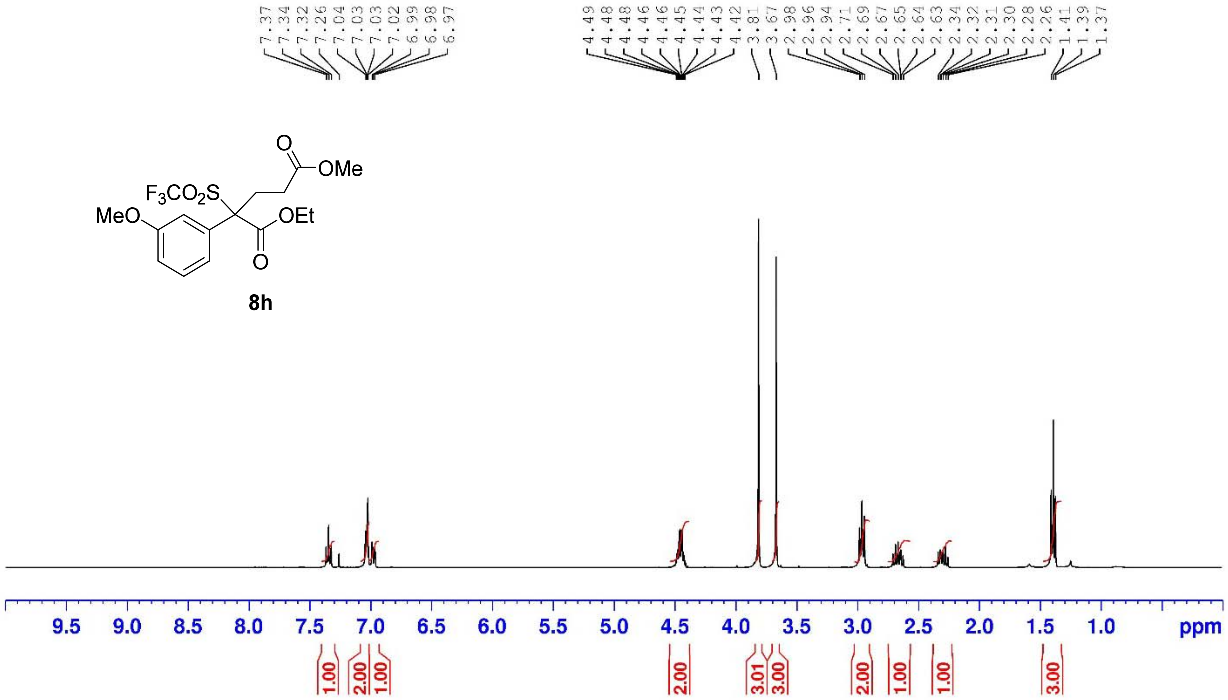

^{13}C NMR in $CDCl_3$ (75 MHz)

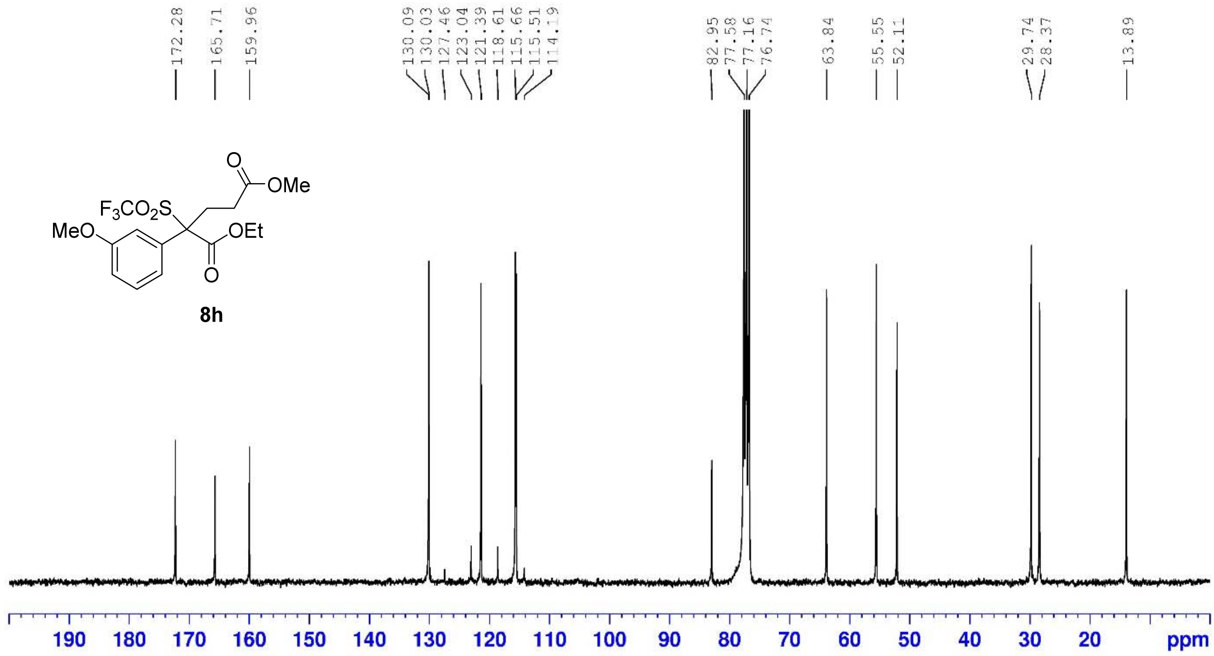

^{19}F NMR in $CDCl_3$ (376 MHz)

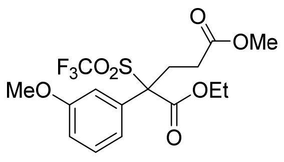

8h

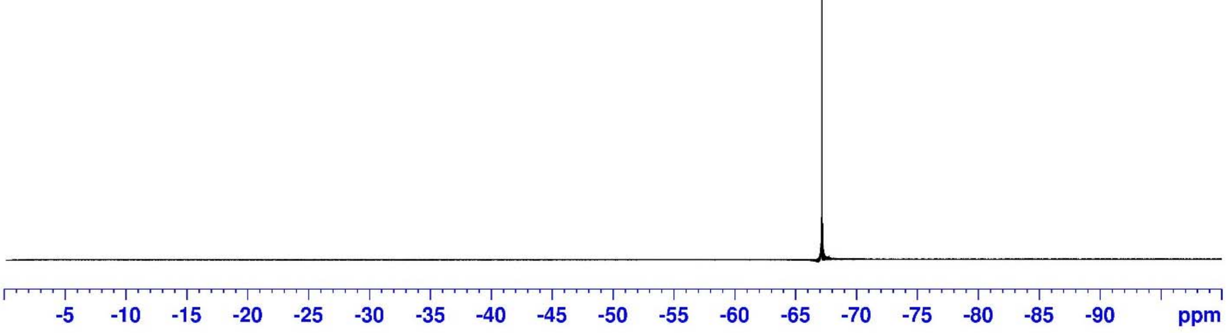

^{1}H NMR in $CDCl_3$ (400 MHz)

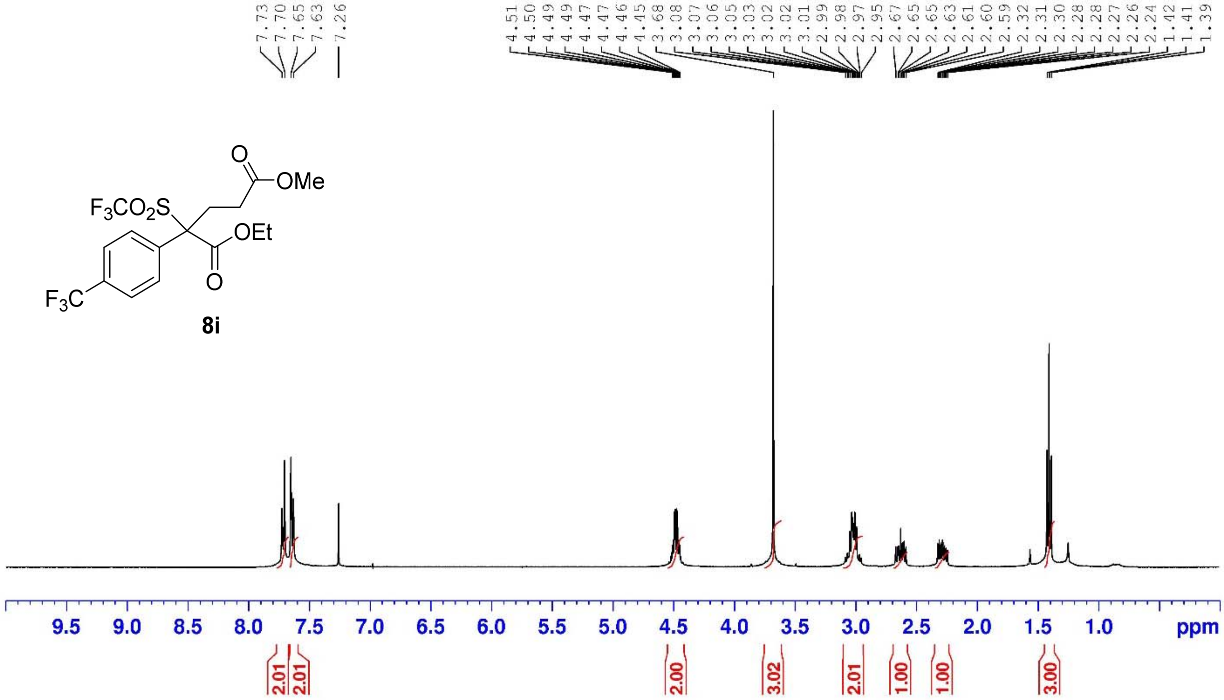

^{13}C NMR in $CDCl_3$ (100 MHz)

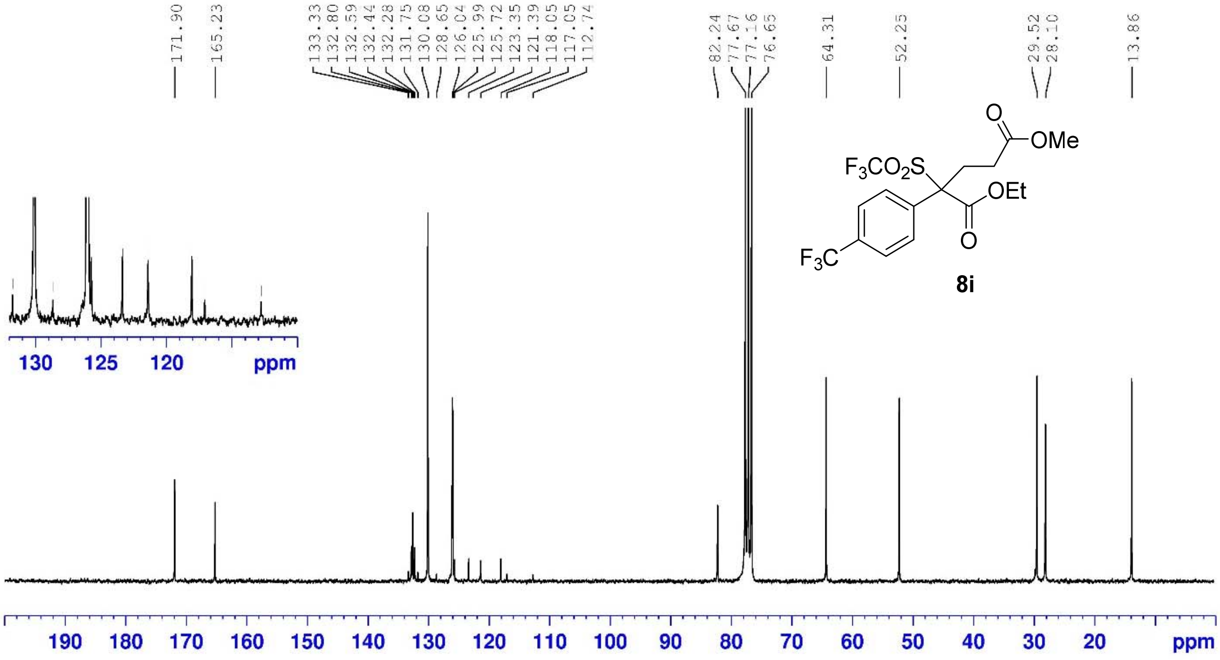

^{19}F NMR in $CDCl_3$ (376 MHz)

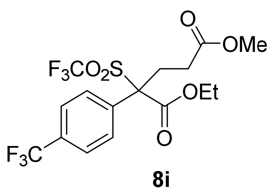

8i

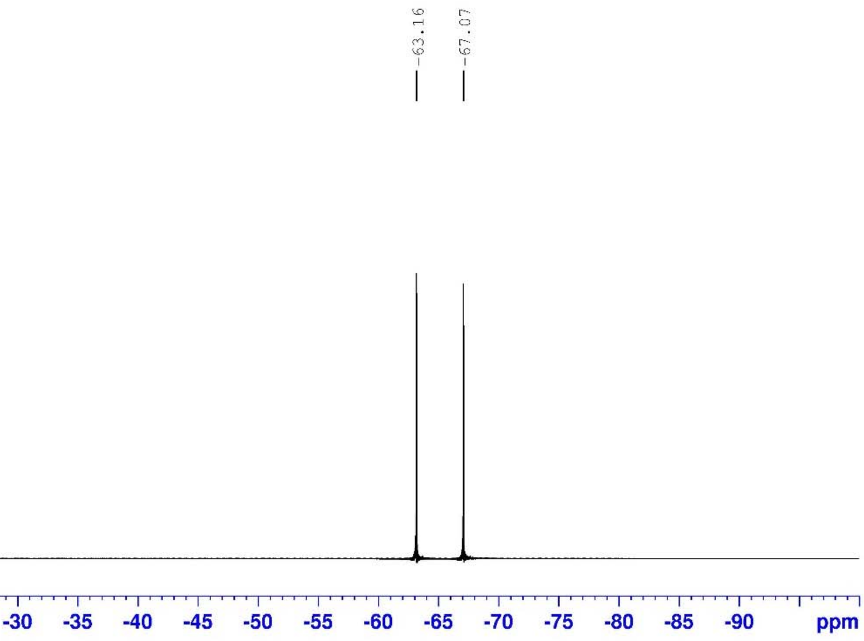

^{1}H NMR in $CDCl_3$ (300 MHz)

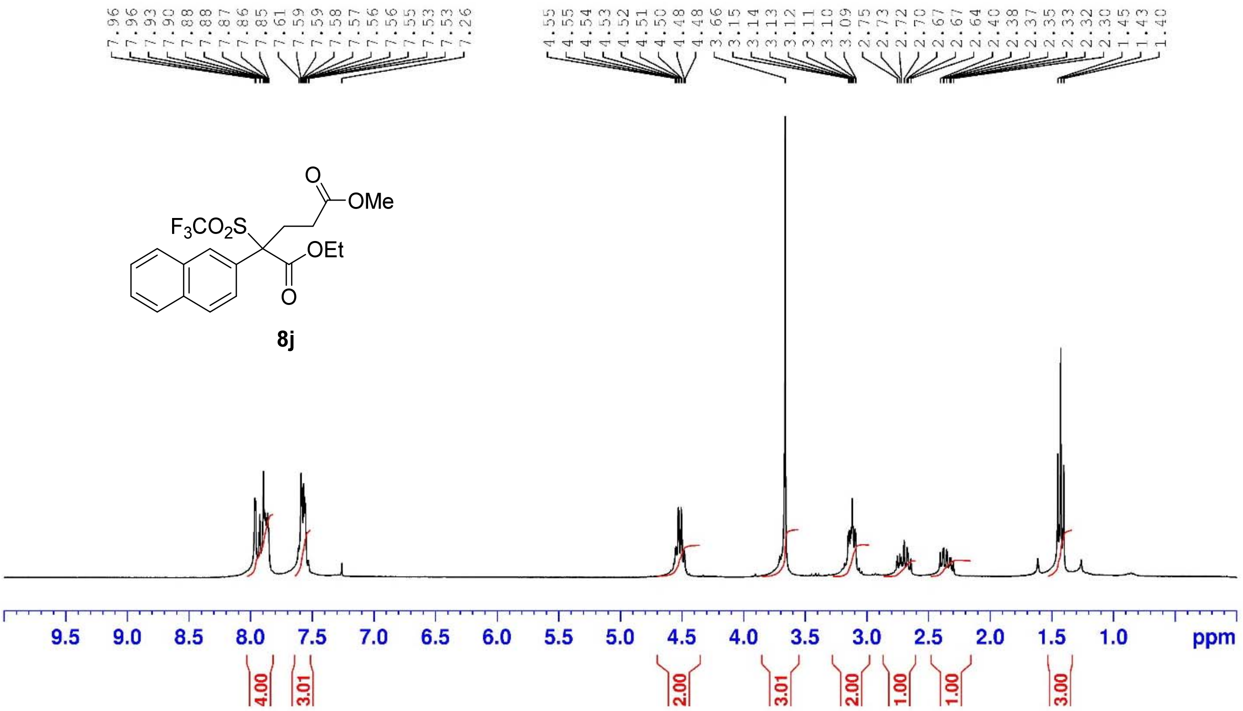

^{13}C NMR in $CDCl_3$ (100 MHz)

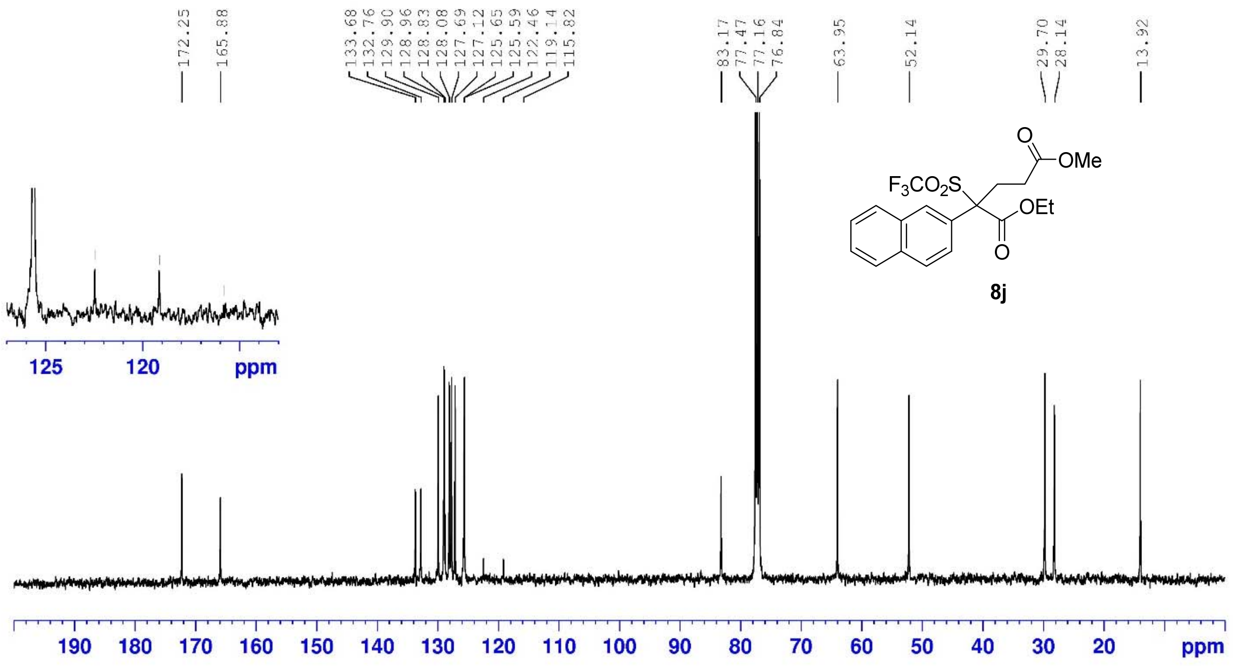

^{19}F NMR in $CDCl_3$ (376 MHz)

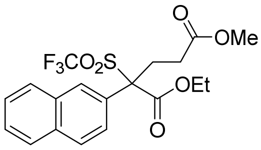

8j

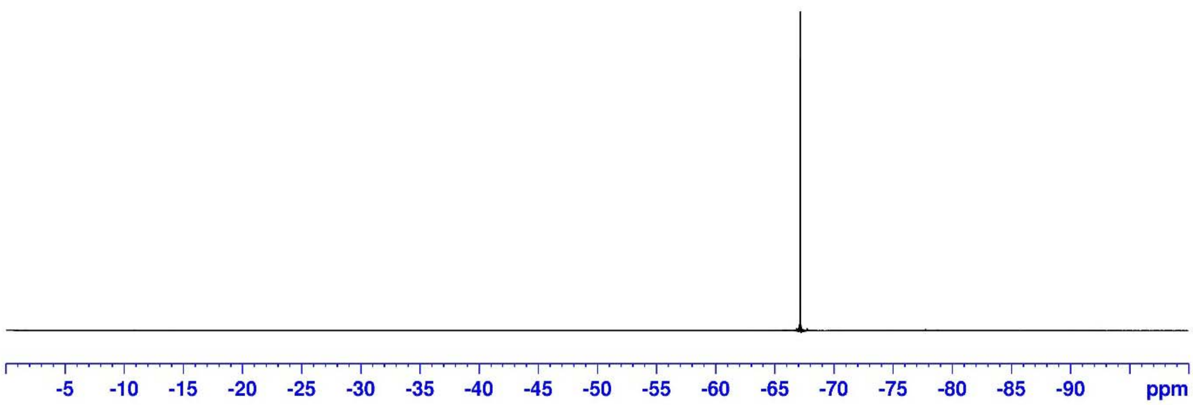

^{1}H NMR in $CDCl_3$ (400 MHz)

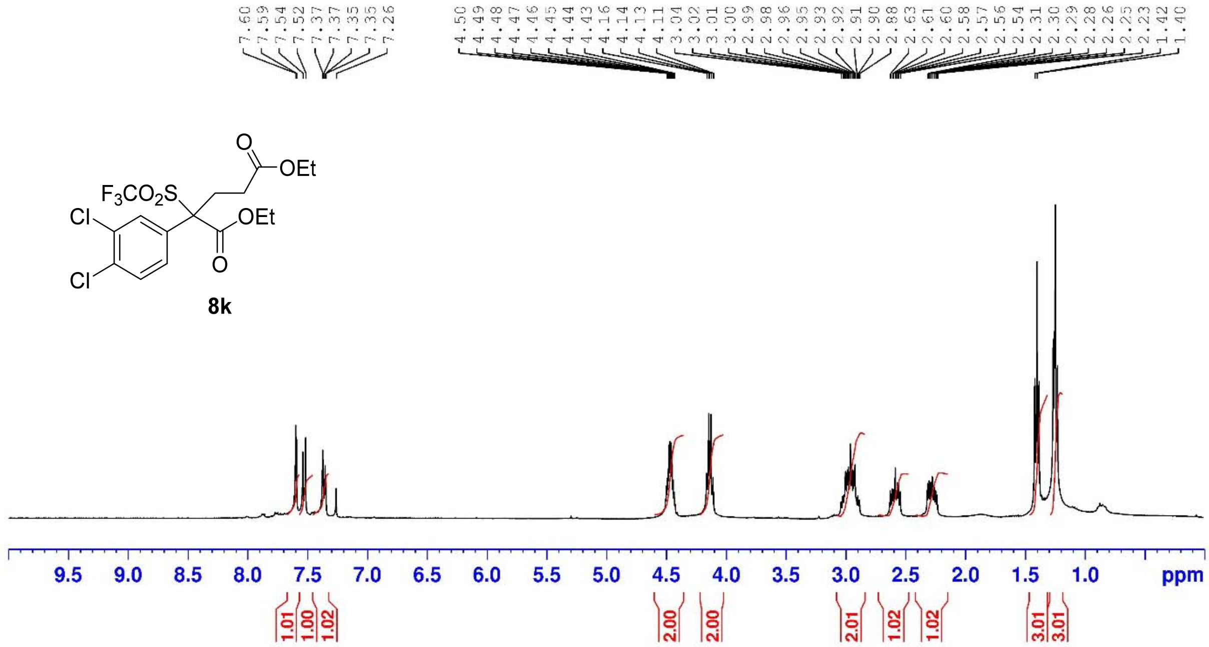

^{13}C NMR in $CDCl_3$ (100 MHz)

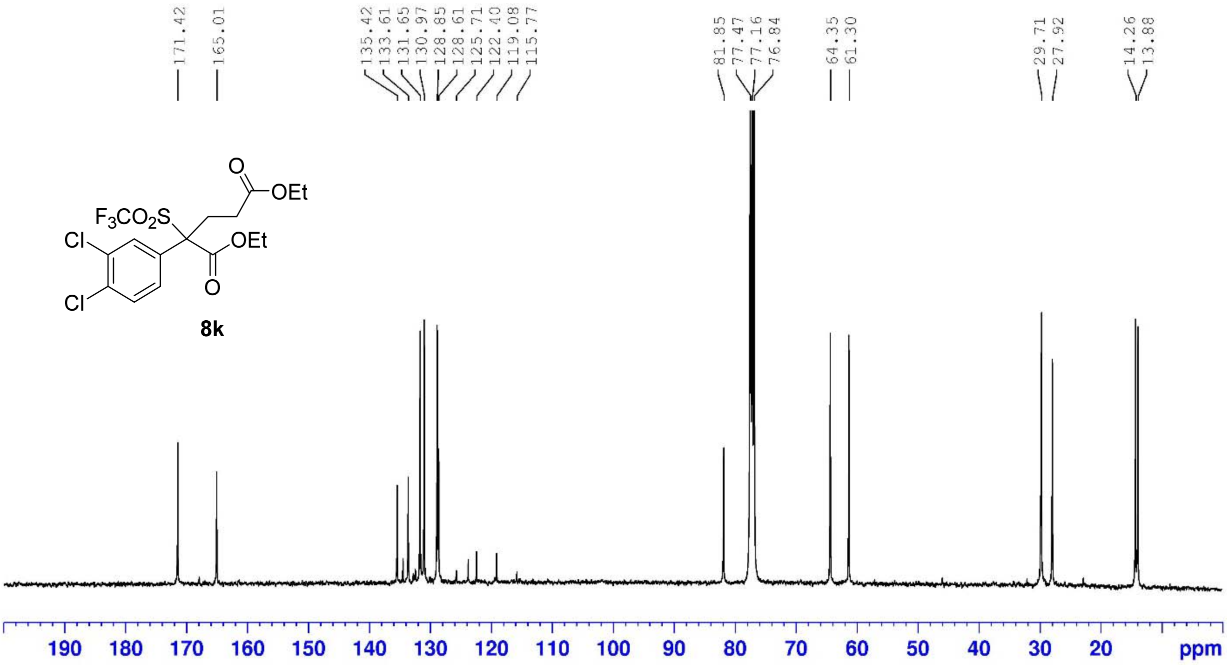

^{19}F NMR in $CDCl_3$ (376 MHz)

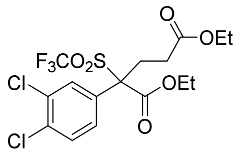

8k

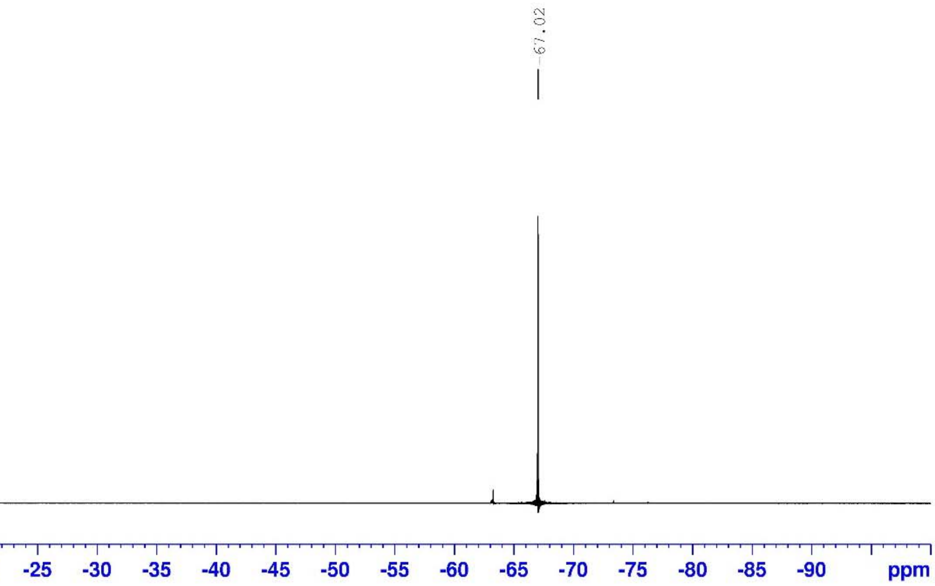

^{1}H NMR in $CDCl_3$ (300 MHz)

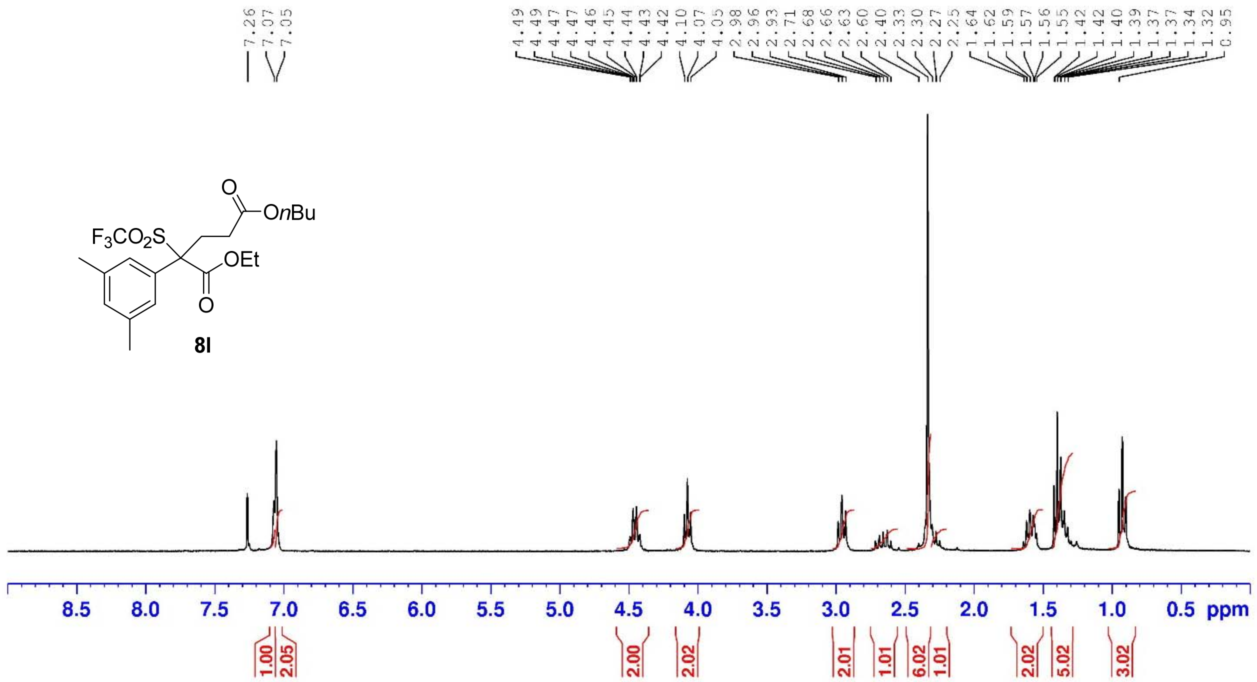

^{13}C NMR in $CDCl_3$ (75 MHz)

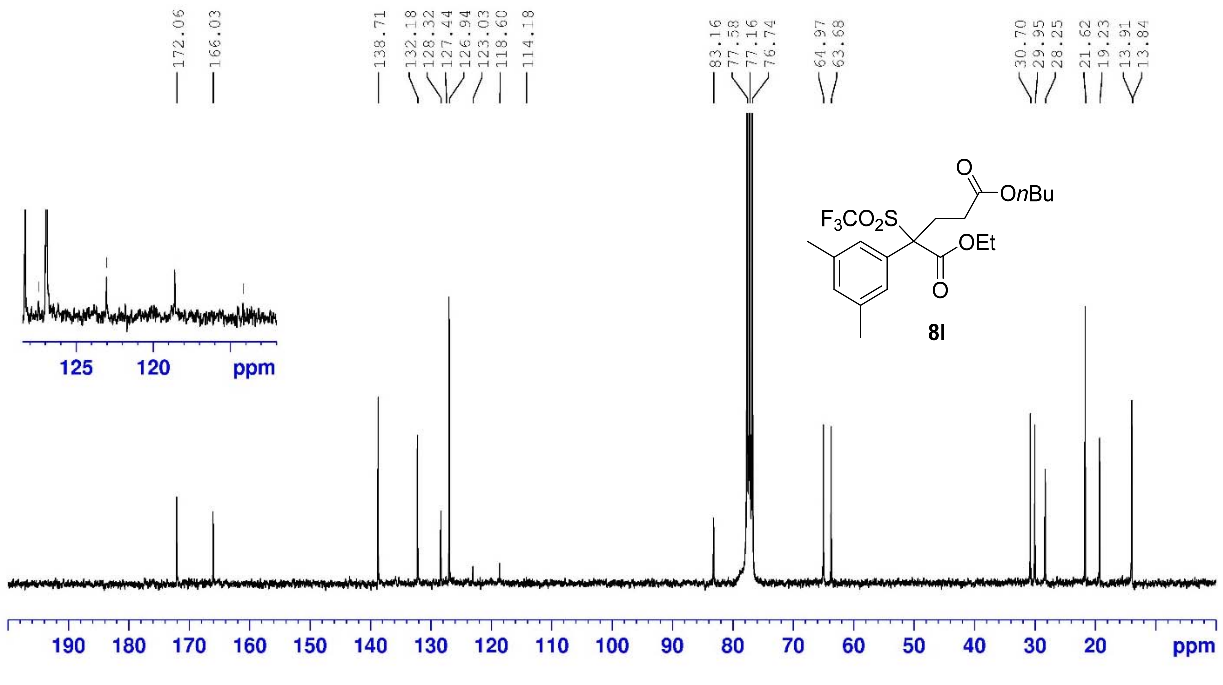

^{19}F NMR in $CDCl_3$ (376 MHz)

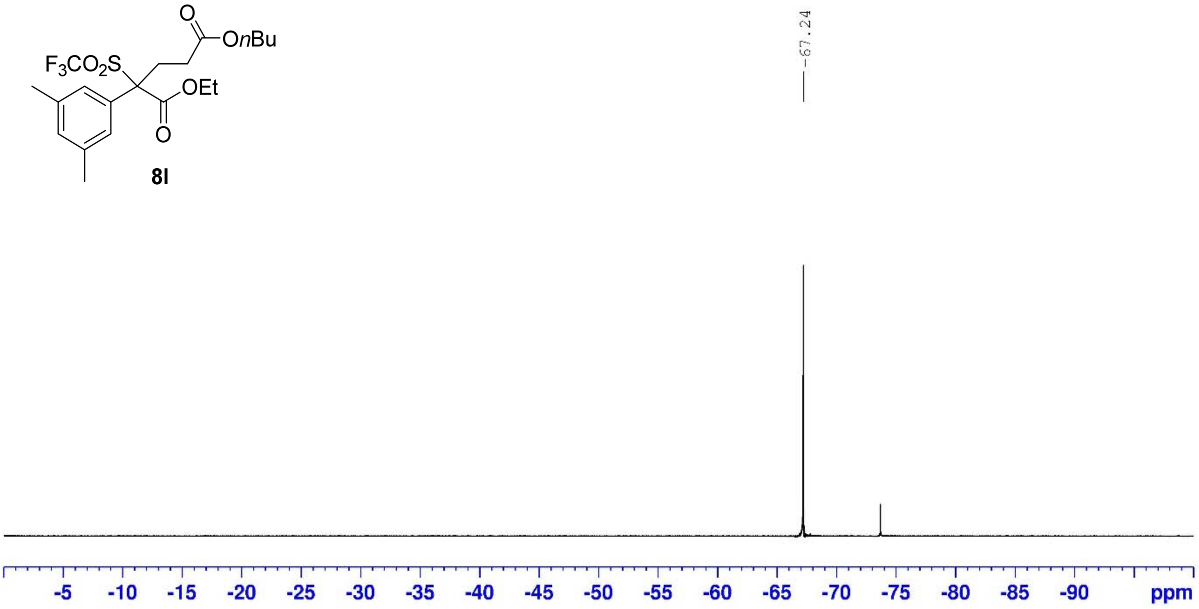

8l

^{1}H NMR in $CDCl_3$ (600 MHz)

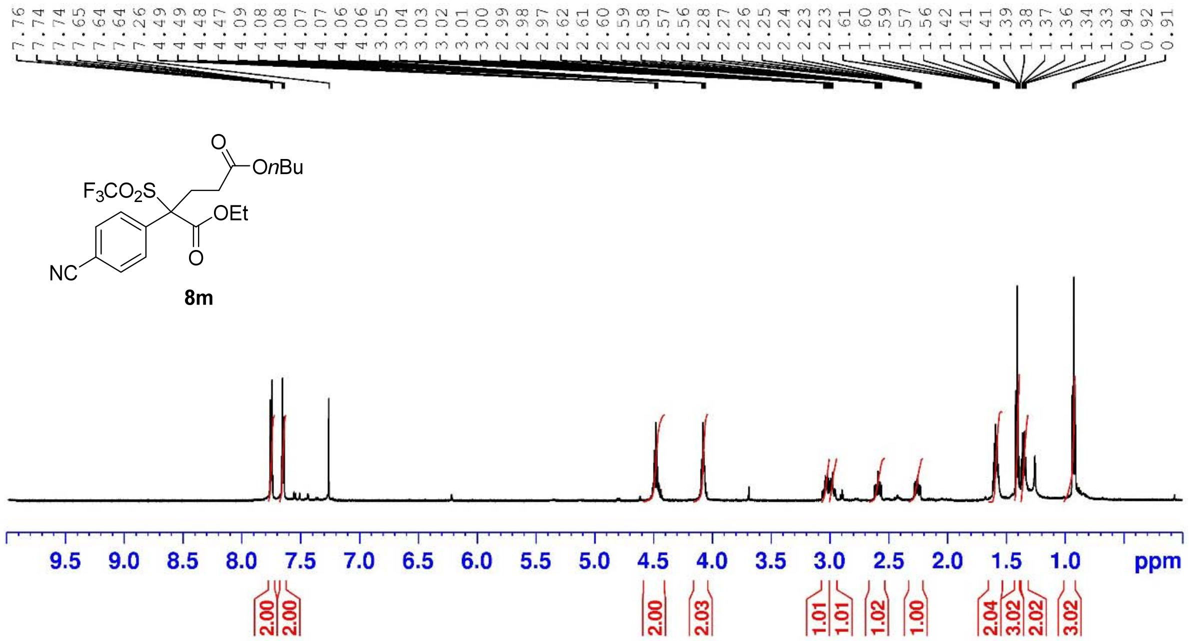

^{13}C NMR in $CDCl_3$ (150 MHz)

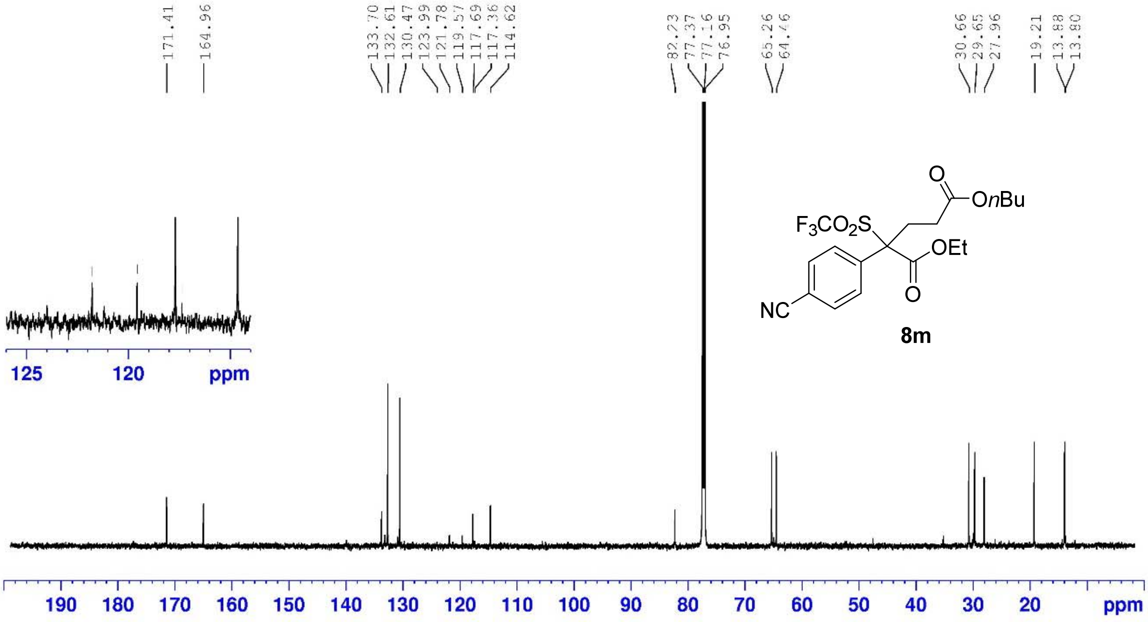

^{19}F NMR in $CDCl_3$ (376 MHz)

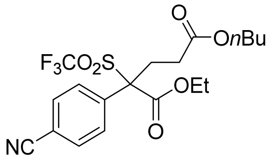

8m

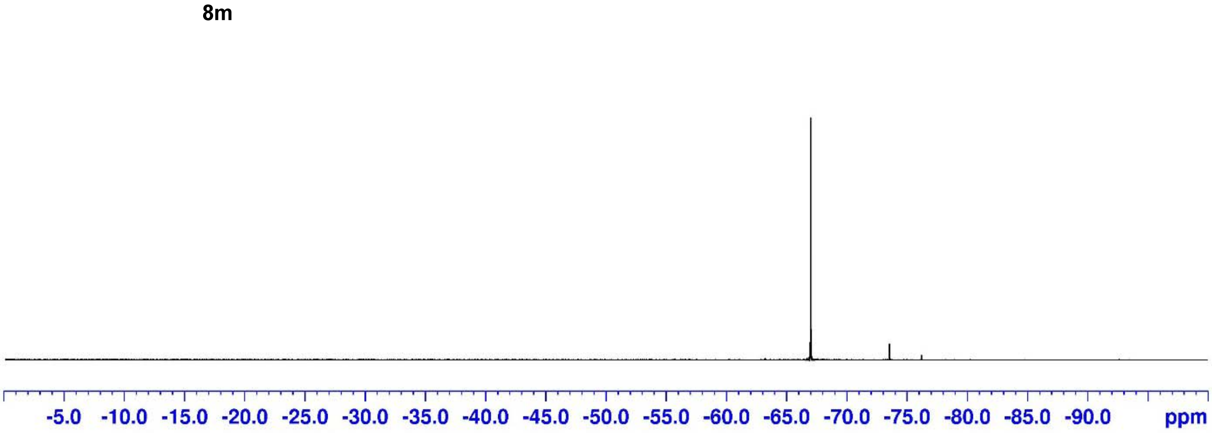

^{1}H NMR in $CDCl_3$ (600 MHz)

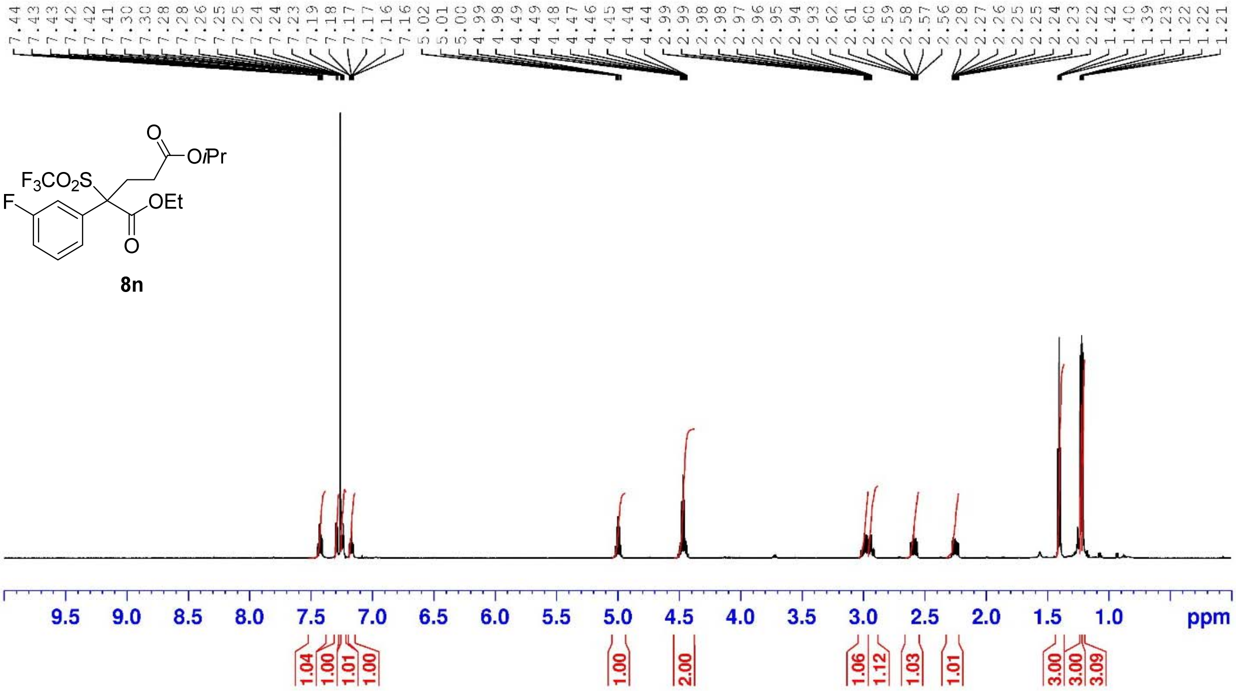

^{13}C NMR in $CDCl_3$ (150 MHz)

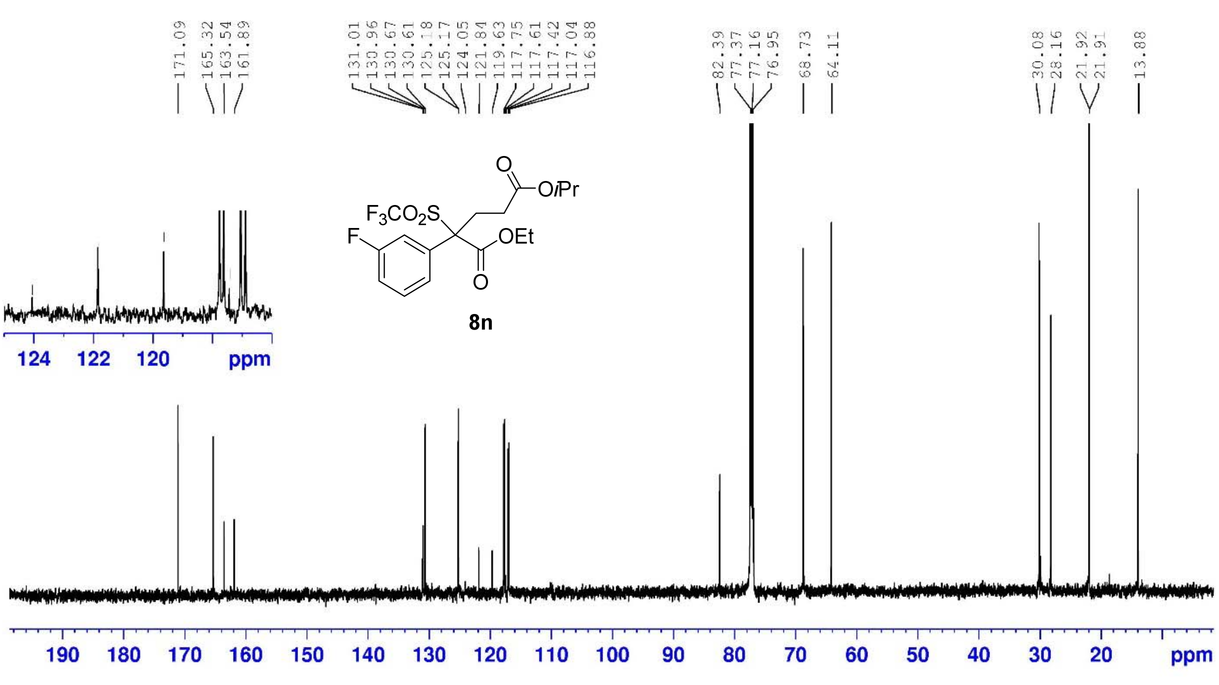

^{19}F NMR in $CDCl_3$ (376 MHz)

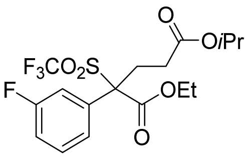

8n

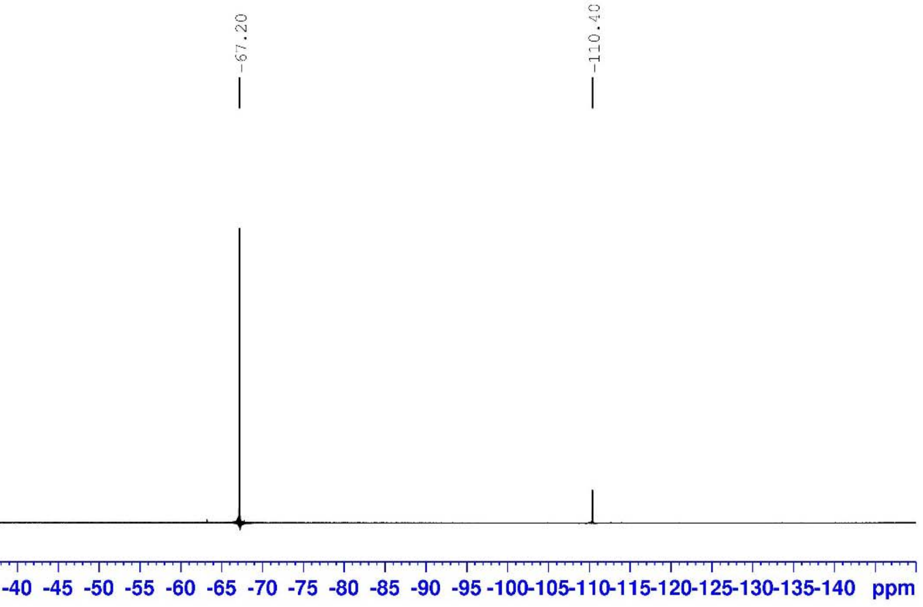

^{1}H NMR in $CDCl_3$ (300 MHz)

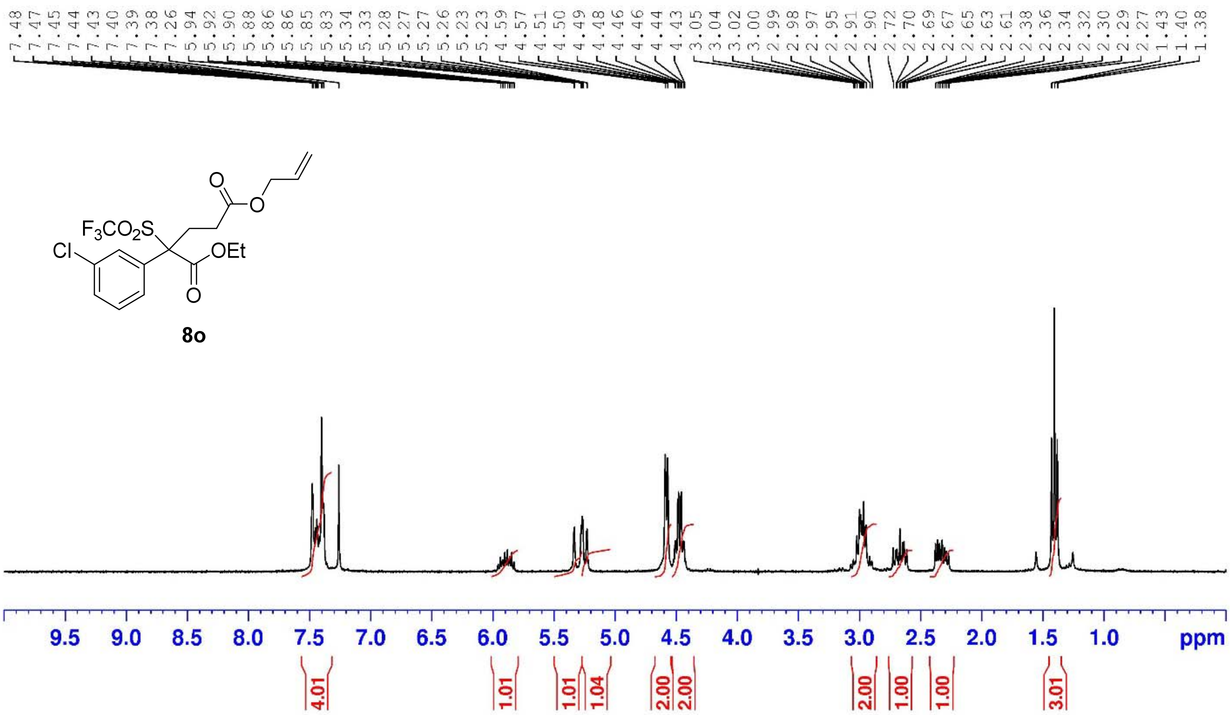

^{13}C NMR in $CDCl_3$ (75 MHz)

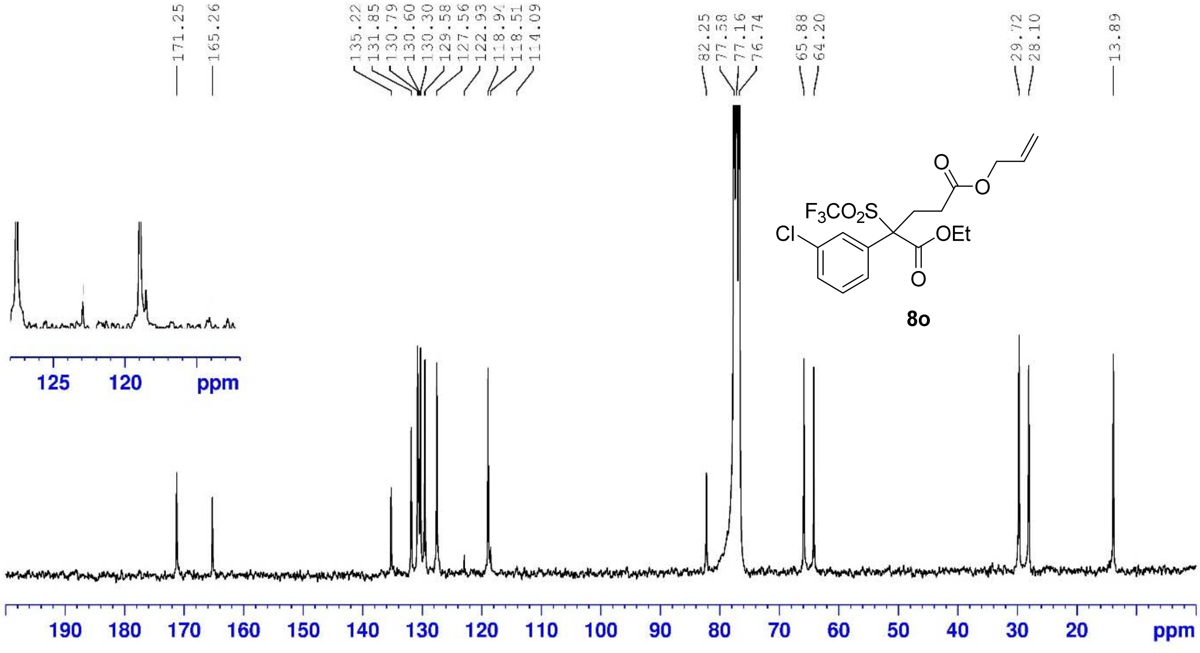

^{19}F NMR in $CDCl_3$ (376 MHz)

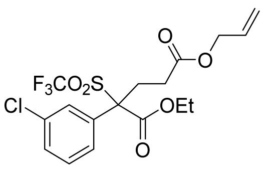

8o

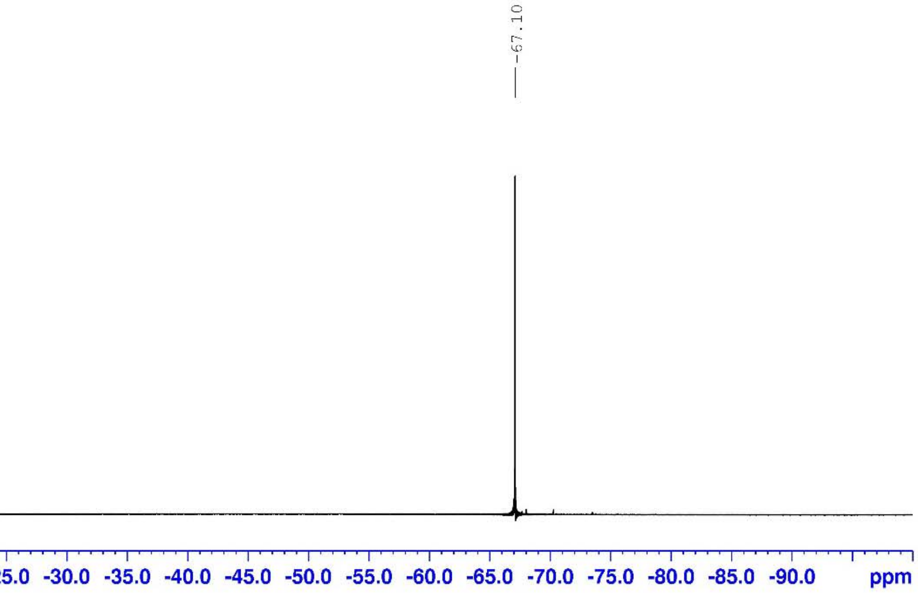

^{1}H NMR in $CDCl_3$ (600 MHz)

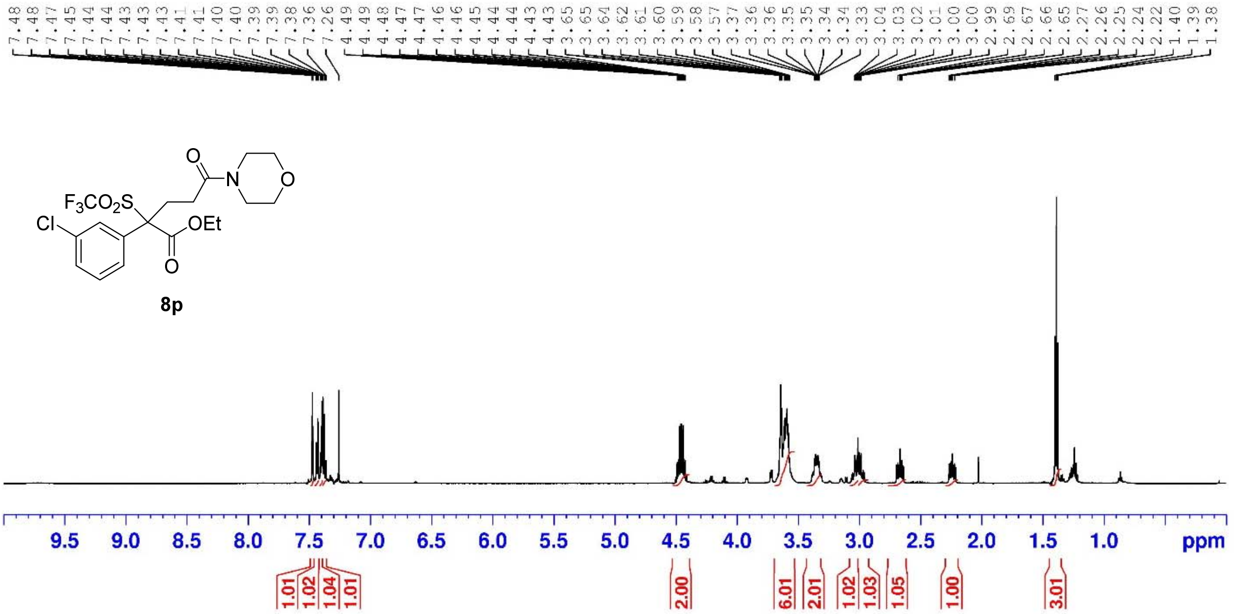

^{13}C NMR in $CDCl_3$ (150 MHz)

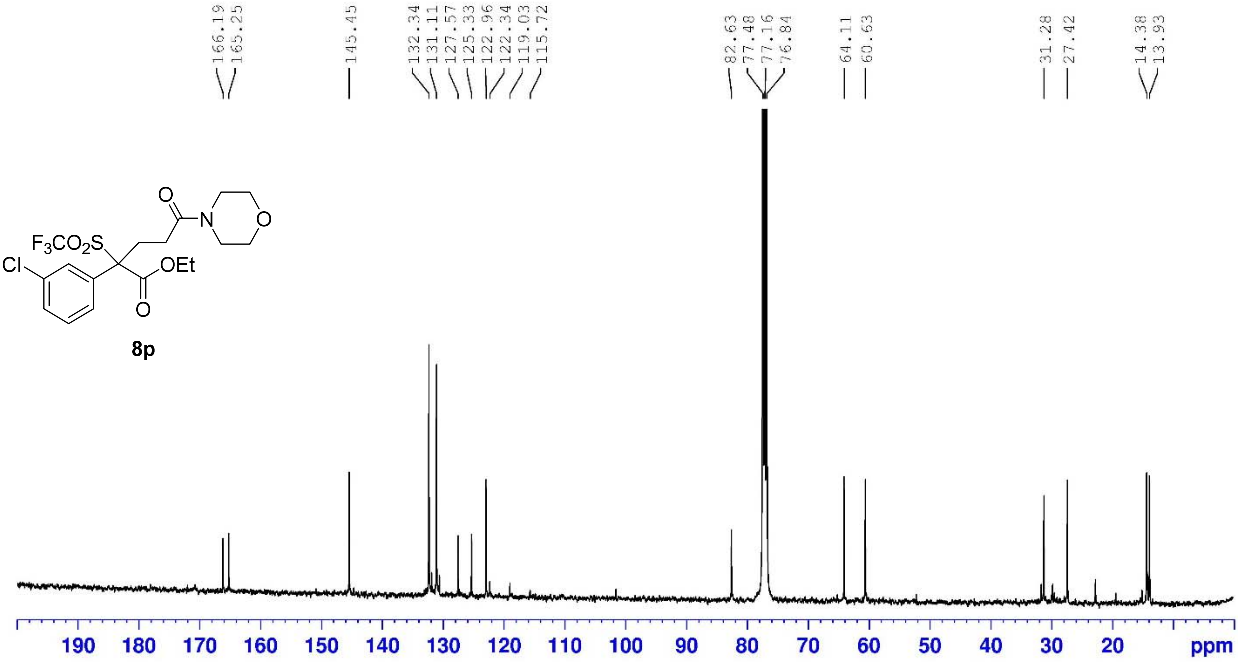

^{19}F NMR in $CDCl_3$ (376 MHz)

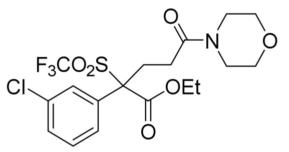

8p

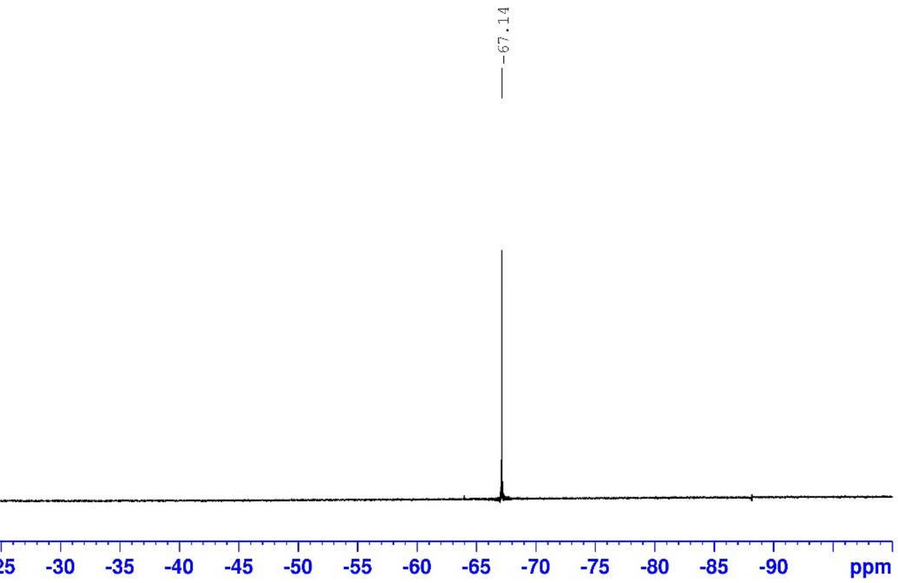

^{1}H NMR in $CDCl_3$ (300 MHz)

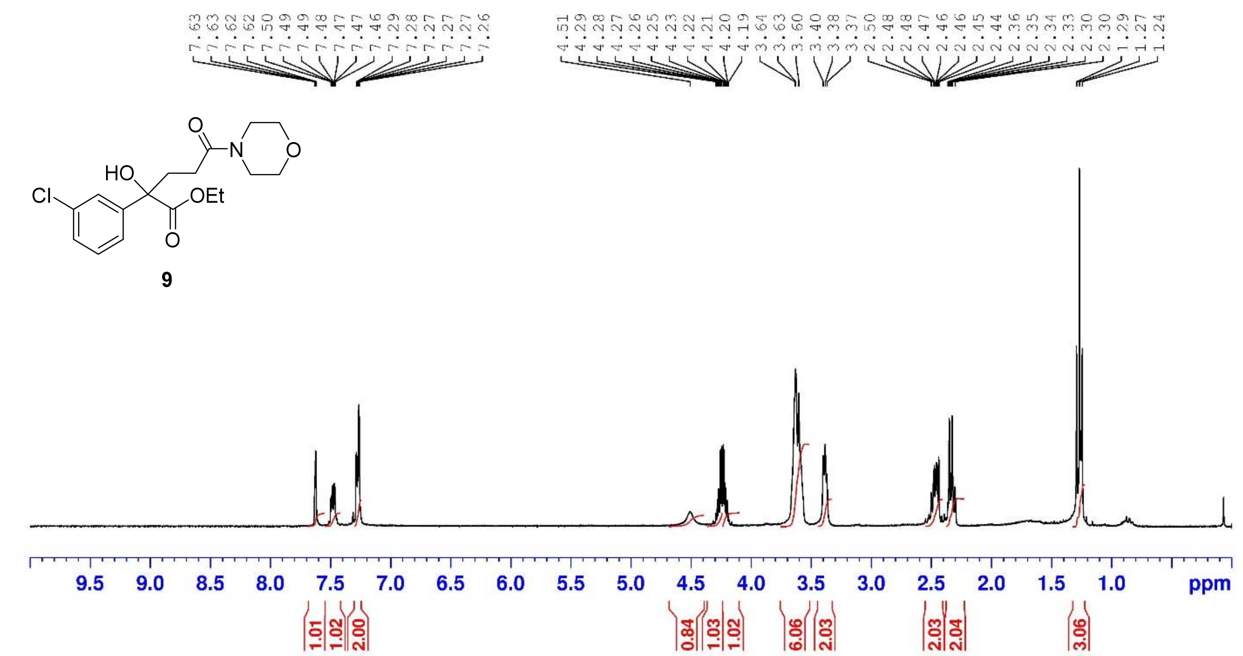

^{13}C NMR in CD_2Cl_2 (100 MHz)

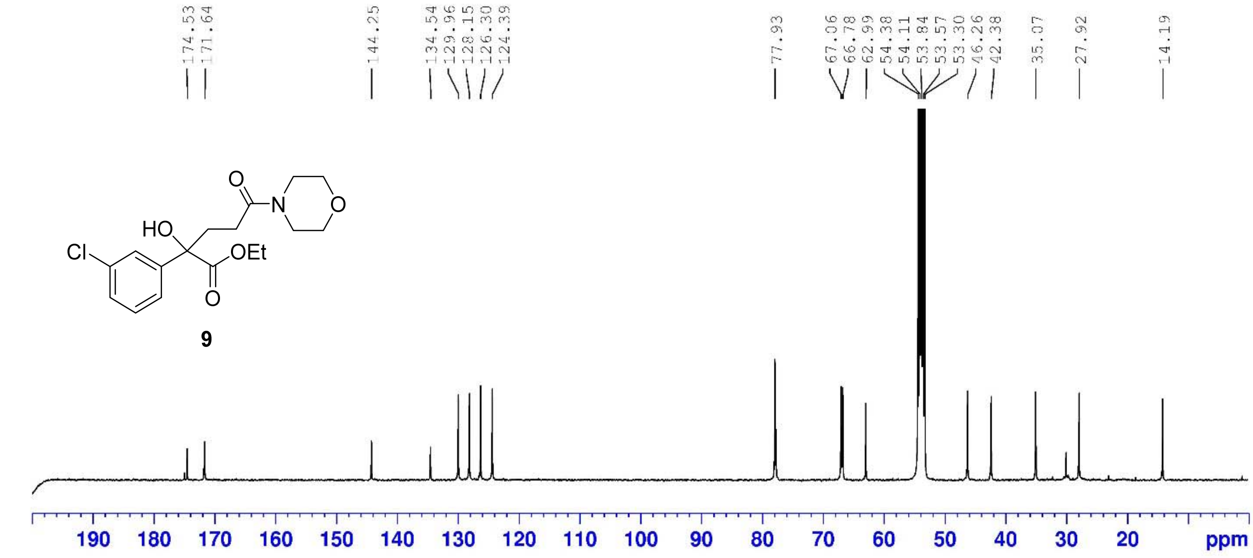

^{1}H NMR in $CDCl_3$ (400 MHz)

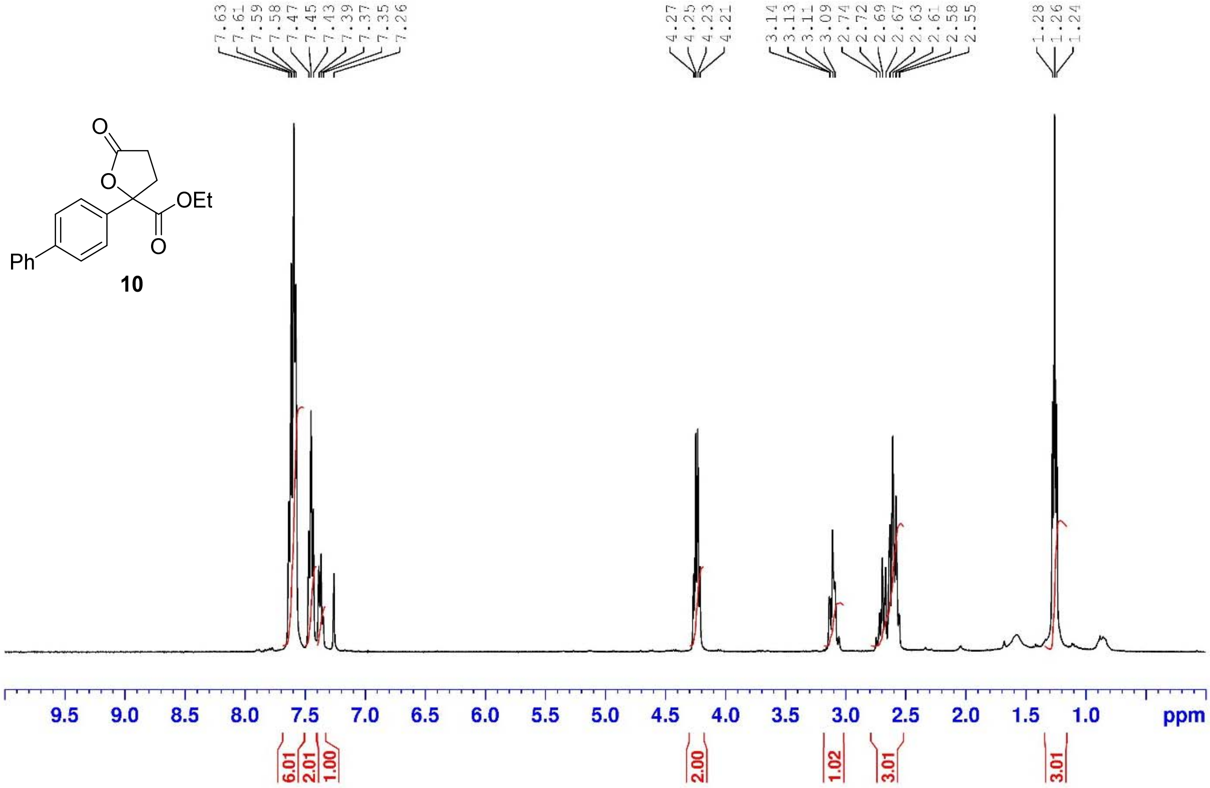

^{13}C NMR in $CDCl_3$ (100 MHz)

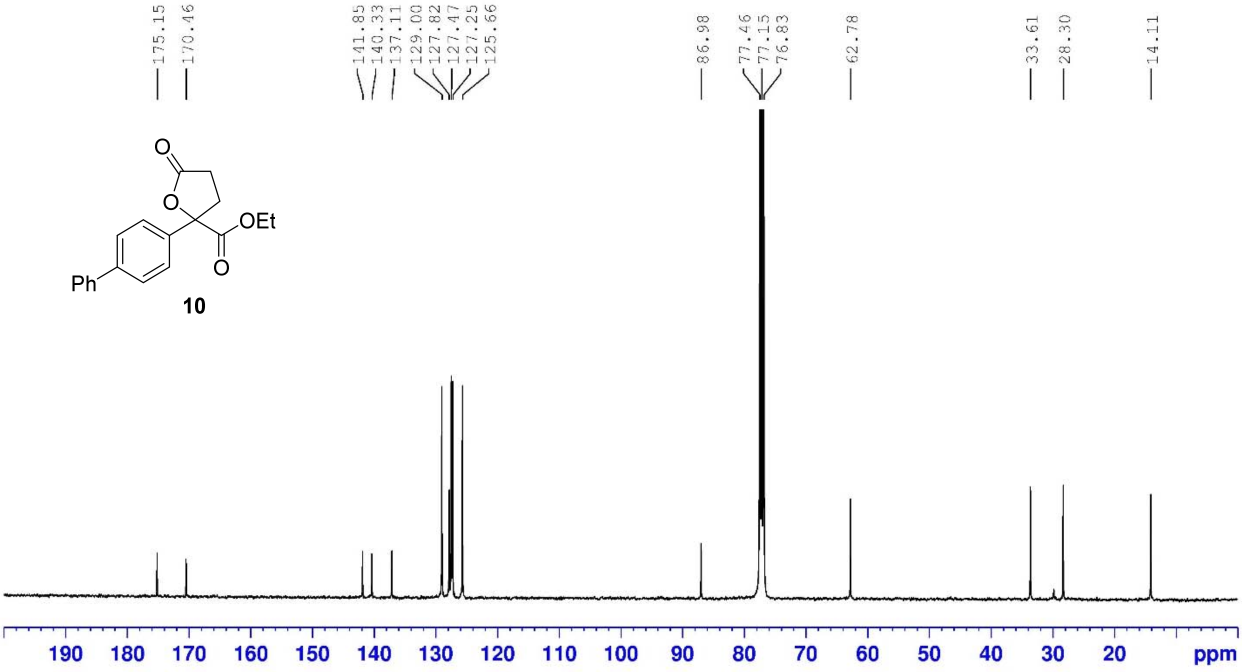

^{1}H NMR in $CDCl_3$ (300 MHz)

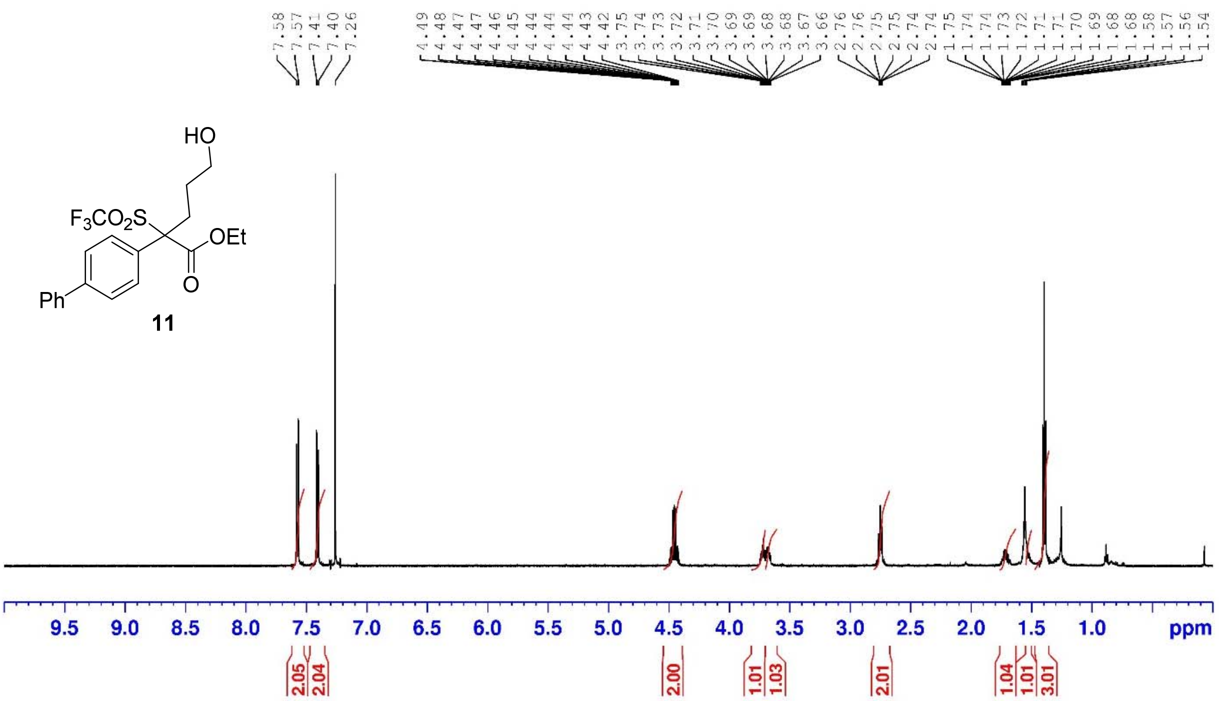

^{13}C NMR in $CDCl_3$ (150 MHz)

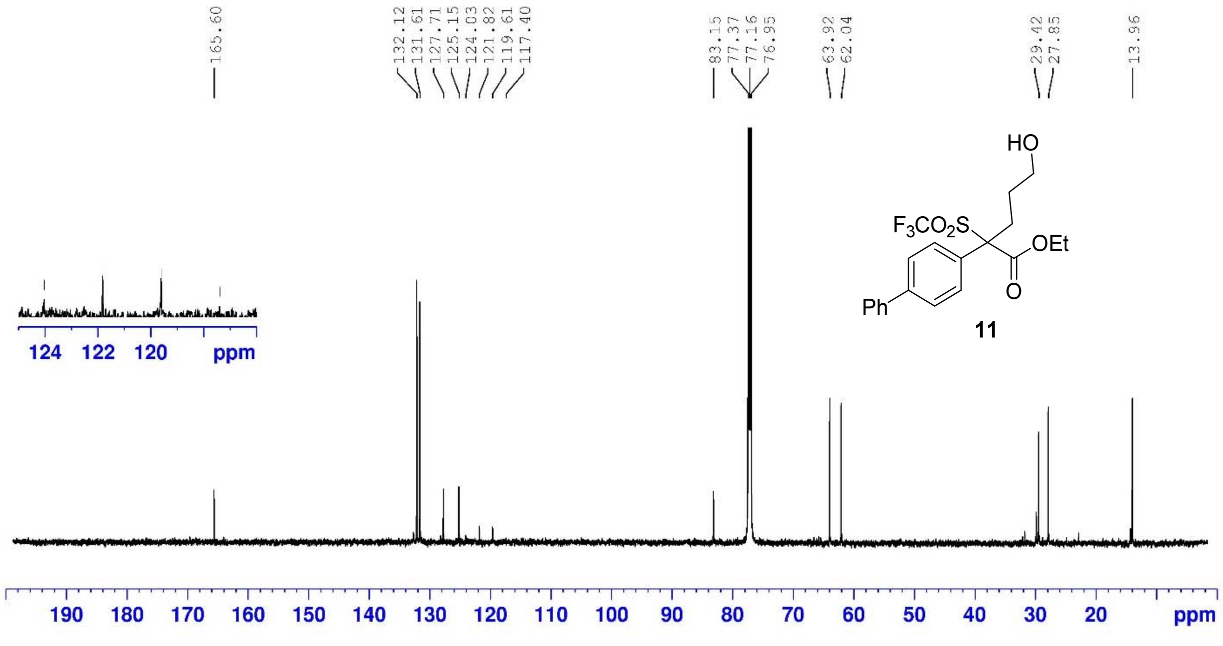

^{19}F NMR in $CDCl_3$ (376 MHz)

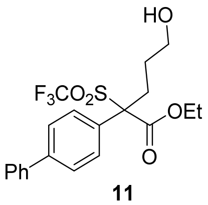

11

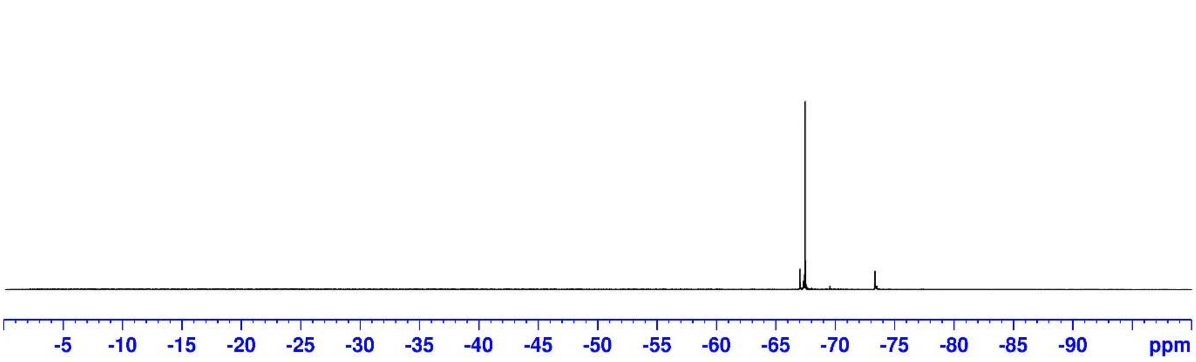

^{1}H NMR in $CDCl_3$ (400 MHz)

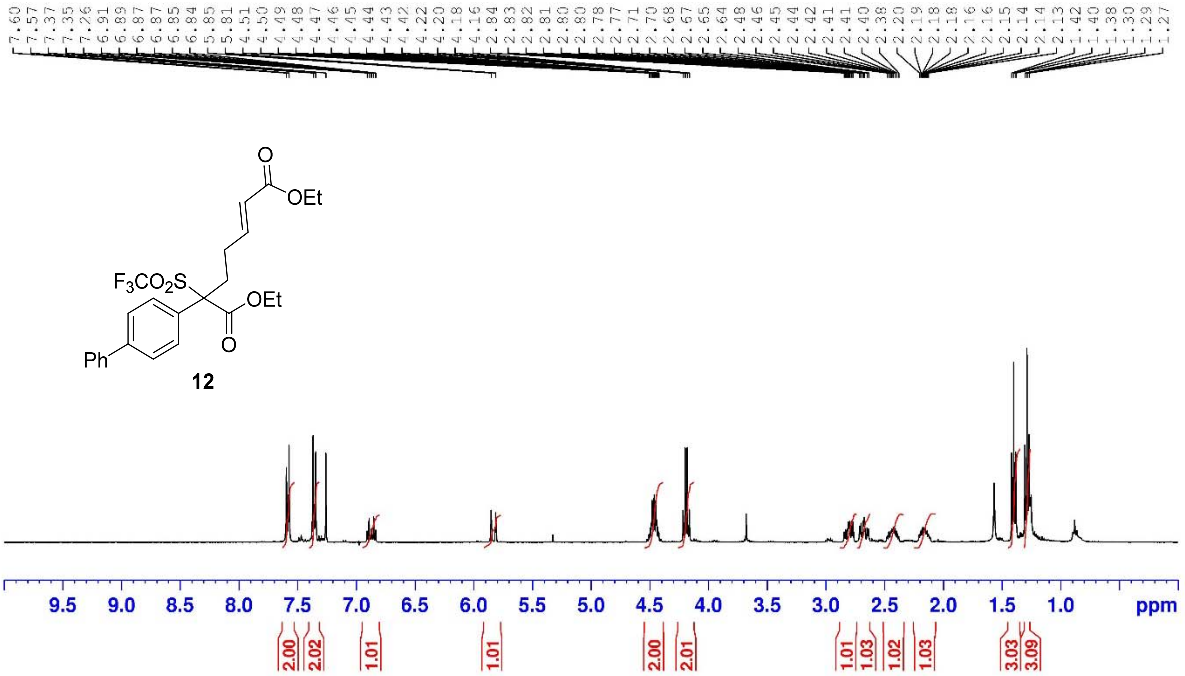

^{13}C NMR in $CDCl_3$ (100 MHz)

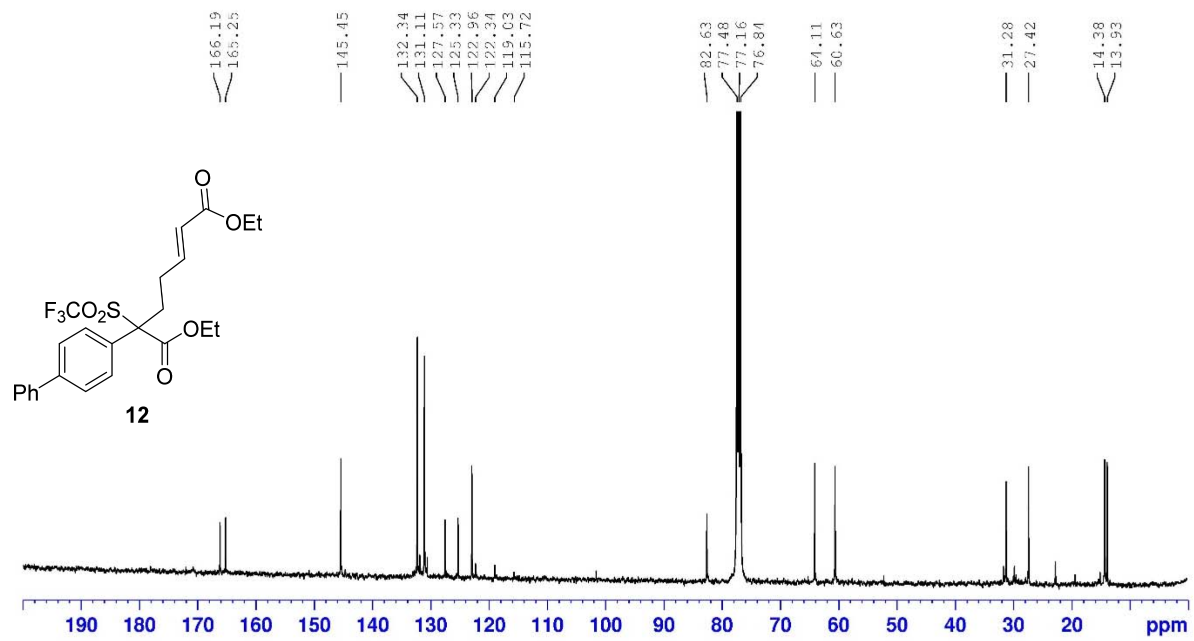

^{19}F NMR in $CDCl_3$ (376 MHz)

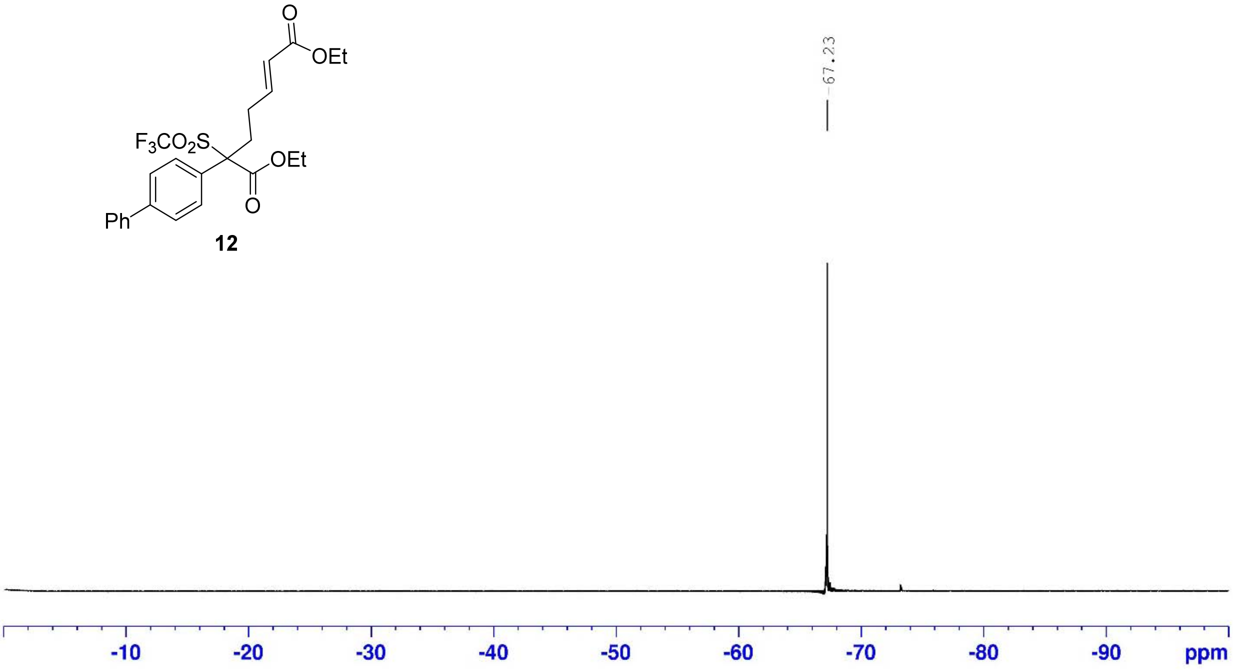

HPLC Chromatograms

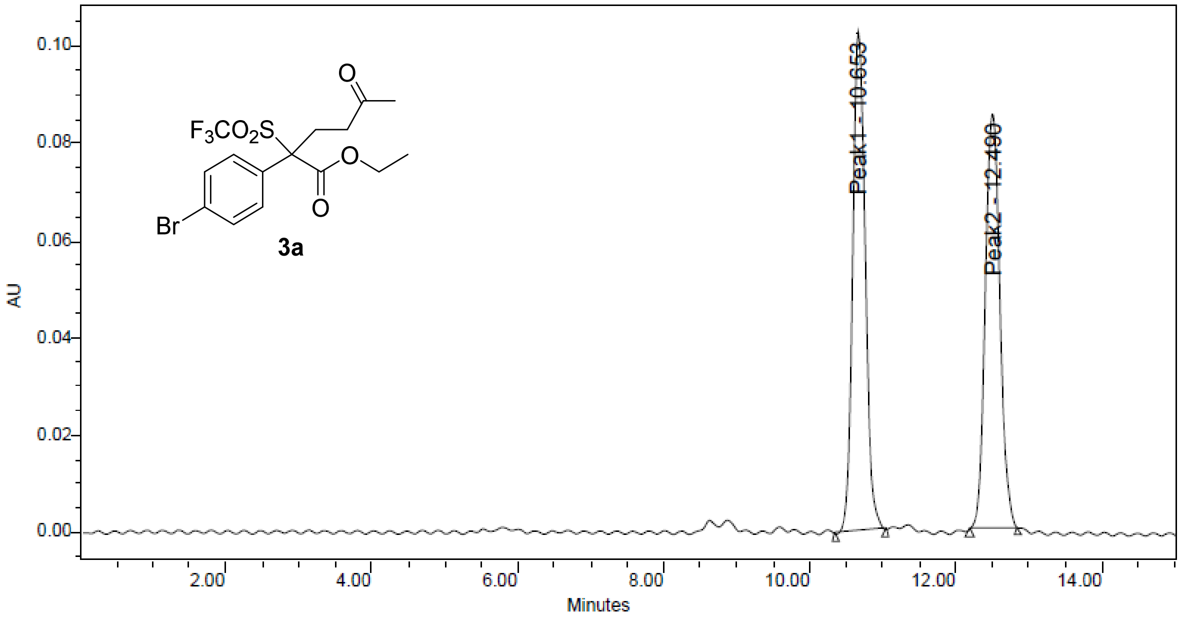

	Peak Name	RT (min)	Area (µV*sec)	% Area	Height (µV)	% Height
1	Peak1	10.653	1223209	50.82	103003	54.64
2	Peak2	12.490	1183876	49.18	85521	45.36

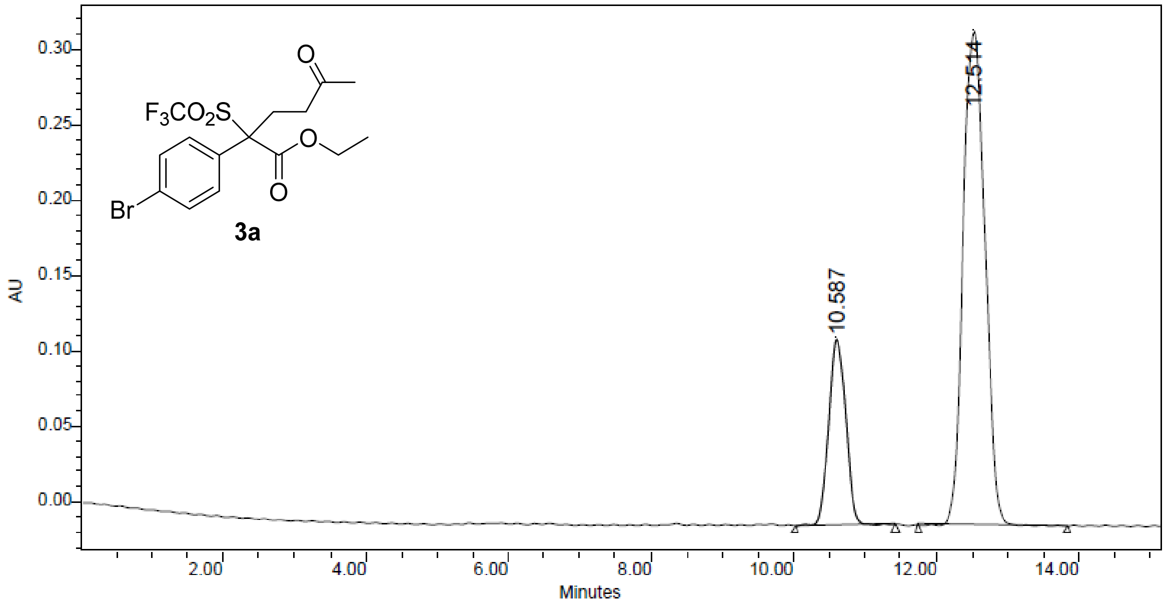

	RT (min)	Area (µV*sec)	% Area	Height (µV)	% Height
1	10.587	2063144	22.73	123386	27.34
2	12.514	7015175	77.27	327964	72.66

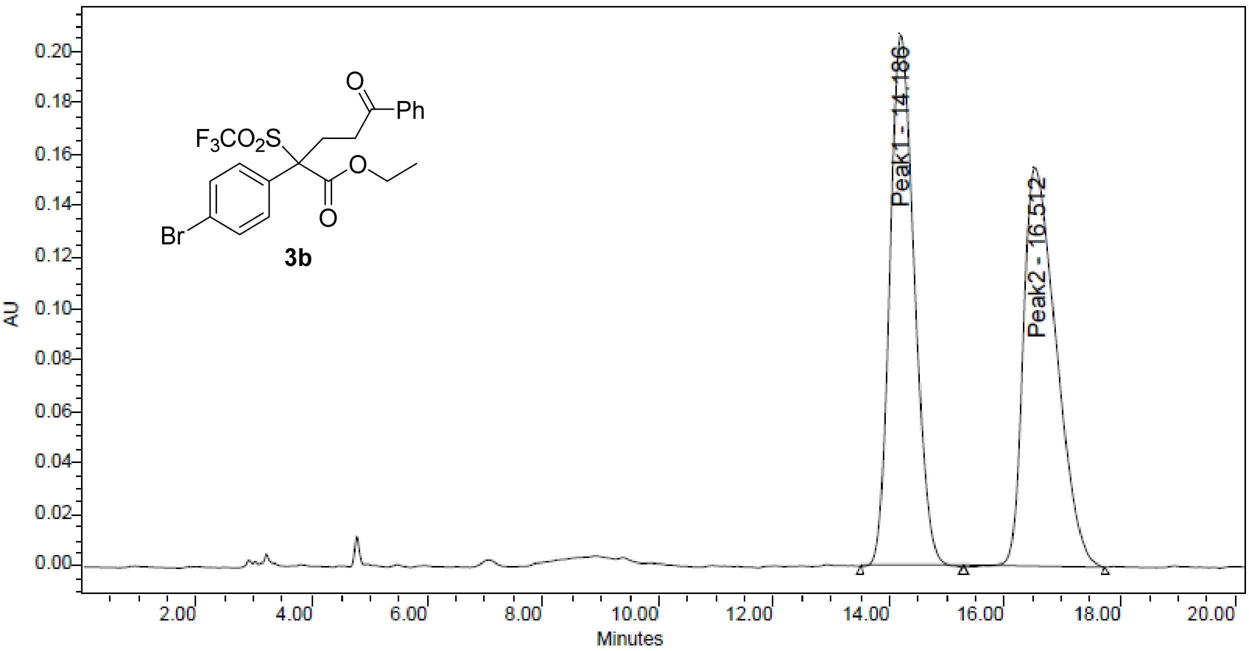

	Peak Name	RT (min)	Area (µV*sec)	% Area	Height (µV)	% Height
1	Peak1	14.186	6123076	49.77	206777	57.08
2	Peak2	16.512	6179322	50.23	155450	42.92

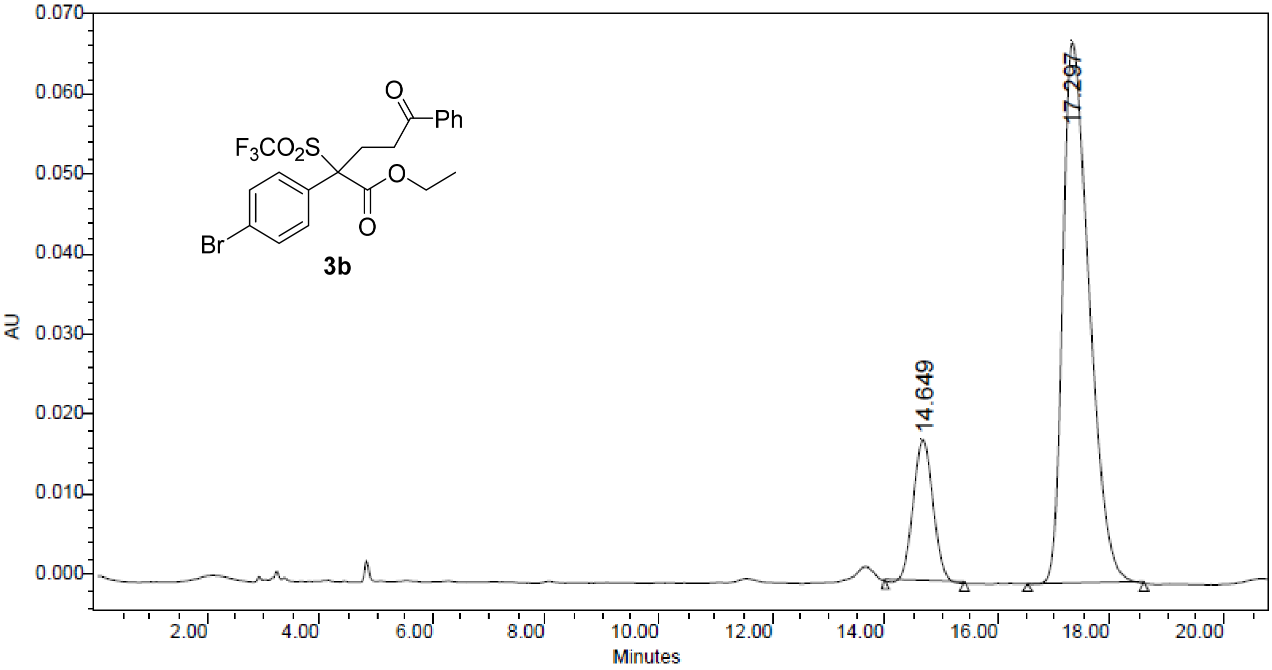

	RT (min)	Area (µV*sec)	% Area	Height (µV)	% Height
1	14.649	438937	16.64	17795	20.84
2	17.297	2199405	83.36	67612	79.16

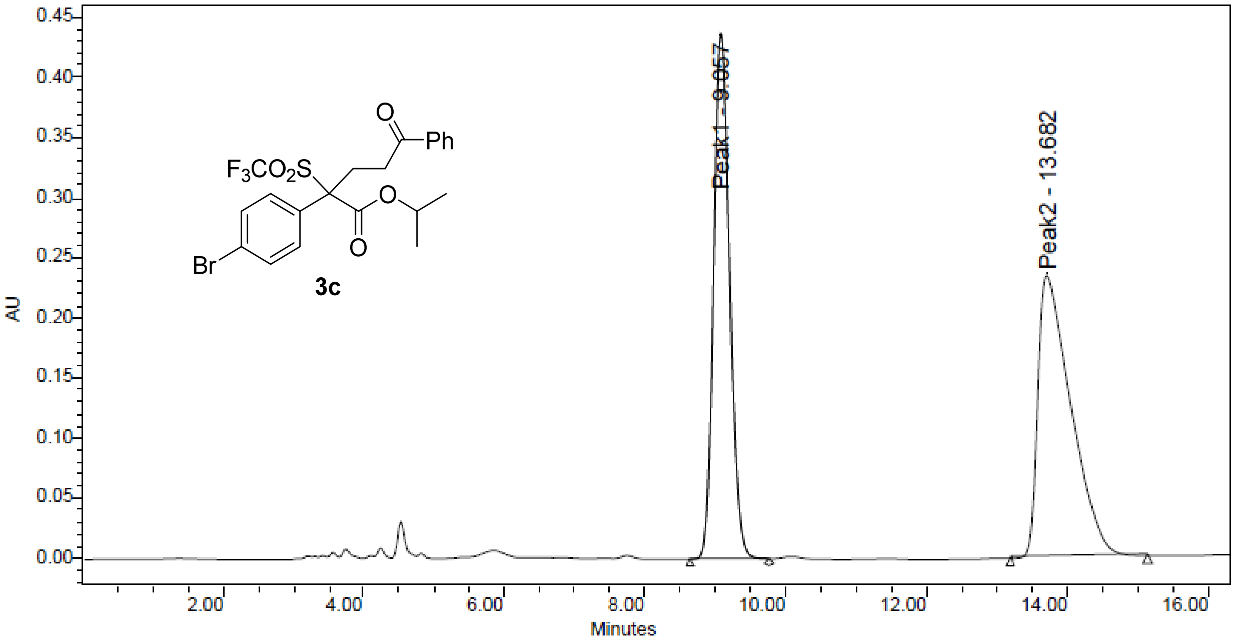

	Peak Name	RT (min)	Area (µV*sec)	% Area	Height (µV)	% Height
1	Peak1	9.057	6956091	47.55	438207	65.11
2	Peak2	13.682	7674422	52.45	234860	34.89

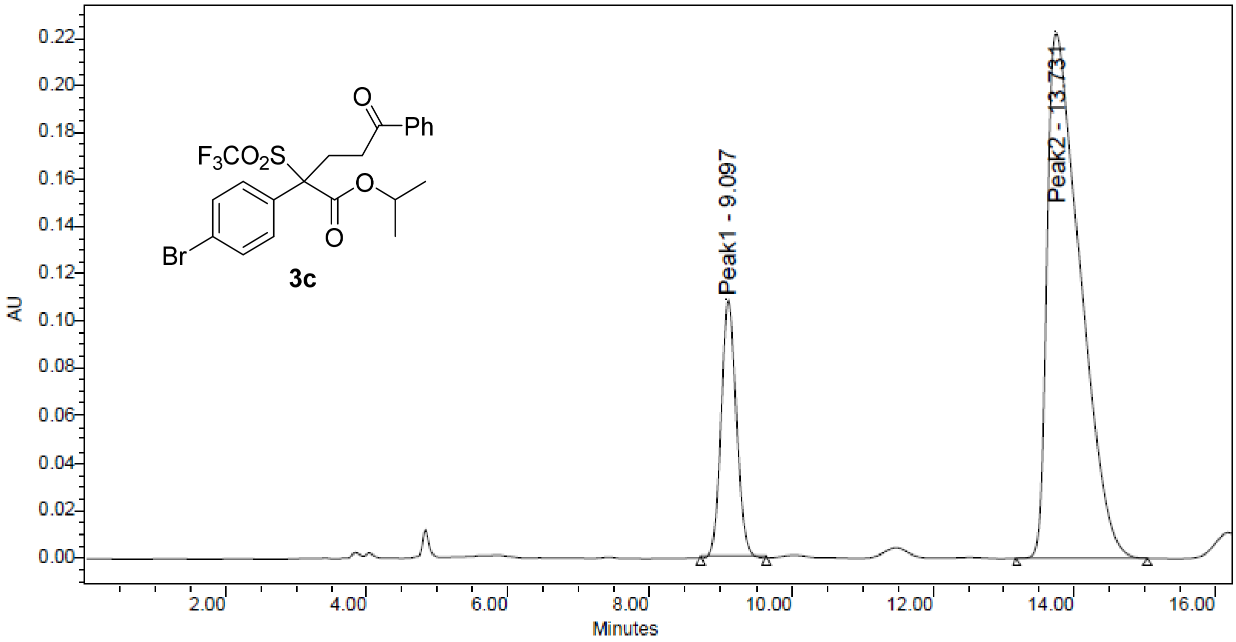

	Peak Name	RT (min)	Area (µV*sec)	% Area	Height (µV)	% Height
1	Peak1	9.097	1658454	18.69	109104	32.93
2	Peak2	13.731	7215734	81.31	222260	67.07

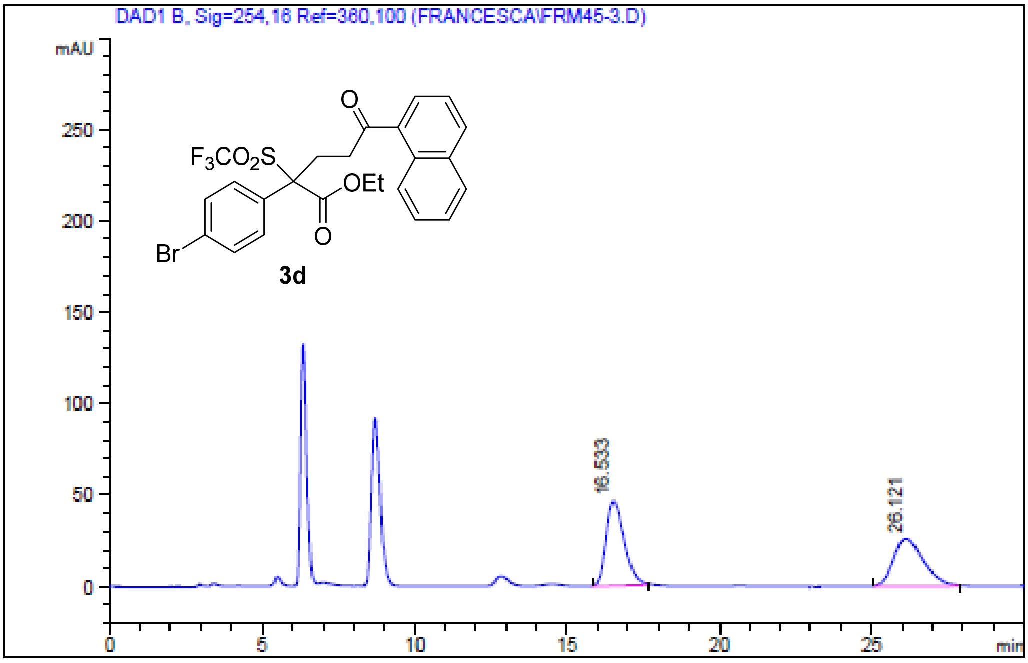

Signal 1: DAD1 B, Sig=254,16 Ref=360,100

Peak #	RT [min]	Type	Width [min]	Area	Area %	Name
1	16.533	BB	0.636	1923.080	51.920	
2	26.121	BB	1.021	1780.875	48.080	

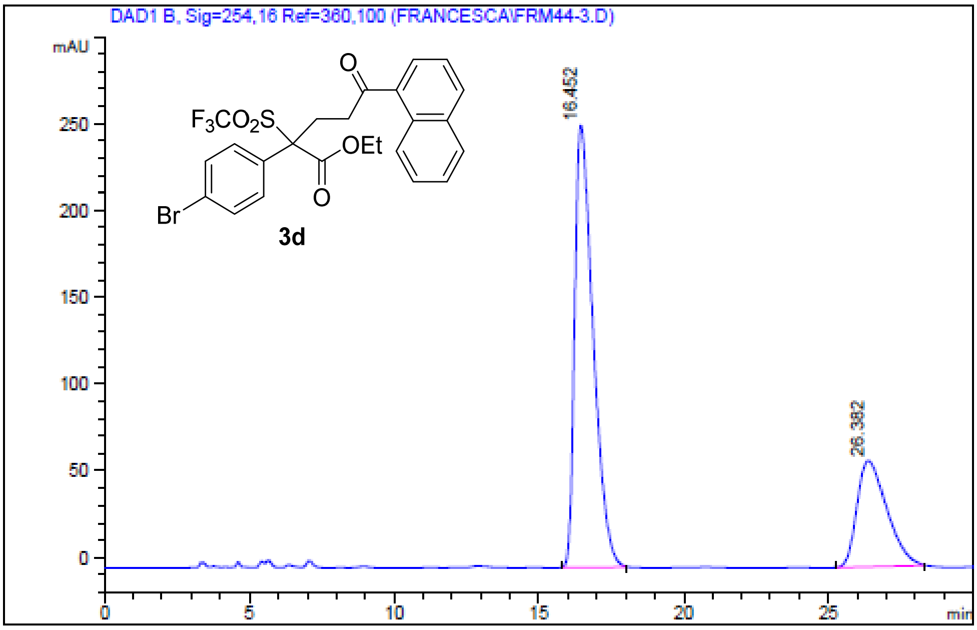

Signal 1: DAD1 B, Sig=254,16 Ref=360,100

Peak #	RT [min]	Type	Width [min]	Area	Area %	Name
1	16.452	BB	0.680	11347.926	72.057	
2	26.382	BB	1.083	4400.603	27.943	

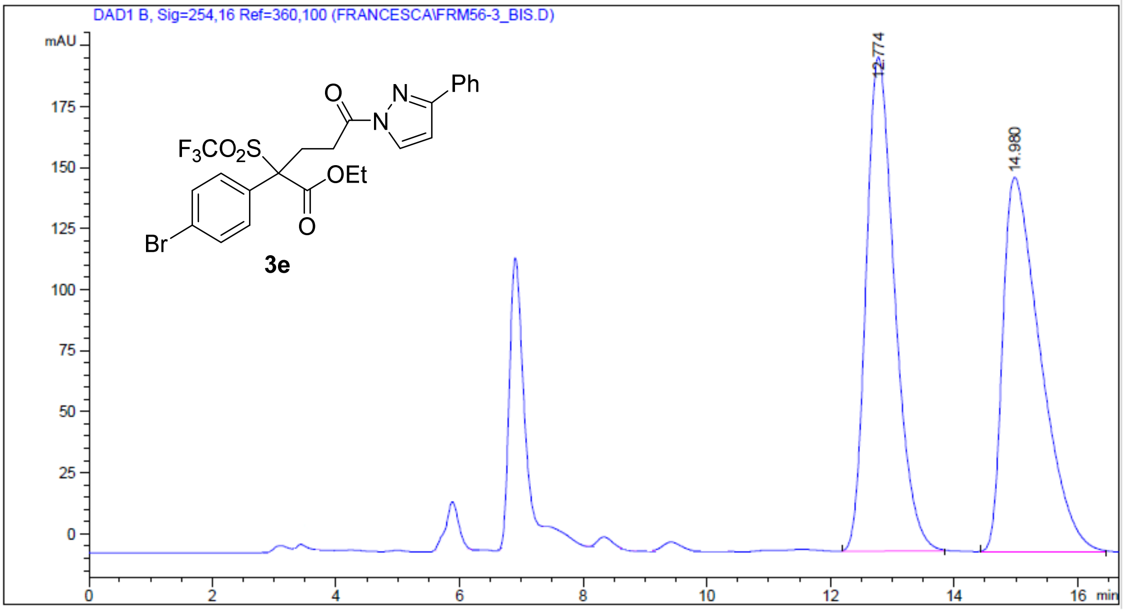

===

Area Percent Report

===

Peak #	RetTime [min]	Type	Width [min]	Area [mAU*s]	Height [mAU]	Area %
1	12.774	BB	0.4918	6481.93164	202.29431	42.4597
2	14.980	BB	0.6387	6466.71338	153.13930	42.3600

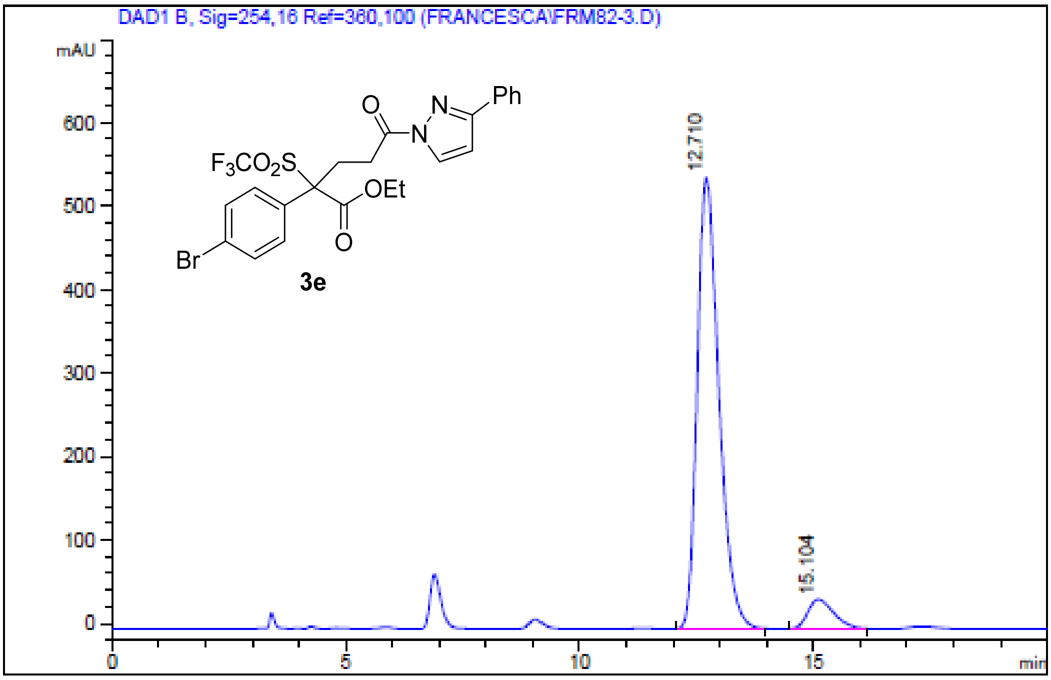

Signal 1: DAD1 B, Sig=254,16 Ref=360,100

Peak #	RT [min]	Type	Width [min]	Area	Area %	Name
1	12.710	BB	0.494	17410.287	92.787	
2	15.104	BB	0.592	1353.520	7.213	

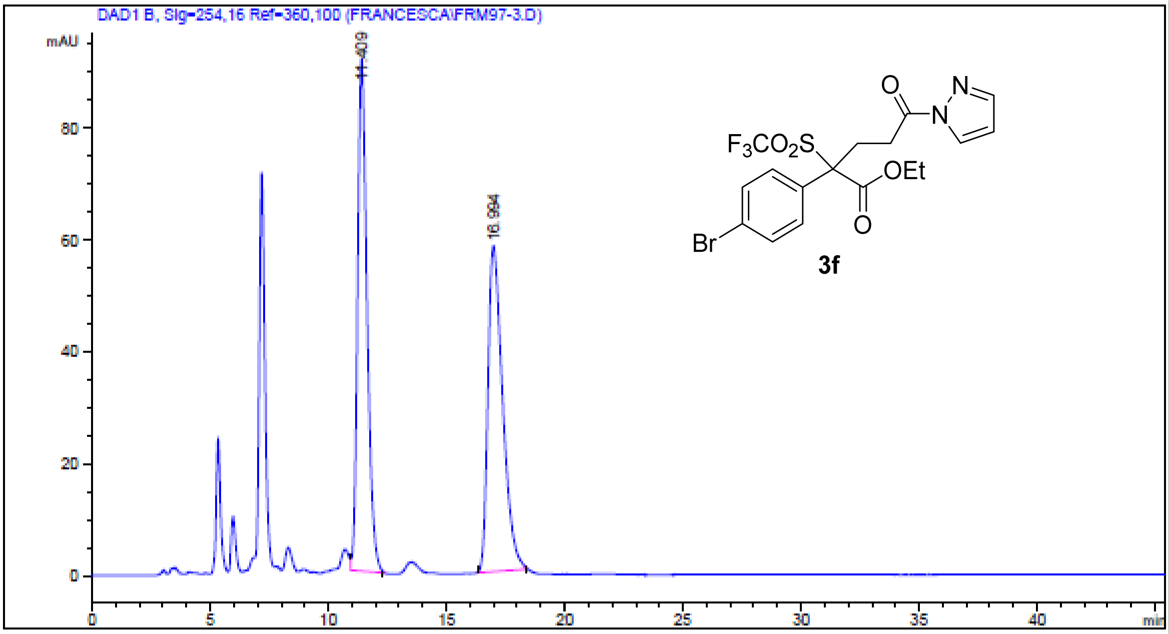

Area Percent Report

Peak #	RetTime [min]	Type	Width [min]	Area [mAU*s]	Height [mAU]	Area %
1	11.409	VB	0.4280	2575.32959	91.59644	50.4815
2	16.994	BB	0.6571	2526.20508	58.12267	49.5185

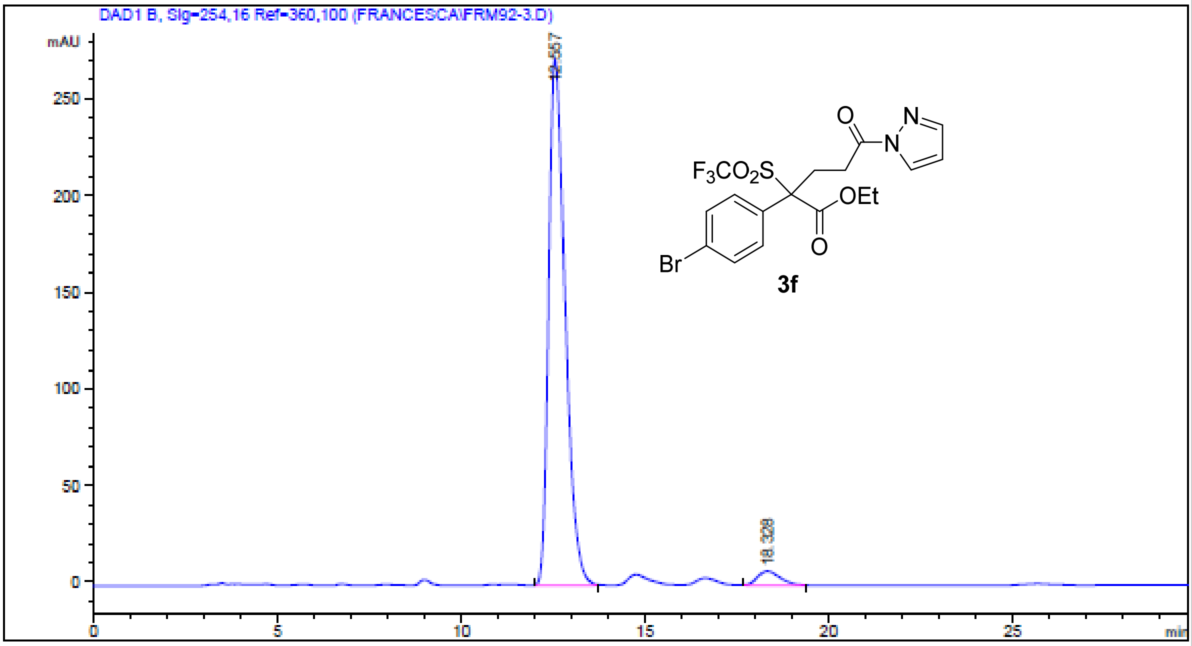

Area Percent Report

Peak #	RetTime [min]	Type	Width [min]	Area [mAU*s]	Height [mAU]	Area %
1	12.557	BB	0.4785	8521.11035	272.70419	96.3199
2	18.328	BB	0.6332	325.56491	7.48916	3.6801

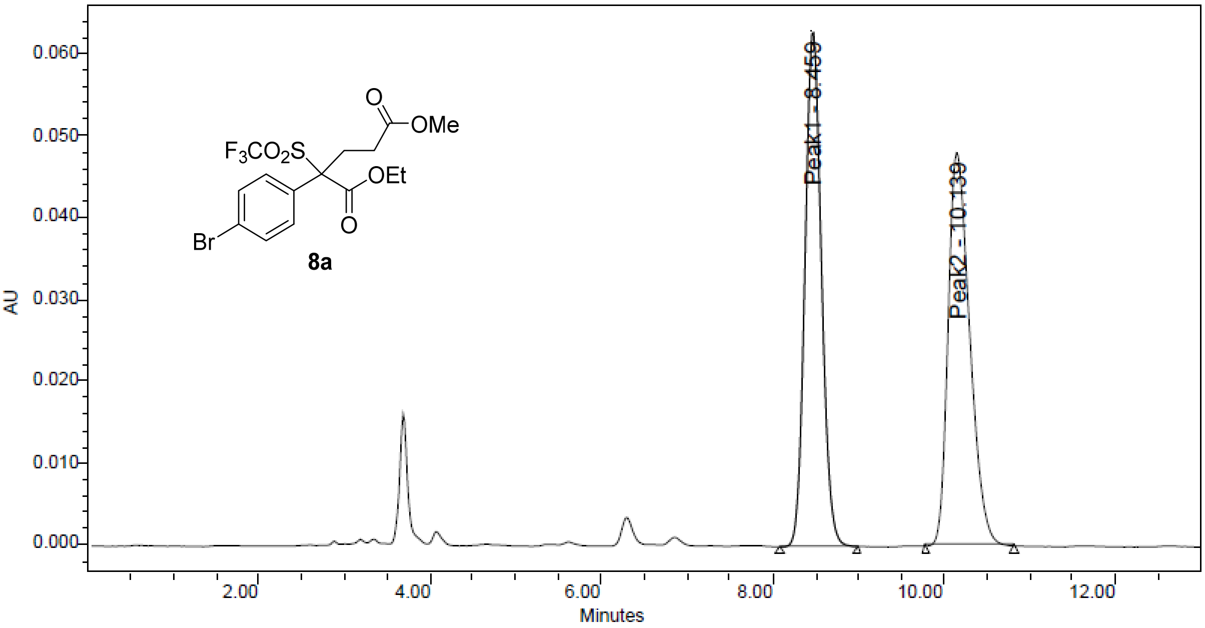

	Peak Name	RT (min)	Area ([illegible]V*sec)	% Area	Height ([illegible]V)	% Height
1	Peak1	8.459	844229	49.95	62986	56.72
2	Peak2	10.139	846061	50.05	48070	43.28

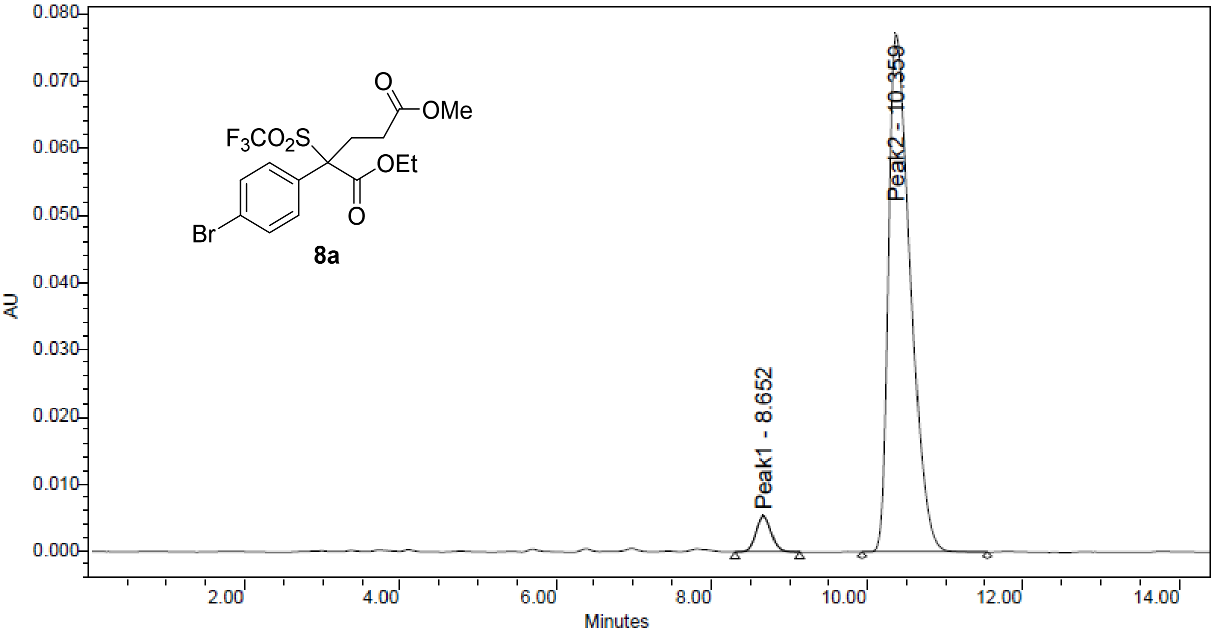

	Peak Name	RT (min)	Area ([illegible]V*sec)	% Area	Height ([illegible]V)	% Height
1	Peak1	8.652	74431	4.67	5392	6.51
2	Peak2	10.359	1518364	95.33	77424	93.49

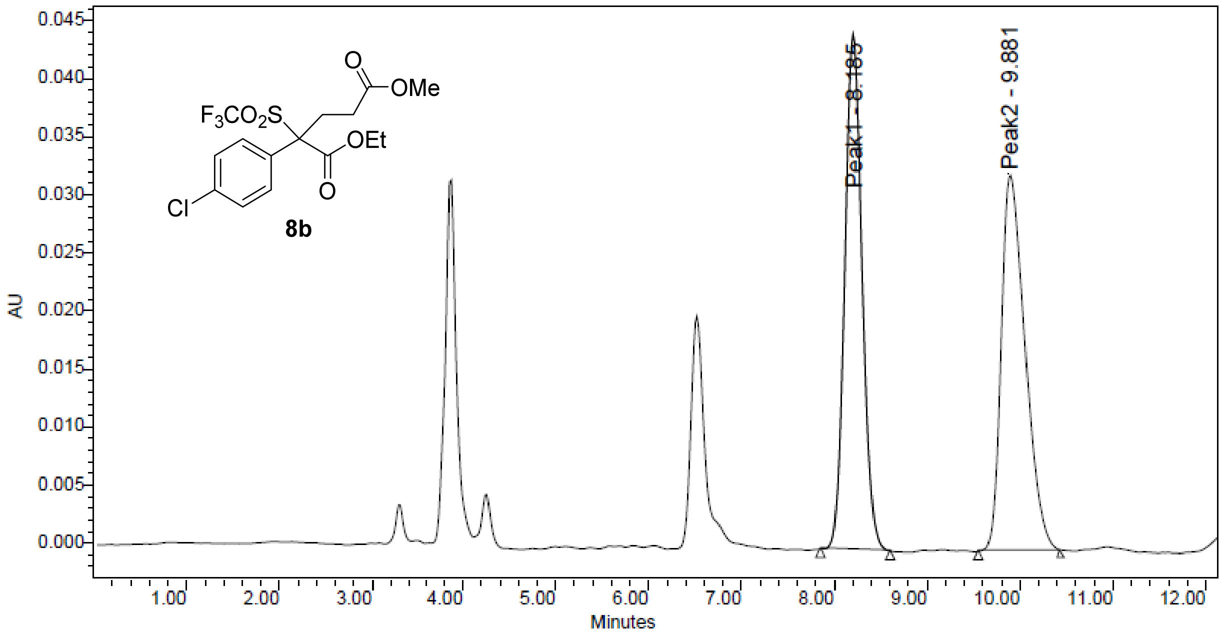

	Peak Name	RT (min)	Area (µV*sec)	% Area	Height (µV)	% Height
1	Peak1	8.185	581391	50.06	44461	57.86
2	Peak2	9.881	580098	49.94	32378	42.14

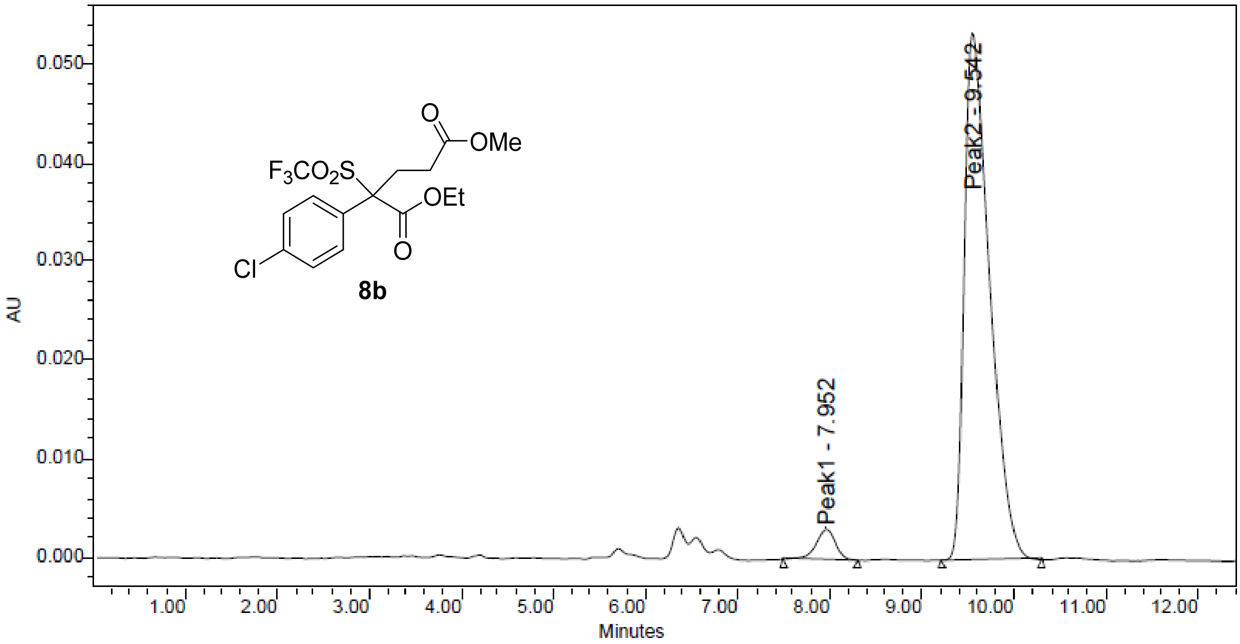

	Peak Name	RT (min)	Area (µV*sec)	% Area	Height (µV)	% Height
1	Peak1	7.952	46765	4.34	3110	5.48
2	Peak2	9.542	1031203	95.66	53688	94.52

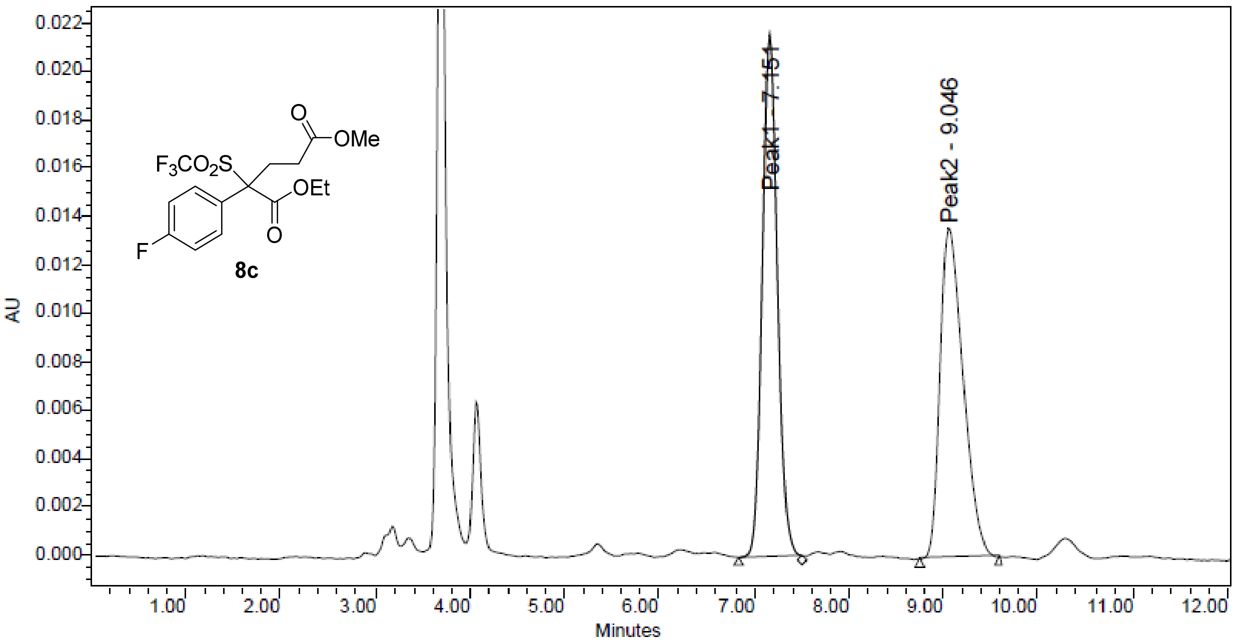

	Peak Name	RT (min)	Area (µV*sec)	% Area	Height (µV)	% Height
1	Peak1	7.151	239231	50.71	21567	61.26
2	Peak2	9.046	232557	49.29	13640	38.74

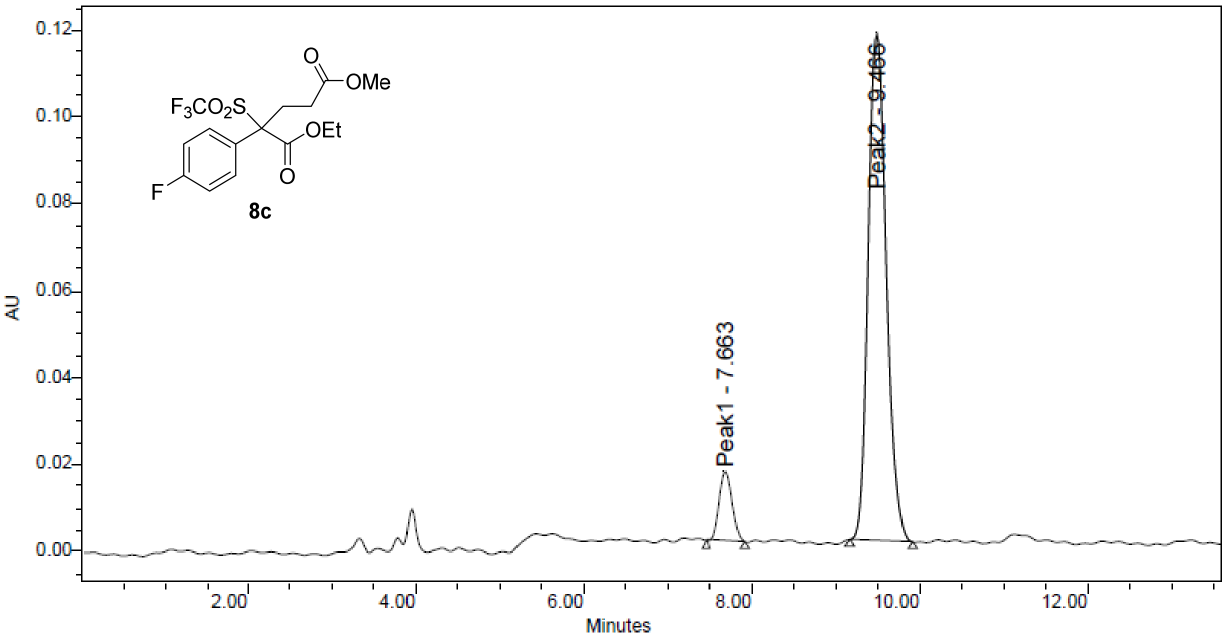

	Peak Name	RT (min)	Area (µV*sec)	% Area	Height (µV)	% Height
1	Peak1	7.663	175535	9.20	16047	12.11
2	Peak2	9.466	1732819	90.80	116435	87.89

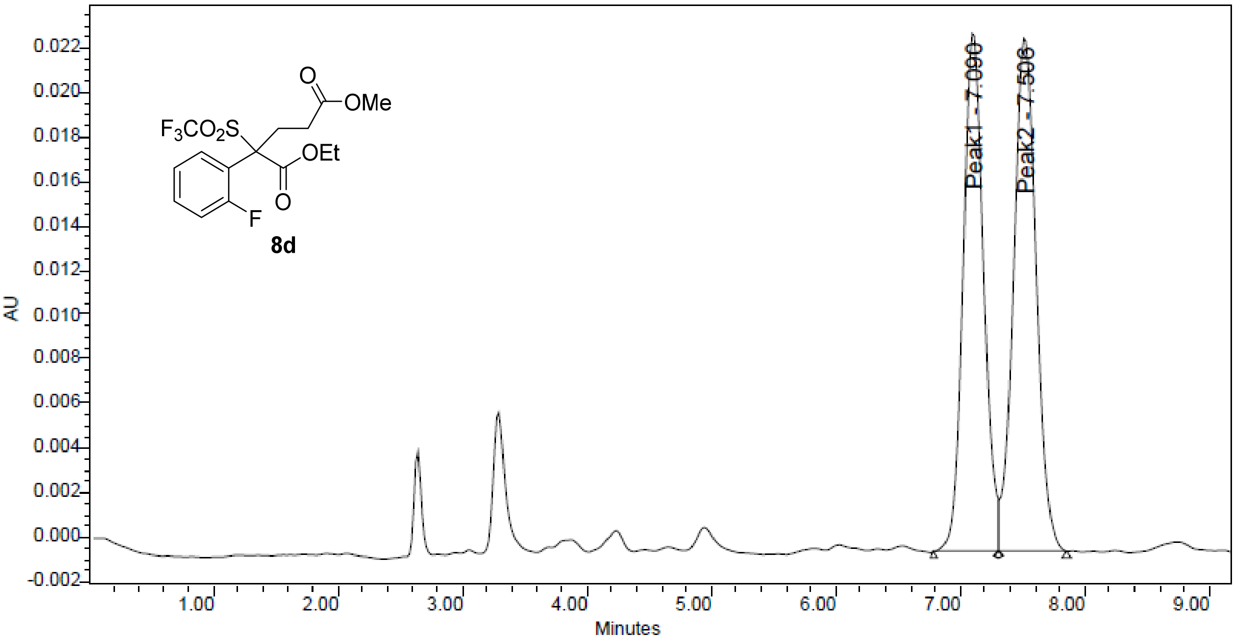

	Peak Name	RT (min)	Area (µV*sec)	% Area	Height (µV)	% Height
1	Peak1	7.090	267676	47.81	23403	50.28
2	Peak2	7.506	292188	52.19	23144	49.72

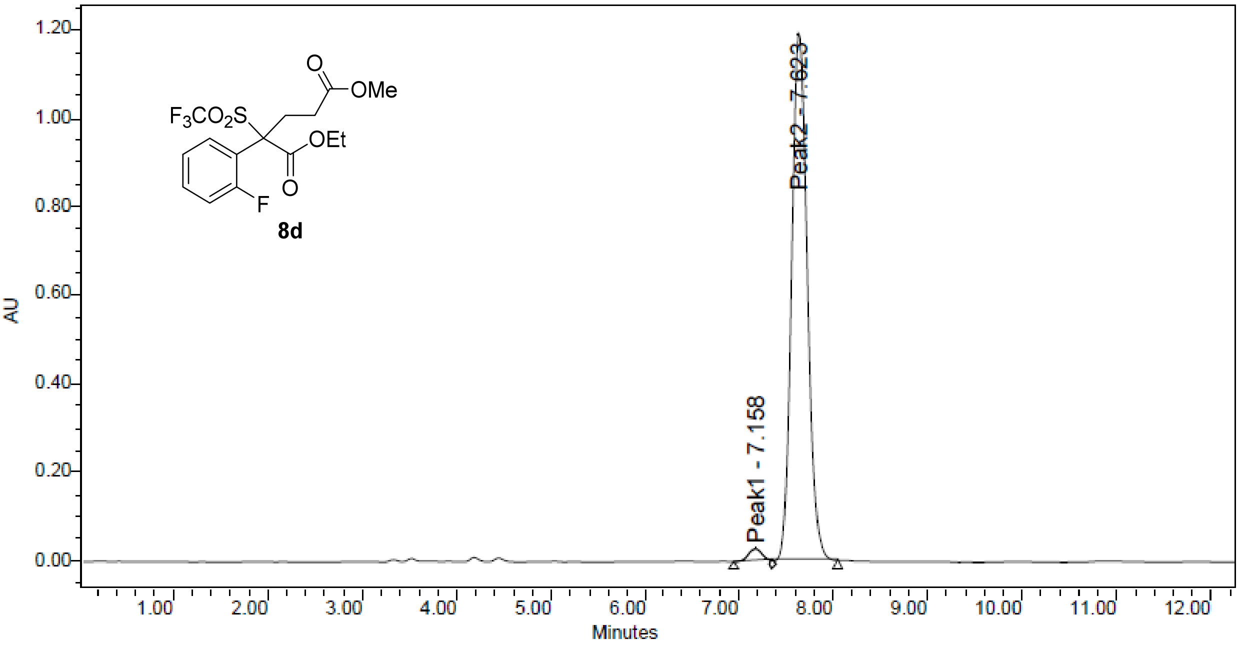

	Peak Name	RT (min)	Area (µV*sec)	% Area	Height (µV)	% Height
1	Peak1	7.158	291775	2.07	28776	2.35
2	Peak2	7.623	13773317	97.93	1196117	97.65

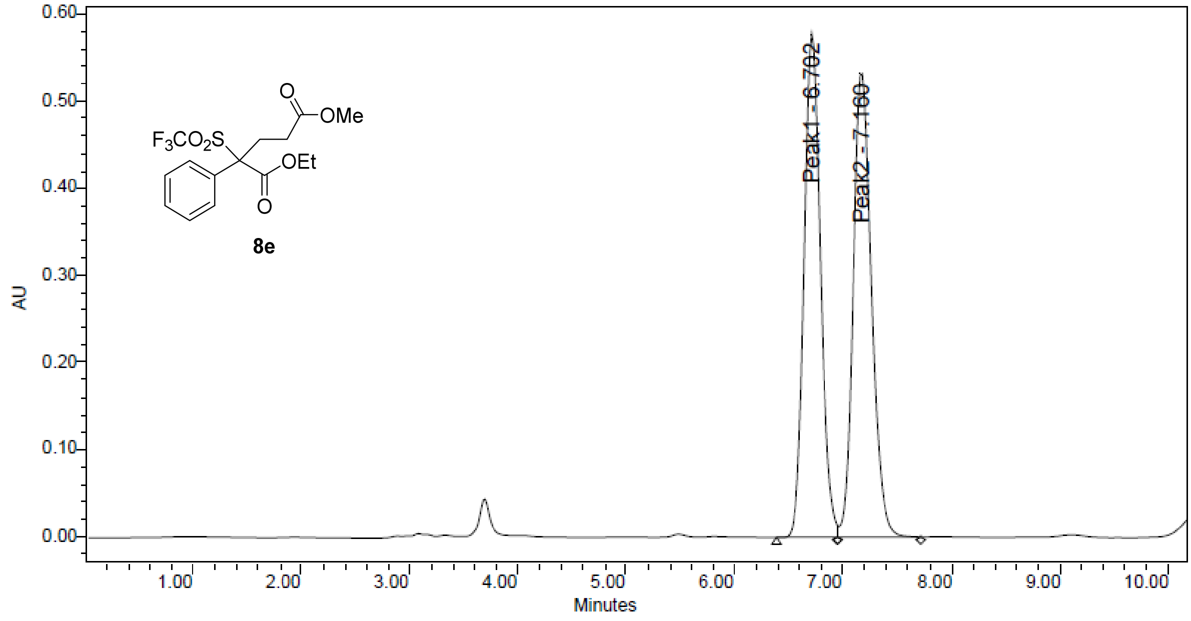

	Peak Name	RT (min)	Area (□V*sec)	% Area	Height (□V)	% Height
1	Peak1	6.702	6103004	49.35	578286	51.97
2	Peak2	7.160	6263378	50.65	534366	48.03

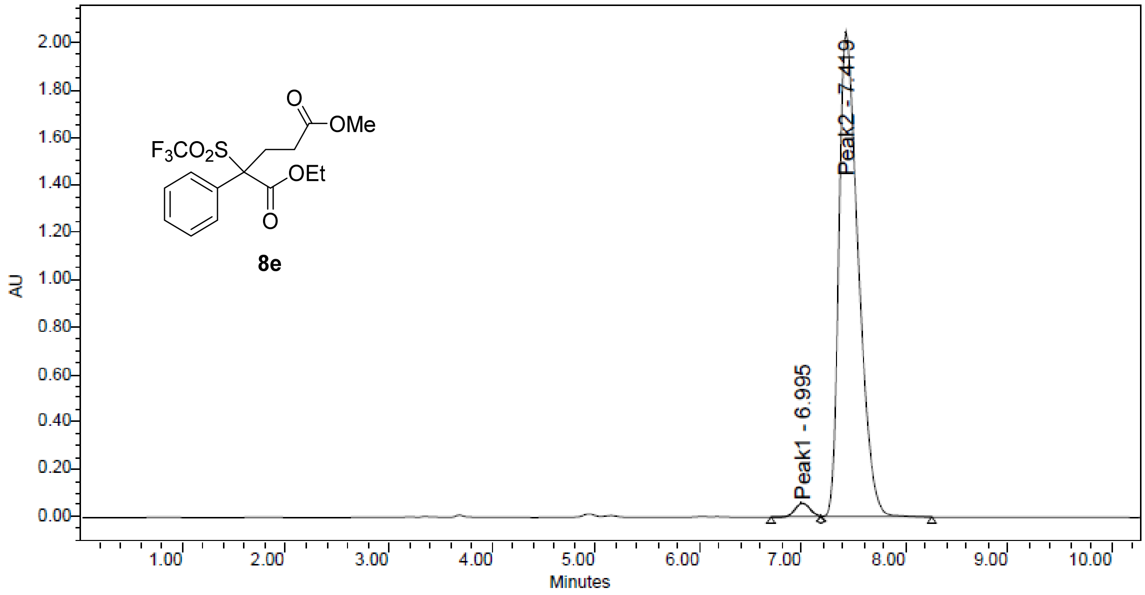

	Peak Name	RT (min)	Area (□V*sec)	% Area	Height (□V)	% Height
1	Peak1	6.995	623793	2.27	59499	2.82
2	Peak2	7.419	26824412	97.73	2051972	97.18

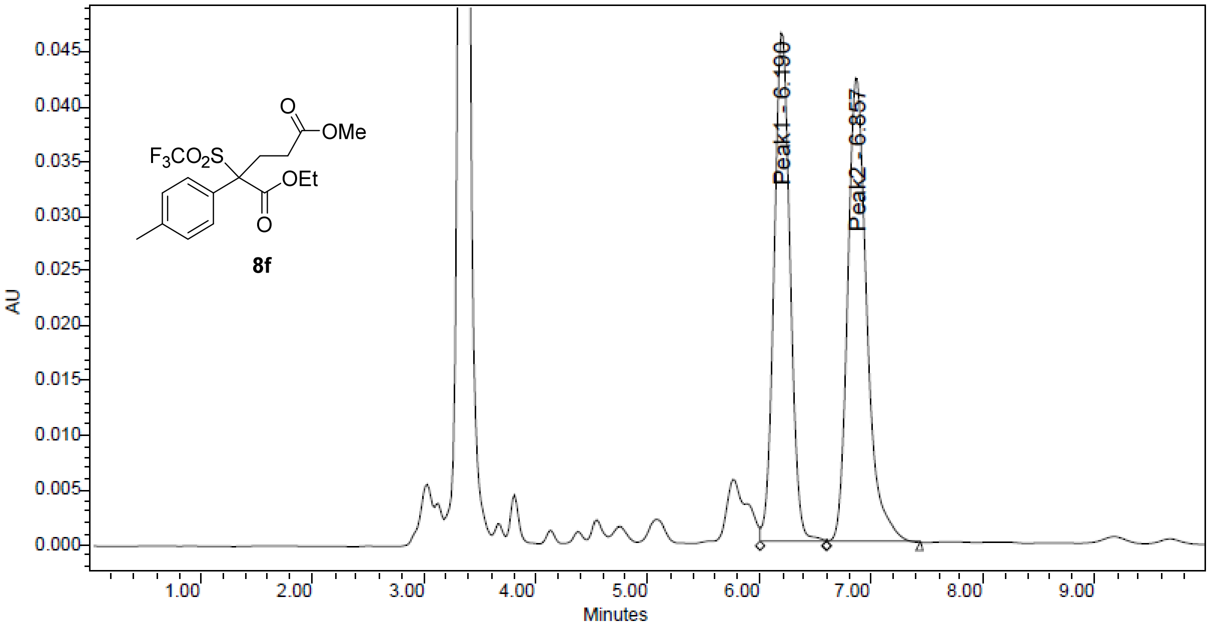

	Peak Name	RT (min)	Area (µV*sec)	% Area	Height (µV)	% Height
1	Peak1	6.190	470433	48.03	46729	52.38
2	Peak2	6.857	508984	51.97	42478	47.62

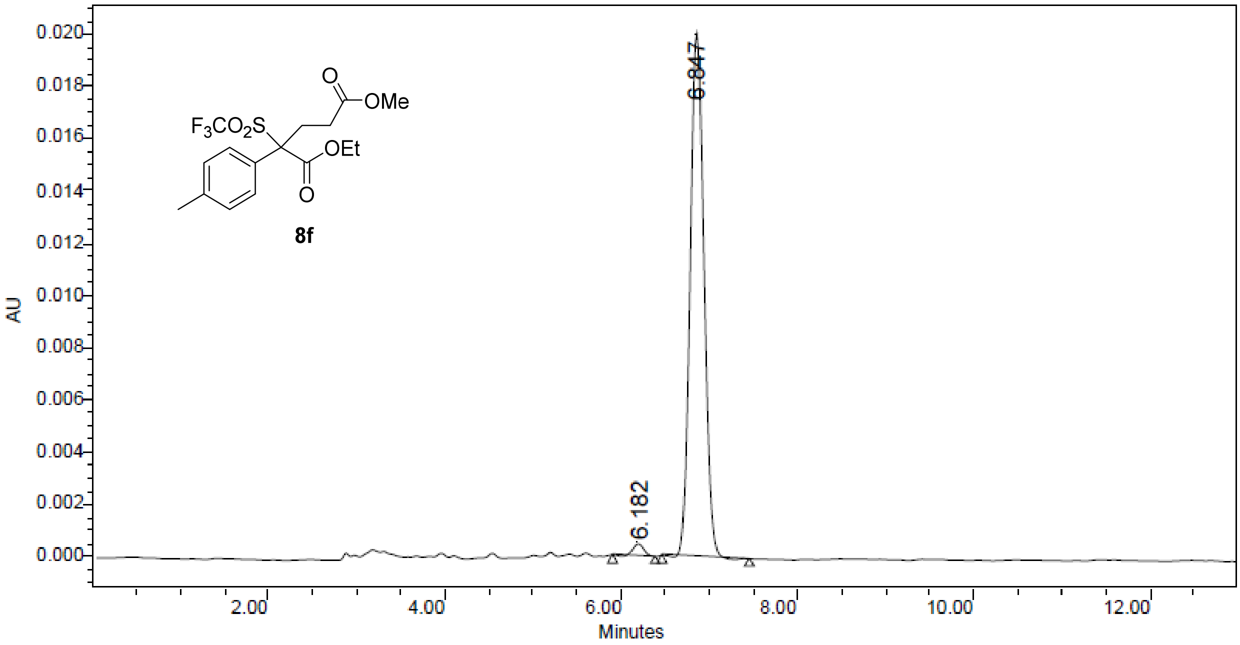

	RT (min)	Area (µV*sec)	% Area	Height (µV)	% Height
1	6.182	4059	1.82	450	2.20
2	6.847	218563	98.18	20043	97.80

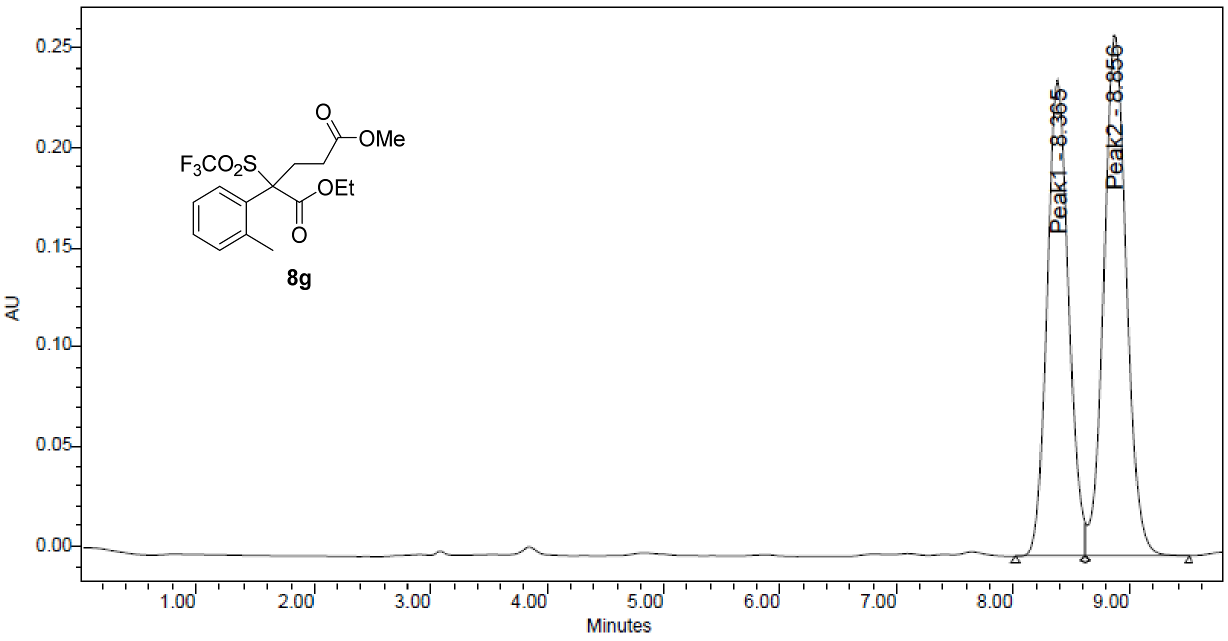

	Peak Name	RT (min)	Area (µV*sec)	% Area	Height (µV)	% Height
1	Peak1	8.365	3087387	46.67	238639	47.71
2	Peak2	8.856	3527878	53.33	261550	52.29

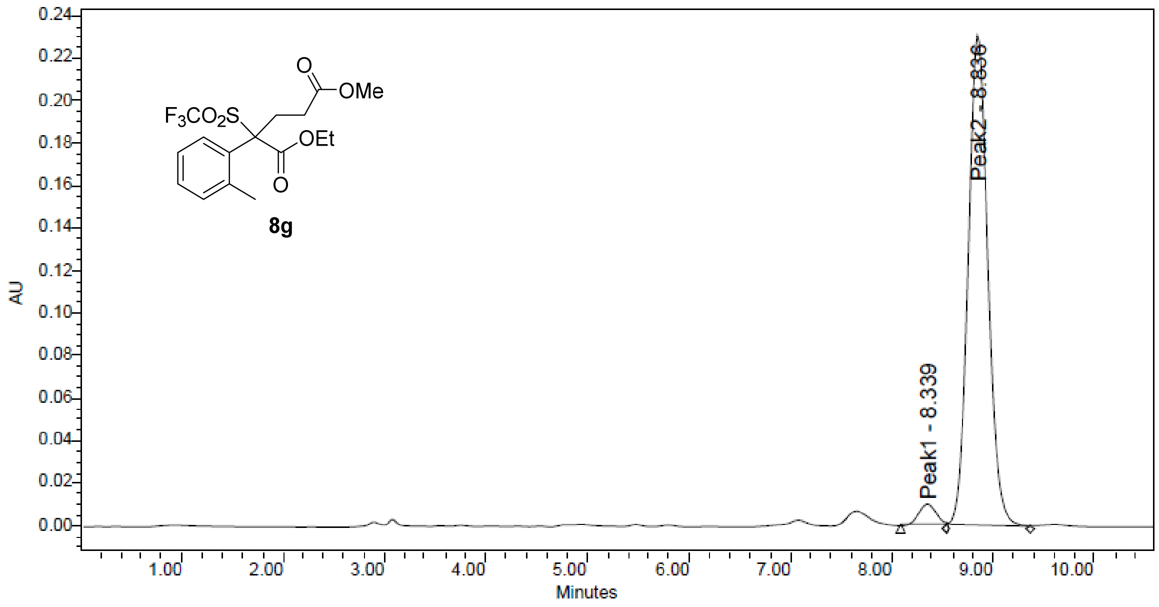

	Peak Name	RT (min)	Area (µV*sec)	% Area	Height (µV)	% Height
1	Peak1	8.339	127053	3.91	10211	4.23
2	Peak2	8.836	3123303	96.09	230911	95.77

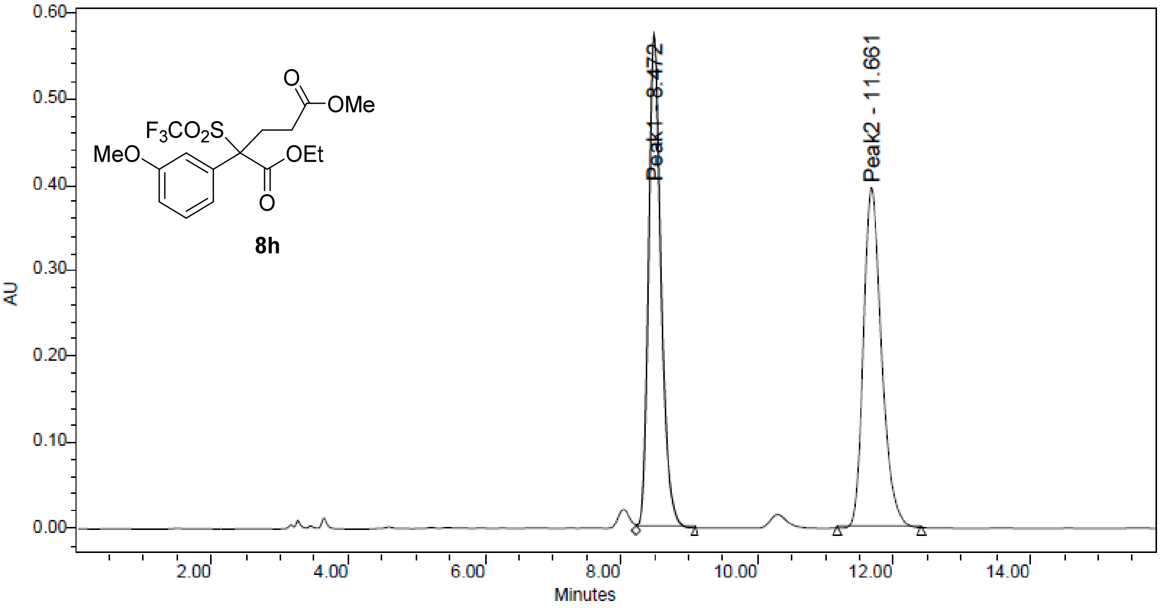

	Peak Name	RT (min)	Area (µV*sec)	% Area	Height (µV)	% Height
1	Peak1	8.472	7337100	49.93	575508	59.22
2	Peak2	11.661	7356659	50.07	396341	40.78

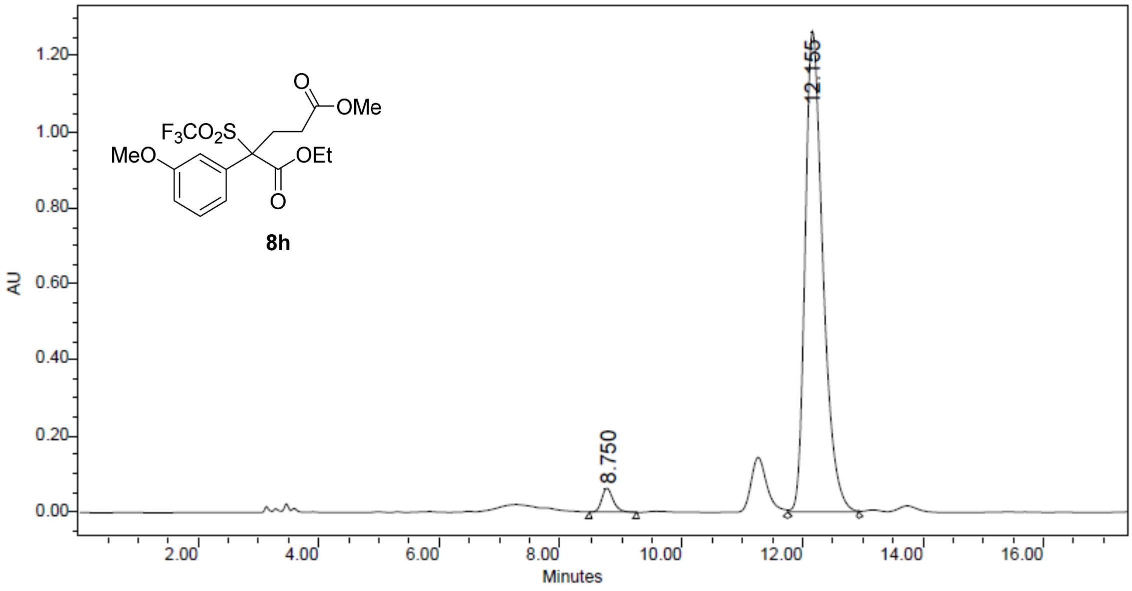

	RT (min)	Area (µV*sec)	% Area	Height (µV)	% Height
1	8.750	831772	3.09	63135	4.74
2	12.155	26079072	96.91	1269287	95.26

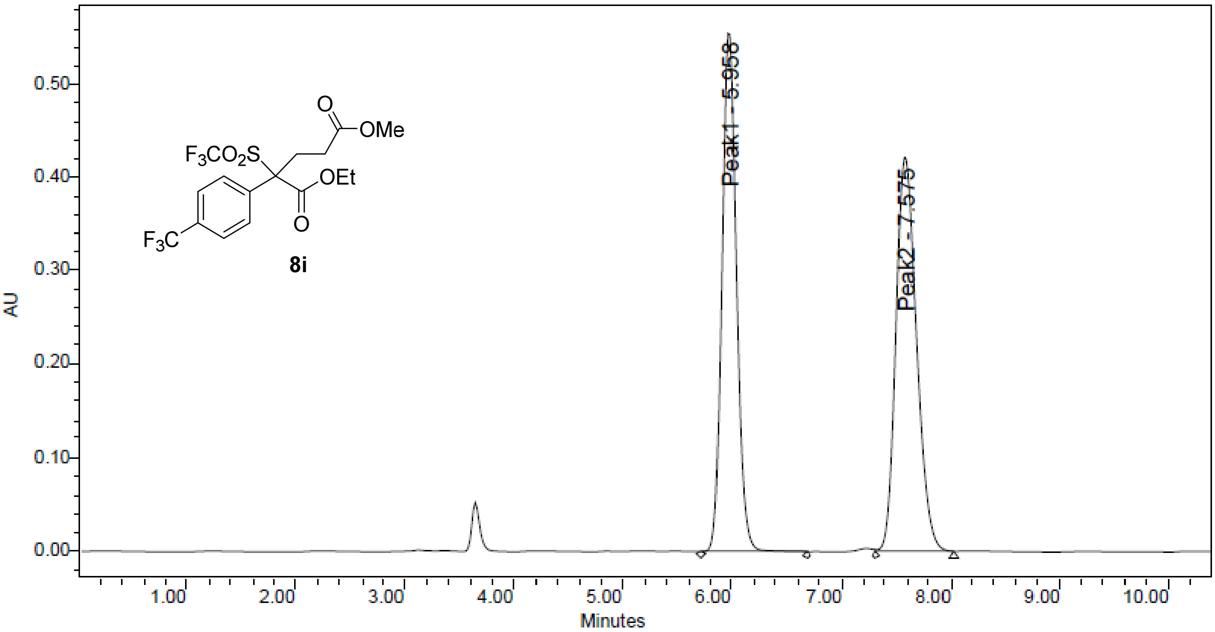

	Peak Name	RT (min)	Area (µV*sec)	% Area	Height (µV)	% Height
1	Peak1	5.958	5176937	48.29	560660	56.92
2	Peak2	7.575	5543405	51.71	424384	43.08

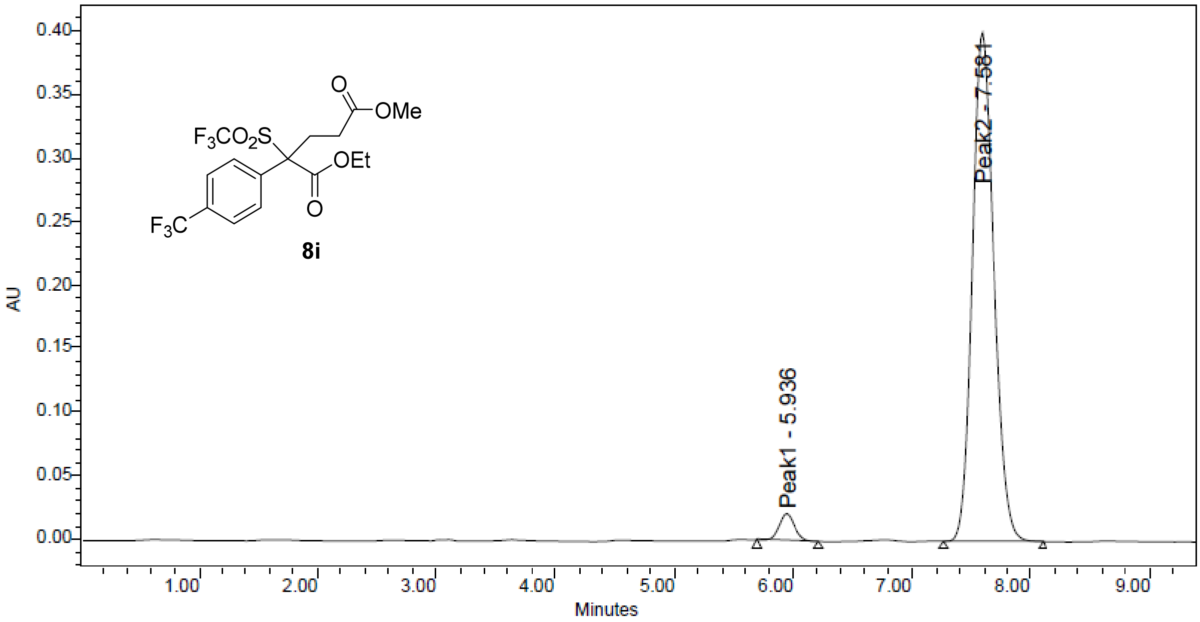

	Peak Name	RT (min)	Area (µV*sec)	% Area	Height (µV)	% Height
1	Peak1	5.936	191617	3.67	21177	5.03
2	Peak2	7.581	5029896	96.33	400001	94.97

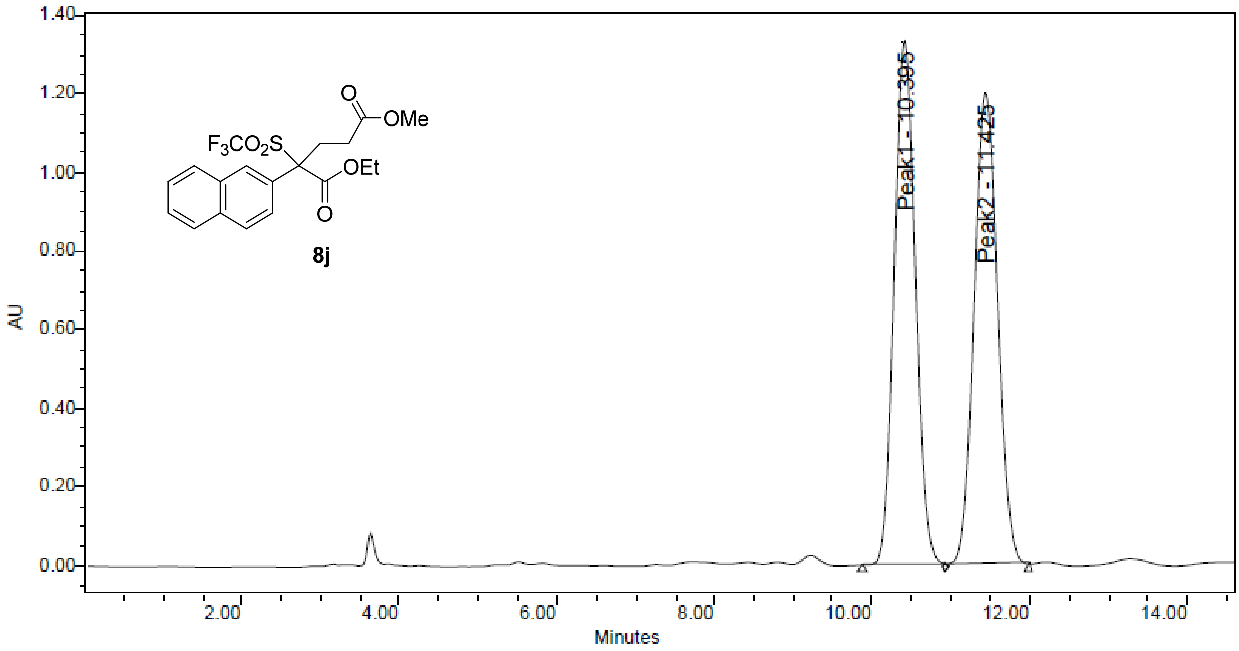

	Peak Name	RT (min)	Area (□V*sec)	% Area	Height (□V)	% Height
1	Peak1	10.395	24957774	50.01	1334299	52.64
2	Peak2	11.425	24950997	49.99	1200525	47.36

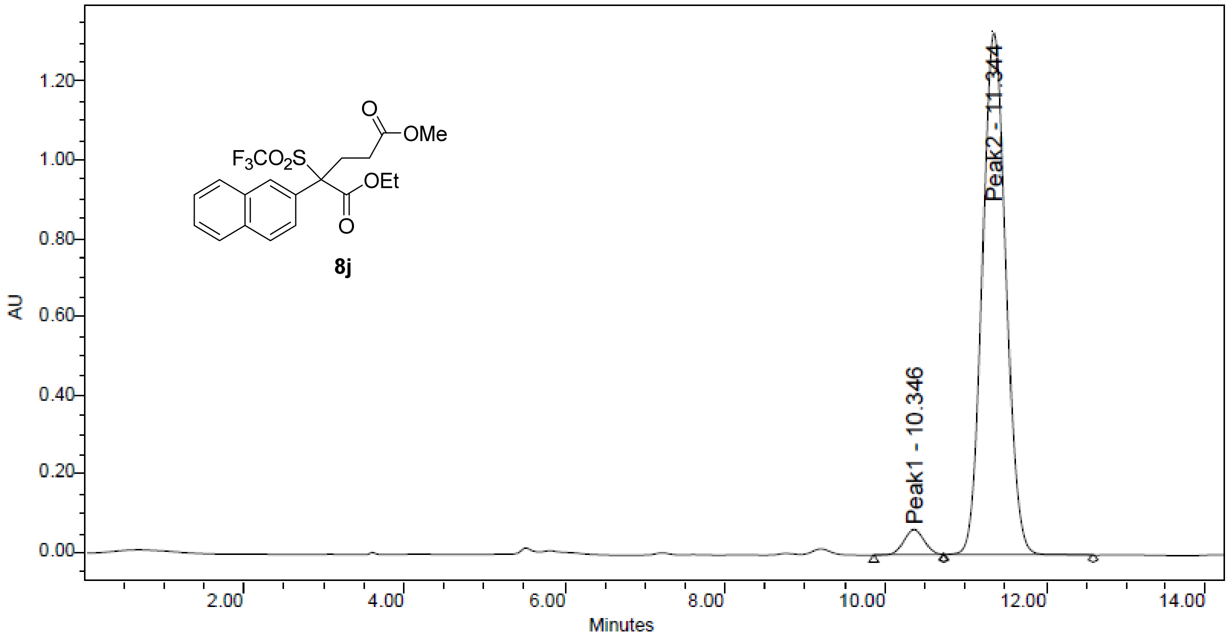

	Peak Name	RT (min)	Area (□V*sec)	% Area	Height (□V)	% Height
1	Peak1	10.346	1213692	4.24	66216	4.73
2	Peak2	11.344	27394076	95.76	1333132	95.27

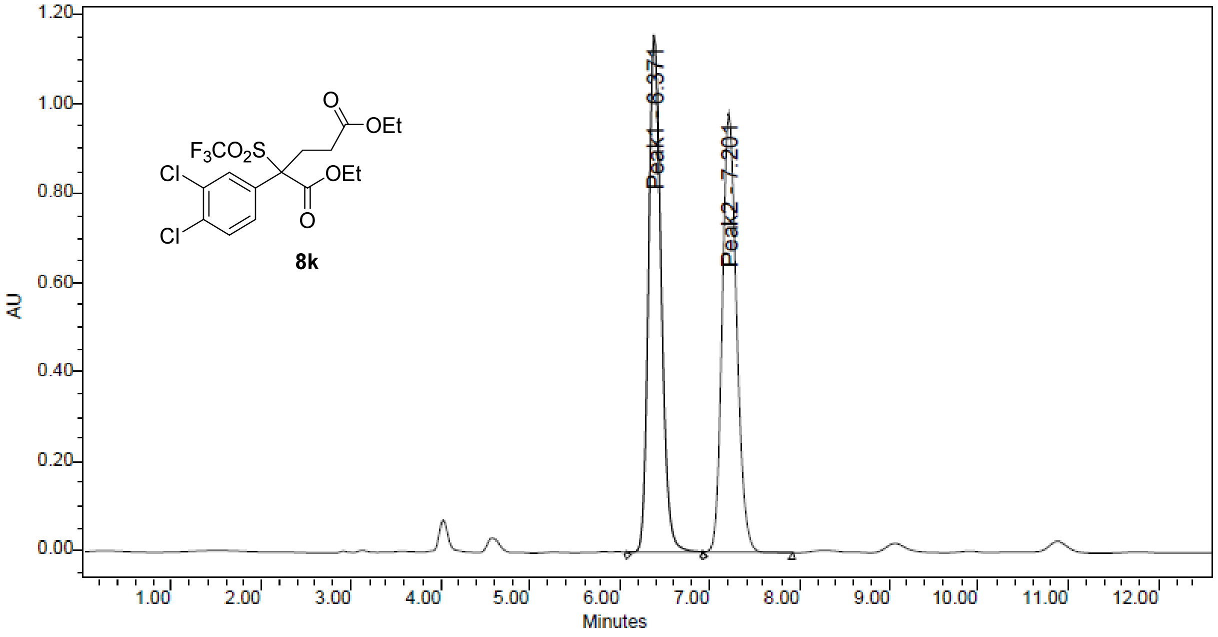

	Peak Name	RT (min)	Area (µV*sec)	% Area	Height (µV)	% Height
1	Peak1	6.371	11248957	50.39	1162301	54.16
2	Peak2	7.201	11076091	49.61	983757	45.84

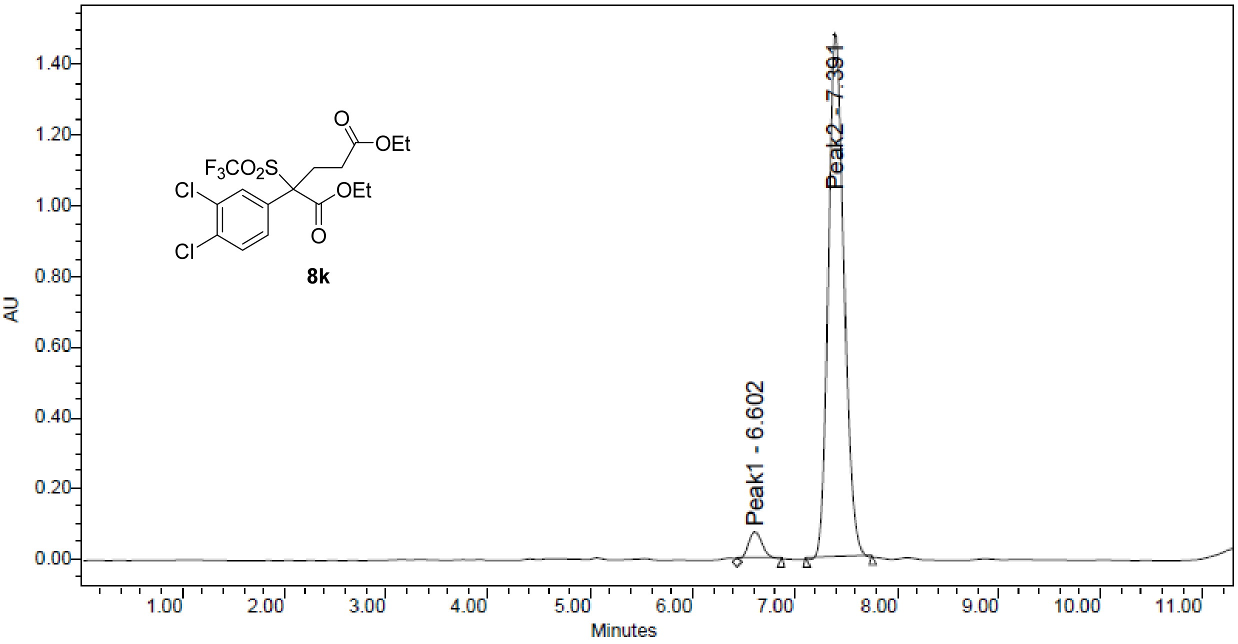

	Peak Name	RT (min)	Area (µV*sec)	% Area	Height (µV)	% Height
1	Peak1	6.602	749181	4.45	78449	4.99
2	Peak2	7.391	16093081	95.55	1494234	95.01

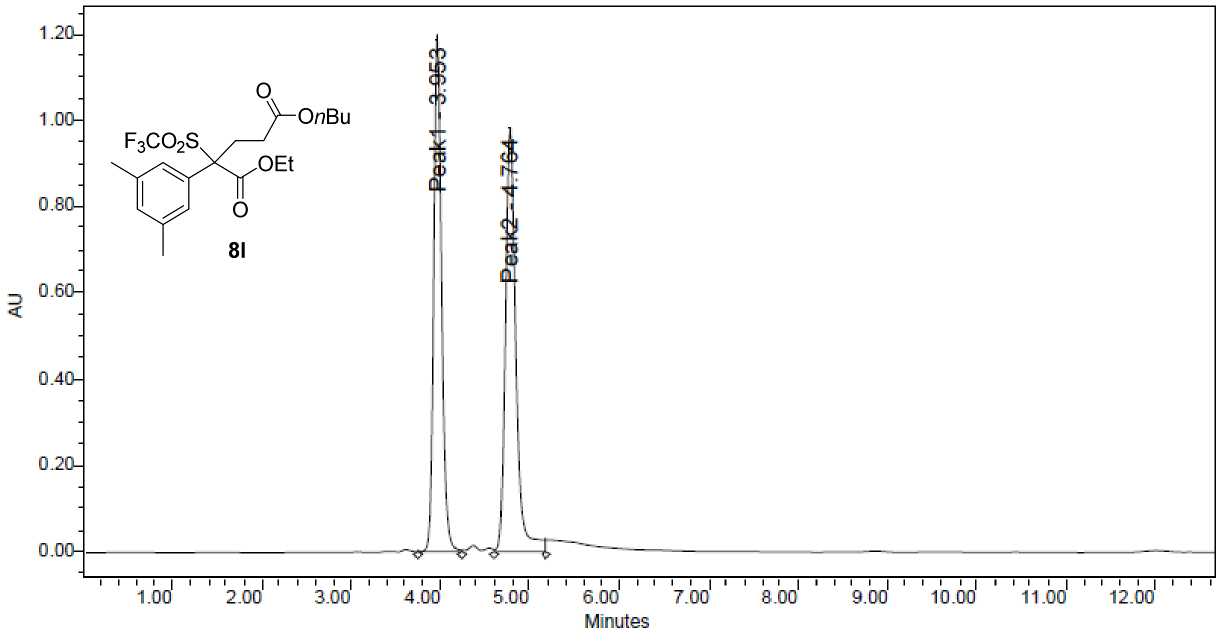

	Peak Name	RT (min)	Area (µV*sec)	% Area	Height (µV)	% Height
1	Peak1	3.953	7419304	48.10	1194141	54.84
2	Peak2	4.764	8005023	51.90	983167	45.16

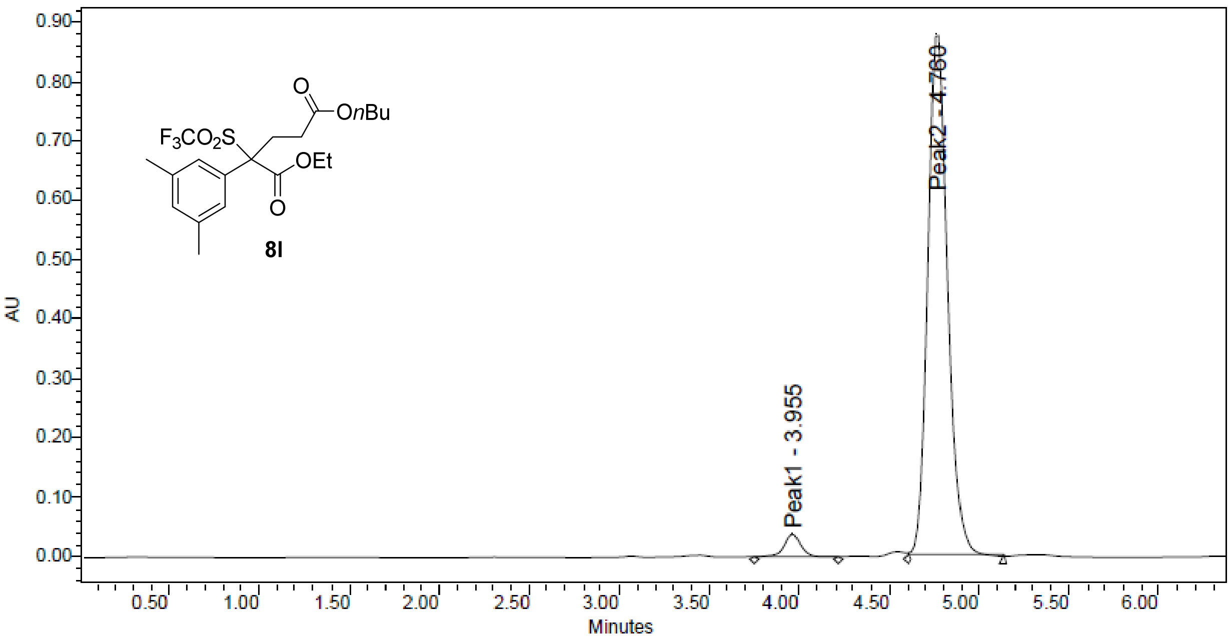

	Peak Name	RT (min)	Area (µV*sec)	% Area	Height (µV)	% Height
1	Peak1	3.955	251163	3.59	38016	4.12
2	Peak2	4.760	6747198	96.41	883828	95.88

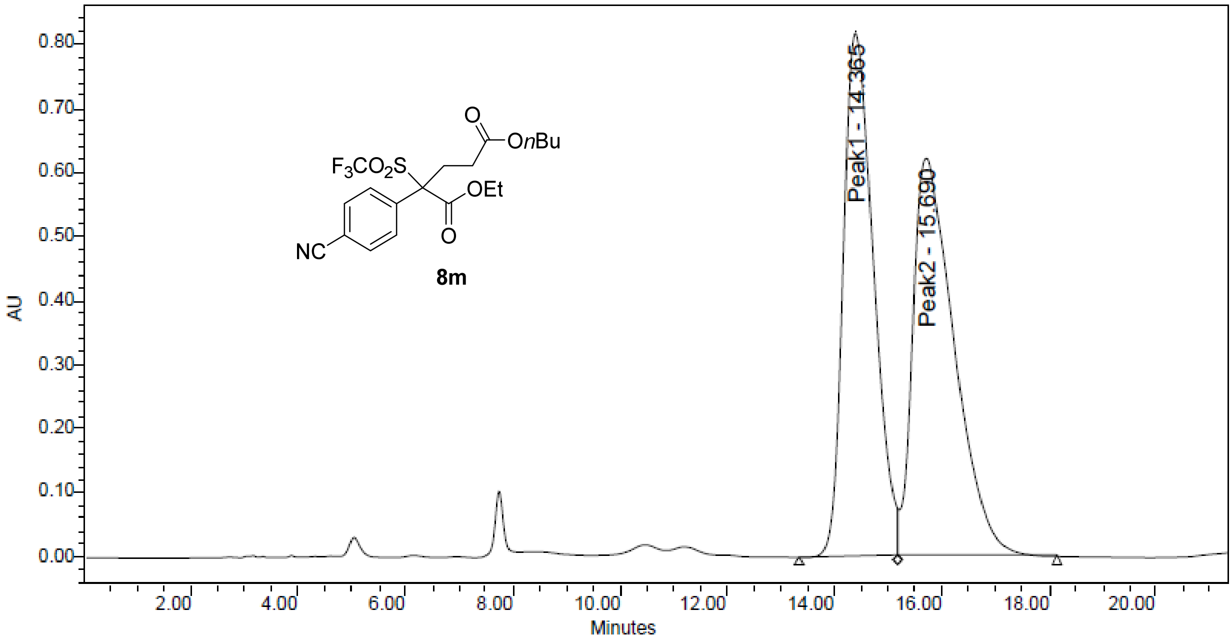

	Peak Name	RT (min)	Area (□V*sec)	% Area	Height (□V)	% Height
1	Peak1	14.365	32231056	49.07	817430	56.78
2	Peak2	15.690	33448025	50.93	622256	43.22

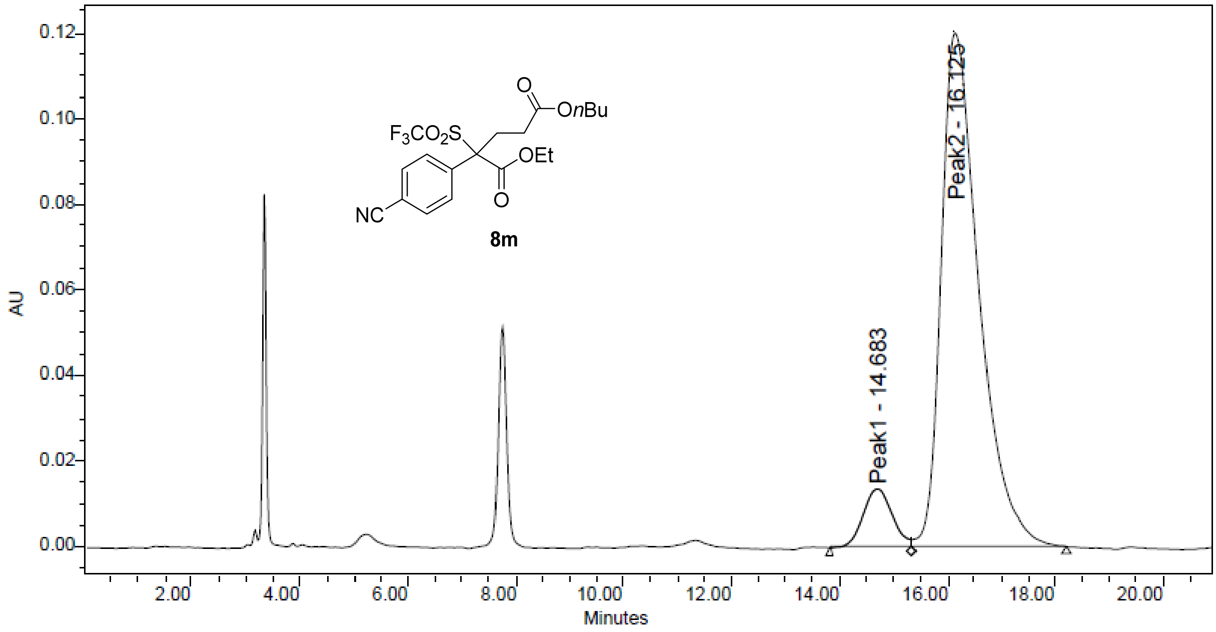

	Peak Name	RT (min)	Area (□V*sec)	% Area	Height (□V)	% Height
1	Peak1	14.683	521900	8.16	13649	10.20
2	Peak2	16.125	5877819	91.84	120210	89.80

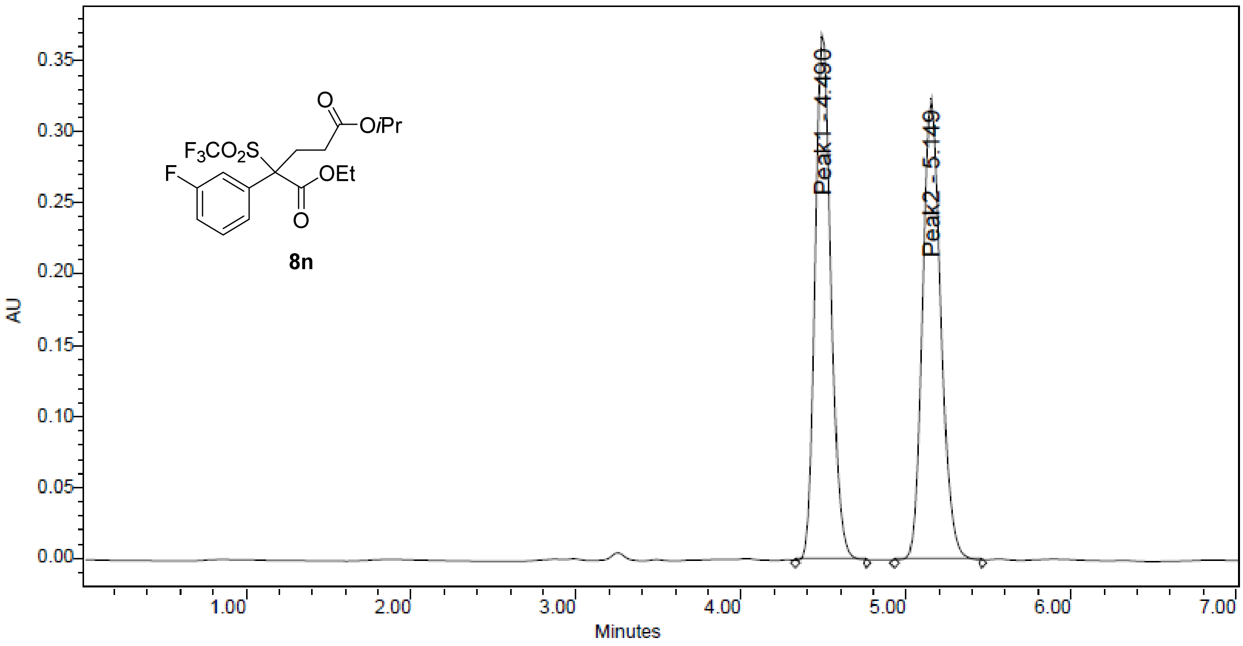

	Peak Name	RT (min)	Area (□V*sec)	% Area	Height (□V)	% Height
1	Peak1	4.490	2477574	49.66	370746	53.43
2	Peak2	5.149	2511319	50.34	323158	46.57

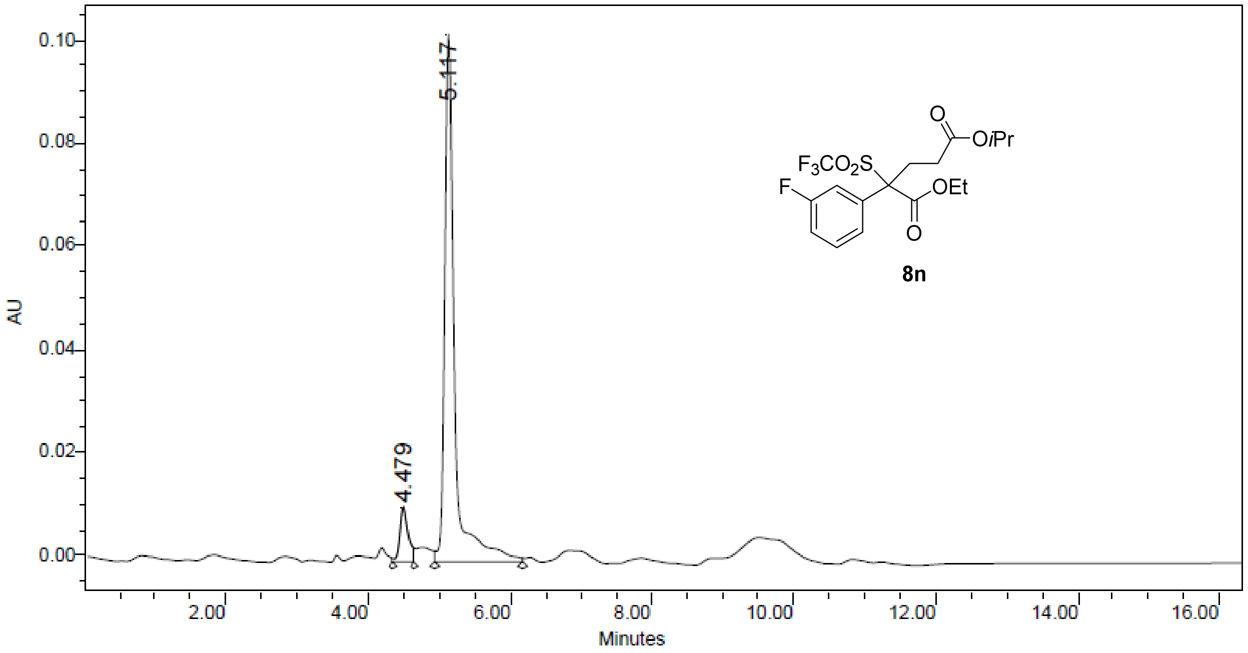

	RT (min)	Area (□V*sec)	% Area	Height (□V)	% Height
1	4.479	92948	8.65	10725	9.49
2	5.117	981280	91.35	102258	90.51

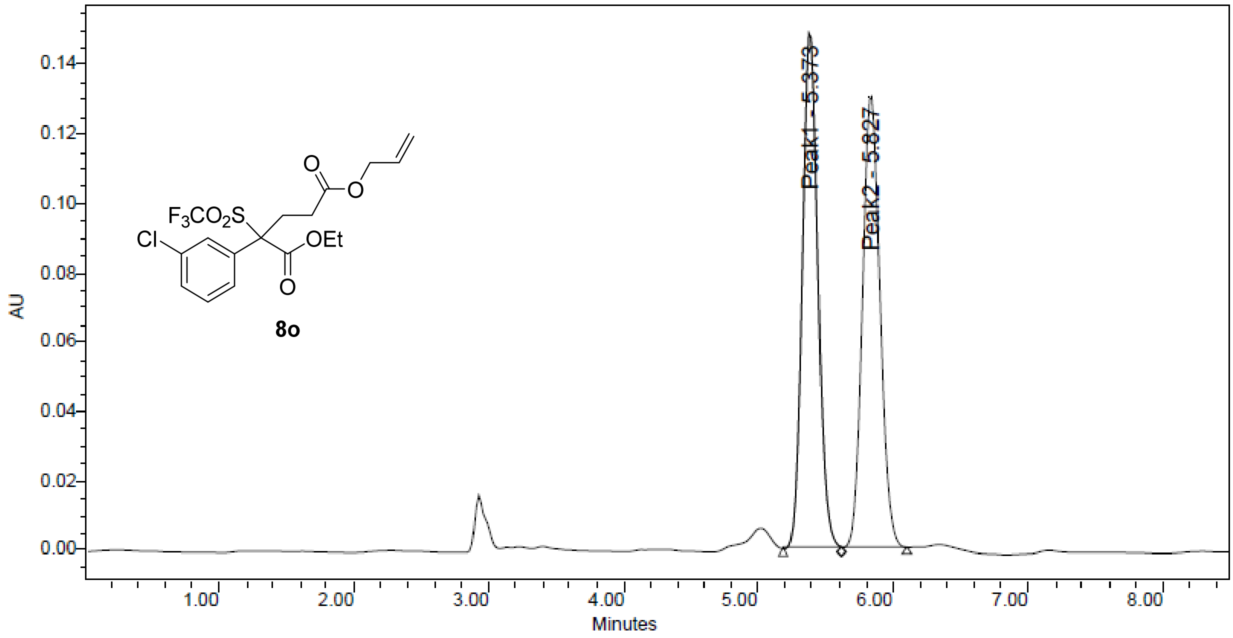

	Peak Name	RT (min)	Area (µV*sec)	% Area	Height (µV)	% Height
1	Peak1	5.373	1199288	50.40	149407	53.34
2	Peak2	5.827	1180276	49.60	130711	46.66

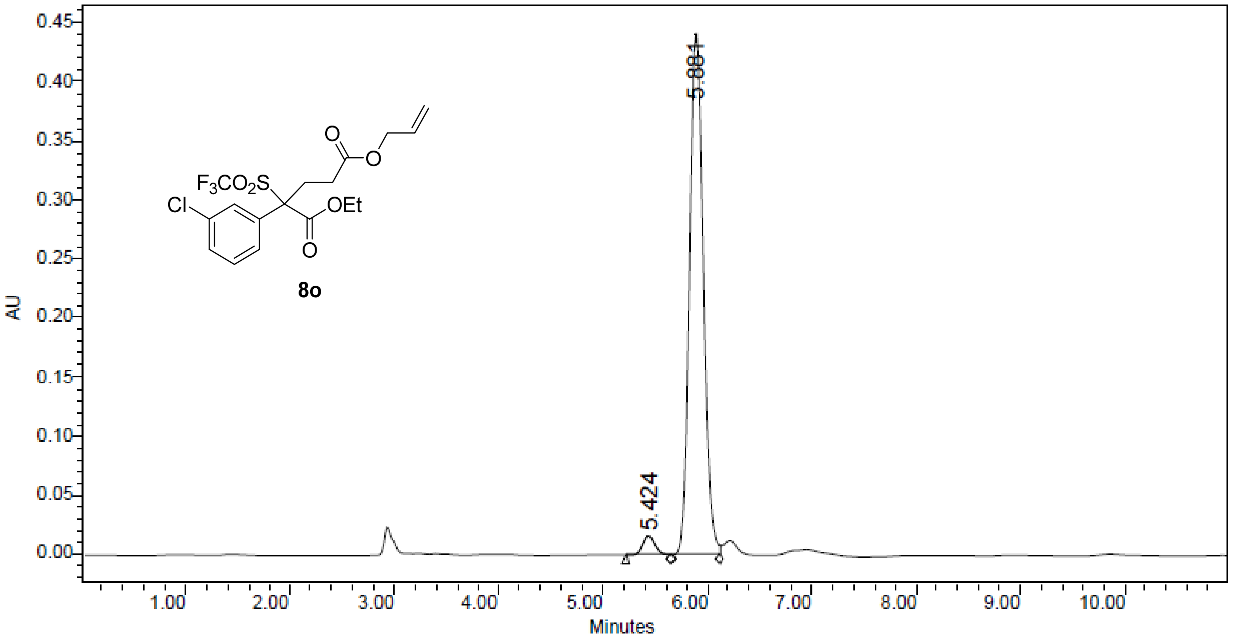

	RT (min)	Area (µV*sec)	% Area	Height (µV)	% Height
1	5.424	136524	3.26	16469	3.60
2	5.881	4047448	96.74	441565	96.40

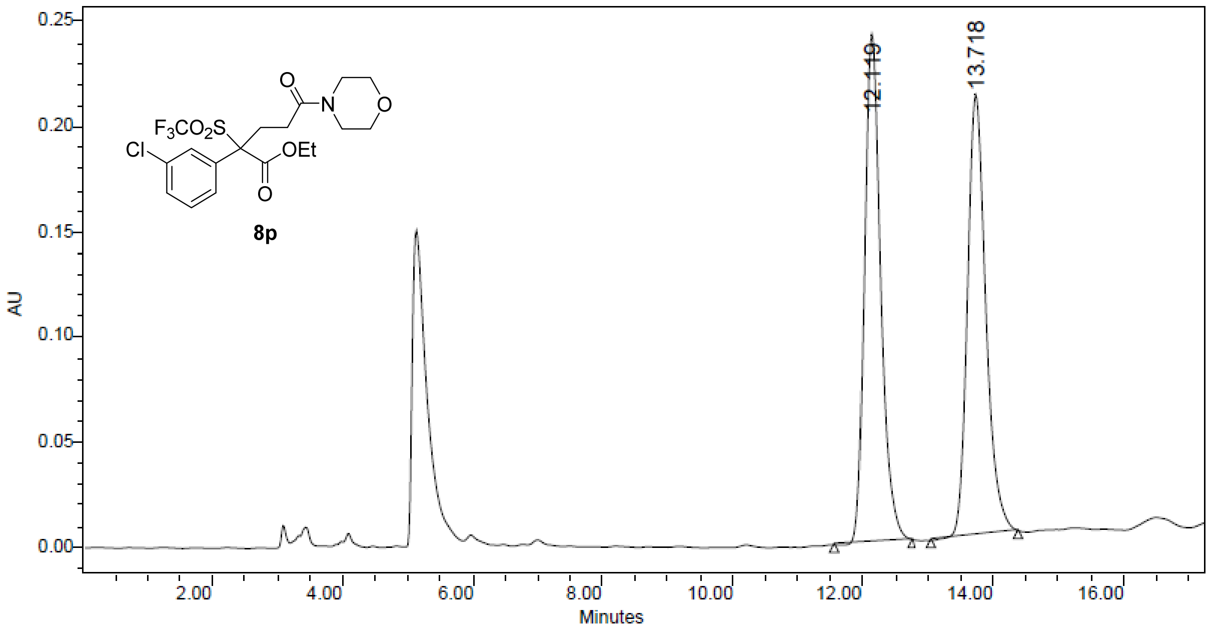

	RT (min)	Area (□V*sec)	% Area	Height (□V)	% Height
1	12.119	4173901	50.13	242026	53.55
2	13.718	4152883	49.87	209915	46.45

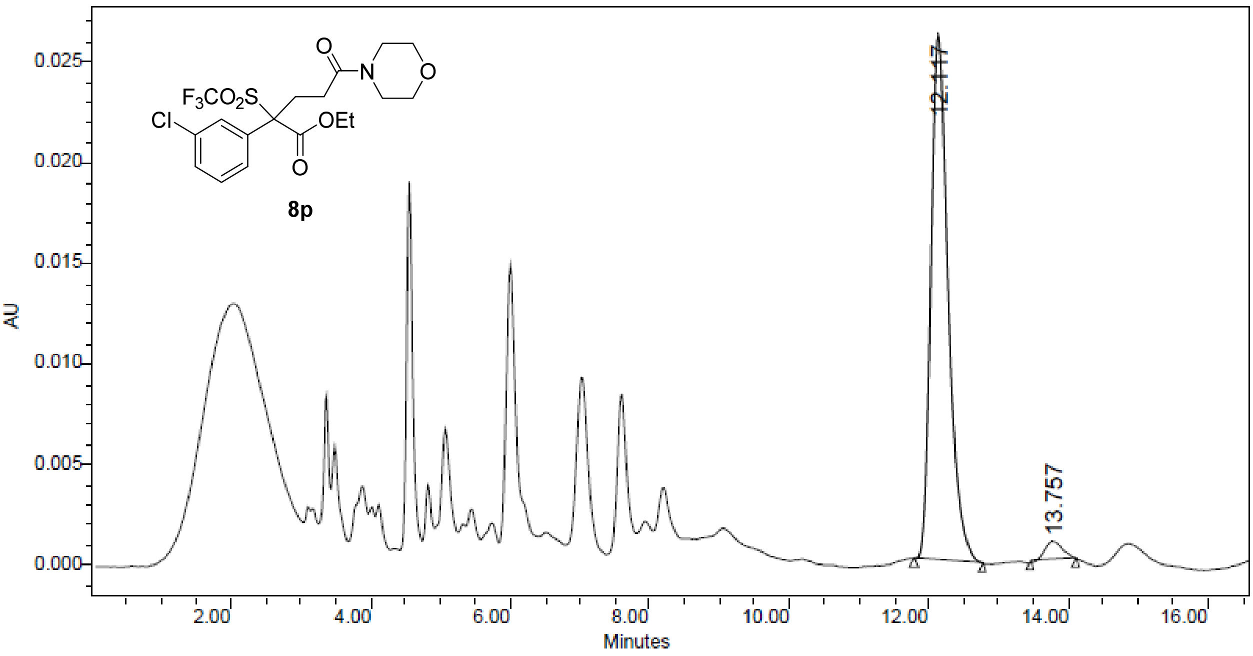

	RT (min)	Area (□V*sec)	% Area	Height (□V)	% Height
1	12.117	451020	96.29	26116	96.45
2	13.757	17358	3.71	961	3.55

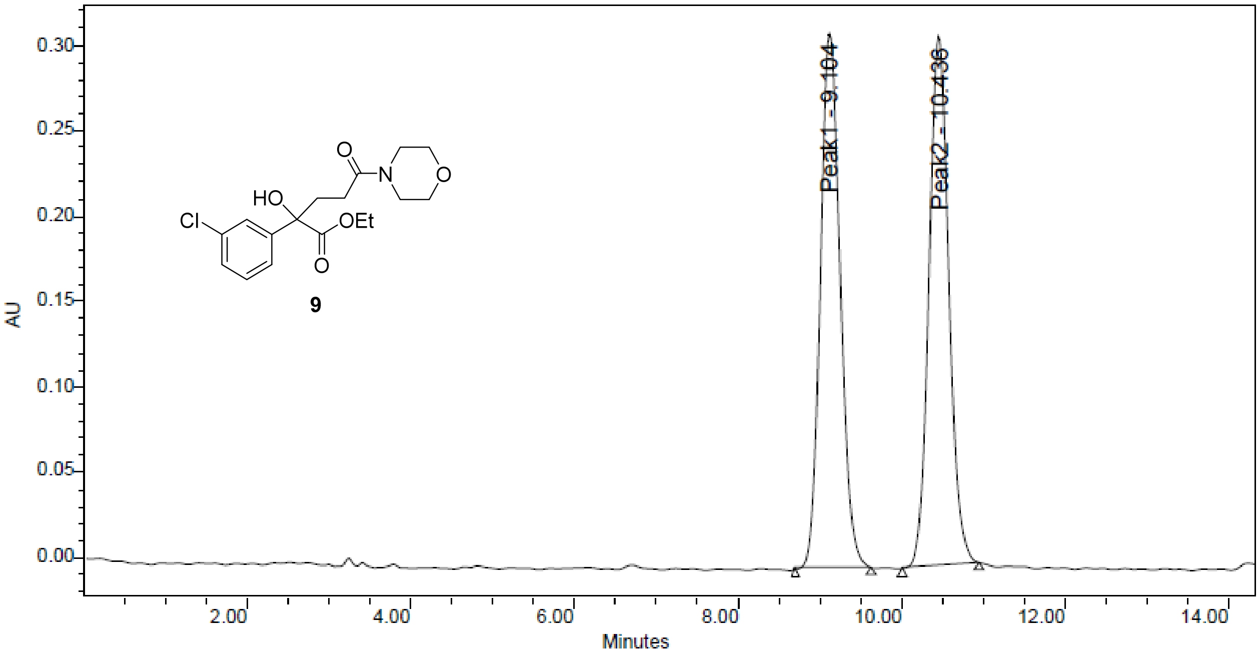

	Peak Name	RT (min)	Area (µV*sec)	% Area	Height (µV)	% Height
1	Peak1	9.104	5281250	50.25	313227	50.25
2	Peak2	10.436	5229484	49.75	310160	49.75

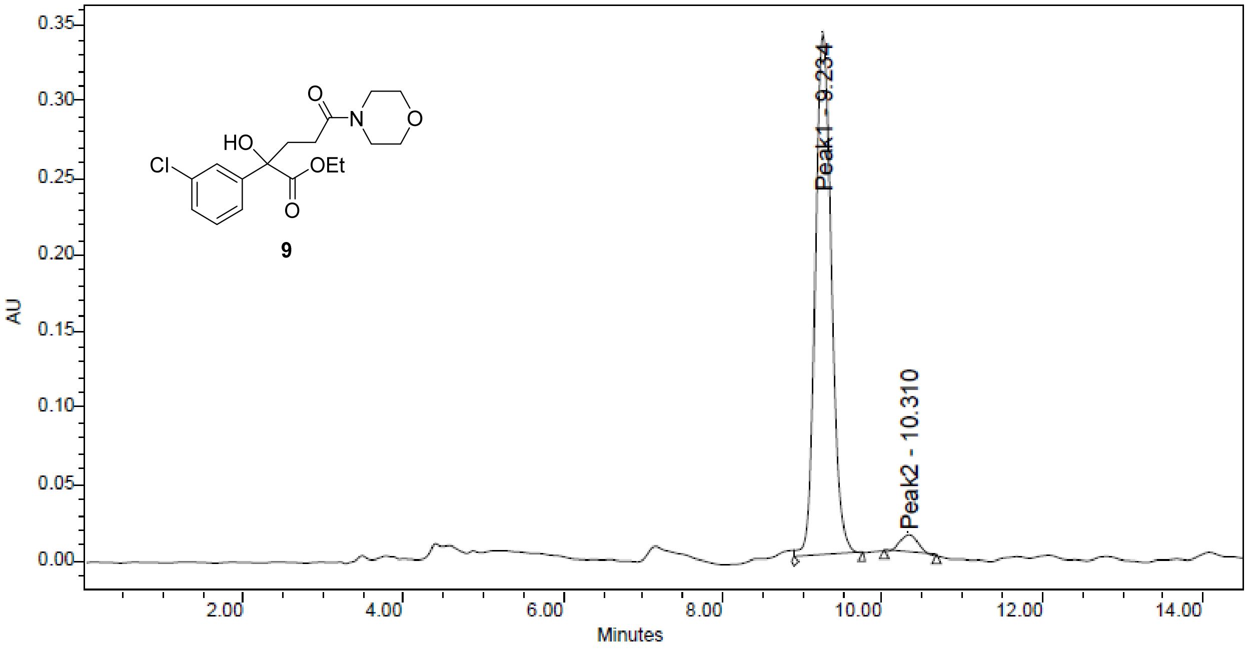

	Peak Name	RT (min)	Area (µV*sec)	% Area	Height (µV)	% Height
1	Peak1	9.234	4812496	95.99	341284	96.51
2	Peak2	10.310	201215	4.01	12346	3.49

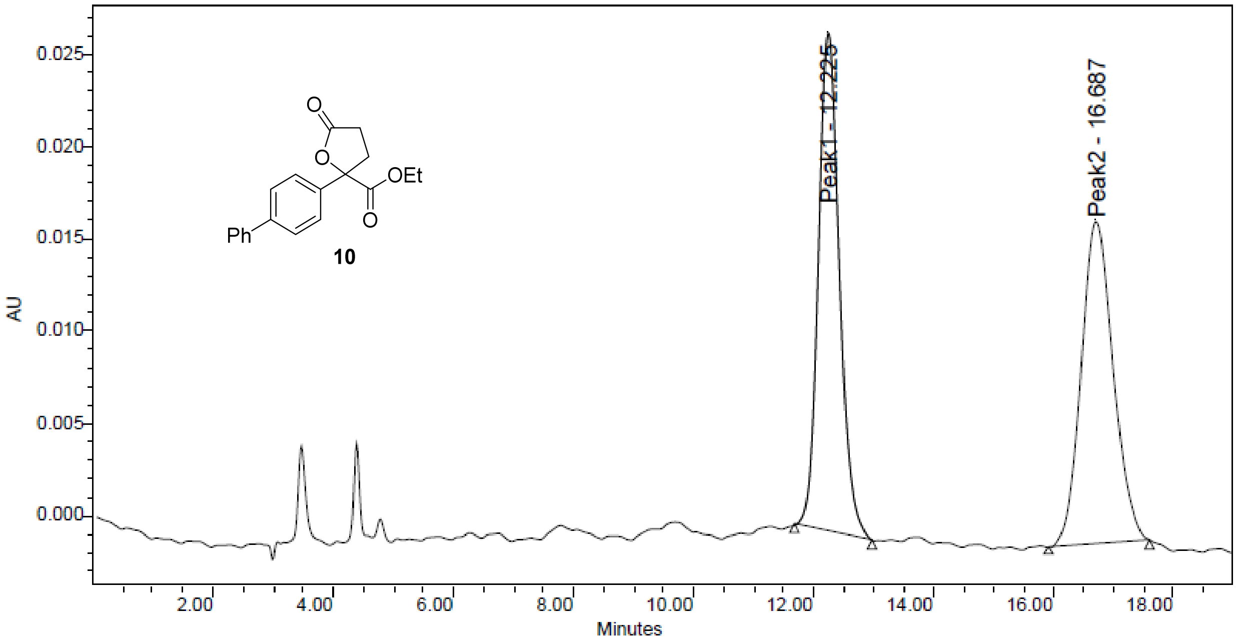

	Peak Name	RT (min)	Area (µV*sec)	% Area	Height (µV)	% Height
1	Peak1	12.225	658242	50.27	27032	60.73
2	Peak2	16.687	651117	49.73	17477	39.27

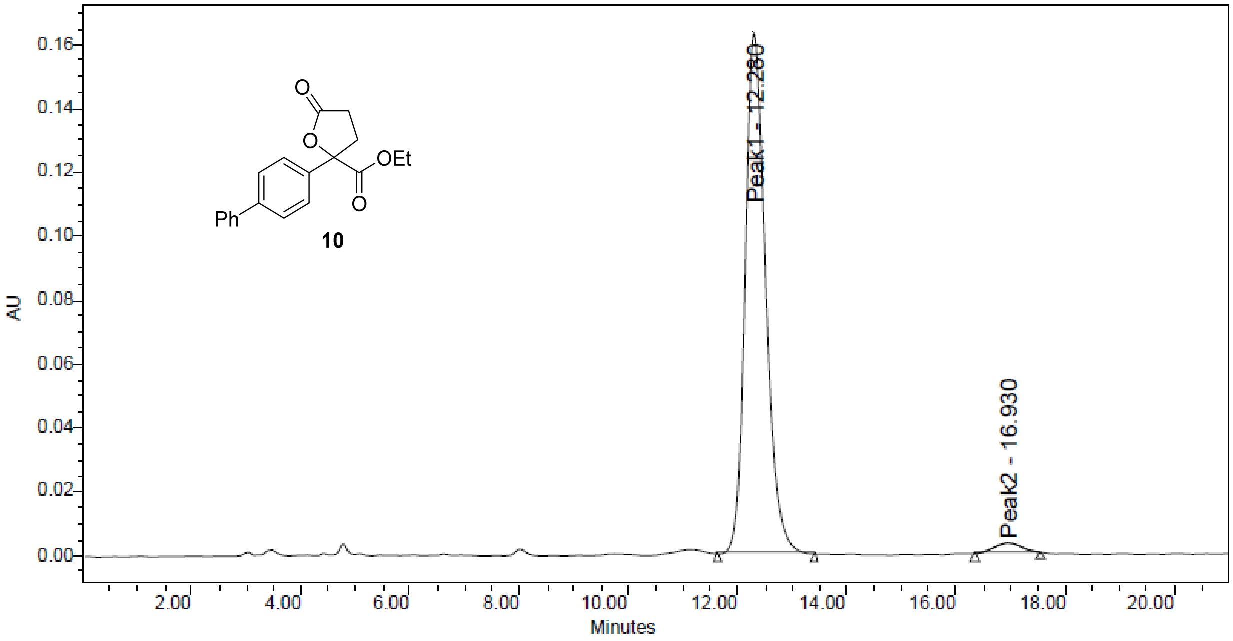

	Peak Name	RT (min)	Area (µV*sec)	% Area	Height (µV)	% Height
1	Peak1	12.280	4252877	97.46	163049	98.06
2	Peak2	16.930	111038	2.54	3221	1.94

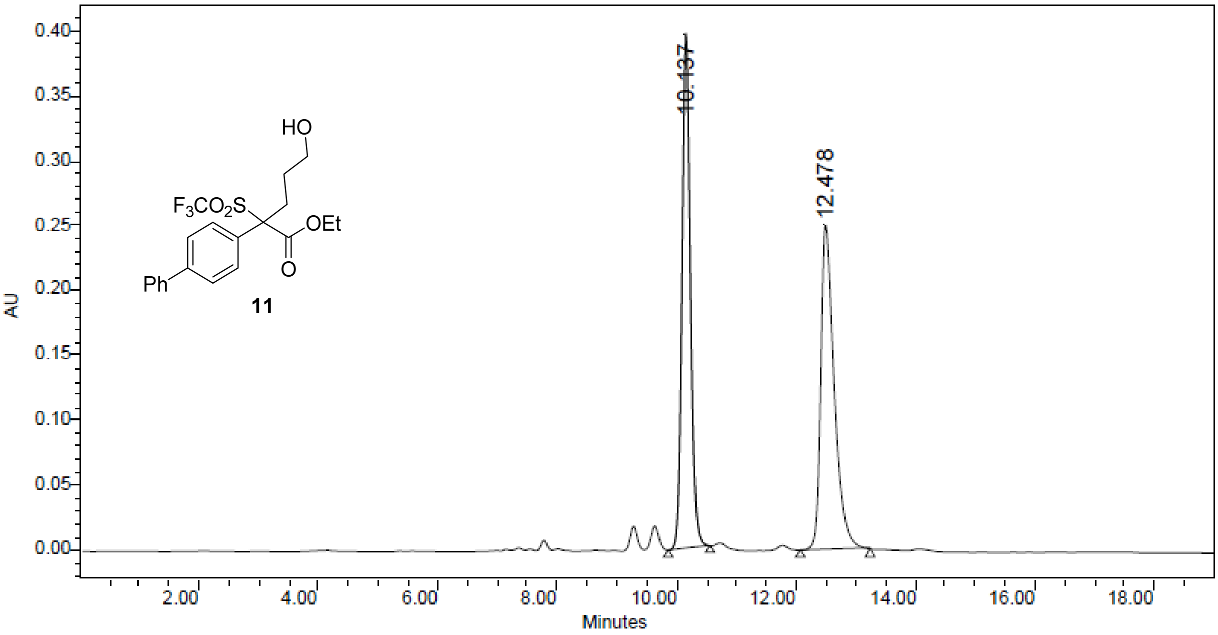

	RT (min)	Area (□V*sec)	% Area	Height (□V)	% Height
1	10.137	3923540	49.69	396974	61.36
2	12.478	3972164	50.31	249941	38.64

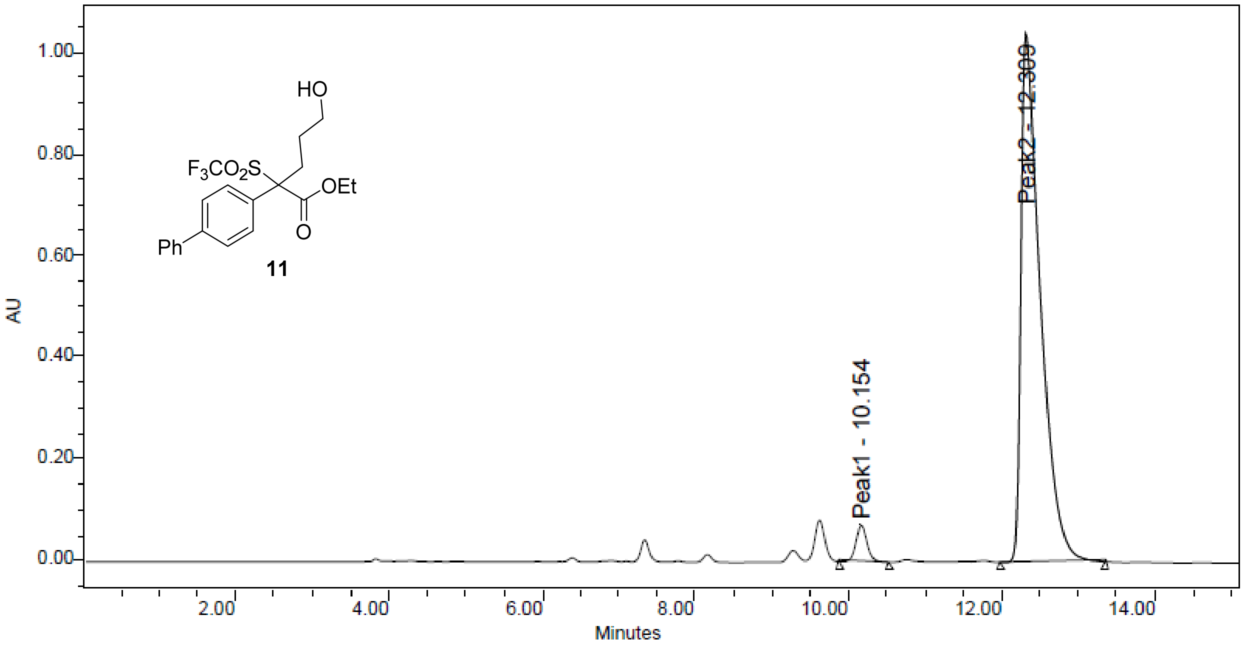

	Peak Name	RT (min)	Area (□V*sec)	% Area	Height (□V)	% Height
1	Peak1	10.154	715905	3.77	71612	6.41
2	Peak2	12.309	18294142	96.23	1045918	93.59

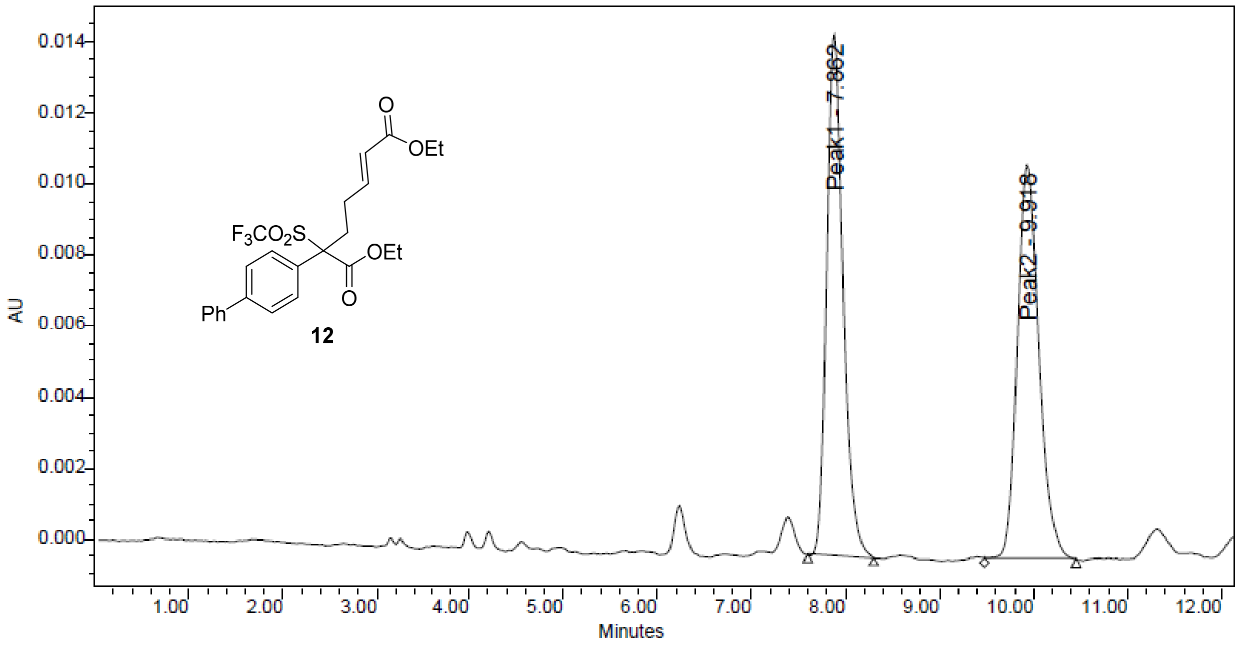

	Peak Name	RT (min)	Area (µV*sec)	% Area	Height (µV)	% Height
1	Peak1	7.862	179241	49.99	14715	56.96
2	Peak2	9.918	179312	50.01	11118	43.04

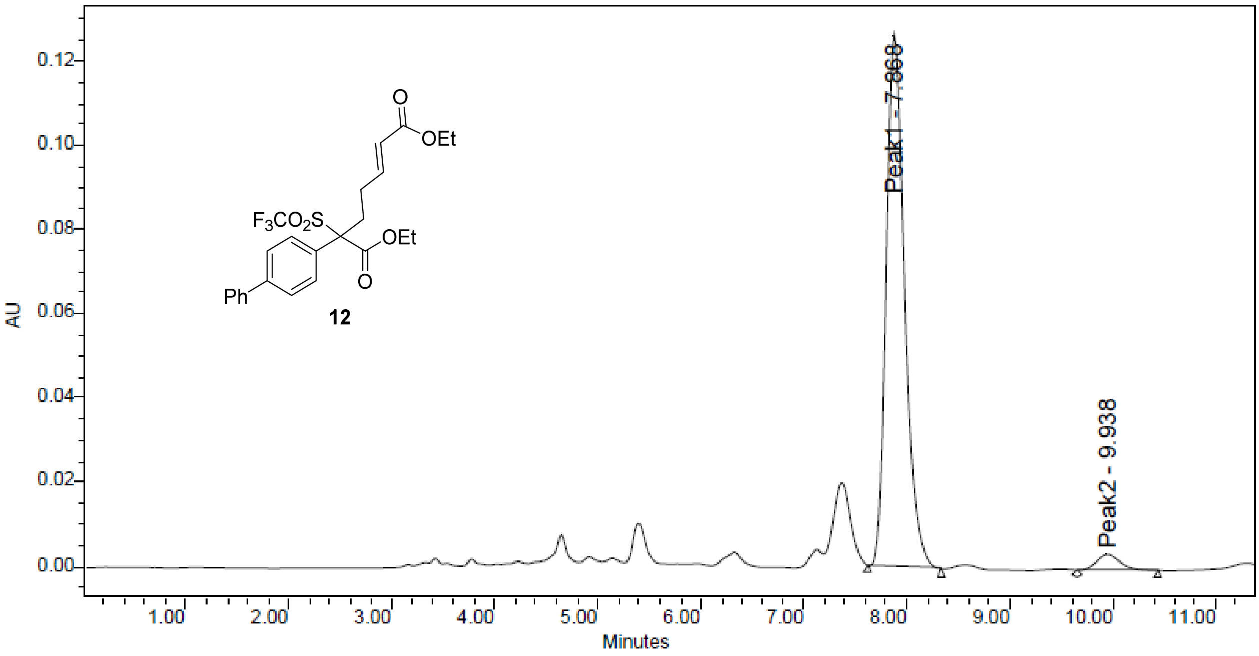

	Peak Name	RT (min)	Area (µV*sec)	% Area	Height (µV)	% Height
1	Peak1	7.868	1564996	96.20	126421	97.04
2	Peak2	9.938	61845	3.80	3859	2.96
